# An integrated network pharmacology and proteomics approach reveals the anti-fibrotic effect of Fushen Granule on peritoneal fibrosis

**DOI:** 10.1186/s12906-026-05333-2

**Published:** 2026-03-09

**Authors:** Kang Yang, Jie Li, Lin Wang, Hangxing Yu, Xinyue Liu, Zhiqing Gao, Zheng Wang, Linqi Zhang, Hongtao Yang

**Affiliations:** 1 Department of Nephrology, The First Affiliated Hospital of Henan University of Chinese Medicine, Zhengzhou, China; 2 Department of Nephrology, First Teaching Hospital of Tianjin University of Traditional Chinese Medicine, Tianjin, China; 3 Department of Nephrology, Chongqing Hospital of Traditional Chinese Medicine, Chongqing, China

**Keywords:** Fushen Granule, Network pharmacology, Proteomics, Peritoneal dialysis, PI3K/AKT signalling

## Abstract

**Background:**

Fushen Granule (FSG), a Chinese medicine formular, was used clinically to improve the efficiency of dialysis in end-stage renal disease patients receiving peritoneal dialysis treatment. However, the mechanisms of its antifibrotic effect on peritoneal fibrosis (PF) have not yet been studied. In this study, we aimed to identify the potential mechanism of FSG in intervening in PF.

**Methods:**

Network pharmacology analysis and molecular docking were used to predict the related ingredients and potential targets of FSG in treating PF. TGF-β1 induced MeT5A cells were used for in vitro verification. Label-free proteomics analysis was performed to detect the differentially expressed proteins in TGF-β1 induced MeT5A cells upon treatment with FSG-containing serum. Western blot and immunofluorescence assays were used to evaluate the expression of proteins related to PF and PI3K/AKT signalling.

**Results:**

PPI network analysis showed that TP53, EGFR, HSP90AA1, AKT1, CCND1, MYC, STAT3, SRC and ESR1 constitute the core network. KEGG analysis found that the PI3K/AKT signalling pathway was significantly enriched. Molecular docking analysis indicated that the main active ingredient from the component–target network can closely integrate with targets in the PI3K/AKT signalling pathway. Proteomics analysis on TGF-β1-treated MeT5A also indicated that the PI3K/AKT signalling pathway was closely correlated with the effect of FSG in intervening PF. Further in vitro experimental validation showed that FSG decreased the protein levels of FN, Col Ⅰ, α-SMA, ITGβ3, PDK1, p-mTOR and p-AKT and reversed the expression of E-cad in TGF-β1-treated MeT5A cells.

**Conclusion:**

FSG showed therapeutic effects against PF, mainly by inactivating PI3K/AKT signalling.

**Supplementary Information:**

The online version contains supplementary material available at 10.1186/s12906-026-05333-2.

## Introduction

 The incidence of chronic kidney disease (CKD) is increasing worldwide, placing a substantial burden on public health and healthcare systems, particularly in China where the economic cost of CKD management is escalating [1, 2]. Approximately 2% of patients progress to end-stage renal disease (ESRD) annually and need renal replacement therapy [3]. Peritoneal dialysis (PD) is an effective renal replacement therapy that has been widely used for ESRD for several decades [4]. Currently, approximately 272,000 patients receive dialysis treatment, accounting for 11% of the dialysis population [5]. The annual growth rate of patients undergoing peritoneal dialysis is 8%, surpassing that of haemodialysis (HD), which ranges from 6 to 7% [3]. Compared with haemodialysis, PD patients have more stable haemodynamics, better protection of residual renal function and a higher survival rate, leading to a better nutritional status and a higher quality of life [6–9]. However, the occurrence of peritoneal fibrosis (PF) during long-term PD treatment can lead to the failure of PD, which is the main reason for dialysis failure [10].

The main feature of PF is the abnormal accretion of the extracellular matrix (ECM) in the peritoneal membrane. Early signs of PF can be observed in 50–80% of patients within the first two years of PD treatment [11]. In the course of long-term PD, the biocompatibility of a PD solution emerges as a primary factor contributing to peritoneal structural and functional impairment and inducing the occurrence of PF [12]. Clinical and experimental studies have shown that microbial infection, chronic inflammation and exposure to high glucose and uremic toxin conditions can damage the peritoneal membrane, leading to the development of PF [13, 14]. Studies have shown that PF is closely related to the epithelial–mesenchymal transition (EMT) of mesothelial cells (MCs), in which MCs become trans-differentiated into the myofibroblast phenotype and acquire an ECM-secreting function [15, 16]. Among the growth factors and cytokines involved in the EMT process, transforming growth factor β1 (TGF-β1) is extremely important in PD-induced PF [17, 18]. Moreover, the levels of TGF-β1 and mesenchymal markers have increased in effluent-derived MCs from patients with long-term PD [19]. Based on this background, studies have found that inhibiting TGF-β1 signal transduction can delay the progress of PF [20–22]. Although progress has been made in profiling the effect of TGF-β1 on the development of PF, the precise mechanisms initiating and sustaining PF remain incompletely elucidated, and there is currently no generally accepted effective treatment for PF.

Traditional Chinese medicine (TCM) is attracting increasing attention globally for its reliable curative effect and safety. Studies have reported that traditional herbal formulas, including *Buyang Huangwu* decoction, *Liuwei Dihuang* pills and *Yinchenhao* decoction, can act against tissue fibrosis [23–25]. Nevertheless, only a few studies have focused on the treatment of PF. Based on our long-term clinical experience, our team developed a TCM formula for Fushen Granule (FSG; compositions of the formula are shown in Table 1) that was approved as a hospital preparation for the treatment of PD-related complications. We found that FSG can ameliorate intestinal mucosal dysfunction and mitigate inflammation in PD rats by inactivating P38 MAPK signalling [26]. The quality of PD treatment mainly depends on the function of the peritoneal tissue. We found FSG can inhibit peritoneal tissues proliferation and inflammatory cell infiltration in PF rats [27]. The mRNA levels of α-SMA and vimentin and protein level of TGF-β1 in PF rats were decreased upon FSG treatment [28, 29].Our clinical study showed that FSG could improve dialysis efficiency and nutritional status in patients receiving PD treatment, which indicates the protective role of FSG on peritoneal MCs and enables delaying the occurrence of PF [30].

**Table 1:** Composition of traditional Chinese medicine in FSG extract

TCM	Scientific Name	Part Used		Amount %
Huang Qi	Astragalus mongholicus Bunge [Leguminosae]		Root	11.1%
Dang Gui	Angelica sinensis (Oliv.) Diels [Apiaceae]		Root	7.4%
Yin Yang Huo	Epimedium macranthum C.Morren & Decne. [Berberidaceae]		Leaf	11.1%
Chen Pi	Citrus reticulata Blanco [Rutaceae]		Peel	7.4%
Ban Xia	Pinellia ternata (Thunb.) Makino [Araceae]		Rhizome	11.1%
Dan Shen	Salvia miltiorrhiza Bunge [Lamiaceae]		Root	22.2%
Da Huang	Rheum officinale Baill [Polygonaceae]		Rhizome	7.4%
Gui Jian Yu	Euonymus alatus (Thunb.) Siebold [Celastraceae]		Winged stem	22.2%
Total				100%

Since herbal medicine treats diseases via multitarget and multidirectional mechanisms, it is hard to uncover a concrete pharmacological mechanism via a single detection method. Some researchers have proposed network pharmacology to address these issues because network pharmacology can elucidate the interaction between diseases and drugs through target prediction and pharmacophore interaction analysis [31, 32]. In addition, proteomics technology offers the possibility of exploring the mechanism of herbal medicine in treating diseases. Large-scale data from proteomics can be processed quickly and efficiently with the development of bioinformatics analysis. Hence, it is feasible to elucidate the potential pathogenesis of PF and to find intervention targets by combining these two methods. In this study, network pharmacology and label-free quantitative proteomics were used to reveal the mechanisms of FSG in delaying PF, followed by further verification using in vitro experiments.

## Materials and methods

### Screening of the potential targets of FSG-mitigated PF

All ingredients of FSG were acquired from TCMSP (https://www.tcmsp.com/) by oral bioavailability (OB) ≥ 30% and druglikeness (DL) ≥ 0.18. Then, the targets of the screened compounds above were predicted via TCMSP and the SwissTargetPrediction tool (http://swisstargetprediction.ch/). Targets with a probability ≥ 30% were selected. Peritoneal fibrosis-associated genes were retrieved from GeneCards (https://www.genecards.org/), OMIM (https://omim.org/), PharmGKB (https://www.pharmgkb.org/), TTD (https://db.idrblab.net/ttd/) and CTD (http://ctdbase.org/). The intersecting target genes were obtained from FSG and PF using a Venn diagram (https://bioinfogp.cnb.csic.es/tools/venny/index.html, version 2.1.0). Then, the component–target network of the FSG delaying PF was mapped by Cytoscape.

### Construction of the protein–protein interaction network and critical sub-network

The protein–protein interaction (PPI) network of overlapped genes was acquired from the STRING website (https://string-db.org/). The core subnetwork was obtained by a two-step topological analysis using the CytoNCA plugin in Cytoscape. In detail, each score of DC, BC, CC, EC, NC and LAC higher than the median value was chosen to map the critical subnetwork.

### GO and KEGG analyses of intersectant genes

GO and KEGG analyses of the overlapped genes were performed using the R4.1.1 software. GO enrichment mainly analyses the biological process (BP), cellular composition (CC), and molecular function (MF). KEGG enrichment analyses the possible signalling pathways related to the overlapped genes.

### Molecular docking of core compounds and target genes

Molecular docking between the main active compounds of FSG and the core protein receptors in the pathway examined in this study was performed. Specifically, the structure of the main effective ingredients was searched in Pubchem (https://pubchem.ncbi.nlm.nih.gov/). The 3D structure of key proteins was acquired from PDB (https://www.rcsb.org/). The optimal binding models were visualised using the AutoDock Vina software and the PyMol software. Binding energy of < -5.0 kcal/mol indicated that the active ingredient can bind firmly with the protein receptor. Binding energy of < -7.0 kcal/mol illustrates that the ligand has a stronger binding ability.

### Chemicals and reagents

Recombinant human TGF-β1 (100-21-2) was purchased from PeproTech (USA). Medium 199 (M199, 01-080-1ACS) and foetal bovine serum (FBS, 04-001-1 C) were purchased from Biological Industries (Israel). Antibodies against fibronectin (FN) (15613-1-AP), Col Ⅰ (14695-1-AP), E-cadherin (20874-1-AP), ITGβ3 (18309-1-AP), PDK1 (10026-1-AP), mTOR (66888-1-Ig), p-AKT (66444-1-Ig), AKT (10176-2-AP), GAPDH (10494-1-AP), rabbit IgG (SA00001-15) and fluorescein rabbit IgG (SA00003-2) were obtained from Proteintech (China). α-SMA (ab5694) and p-mTOR (ab137133) were purchased from Abcam (England, UK).

### Animals

Forty male SD rats aged 8–10 weeks were purchased from Beijing Huafukang Bioscience Co., Ltd. Animals were housed in a specific pathogen-free condition with a normal light-dark cycle. All rats were provided free access to standard food and water and were maintained for 1 week before the experiment as an acclimatisation period. The study protocol was approved by the Animal Research Committee at Tianjin University of Traditional Chinese Medicine (approval no. TCM-LAEC2019097).

### Preparation of FSG-containing serum

FSG (batch no. 140928) was provided and controlled for quality by the First Teaching Hospital of Tianjin University of TCM. It comprised eight commonly used Chinese medicines in specific proportions **(**Table 1**)**. In this preparation, 1 g of prepared FSG powder was equivalent to 3.75 g of original crude herbs. In our previous studies, we tentatively identified 55 chemical compounds in FSG using UPLC/Q-TOF analysis, and the dose of FSG employed in this study also refers to these prior investigations [33, 34]. Concretely, the powder extract of FSG was dissolved with an appropriate amount of water and diluted to a final concentration of 0.54 g/ml (equivalent to 2.02 g/ml of crude herbs). To obtain drug-containing serum, all rats were randomly and equally divided into control and FSG groups (*n* = 15 per group). The rats in the FSG group were orally administered FSG (9 ml/kg/d) continuously for 7 days, and those in the control group were given an equal volume of physiological saline. All animals were euthanized by intraperitoneal injection of pentobarbital sodium (40 mg/kg body weight) 2 h after the last administration. Rat blood was acquired and separated by centrifugation, filtered with 0.2 μm microporous filter membrane and then preserved at -80 °C for further use.

### Cell culture and treatments

The immortalised mesothelial cell line MeT5A was purchased from ATCC (Lot. 63990138). The PF model-building method was based on a previous report [35]. Generally, MeT5A cells were incubated with M199 medium and 10% FBS was added. Cells were synchronized in serum-free medium for 24 h before the experiments. To induce the PF model in vitro, TGF-β1 (5ng/ml) was used to induce MeT5A cells for 72 h. The MeT5A cells were randomized into three groups to evaluate the effect of FSG serum on TGF-β1-induced ECM secretion: a control group (10% control serum alone), a model group (TGF-β1 + 10% control serum) and an FSG group (TGF-β1 + 10% FSG serum).

### MTT assay

MeT5A cells were cultured in M199 medium that had different doses of FSG serum or control serum (5–15%) for 72 h. Then, an appropriate volume of 3-(4,5-dimethylthiazol-2-yl)-2,5-diphenyltetrazolium (MTT) was added and the cells were incubated at 37℃. After 4 h, dimethyl sulfoxide (DMSO) was added, and the absorbance was measured at a wavelength of 450 nm.

### Immunofluorescence assay

Cells were seeded and cultured on 24-well plates. When the growth rate reached 80% above, the complete medium was replaced with 5 ng/ml of TGF-β1 alone or in combination with 10% FSG serum for 72 h. Afterward, the cells were fixed with 4% paraformaldehyde, followed by 0.5% triton X-100, and 5% BSA was used for nonspecific protein blocking. Then, the primary antibody anti-FN (1:100) or anti-α-SMA (1:100) was used overnight at 4℃. The cells were incubated with fluorescein rabbit IgG antibody (1:200) for 1 h at 37℃. The nucleus was coloured with DAPI for 20 min. Fluorescence-stimulated images were obtained using an Olympus IX73 fluorescence microscope (Tokyo, Japan).

### Western blot analysis

A RIPA buffer containing 1% protease inhibitor and phosphatase inhibitor was used for cell fragmentation, and the suspension was centrifuged at a speed of 10,000 g (10 min, 4℃). The concentration of protein in the cellular supernatant was determined using a BCA kit (Boster, China). After mixing with 2 × protein loading buffer, the protein electrophoresis was performed using 10% SDS-polyacrylamide gels (SurePAGE, GenScript). Then, the proteins were transferred to the PVDF membrane using the wet rotation method. The membranes were blocked with 5% skimmed milk powder for 1 h at 37℃ and then incubated at 4℃ overnight with anti-FN (1:2000), anti-Col Ⅰ (1:1000), anti-α-SMA (1:500), anti-PDK1 (1:2000), anti-ITG β3 (1:1000), anti-GADPH (1:10000), anti-E-cadherin (1:5000), anti-p-mTOR (1:1000), anti-mTOR (1:5000), anti-p-AKT (1:2000) and anti-AKT (1:1000). Then, HRP-conjugated affinipure goat antirabbit IgG (1:10000) or antimouse IgG (1:10000) were used for secondary antibody incubation. The enhanced chemiluminescence reagent and ChemStudio Imaging System (Germany) were used to analyse the positive bands expressed on the membranes.

### Sample preparation for label-free quantitative proteomics analysis

Samples in lysis buffer (8 M urea, 1% protease inhibitor cocktail) were sonicated three times on ice. Then, the supernatant was acquired by centrifugation (× 18000 g), and the concentration was determined using the BCA kit. The protein in the solution was digested using 10 mM DTT for 1 h and alkylated with 20 mM IAA for 45 min in a dark environment at room temperature. After diluting the urea below 2 M using Label Free label buffer, trypsin was added at 1:50 trypsin-to-protein (m/m) for the first digestion overnight and 1:100 trypsin-to-protein (m/m) for a second 4 h digestion. After trypsin digestion, the peptide was desalted by Strata X C18 SPE column (Phenomenex) and vacuum dried.

### Mass spectrometry analysis

The peptides were subjected to an NSI source followed by tandem mass spectrometry (MS/MS) in timsTOF Pro (Bruker), coupled with a NanoElute UPLC system. The gradient comprised an increase from 6% to 22% solvent B (0.1% formic acid in 90% acetonitrile) over 47 min, 22% to 32% in 20 min, climbing to 80% in 4 min and then holding at 80% for the last 4 min. The electrospray voltage applied was 1.4 kV. A data-dependent procedure alternated between one MS scan followed by 30 MS/MS scans with 30.0 s of dynamic exclusion. Automatic gain control (AGC) was set at 5E4. The fixed first mass was set at 100–1700 m/z.

The acquired MS/MS data were processed using the MaxQuant search engine (v.1.5.2.8). Tandem mass spectra were searched against a concatenated Homo sapiens database, including a reverse decoy database. Trypsin/P was specified as the cleavage enzyme, allowing for up to 2 missed cleavages. The precursor ion mass tolerance was set at 20 ppm in the first search and at 5 ppm in the main search; the fragment ion mass tolerance was set at 0.02 Da. Carbamidomethyl on Cys was designated as a fixed modification, whereas oxidation on Met and acetylation on the protein N-term were considered variable modifications. The false discovery rate (FDR) was adjusted to < 1%, with a minimum score of > 40 for the requirement of modified peptides for identification purposes. Additionally, a minimum peptide length of 7 was set. Label Free was employed for quantification. GO and KEGG enrichment analyses were performed to explore the function and biological processes associated with differentially expressed proteins (DEPs).

### Statistical analysis

Statistical analysis was performed using GraphPad Prism 5. Data are presented as mean ± standard deviation (SD). The statistical significance was determined using a two-tailed Student’s test. A P value < 0.05 was considered statistically significant.

## Results

### The potential mechanism of FSG delaying PF

**Fig. 1:**
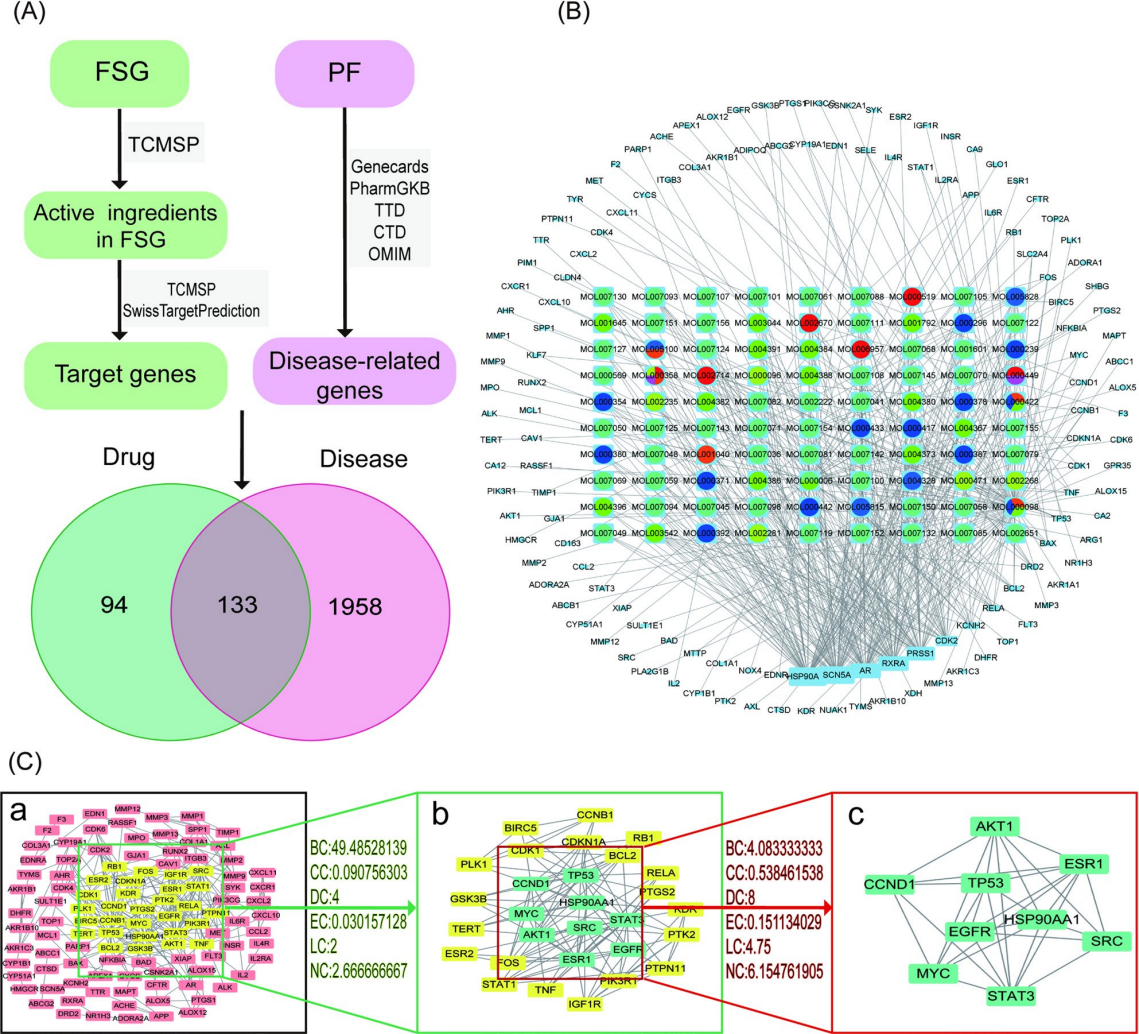
The Component-Target network construction and overlapped genes analysis. **A** The collection of targets of FSG and related genes of PF. **B** The Component-Target Network of FSG repressing PF. The light blue squares stand for targets. Larger size indicates higher centrality; the colored circles with light blue square background indicates the components from different herbs. Circles with different color indicate ingredients were contained in multi herbs. **C**–**a** The primary PPI network of FSG inhibiting PF. **C**–**b** PPI network of significant proteins extracted from C-a. **C**–**c** Core PPI network of FSG restraining PF extracted from C-b. BC, betweenness centrality; CC, closeness centrality; DC, degree centrality; EC, eigenvector centrality; LC, local average connectivity-based method; NC, network centrality

A total of 94 FSG active ingredients and 227 corresponding target genes were obtained through the TCMSP database and SwissTargetPrediction database. In addition, 2091 peritoneal fibrosis-related genes were searched from an online database (Supplementary Table S1–3). There were 113 overlapped genes and 90 mapped active components (Fig. 1A–B, Supplementary Table S4). According to the “Mol score”, MOL000098 (Quercetin), MOL000006 (Luteolin), MOL007154 (Tanshinone iia), MOL000422 (Kaempferol), MOL004328 (naringenin) and MOL002714 (Baicalein) might be the main active components of FSG in delaying PF (Supplementary Table S5). The PPI network of these overlapped genes was obtained from the STRING website. We acquired a core subnetwork containing 9 nodes and 64 edges through two-step topological screening, which may be the key targets for FSG delaying PF (Fig. 1C).

**Fig. 2:**
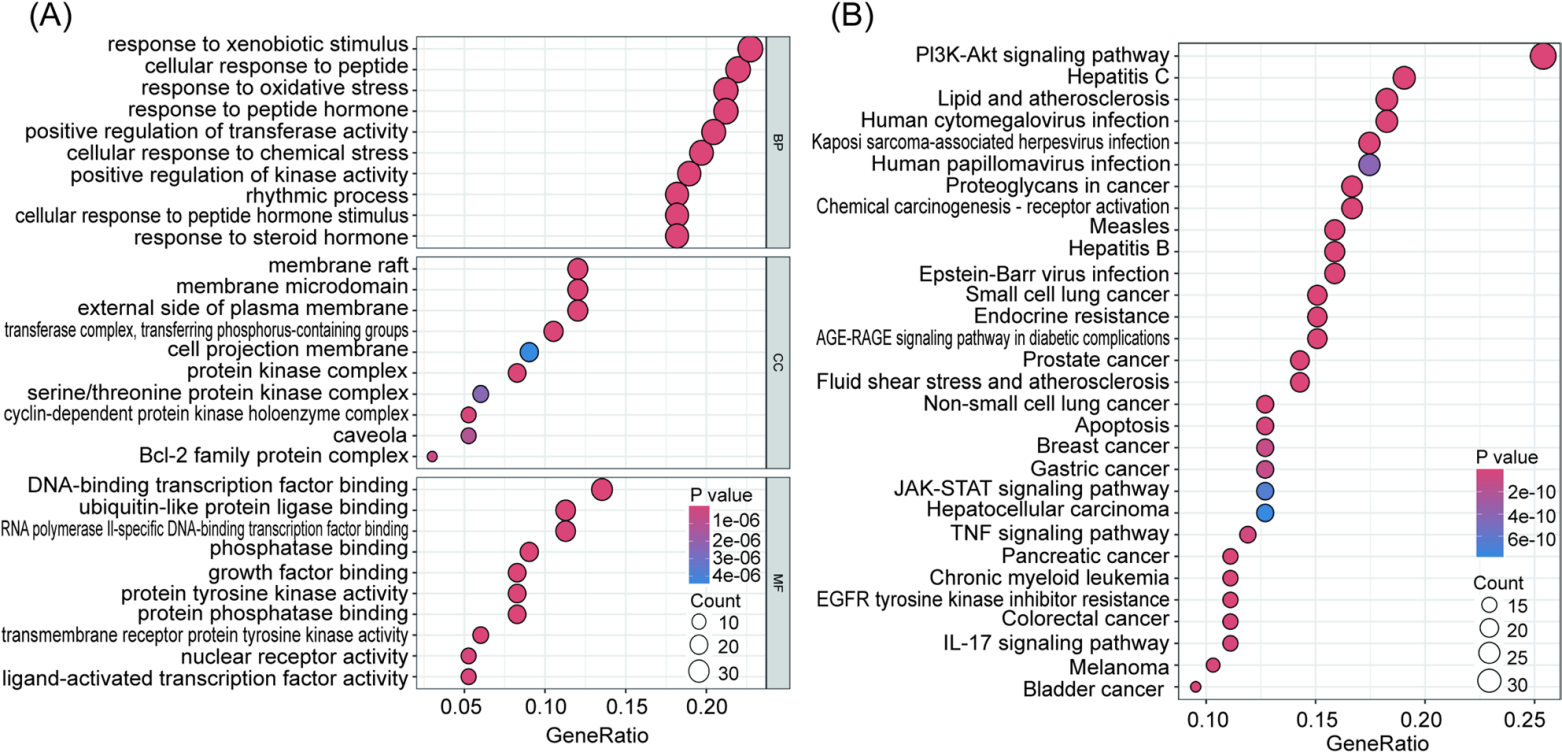
GO and KEGG pathway enrichment analysis of the overlapped genes. **A** CC, BP and MF analysis of the overlapped genes. **B** KEGG pathway enrichment analysis of the overlapped genes

A total of 2311, 69 and 172 terms associated with BP, CC and MF, respectively, were enriched in GO enrichment analysis (Supplementary Table S6). Terms such as response to xenobiotic stimulus, membrane raft and DNA-binding transcription factor binding were significantly enriched (Fig. 2A).

KEGG analysis showed that 145 signalling pathways were enriched (Supplementary Table S7). Pathways were ranked by P-values < 0.05 using Fisher’s exact test. The top 30 terms, such as PI3K/AKT signalling pathway, Hepatitis C and Lipid and atherosclerosis were significantly enriched (Fig. 2B).

### In silico validation of FSG with key targets

**Fig. 3:**
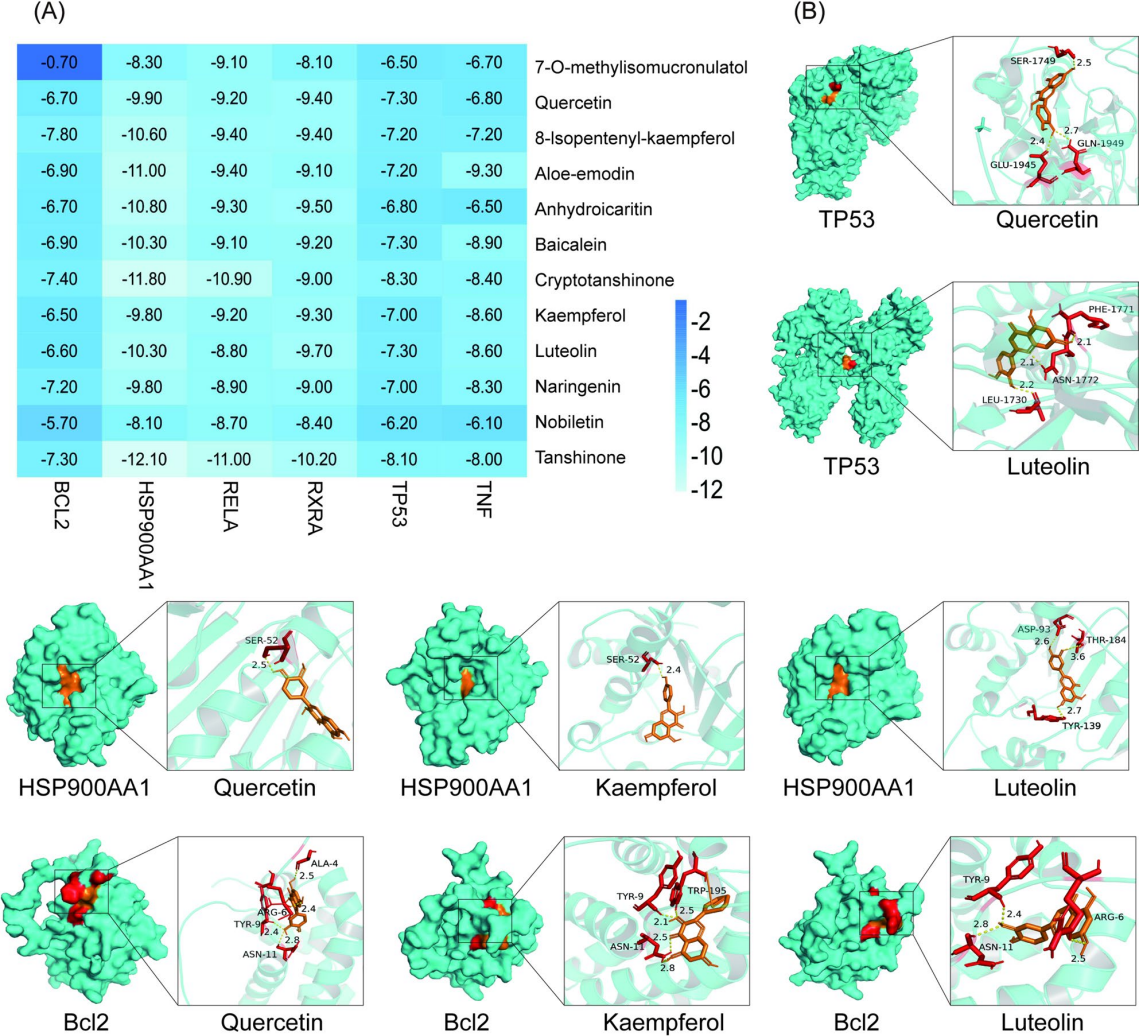
Molecular and key targets docking verifications. **A** Molecular docking score of the main ingredients of FSG with key targets in PI3K/Akt signaling pathway. **B** The interacted action mode of TP53, HSP900AA1, Bcl2 with Quercetin, Luteolin and Kaempferol

To confirm the effect of the main active components of FSG on PI3K/AKT signalling, we performed a molecular docking analysis. The results showed that except for 7-O-methylisomucronulatol binding to BCL2, the binding energy of the main ingredients to BCL2, HSP900AA1, RELA, RXRA, TP53 and TNFα were all < -5.0 kcal/mol. Moreover, the docking scores of these 11 active compounds to HSP900AA1, RELA and RXRA were <7.0 kcal/mol (Fig. 3A–B). These results suggest that the inhibition of these key proteins in the PI3K/AKT signalling pathway might be an important mechanism by which FSG delays PF.

### Cell activity detection upon FSG treatment

**Fig. 4:**
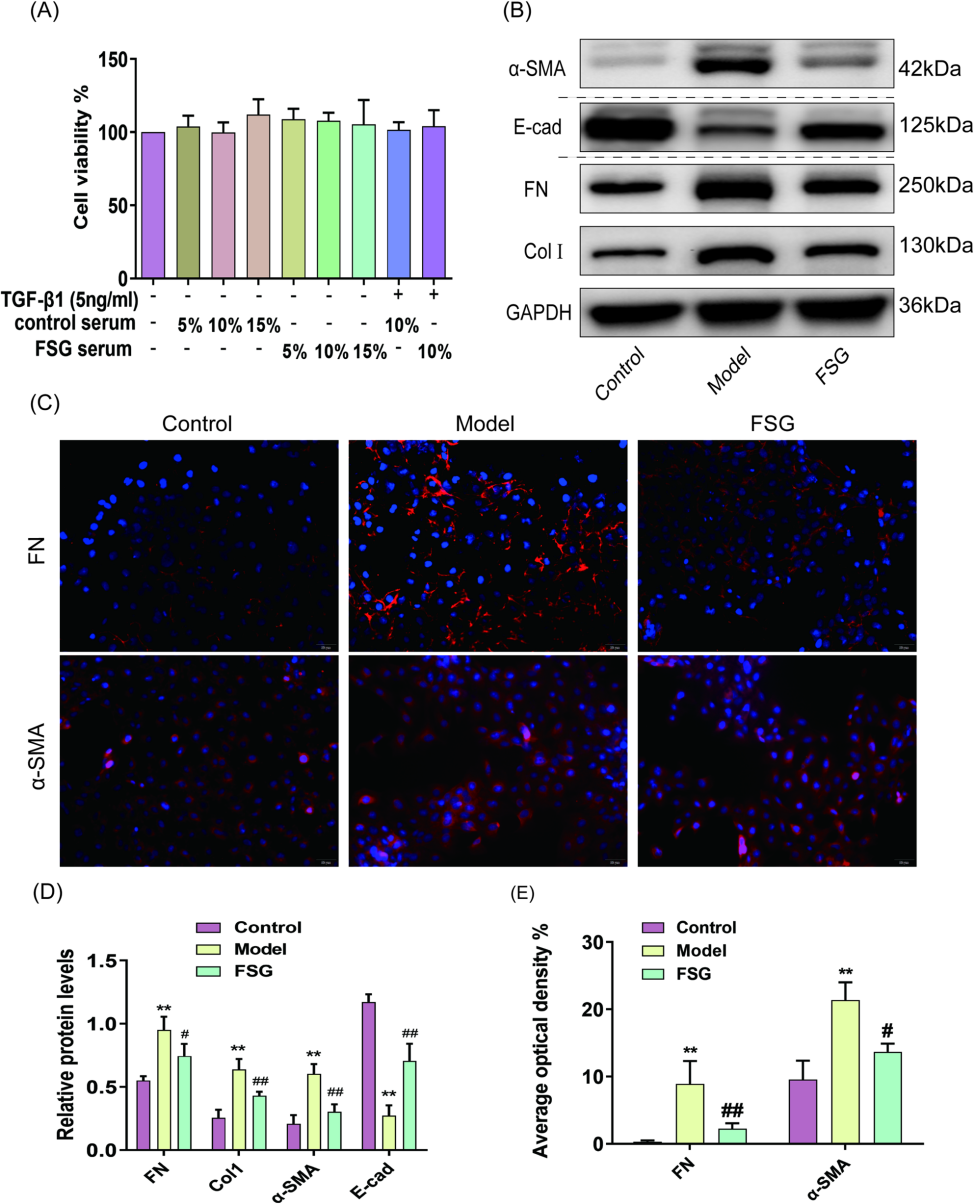
FSG attenuates the accumulation of ECM and reverses EMT in TGF-β1-challenged MeT5A cells. **A** Cell viability was measured by MTT assay. **B** Western blot assay of FN, Col Ⅰ, α-SMA and E-cad. The samples derive from the same experiment and the blots were processed in parallel. Full-length blots are presented in Supplementary materials. **C** Immunofluorescence staining of FN and α-SMA (scale bar = 50 μm). **D** Protein levels of FN, Col Ⅰ, α-SMA and E-cad as related to GAPDH in each group. **E** The average optical density of FN and α-SMA based on immunofluorescence staining. Data are expressed as mean ± SD, **indicates *P* < 0.01 compared with the control group. # indicates *P* < 0.05, ## indicates *P* < 0.01 compared with the model group, *n* = 4 per group

To confirm the effect of FSG in delaying PF, we conducted the following in vitro study using TGF-β1-treated MeT5A cells. First, MeT5A cells were treated with increasing doses of FSG serum and control serum (5–15%) for 72 h. The viability of the cells was measured using the MTT test to evaluate the potential cytotoxicity of FSG serum and control serum on TGF-β1-induced MeT5A cells. The results indicated that all doses of FSG serum and control serum had a nontoxic effect on MeT5A cells (Fig. 4A). A 10% dosage of FSG serum and control serum were selected for subsequent experiments.

### FSG attenuates the accumulation of ECM and inhibits EMT in TGF-β1-challenged MeT5A cells

To examine the inhibitory effect of FSG on ECM secretions in MeT5A cells, we performed WB and IF assays to detect the protein levels of FN and ColⅠ. The results showed that cells treated with TGF-β1 produced remarkably higher levels of Col Ⅰ and FN, while FSG decreased the levels of these proteins (Fig. 4B and D). IF staining confirmed these changes. Specifically, FN was distributed extensively in the extracellular space in the model group, and the FSG treatment attenuated its distribution (Fig. 4C and E).

It is generally acknowledged that EMT progress is essential for ECM accumulation. Our study observed an inhibitory effect of FSG on the levels of α-SMA and E-cadherin in MeT5A cells. WB showed TGF-β1 stimulation upregulated the level of α-SMA and downregulated the level of E-cad in the model group. However, FSG reversed these changes (Fig. 4B and D). Meanwhile, IF demonstrated that the number of α-SMA-positive cells decreased after FSG treatment (Fig. 4C and E).

### Identification and assessment of DEPs

In this study, proteomic data were acquired by tandem mass spectrometry (MS/MS) in timsTOF Pro (Bruker), coupled with the NanoElute UPLC system. After merging the data from the respective replicates (three biological replicates), 56,321 unique peptides (99% confidence) matching 6239 proteins (≥ 1 peptide) were identified, and 5378 proteins were finally quantified in cell lysates from the control, model and FSG groups. A criterion of |fold change|>1.5 and the relative quantification P values of 0.05 were used for DEP candidates. Following these criteria, a total of 303 DEPs were identified between the control and model groups, of which 98 proteins were upregulated and 205 proteins were downregulated in the model group compared to the control group (Supplementary Table S8). Similarly, 386 DEPs were identified in the FSG group versus the model group. Among these proteins, 253 were upregulated and 133 were downregulated (Supplementary Table S9).

### DEPs for comprehensive analysis

**Fig. 5:**
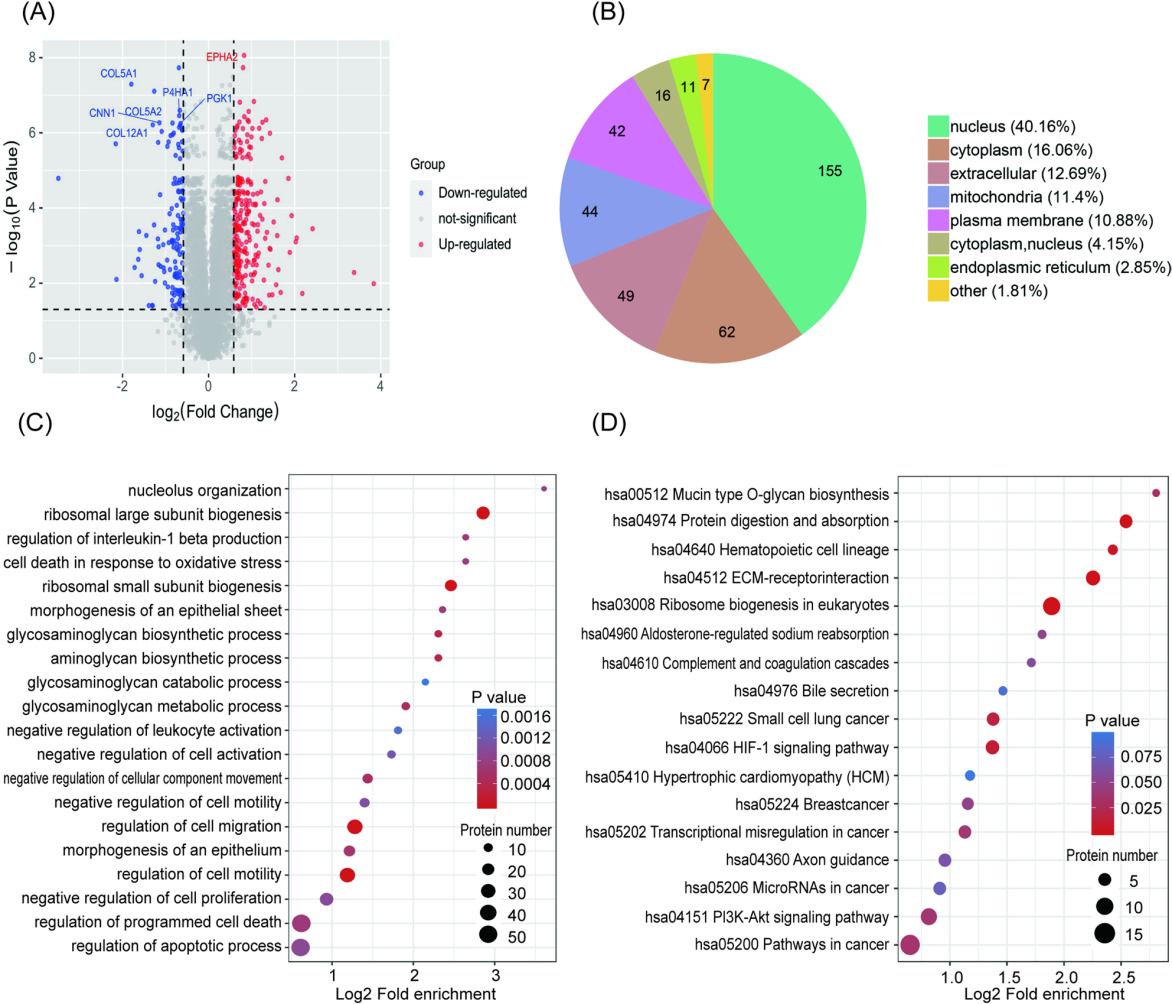
Bioinformatics analysis of the label-free proteomics results. **A** Volcano plot of protein expression profile data of differentially expressed proteins (DEPs) from model group vs. FSG group. The ordinate represents the statistical significance of the variations in protein expression, and the abscissa represents the fold changes (FC) in protein expression. **B** Subcellular localization of DEPs. **C** Biological process analysis of DEPs identified in model group vs. FSG group. **D** KEGG pathway enrichment analysis of DEPs in model group vs. FSG group. The proteomic data was obtained from three biological replicates per group

DEPs from the model group versus the FSG group were used for further bioinformatics analysis to reveal the mechanism of FSG against PF. The volcano plot of protein expression profile data from the model and FSG groups and the subcellular localization of these 386 DEPs are shown in Fig. 5A and B.

We used GO BP enrichment to analyse the DEPs in the FSG group versus the model group to visualise the pharmacological network of FSG delays in PF. The significance of each term was indicated by Benjamini-Hochberg’s corrected Fisher’s exact test to calculate the P values. Highlights from the tables are presented in Fig. 5C. Many processes related to ECM formation are enriched, such as the aminoglycan biosynthetic process and the glycosaminoglycan biosynthetic/metabolic process. The results also indicated that FSG can regulate the EMT progress of MeT5A cells, which can be confirmed by the regulation of cell migration, regulation of cell motility, negative regulation of cellular component movement and epithelium morphogenesis.

The result of the KEGG pathway annotation showed that 17 pathways were significantly affected by FSG (Fig. 5D). The main enriched pathways included ribosome biogenesis in eukaryotes, ECM–receptor interaction, protein digestion and absorption, the HIF-1 signalling pathway, and the PI3K-AKT signalling pathway. Because the PI3K/AKT signalling pathway was enriched both in the network pharmacology and the proteomics analysis, it may be the most important mechanism of FSG’s pharmacological effects; therefore, we selected it for further verification.

### FSG suppressed the PI3K/AKT pathway in TGF-β1-induced MeT5A cells

**Fig. 6:**
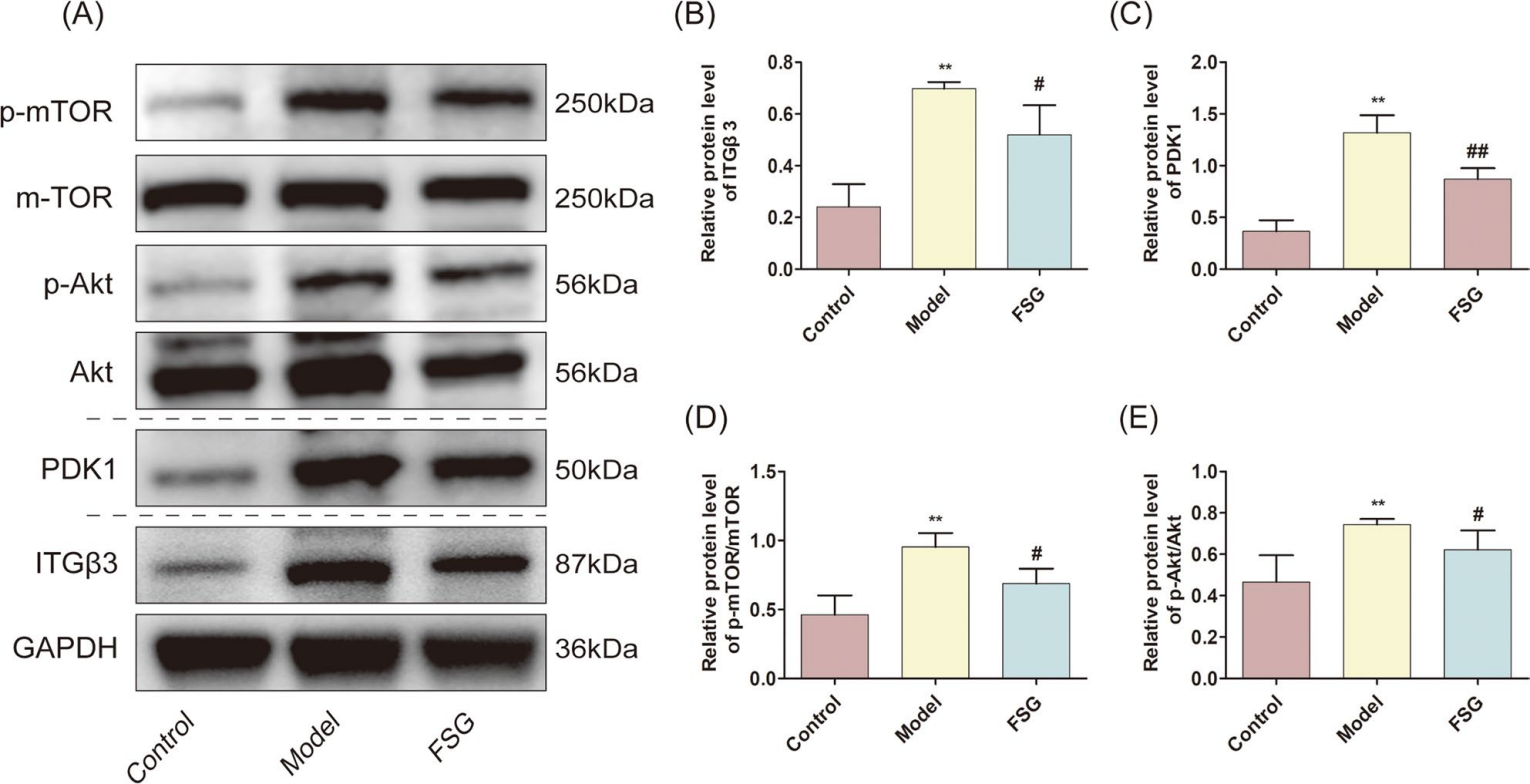
FSG suppressed PI3K/Akt pathway in TGF-β1-induced MeT5A cells. **A** Western blot assay of ITGβ3, PDK1, p-mTOR, mTOR, p-Akt, Akt. The samples derive from the same experiment and the blots were processed in parallel. Full-length blots are presented in Supplementary materials. **B-E** Protein levels of ITGβ3, PDK1, p-mTOR/mTOR, p-Akt/Akt as related to GAPDH in each group. Data are expressed as mean ± SD, **indicates *P* < 0.01 compared with the control group. # indicates *P* < 0.05, ## indicates *P* < 0.01 compared with the model group, *n* = 4 per group

We further detected the influence of FSG on the PI3K/AKT signalling pathway in TGF-β1-treated MeT5A cells. The WB results showed that TGF-β1 significantly upregulated the protein expression of ITGβ3, PDK1, phosphorylated-AKT and phosphorylated-mTOR, and FSG abolished these changes (Fig. 6A–E). This result indicates that the delaying effect of FSG on PF might be associated with the inhibition of PI3K/AKT signalling.

## Discussion

Fibrosis is mainly characterised by an abnormal accumulation of ECM (such as FN and collagens) and a disorder of ECM degradation in many tissues. Studies have indicated that TGF-β1 accelerates EMT as the initiating factor involved in the occurrence of fibrosis [36–39]. Although drugs such as TGF-β blockers, statins and tamoxifen have been reported to effectively regulate EMT [40–43], these agents have not achieved the anticipated effects in PD patients [44]. Therefore, a better treatment strategy for delaying PF is urgently needed.

Traditional Chinese medicine has various advantages and has achieved effects in treating fibrotic disease. We previously found *Saikosaponin D*, an ingredient extracted from Chinese medicine *Radix Bupleuri*, could ameliorate PF through regulating TGFβ1/Gremlin1/Smad signalling [45]. Other studies have reported that Chinese medicine formulas (such as the *Niao Du Kang* mixture) can intervene in fibrosis in PD-related PF [46, 47]. Our clinical study showed that FSG can increase plasma HGB and ALB levels and improve dialysis efficiency in PD patients [30]. FSG can also alleviate inflammation in PD rats by regulating P38 MAPK signalling [26]. Traditional Chinese medicine has the advantages of multi-active ingredients, multi-targets and multi-level regulatory functions in treating diseases. Network pharmacology provides researchers with a quick and comprehensive understanding of the interaction between the active ingredient of Chinese herb prescription and disease-related targets [48]. Therefore, we applied this method to explore the possibility of an interaction between FSG and PF.

In this study, 133 overlapped genes and 90 mapped active components were obtained. The top 5 components with the highest degree might be the main effective components of FSG in treating PF. Quercetin (MOL000098) and Luteolin (MOL000006) have been verified to alleviate PF via regulating inflammation, EMT and oxidative stress [49–51]. In addition, Tanshinone IIA (MOL007154), Kaempferol (MOL007154) and Baicalein (MOL002714) may be the key and active compounds of FSG in treating PF, as they reduce TGF-β1, CTGF and MMP2 expression, respectively [52–54].

We acquired 9 hub genes through two-step topological screening, which may be the key targets for FSG delaying PF. Target genes such as STAT3, AKT1 and TP53 have been proven to participate in the occurrence of PF and were considered potential targets of the effect component in FSG [50, 55, 56]. EGFR can contributes to peritoneal fibrosis via regulating TGF-β1/Smads signalling [57]. As downstream proteins of the β-catenin signalling pathway, CCND1 and MYC accelerate EMT and fibroblast transformation in fibrotic disease [58].

KEGG pathway analysis of these 113 overlapped genes showed PI3K/AKT signalling was significantly enriched. Numerous studies have indicated that the PI3K/AKT signalling pathway plays an essential role in the development of fibrotic diseases [59–61]. Inhibition of the PI3K/Akt signaling pathway can delay the progression of PF by regulating macrophage polarization, autophagy and EMT [55, 62, 63]. Therefore, it is essential to assess the activation status of PI3K/AKT signalling in PF and to illuminate its role in the FSG-mediated suppression of PF.

Molecular docking can effectively calculate the binding energy between small chemical ligands and protein receptors [64]. Therefore, we carried out molecular docking of the major compounds in FSG and the key proteins to test whether FSG regulates PI3K/AKT signalling. The results showed that, except for 7-O-methylisomucronulatol binding to BCL2, the affinities of the 12 FSG-effective components with key targets participating in PI3K/AKT signal transduction were all<-5 kcal/mol. In addition, 8-isopentenyl-kaempferol, cryptotanshinone, naringenin and thanshinone exhibited stronger affinity to these proteins (-7 kcal/mol). The molecular docking analysis indicated that the main effective ingredient of FSG could regulate PI3K/AKT signalling through direct interaction.

To verify the above hypothesis, we chose a TGF-β1 induced MeT5A cell model to conduct the experiment. As expected, we reconfirmed that TGF-β1 induced the secretion of FN and Col Ⅰ and upregulation of the mesenchymal phenotype α-SMA as well as downregulation of the epithelial phenotype E-cadherin in Met5A cells. However, these changes were reversed with FSG treatment, indicating the antifibrotic properties of FSG.

We performed label-free proteomics analysis to investigate the underlying mechanism of FSG interfering with PF. We found 386 DEPs in the model group versus the FSG group. Among these, 253 proteins were upregulated and 133 were downregulated after FSG treatment. Some of these DEPs with significant *P* values were documented as having pivotal involvement in the pathogenesis of fibrotic disease. Research has demonstrated that upregulating the expression of ephrin type-A receptor 2 (EPHA2) in human umbilical cord mesenchymal stem cells can enhance their anti-fibrotic properties [65]. Inhibition of Phosphoglycerate kinase 1 (PGK1), prolyl 4-hydroxylase subunit alpha-1 (P4HA1), and calponin-1 (CNN1) expression can alleviate tissue fibrosis, indicating the important roles of these targets in the progression of tissue fibrosis [66–68]. Consistent with these studies, we found that the expression of the PGK1, P4HA1 and CNN1 proteins in Met5A cells was downregulated, while EPHA2 was upregulated with FSG treatment. This result indicates that the intervening effect of FSG on PF may be related to its regulation of these DEPs.

The BP analysis of GO showed that morphogenesis of the epithelium and regulation of cell motility were significantly enriched. This confirmed that FSG could regulate the EMT process by improving these proteins in TGF-β1-induced Met5A cells. We also conducted a KEGG enrichment analysis of these 386 DEPs. The results showed that 9 DEPs were enriched in the ‘ECM-receptor interaction’. Among the ECM-binding receptors, integrins play a dominant role in the process of protein–protein interaction. It has been reported that integrin α5 can participate in PF through interaction with fibronectin. The activation of integrin β3 accelerates the accumulation of ECM in a pressure overload hypertrophy mouse [69]. We also found that the HIF-1α signalling pathway was significantly enriched. Dysregulated expression of HIF-1α has been implicated in the pathogenesis of fibrosis in myocardial and pulmonary tissues [70, 71]. Under hypoxic conditions, integrin β3 can regulate cardiomyocyte apoptosis via HIF-1α [72]. Another enriched signaling pathway, PI3K/AKT, plays a vital role in tissue fibrosis by virtue of its regulatory effects on processes such as autophagy and apoptosis [73–76]. Therefore, the regulation of these signalling pathways may represent a crucial mechanism for FSG to modulate PF.

Combined with our network pharmacology results, we chose several key proteins involved in the PI3K/AKT signalling pathway for further verification. It was proven that knockdown of ITGβ3 can reverse LPS-induced pulmonary fibrosis through inactivated PI3K/AKT/mTOR signalling [77]. The activity of 3-phosphoinositide-dependent kinase 1 (PDK1) is closely related to inflammatory responses and the development of fibrosis. Restraining the activity of PDK1 can induce decreased levels of TNF-α, IL-6, MCP-1 and TGF-β [78]. Inhibition of AKT/mTOR signalling can suppress PF in the process of PD by activating autophagy and reducing the ROS level [55, 79]. In this study, TGF-β1 promoted the protein expression of ITGβ3, PDK1, p-AKT and p-mTOR in MeT5A cells. However, FSG abolished these changes, indicating that FSG could repress PI3K/AKT signalling in the PF process. On the basis of these findings, our results demonstrate that the reason for PF induced by TGF-β1 may be the activation of the PI3K/AKT signalling pathway. FSG could restrain PF through a repressed PI3K/AKT signalling pathway and reverse EMT development.

## Conclusion

In this study, we used network pharmacology to predict the effective ingredient, potential targets and signalling pathways of FSG in treating PF. Molecular docking analysis showed that the regulation of PI3K/AKT signalling could be an important mechanism of FSG delaying PF. We then conducted an in vitro experiment, and the results showed that FSG indeed reduces ECM secretion and reverses EMT progression in TGF-β1-induced MeT5A cells. Proteomics analysis identified many DEPs that were related to the TGF-β1 stimulation and/or FSG treatment. We demonstrated the antifibrotic effect of FSG on PF, possibly by regulating PI3K/AKT signalling. Further studies are needed to investigate the precise mechanism of these targets and the pathways in which they participate, as this information is essential for developing potential therapeutic targets for PF and for understanding the potential role of FSG in treating PF.

## Supplementary Information

Supplementary Material 1.

Supplementary Material 2.

Supplementary Material 3.

Supplementary Material 4.

Supplementary Material 5.

Supplementary Material 6.

Supplementary Material 7.

Supplementary Material 8.

Supplementary Material 9.

Supplementary Material 10.

## Data Availability

The datasets used in this study are available from the corresponding author on reasonable request. The mass spectrometry proteomics data were deposited with the ProteomeXchange Consortium via the PRIDE partner repository with the dataset identifier PXD033058.
