# Supplementary material for: An integrated network pharmacology and proteomics approach reveals the anti-fibrotic effect of Fushen Granule on peritoneal fibrosis
Source: BMC Complement Med Ther. 2026 Mar 9;26:143. doi: 10.1186/s12906-026-05333-2 (PMC13085474; doi:10.1186/s12906-026-05333-2)
Supplement: Supplementary file 1 — Supplementary Material 1. [file 12906_2026_5333_MOESM1_ESM.pdf]

Article title: An Integrated Network Pharmacology and Proteomics Approach Reveals the Anti-fibrotic Effect of Fushen Granule on Peritoneal Fibrosis
Author names: Kang Yang, Jie Li, Lin Wang, Hangxing Yu, Xinyue Liu, Zhiqing Gao, Zheng Wang, Linqi Zhang, Hongtao Yang
Affiliation and e-mail address of the corresponding author: First Teaching Hospital of Tianjin University of Traditional Chinese Medicine, tjtcmht@126.com

94 active ingredients of FSG were retrieved from TCMSP database	
MolId	MolName
MOL002670	Cavidine
MOL002714	baicalein
MOL000358	beta-sitosterol
MOL000449	Stigmasterol
MOL000519	coniferin
MOL006957	(3S,6S)-3-(benzyl)-6-(4-hydroxybenzyl)piperazine-2,5-quinone
MOL004328	naringenin
MOL005100	5,7-dihydroxy-2-(3-hydroxy-4-methoxyphenyl)chroman-4-one
MOL005815	Citromitin
MOL005828	nobiletin
MOL002235	EUPATIN
MOL002268	rhein
MOL002281	Toralactone
MOL000471	aloe-emodin
MOL000096	(-)-catechin
MOL001601	1,2,5,6-tetrahydrotanshinone
MOL002222	sugiol
MOL002651	Dehydrotanshinone II A
MOL000569	digallate
MOL000006	luteolin
MOL007036	5,6-dihydroxy-7-isopropyl-1,1-dimethyl-2,3-dihydrophenanthren-4-one
MOL007041	2-isopropyl-8-methylphenanthrene-3,4-dione
MOL007045	3α-hydroxytanshinone II a
MOL007048	(E)-3-[2-(3,4-dihydroxyphenyl)-7-hydroxy-benzofuran-4-yl]acrylic acid
MOL007049	4-methylenemiltirone
MOL007050	2-(4-hydroxy-3-methoxyphenyl)-5-(3-hydroxypropyl)-7-methoxy-3-benzofurancarboxaldehyde
MOL007058	formyltanshinone
MOL007059	3-beta-Hydroxymethyllenetanshiquinone
MOL007061	Methylenetanshinquinone
MOL007068	Przewaquinone B
MOL007069	przewaquinone c
MOL007070	(6S,7R)-6,7-dihydroxy-1,6-dimethyl-8,9-dihydro-7H-naphtho[8,7-g]benzofuran-10,11-dione

MOL007071	przewaquinone f
MOL007079	tanshinaldehyde
MOL007081	Danshenol B
MOL007082	Danshenol A
MOL007085	Salvilenone
MOL007088	cryptotanshinone
MOL007093	dan-shexinkum d
MOL007094	danshenspiroketallactone
MOL007098	deoxyneocryptotanshinone
MOL007100	dihydrotanshinlactone
MOL007101	dihydrotanshinone Ⅰ
MOL007105	epidanshenspiroketallactone
MOL007107	C09092
MOL007108	isocryptotanshi-none
MOL007111	Isotanshinone II
MOL007119	miltionone Ⅰ
MOL007120	miltionone Ⅱ
MOL007121	miltipolone
MOL007122	Miltirone
MOL007124	neocryptotanshinone ii
MOL007125	neocryptotanshinone
MOL007127	1-methyl-8,9-dihydro-7H-naphtho[5,6-g]benzofuran-6,10,11-trione
MOL007130	prolithospermic acid
MOL007132	(2R)-3-(3,4-dihydroxyphenyl)-2-[(Z)-3-(3,4-dihydroxyphenyl)acryloyl]oxy-propionic acid
MOL007142	salvianolic acid j
MOL007143	salvilenone Ⅰ
MOL007145	salviolone
MOL007150	(6S)-6-hydroxy-1-methyl-6-methylol-8,9-dihydro-7H-naphtho[8,7-g]benzofuran-10,11-quinone
MOL007151	Tanshindiol B
MOL007152	Przewaquinone E
MOL007154	tanshinone iia
MOL007155	(6S)-6-(hydroxymethyl)-1,6-dimethyl-8,9-dihydro-7H-naphtho[8,7-g]benzofuran-10,11-dione
MOL007156	tanshinone Ⅵ
MOL001040	(2R)-5,7-dihydroxy-2-(4-hydroxyphenyl)chroman-4-one
MOL001420	ZINC04073977
MOL000422	kaempferol
MOL000098	quercetin
MOL000239	Jaranol

MOL000296	hederagenin
MOL000354	isorhamnetin
MOL000371	3,9-di-O-methylnissolin
MOL000378	7-O-methylisomucronulatol
MOL000380	(6aR,11aR)-9,10-dimethoxy-6a,11a-dihydro-6H-benzofurano[3,2-c]chromen-3-ol
MOL000387	Bifendate
MOL000392	formononetin
MOL000417	Calycosin
MOL000433	FA
MOL000442	1,7-Dihydroxy-3,9-dimethoxy pterocarpene
MOL001645	Linoleyl acetate
MOL001792	DFV
MOL003044	Chryseriol
MOL003542	8-Isopentenyl-kaempferol
MOL004367	olivil
MOL004373	Anhydroicaritin
MOL004380	C-Homoerythrinan, 1,6-didehydro-3,15,16-trimethoxy-, (3.beta.)-
MOL004382	Yinyanghuo A
MOL004384	Yinyanghuo C
MOL004386	Yinyanghuo E
MOL004388	6-hydroxy-11,12-dimethoxy-2,2-dimethyl-1,8-dioxo-2,3,4,8-tetrahydro-1H-isochromeno[3,4-h]isoquinolin-2-ium
MOL004391	8-(3-methylbut-2-enyl)-2-phenyl-chromone
MOL004396	1,2-bis(4-hydroxy-3-methoxyphenyl)propan-1,3-diol
MOL000622	Magnograndiolide
