# Supplementary material for: An integrated network pharmacology and proteomics approach reveals the anti-fibrotic effect of Fushen Granule on peritoneal fibrosis
Source: BMC Complement Med Ther. 2026 Mar 9;26:143. doi: 10.1186/s12906-026-05333-2 (PMC13085474; doi:10.1186/s12906-026-05333-2)
Supplement: Supplementary file 2 — Supplementary Material 2. [file 12906_2026_5333_MOESM2_ESM.pdf]

Article title: An Integrated Network Pharmacology and Proteomics Approach Reveals the Anti-fibrotic Effect of Fushen Granule on Peritoneal Fibrosis
Author names: Kang Yang, Jie Li, Lin Wang, Hangxing Yu, Xinyue Liu, Zhiqing Gao, Zheng Wang, Linqi Zhang, Hongtao Yang
Affiliation and e-mail address of the corresponding author: First Teaching Hospital of Tianjin University of Traditional Chinese Medicine, tjtcmht@126.com

227 targets related to active ingredients of FSG were obtained from TCMSP and SwissTargetPrediction database:

Target
CHRM3
KCNH2
CHRM1
ADRB1
SCN5A
CHRM5
ADRA2C
CHRM4
RXRA
ADRA1B
ADRB2
ADRA1D
HSP90AA1
RXRB
CAMTA2
DRD1
AR
PRSS1
RELA
BCL2
FOS
BAX
TP53
CDK1
CCNB1
CYCS
TDRD7
FABP5
ADRA1A
CHRM2
CHRNA2
GABRA1
IGHG1

ADRA2A
SLC6A2
CDK2
BAD
MTTP
CYP19A1
ADIPOQ
CES1
TIMP1
CD163
CDKN1A
TNF
MYC
KLF7
COLQ
DRD2
CCND1
IL10RB
RB1
CDK4
IL6R
NFKBIA
APP
MCL1
BIRC5
IL2RA
IL4R
XIAP
SLC2A4
INSRR
STAT3
EDN1
DRD5
ADRA2B
GABRG3
GABRE
EDNRA
NR1I2

ITGB3
STAT1
SELE
PSMD3
NR1I3
PPP3CA
ELK1
RUNX1T1
CAV1
GJA1
CCL2
SULT1E1
COL1A1
ABCG2
COL3A1
CXCL11
CXCL2
DCAF5
CLDN4
CXCL10
SPP1
RUNX2
RASSF1
PCOLCE
ADH1B
LYZL6
HSD3B2
HSD3B1
NOX4
AKR1B1
CDK5R1
XDH
MAOA
FLT3
CA2
CCNB3
ALOX5
ADORA1

CA7
GLO1
SYK
GSK3B
PARP1
TTR
MMP9
CA12
MMP2
CA4
MMP12
CD38
CYP1B1
AKR1B10
TNKS2
TNKS
TOP1
ARG1
AVPR2
IGF1R
EGFR
F2
PIM1
AURKB
DRD4
MPO
PIK3R1
ADORA2A
DAPK1
PYGL
CA1
SRC
PTK2
HSD17B2
KDR
MMP13
MMP3
CA3

ALOX15
ABCC1
PLK1
CA6
PKN1
CA14
CA9
CSNK2A1
ALOX12
MET
NEK2
CXCR1
CAMK2B
ALK
AKT1
ABCB1
NEK6
PLA2G1B
CA5A
BACE1
AXL
NUAK1
AKR1C2
AKR1C1
AKR1C3
AKR1C4
CA13
AKR1A1
GPR35
IL2
KDM4E
GRK6
PDE5A
ACHE
CES2
PTPN6
PTPN11
TERT

EED
POLA1
POLB
HSD17B1
SHBG
CBR1
NPC1L1
NR1H3
PTPRS
TYMS
CTSD
HMGCR
CYP51A1
FTO
MAPT
TOP2A
INSR
MYLK
PIK3CG
APEX1
RORC
CDK6
ESR1
ESR2
MPG
SLC22A12
CYP17A1
CAPN1
HTR7
PTGS1
TYR
AHR
ESRRA
PTGS2
CFTR
MAOB
AMY1A
F3

DHFR
SREBF2
HTR1A
MMP1
