# Supplementary material for: An integrated network pharmacology and proteomics approach reveals the anti-fibrotic effect of Fushen Granule on peritoneal fibrosis
Source: BMC Complement Med Ther. 2026 Mar 9;26:143. doi: 10.1186/s12906-026-05333-2 (PMC13085474; doi:10.1186/s12906-026-05333-2)
Supplement: Supplementary file 3 — Supplementary Material 3. [file 12906_2026_5333_MOESM3_ESM.pdf]

Article title: An Integrated Network Pharmacology and Proteomics Approach Reveals the Anti-fibrotic Effect of Fushen Granule on Peritoneal Fibrosis
Author names: Kang Yang, Jie Li, Lin Wang, Hangxing Yu, Xinyue Liu, Zhiqing Gao, Zheng Wang, Linqi Zhang, Hongtao Yang
Affiliation and e-mail address of the corresponding author: First Teaching Hospital of Tianjin University of Traditional Chinese Medicine, tjtcmht@126.com

2091 peritoneal fibrosis-related genes were searched from 5 database

Targets
VCAM1
PDE7B
MAPK8
ADGRE1
VEGFA
FOS
AGER
TGFB1
CCL2
INSR
CFLAR
NOS2
CTSB
AKR1C3
HMOX1
COL3A1
HRAS
ABCC4
PTGS1
ABCB11
IL1B
LEP
CASP7
IL6
FN1
PPARGC1A
INS1
CYCS
GPX1
ZNF681
GSK3B
ACTA2
PHAF1

HIF1A
COL1A1
CASP3
TNF
STAT3
GSR
AP5S1
FAM76A
SCYL3
ZCCHC3
DNAJC14
BCL2L1
POT1
ATG4A
RNF216
ZMYM4
DHX35
DMAP1
DNAJC28
ERLEC1
STX4
DDIT3
CPA3
MYSM1
AASDH
HAPSTR1
DNAAF2
NDST2
DCBLD1
WASHC4
SMCO4
INS2
BRWD3
IFT81
RNF217
ZYG11B
DUS2
USP6NL

MKRN1
SSH2
SOD1
ARHGAP12
CAPRIN2
RFLNA
TMEM177
MMP9
NFKBIA
ABCB10
NUP107
ZNF367
ATAD2B
CASP8
H2BC9
SAFB2
CDC73
CNOT2
CRTC2
GAB2
HIF1AN
ZC2HC1A
BRD1
PPP6R3
FBXL7
NBEAL1
POLR3B
RNF26
SFMBT1
XPO6
FER
PIGP
SOD2
OSTM1
CIZ1
HECA
RMND5A
RNF6

CNOT6L
IFT70B
NFX1
CYP1A2
GPR160
GTF3C2
HERC1
IBTK
ZKSCAN1
BCL7A
CBLL1
CLASP1
NFS1
SENP7
ATN1
RAB11B
CDK19
H4C9
LRRC28
NCK1
PPARG
ZHX3
ADAMTS12
BPNT2
GIGYF2
RBFOX2
CREBZF
MON2
PBRM1
RNASE1
CAND1
CCNT2
HAND2
KIF13A
RALGAPA2
DUSP7
PARP8
SCLY

SSBP3
AOPEP
ASAP2
LEAP2
NAPEPLD
UBFD1
USP24
DIXDC1
EPN2
FGD6
ITGB3BP
SASH1
SCAPER
SRRM1
YTHDC1
KAT6A
SH2D4A
STX11
TANC1
TRAM2
VRK1
CMIP
EXOC4
UPF3B
AIFM2
SRPK2
WDFY3
DYRK1A
GRAMD1B
TINF2
TPST1
NME7
TMCC1
BAZ2B
CIART
PHACTR2
SGO2
ATXN2

KMT2C
LACTB2
SIPA1L1
CHST11
DROSHA
TLE4
APPL2
MNS1
PTER
TAOK3
MCU
PHLDB1
XRCC3
CHD9
FMNL2
MED13L
NFE2L2
CXCL8
ELF1
GJA4
GTF2IRD1
OLFML2B
PIAS1
RPL14
TLE1
AJUBA
EFNA5
NUAK1
RBMS1
TNRC6B
APBB2
DAAM1
LATS2
NBR1
PHIP
GPHN
NEIL3
PHF19

RNASEH2A
DLL4
FRMD6
KLF7
RASGRP3
PARP1
ARHGAP29
MAGI1
MOV10
ANXA11
KANK1
RAB27B
SLC6A13
CBX4
ETV6
SERPINB1
GAB1
KIF14
ARHGAP24
ASAP1
CHD7
LGR4
ND1
PIGA
FXYD5
KLHL21
MEIS2
SERPINB5
SLCO3A1
TOB1
CSNK2A2
EHBP1
KIF4A
GFPT2
GNE
IP6K2
POLA2
CFTR

TERT
LOC110806263
SFTPC
TERC
SFTPA1
TMEM67
CCN2
BRCA1
ALB
HFE
SERPINA1
IL10
MIF
TP53
EDNRA
MUC1
IFNG
ELANE
FCGR2A
CRP
CTNNB1
CEACAM3
SERPINE1
GCLC
HLA-DRB1
CEACAM6
PKHD1
BRCA2
TIMP1
CAV1
MPO
TLR4
CDH1
IL17A
IL13
WT1
IL1RN
HGF

EGFR
BMP6
MMP2
CDKN2A
SMAD4
CALR
ICAM1
ABCB4
ACE
CD36
SMAD3
SPP1
IL18
IL4
MUC16
IL1A
CCR6
JAK2
THBD
MIR21
CXCL10
MEFV
CSF3
NPHP1
CP
AKT1
HMGB1
TLR2
EDN1
MKS1
SMAD2
ITGAM
IL2
IL5
CXCL2
SMAD7
PRTN3
S100A9

FGF2
PKD1
CSF2
PDGFRA
CCL5
MMP1
IGF1
INS
MUC5AC
NLRP3
EGF
DCDC2
PTEN
MBL2
CXCL1
SPINK1
ENG
S100A8
B2M
KRT18
PTGS2
PGR
MET
REN
ELN
MMP7
ERBB2
POLE
CR1
IRF5
EPO
IL1R1
JUN
SFTPD
LCN2
CCL3
KRAS
VIM

ATM
TNFRSF1A
ALOX5
AGT
F2
FASLG
TGFB2
LBP
MMP8
PRSS1
CCL11
CALB2
CHI3L1
PKD2
KRT7
HP
NR1H4
NF2
LOX
TF
SFTPB
MIR34C
CXCR4
WDR35
LGALS3
MPL
NOS3
CEACAM5
SERPINH1
IL33
INVS
TET2
MAPK3
PDGFB
FAS
ARG1
BAP1
SLPI

PDGFRB
TGFBR1
ESR1
GUCY2C
SLC40A1
NEK8
CXCL12
AGTR1
SELP
HAMP
CD34
PIK3CA
CTSG
IGFBP3
BRIP1
CCL4
AQP1
INSL6
MIRLET7D
MIR214
TGFB3
GPT
BMP7
CCL18
NKX2-1
TGM2
RAD51C
MIR155
CXCR2
GREM1
MMP3
CXCR3
IGFBP5
CCL22
HSPB1
GSTM1
ATP8B1
IL9

MIR200B
SNAI1
CCL17
ABCB1
PDGFA
ADA
ITGB2
RNASE3
CD14
POSTN
HJV
FGF7
CD4
PLG
RBP4
KRT20
BGLAP
STAT1
PTX3
ADIPOQ
ABCC1
C3
BRAF
GC
DEFB1
CXCR1
SLC17A5
MIR223
CD40
TOLLIP
GSN
VIP
ACP5
ANGPT2
HNF1B
ANXA5
CLCA1
MIR199A1

DZIP1L
LACTB
DAB2
CD80
MYO5B
LPAR1
AP3B1
LTF
GGT1
ACTC1
COL4A3
SPARC
UMOD
EPCAM
ANO1
MIR30A
ALMS1
KRT19
CMA1
HSP90AA1
MED12
SH2B3
GSTP1
CSF1
AMBP
BIRC5
SP110
APOE
NPHS1
CDKN3
TTR
DES
MIP
SRC
CYP19A1
CD69
MSLN
IL2RA

CD274
COL1A2
CXCL5
PMS2
AQP3
MUC4
LIPA
BPI
DNAH8
GUSB
TLR9
ATF6
TTC26
HSPA4
CCK
CCR2
COL4A5
PTPRO
HSPD1
HSPA5
CREB1
S100A4
EZR
DEFB4A
SGK1
MDM2
PRKCA
MRC1
PTPRC
CAMP
BMPR2
HSPG2
MASP2
MIR146A
HSPH1
GRP
DCN
PAX2

AFP
KIT
MST1
SCN5A
ERN1
NOX4
MSH6
MAPK1
F2R
PLAU
APOA1
MTOR
NOTCH1
CDH2
SCGB1A1
PDPN
KNG1
IGF2
DDR1
ANXA1
XBP1
FCGR3B
FCGR1A
APOB
CD44
MIR130A
ANXA2
APC
MIR125A
RYR1
TGFA
MMP12
SLC2A1
APEX1
F3
MIR34A
ADORA2B
PTGER4

MLH1
IL3
MIR34B
CD79A
FSHR
S100A1
LPL
MIR200C
SMPD1
NFKB1
VDR
NOS1
IL12RB1
AGTR2
ITGB1
NOD2
MIR200A
MIR195
LPO
BCL2
H19
CCR7
MUC2
PLAT
MUC6
TXN
FGFR1
GAPDH
PRKG1
COL4A4
VDAC1
CLCN2
CTLA4
STUB1
MIR17
TNPO3
DEFB103B
PAX8

TGFBR2
IFNA1
MAPK14
TIMP2
PRSS8
VEGFC
LOXL2
SLC34A2
GBA
ADORA1
PTPN11
NTS
THBS1
FOXP3
STING1
TFR2
FGF10
NLRC4
TLR1
MIR140
GNRH1
REG3A
CD8A
CD40LG
BBS9
F5
FHIT
CCR4
TWIST1
TLR5
SELE
FADD
HLA-A
FGF23
IL12A
CLDN4
CHUK
SIRT1

MYC
MIR29C
CYP1A1
MIR122
CST3
ADCY10
TNNT2
PSMC6
TNFRSF11B
CALD1
ITGA3
MIR31
GPR35
MIR497
NPHS2
MIR15A
MIR210
PIBF1
IGHE
MUTYH
RPS27A
VWF
ENTPD1
TRPC6
ACTN4
MMP14
NR3C2
NAT2
BARD1
EPX
MIR142
FIP1L1
TNFSF10
VCL
TNFSF15
RAD51
THY1
SERPINC1

SOX9
VTN
EZH2
MIRLET7I
RET
AMFR
ACTB
CAT
MRE11
KRT8
ITGAV
NPPB
MTHFR
SOCS3
KRT13
HLA-DQB1
NPM1
RHOA
CLEC7A
SLC37A4
CHGA
PECAM1
MIR145
IL15
PI3
GBE1
CALCA
ANXA6
FURIN
MIR29A
PPARA
HSPA1A
MIR221
THPO
MAP3K1
APOL1
TNFRSF1B
ERCC1

LEPQTL1
MIR204
KITLG
PTK2
SPIB
LMNA
PRKN
SHH
GJA1
SYP
TBX4
PRG2
RGCC
CASP1
HBEGF
CDKN1A
HLA-G
MTTP
IL12B
SERPINA3
PLAUR
BAX
ENDO1
FARSB
ANGPT1
CCR5
MALAT1
AHSG
ACTA1
MIR10B
NPPA
PMM2
PTCH1
PDCD1
CDX2
MYH11
TCF4
KL

GPC3
SOD3
TYMP
MT-ATP6
IGF1R
NUP133
IL7
CX3CR1
CD2AP
IFNA2
SEMA4D
POU2AF1
MMEL1
TUBB
HOTAIR
MIR338
PRL
MIRLET7C
MIR429
SST
CADM1
CEBPB
MAGI2
TTC7A
TFRC
TRIM28
IL13RA2
BMP2
ARHGAP31
KRT5
S100A12
CLDN1
C11orf65
HBA1
ALG9
MVK
CTNNA1
FLT1

PIK3CG
RAD50
MIR483
INF2
PI4KA
FGFR2
GZMB
YAP1
ASL
RETN
TGIF1
PIP
DDR2
TFF1
ALK
PCNA
BSG
MMP13
CASR
ALPP
CCND1
GHRL
PTH
LBR
TTC37
CD28
IL2RB
DGUOK
F13A1
MIR29B1
DOCK6
H2AC18
PLOD2
FGA
NEU1
TNNI3
LRG1
HPSE

MEG3
CD86
ADM
BAAT
CD163
MIRLET7G
RAPSN
PON1
LEPR
NQO1
GAST
COQ8B
FGF1
SOCS1
RINT1
MIR148A
NR5A1
TNFSF13B
PLA2G2A
ABCC2
MT-CYB
ITGA4
KIF20A
CCL7
XIAP
JAK1
FAP
EGR1
TIMP3
WFDC2
GDF15
TNC
HMGA2
ASAH1
NME1
LPA
SELL
F2RL1

RELA
GSTT1
CFH
CETP
ETS1
ERBB4
SKIV2L
LDLR
ITGA6
MIR126
CFB
PLA2G6
TJP1
MYD88
ADK
ILK
PLA2G7
TYMS
TLR3
IL32
A2M
MFF-DT
NUP85
RPS6KB1
NR3C1
CDH23
STK11
LGALS1
EPOR
ARHGDIA
CD46
CYP1B1
ABL1
MYO1E
PLCE1
JAG1
DNMT1
SNAI2

MECOM
SERPINB2
C5
SERPINF2
SP1
MAP2K1
TYR
RIPK2
CD209
GFAP
ENO2
TOP1
GPER1
RBPJ
VEGFD
TSC1
ITGA5
ZEB1
IGFBP1
CDKN2B-AS1
IL1RL1
ADORA2A
NPC1
KDR
EOGT
TNFSF11
MKI67
DPP4
CSF1R
LTA
ITGB3
CDKN1B
ZEB2
FASN
MSR1
XRCC1
ADAM17
XDH

UROD
CASP9
NUP93
RAF1
PROM1
CSF3R
AIP
COMP
IL18R1
NR0B2
FOXM1
AREG
CRYAB
CCN1
RAC2
SAA1
IL16
NPY
CHIT1
CCR1
STAT4
ACVR1B
SREBF1
TAC1
TKT
TEK
PRKCSH
RUNX3
IFIH1
NGF
CA12
TRIM37
TP63
C5AR1
NUP160
NPPC
DAAM2
IL1RAPL2

MAPK10
IFNB1
ID1
NAMPT
CD59
KRT14
CDK2
HSD17B4
MIR146B
RNU7-1
MIR222
EHMT2
FST
UCP2
RASGRP1
IL11
PRG4
NTRK1
CCL26
CD63
YBX1
RNF168
CD68
WNT5A
CDH3
CBL
LRP1
LDHA
CTSD
CFHR5
CPA1
PGK1
PHB1
GLI1
CX3CL1
ITGAL
FCN2
POLD1

ABCA1
ANPEP
MAP2K4
SLC26A3
NUP205
MIR92A1
BMP4
TREM1
INHBA
NSD1
PALLD
CRB2
DEFA1
CDH11
ITGA2
SQSTM1
HDAC9
HAVCR2
SRF
CXCL16
EPAS1
TRAF6
MYBPC3
VCAN
OSM
GNAS
GLA
PLK1
EMP2
ANLN
GAPVD1
ANKFY1
NUP37
TBC1D8B
COL18A1
PLA2G4A
TNNI2
RECK

SIRT3
TNFAIP3
AIF1
TSLP
CCN3
APOC3
SCT
AQP2
LAMP1
IFNGR1
MIR27A
KRT17
GATA2
PIK3C2A
PRKAA1
DIS3L2
FCGR2B
ABCC3
FLI1
RIPK1
PRKCB
ESR2
TNFSF12
MIR29B2
TNFRSF10B
PLK2
RAC1
MYH9
LEFTY2
ITGA1
F10
LOXL1
HAX1
TFF3
LYZ
ACKR3
BCL10
BGN

CXCR5
MIR203A
PGM1
FCGR3A
IL22
AR
DNAH9
DKK1
PTN
LGALS3BP
COL4A1
NRG1
GH1
MIR494
MIR96
AXL
IL1R2
RYR3
PCSK9
ANGPTL2
ALPL
MYOD1
RIT1
TNFRSF6B
ADIPOR2
HPX
ITGB4
STXBP2
TMSB4X
MIR196A1
IGF2BP3
BCAR1
SPINT2
ENO1
PF4
EPRS1
U2AF1
REST

CYP7A1
LIPC
IKBKG
MIR152
ST2
LCP1
FABP4
PVT1
SLC30A7
CXCL6
RABL3
DEFA3
MCL1
MRPS22
NOTCH3
SCTR
CFHR1
CTSL
ORM1
CYSLTR2
HBA2
ACE2
CD177
PARK7
PYY
TRIM8
DNMT3A
PTPN3
EIF2AK4
ORAI1
TSC2
MGP
FLT4
REG1A
BMP15
IL6R
PSMC3IP
AKT2

ACTG2
MAP3K7
TNFRSF10A
GRN
MIR139
FSCN1
LRRC56
BNIP3
TLR7
DICER1
FLT3
MIR150
CD5L
NEAT1
L1CAM
TUG1
GHR
TP53BP1
VHL
BCS1L
MLN
CGB5
CDKN2B
AOC3
CEL
RAB27A
LMOD1
CCL20
LGALS9
NRP1
HRH2
SOST
NR1H3
TBXT
DHCR24
APCS
H6PD
MT-CO1

POU6F2
CSN1S1
EIF4EBP1
LY96
CD55
KIF19
MERTK
NGFR
SCARB1
NFATC1
MIR143
MIR212
CGA
PAPPA
ATP6AP2
NTRK2
GPC4
RARA
CLDN3
NFAT5
EPHA3
PRKD1
GATA1
PYCARD
COL8A1
ITPR3
SLC5A2
PXDN
S100A6
EP300
NLRP1
RACK1
MIR199A2
FBLN1
BDNF
MIR22
ALDH2
SETD2

SRY
CDK4
SAA4
INHA
APLN
MGMT
CCAT1
FUT4
G6PC3
ICOSLG
SERPINF1
RPS6KA1
MIR185
IL4R
LCN1
RPS6KA3
CYP11B2
GIPC1
CYBB
MIR10A
MIR148B
BAK1
FUT3
ATL1
WT1-AS
LIF
ADAMTSL1
IL6ST
SRSF2
MICA
RUNX1
STAT5A
CASP10
GAS5
CLU
E2F1
BECN1
MIR132

CD9
PRF1
MFGE8
AZU1
SLC2A3
ATF1
S100B
SHBG
DSG2
KLRK1
JAK3
EIF2AK3
BCL6
IRS1
SOAT2
PRKAG2
WDR73
RHOD
SETD7
SPINT1
H2AX
MIR99A
CXCL11
FCN3
UCHL1
HSPA1B
XPO1
CA2
GRK2
ICAM2
LAMA2
XRCC2
LCAT
MFAP5
ATP7B
PDE4A
AURKA
IGFBP2

MIR205
KLF4
CCNB1
PGF
MSRA
ELAVL1
IL7R
PLCG1
EGLN2
MIR141
CDC42
IL10RA
LIMK1
IL25
TRPV1
IFNGR2
LYN
TRPM7
FLNA
GATA3
TAFAZZIN
MAP3K5
SLIT2
SNCA
NR0B1
TPM4
IDO1
PDP1
MIR33A
CTHRC1
KLF11
DYNLL1
CTNNA2
MMACHC
RELB
HMGCR
GDNF
JUP

AKR1B1
MIR16-1
ASXL1
CD1A
IL13RA1
G6PD
CFI
AIM2
SOX6
ARG2
HAVCR1
P2RX7
CD247
ALG8
CPB2
CYP27B1
MIR589
MAPT
RAB7A
MIR181A1
FOXO1
ST3GAL4
AOAH
FOXO3
RBM15
NR1H2
MMP11
LTB4R2
MVP
LAMA5
AOC1
FANCD2
PTPA
SLC29A1
CCL19
JMJD6
ADCYAP1
CDK6

ERBB3
MIR25
TGFBR3
NAGLU
HDAC1
BCHE
FUT2
HSP90B1
EWSR1
FABP1
XIST
APOC2
FNDC5
TGFBI
CFD
NF1
F7
MIR130B
OXTR
CYP3A4
NOTCH2
AQP4
BAZ1A
SCARF1
ATF3
CCNA2
IRF1
SDHA
HAS2
TACR1
UTS2
GPR68
SYK
DPYD
IGKC
KIR3DL1
ROCK1
MX1

ROR2
CARD11
COG2
ST14
NCF2
ADAMTS13
CAMK2G
CDC25A
MEN1
DUSP19
IL37
CD27
LTBR
NPC2
BIRC3
MIR181B1
MIR361
DCK
CRNDE
CNR1
HNRNPC
FADS2
TPT1
MIR302A
TNFAIP6
RARS1
DUSP1
CXADR
ARRB2
SLC16A1
CCR3
DHFR
ENPP1
MAN1B1
MTAP
OXA1L
TAGLN2
SULF1

EHF
PON2
CD83
BMI1
AIMP1
NRAS
RETNLB
FSHB
STAT6
DDAH1
SATB2
C1QBP
HOXA11
DNM2
COL4A6
ITGB6
CSNK2A1
MAP3K8
MB
NID1
PTK2B
ASS1
S1PR1
BDKRB2
GALC
ATF2
SFRP2
NOTCH4
CIITA
PPBP
GSDMB
ABCG2
ACHE
ALOX5AP
SMAD6
RB1
ACSL4
MST1R

MIR125B1
BAG1
APOH
MLXIPL
CDH5
IRAK1
IDH1
FOSL2
CTNS
AFAP1-AS1
ALG1
PTGER2
CRYAA
CHEK1
THBS2
PRKDC
LPAR2
KEAP1
TPM2
MIR424
TOP2A
ATG5
CA9
NT5E
MYO18A
CFP
MIR144
LTB4R
MCAM
CYP3A5
RAN
MIAT
CD226
SMAD9
KCNQ1OT1
CNR2
CEBPA
IRAK4

ANXA3
BDKRB1
GFI1
COL4A2
F2RL2
IRS2
APOA4
NES
AIFM1
FAT4
RUNX2
TFAM
FSTL1
NCOA3
MARCO
HMBS
SCD
CLEC4E
SLC11A2
LAMB1
HAS1
FUS
TAP2
MIR542
NCR1
GNA12
LGMN
SLC5A1
CRH
POMC
NOX1
CGB3
TP73
MIR106A
TLR6
SCARB2
FAH
EEF2

FANCB
ITLN1
CIB1
B3GAT1
TMEM38B
HTRA1
CTCF
LIPJ
LIPM
PTPN1
RAPGEF3
NPY4R
MYB
FYN
RPN2
ALOX15
IKBKB
DCT
CNTF
MIR193A
PRKCE
SH3PXD2A
PROKR2
CYP2D6
ITCH
MIR19B1
ADAM10
PEX2
IL23A
ALDH1A1
AMH
SKP2
OTC
AXIN1
HYAL1
CR2
CLIC1
TYRP1

P2RY6
CYP2C9
RARRES2
UGDH
RASSF1
CXCL13
RDX
GHRH
GFER
GNRHR
LAMB2
WWOX
TNFSF14
MIR20A
MIR335
HLA-DRA
UBR5
RPS6KA2
PLA2G1B
HSPB2
NTN1
PLXNA1
CBS
ADORA3
CD151
RAD52
FERMT2
MIR627
SLC7A11
SLX4
TAGLN
GCH1
FGF9
PXN
NANOG
MIR183
RHOH
EIF5A

MIR9-1
CREBBP
LPAR3
GRB2
CCBE1
PDE4B
LNCRNA-ATB
PTPN13
C1S
MIR486-1
VLDLR
BTK
ABCC6
PRKAR1A
DDX58
PKM
LEF1
CYSLTR1
CDK1
CANT1
FPR1
SGCB
TNKS1BP1
ARRB1
JUND
GLI2
HOTTIP
TPI1
AHR
SIX2
TSHR
MITF
SIRT6
SOAT1
CYTOR
FPR2
TGIF2
VAV3

STX3
ATP8A1
ACVR1
VASH1
CCND2
CYP51A1
MC1R
APOA2
IFITM1
STAR
MCM2
MT-CO2
PPIA
PAK2
WNT3A
CCL8
CD207
PTGIR
SULT1E1
SLC1A4
GIMAP6
ZIC1
ROBO1
E2F3
MIR422A
PIK3R1
PNPLA2
TUFM
LINC00092
KCNH2
HOXA9
ID3
CXCR6
SEM1
PIK3C3
UGT1A1
ALPI
MIR495

FABP2
MMP16
VKORC1
LMX1B
BICC1
MIR93
SMAD1
LIPE
PCBP1
WRN
PTMA
MIR23B
FCAR
PPARD
ITPR1
FOXA1
YWHAZ
FZD6
CHAC1
MIR608
TBX18
IL12RB2
COX5A
SSTR2
MIR181C
SERPING1
MIR532
HEPH
FERMT3
ATP1B1
DRD2
MMP24
PTH1R
SMC4
KLK3
FHL2
SYNPO
SLC16A4

LRRFIP2
CUL1
MIR182
PIEZO1
TIAM1
CD47
GAS6
SLC2A4
GPBAR1
CYBA
MIR346
MIR296
KLF6
ETS2
ASIC5
INPP5D
FANCI
ERCC2
MMP15
PTGES
TIA1
AKR1B10
LRPAP1
VIPR1
SERBP1
MEP1B
HTN3
MIR23A
REL
ATP7A
TIMD4
SLC12A3
TBK1
AKR1A1
LMNB1
EIF2AK2
VSIG4
YWHAB

IRF8
POLR2B
BHMT
ATP2A3
ST6GAL1
SMURF1
RPTOR
ADIPOR1
BID
HMOX2
F9
MIR375
CPOX
CARD9
TBCD
KRT37
F12
PANX1
TRPA1
HTR2A
ALCAM
MIR301A
MAP1LC3A
MIR1225
TBX21
IGF2R
CS
YTHDF2
YTHDF3
SPRR1B
FANCM
FCN1
SOS1
PDE3B
STEAP3
PLCG2
RRM2
NDUFA9

XRCC5
KIR2DL3
C4B
TRIM72
GAA
MIR103A2
MYO5A
GADD45A
IFI27
PDPK1
BAD
GOT1
CDCP1
MMP17
SMARCAL1
PROS1
ATR
PTTG1
MSTN
SESN2
RGMA
NOD1
TICAM1
NEK7
TAPT1
AGXT
ABCG1
YTHDF1
TUSC3
MIRLET7B
AGRN
PRDX2
IRAK3
BIRC2
UCP3
MIR26B
MDK
SCN8A

CLEC12A
NPR1
METTL3
PIGR
CD1D
TNFRSF12A
SERPINA4
FOXP1
HK2
PAX6
IGF2BP2
CDKN2C
CPT1A
FBXW7
RASSF2
PLCD1
GLUL
ATG14
P4HA1
ZFYVE9
SOX18
ADCY1
APP
PRRC2C
MCM7
HGFAC
MIRLET7E
SERPINB4
RPL36A-HNRNPH2
MYOG
TUB
ID2
NEO1
MAPK7
TCN2
HDAC3
SPHK2
SEPTIN9

FGFBP1
NNMT
IL27
CXCL14
CYP2C19
IL24
CDKN1C
GPX3
RBP1
TNFRSF10C
GPR37
RXRA
PPM1D
PIM1
TH
AVP
FDFT1
DNM3
STC1
CYP11A1
ADGRE5
MIR885
GHSR
CEMIP
ARSH
RSPO1
MTDH
MAD2L2
DLEU2
PITX2
AQP8
PROCR
CD7
CDC25C
MIR32
GLO1
POLR2A
KDM6B

GDF11
CORO1A
CPQ
TCF21
HSD11B2
MT-ND1
CLINT1
HDAC7
NFATC3
IFNAR1
APLNR
GP9
MTR
NSF
ALOX12
UBB
PON3
MIR217
CTSK
NTF4
DIABLO
TSG101
G3BP1
MIR544A
CSTB
SMO
THBS4
PRKCH
GCG
PF4V1
TLN1
NCAM1
HRH1
UBE2D3
MIRLET7A1
FCER2
HPRT1
FRS2

LINC01194
MIR138-1
FABP12
PRDM1
METAP2
S100A2
HCK
DNM3OS
MACC1
INTS2
CHRNA7
M6PR
INPP4A
DLX5
ACKR2
PPP1R15A
COL25A1
NAF1
MUC5B
FAM111B
PHOX2A
TULP3
KIF21A
ZCCHC8
NOP10
PARN
TUBB3
RPA1
RTEL1
CFTRP1
AREL1
USP10
GOPC
FBRSL1
MAFK
FBRS
MAFB
MAF

MAFG
MAFA
MAFF
FLT-3
PDGFR
VEGFR
