# Supplementary material for: An integrated network pharmacology and proteomics approach reveals the anti-fibrotic effect of Fushen Granule on peritoneal fibrosis
Source: BMC Complement Med Ther. 2026 Mar 9;26:143. doi: 10.1186/s12906-026-05333-2 (PMC13085474; doi:10.1186/s12906-026-05333-2)
Supplement: Supplementary file 4 — Supplementary Material 4. [file 12906_2026_5333_MOESM4_ESM.pdf]

Article title: An Integrated Network Pharmacology and Proteomics Approach Reveals the Anti-fibrotic Effect of Fushen Granule on Peritoneal Fibrosis
Author names: Kang Yang, Jie Li, Lin Wang, Hangxing Yu, Xinyue Liu, Zhiqing Gao, Zheng Wang, Linqi Zhang, Hongtao Yang
Affiliation and e-mail address of the corresponding author: First Teaching Hospital of Tianjin University of Traditional Chinese Medicine, tjtcmht@126.com

133 overlapped genes and their 90 mapped active components in "Component-Target network"			
Node1	Node2	Net	MolName
MOL002670	KCNH2	target	Cavidine
MOL002670	SCN5A	target	Cavidine
MOL002670	RXRA	target	Cavidine
MOL002670	HSP90AA1	target	Cavidine
MOL002714	AR	target	baicalein
MOL002714	HSP90AA1	target	baicalein
MOL002714	PRSS1	target	baicalein
MOL002714	RELA	target	baicalein
MOL002714	BCL2	target	baicalein
MOL002714	FOS	target	baicalein
MOL002714	BAX	target	baicalein
MOL002714	TP53	target	baicalein
MOL002714	CDK1	target	baicalein
MOL002714	CCNB1	target	baicalein
MOL002714	CYCS	target	baicalein
MOL000358	HSP90AA1	target	beta-sitosterol
MOL000358	KCNH2	target	beta-sitosterol
MOL000358	SCN5A	target	beta-sitosterol
MOL000358	BCL2	target	beta-sitosterol
MOL000358	BAX	target	beta-sitosterol
MOL000449	RXRA	target	Stigmasterol
MOL000449	SCN5A	target	Stigmasterol
MOL000519	AR	target	coniferin
MOL000519	SCN5A	target	coniferin
MOL000519	CDK2	target	coniferin
MOL006957	AR	target	(3S,6S)-3-(benzyl)-6-(4-hydroxybenzyl)piperazine-2,5-quinone
MOL004328	HSP90AA1	target	naringenin
MOL004328	RELA	target	naringenin
MOL004328	BCL2	target	naringenin
MOL004328	BAD	target	naringenin
MOL004328	MTTP	target	naringenin
MOL004328	CYP19A1	target	naringenin
MOL004328	ADIPOQ	target	naringenin

MOL005100	HSP90AA1	target	5,7-dihydroxy-2-(3-hydroxy-4-methoxyphenyl)chroman-4-one
MOL005100	SCN5A	target	5,7-dihydroxy-2-(3-hydroxy-4-methoxyphenyl)chroman-4-one
MOL005815	KCNH2	target	Citromitin
MOL005815	SCN5A	target	Citromitin
MOL005815	HSP90AA1	target	Citromitin
MOL005828	KCNH2	target	nobiletin
MOL005828	AR	target	nobiletin
MOL005828	HSP90AA1	target	nobiletin
MOL005828	PRSS1	target	nobiletin
MOL005828	SCN5A	target	nobiletin
MOL005828	BCL2	target	nobiletin
MOL005828	BAX	target	nobiletin
MOL005828	TP53	target	nobiletin
MOL005828	TIMP1	target	nobiletin
MOL005828	CD163	target	nobiletin
MOL002235	AR	target	EUPATIN
MOL002235	HSP90AA1	target	EUPATIN
MOL002235	PRSS1	target	EUPATIN
MOL002235	SCN5A	target	EUPATIN
MOL002268	HSP90AA1	target	rhein
MOL002281	HSP90AA1	target	Toralactone
MOL000471	HSP90AA1	target	aloe-emodin
MOL000471	CDKN1A	target	aloe-emodin
MOL000471	BAX	target	aloe-emodin
MOL000471	TNF	target	aloe-emodin
MOL000471	TP53	target	aloe-emodin
MOL000471	CDK1	target	aloe-emodin
MOL000471	MYC	target	aloe-emodin
MOL000471	CCNB1	target	aloe-emodin
MOL000096	HSP90AA1	target	(-)-catechin
MOL000096	KLF7	target	(-)-catechin
MOL001601	SCN5A	target	1,2,5,6-tetrahydrotanshinone
MOL001601	RXRA	target	1,2,5,6-tetrahydrotanshinone
MOL001601	HSP90AA1	target	1,2,5,6-tetrahydrotanshinone
MOL002222	SCN5A	target	sugiol
MOL002222	DRD2	target	sugiol
MOL002651	AR	target	Dehydrotanshinone II A
MOL002651	SCN5A	target	Dehydrotanshinone II A

MOL000569	HSP90AA1	target	digallate
MOL000006	AR	target	luteolin
MOL000006	HSP90AA1	target	luteolin
MOL000006	PRSS1	target	luteolin
MOL000006	RELA	target	luteolin
MOL000006	CCND1	target	luteolin
MOL000006	CDKN1A	target	luteolin
MOL000006	RB1	target	luteolin
MOL000006	CDK4	target	luteolin
MOL000006	TNF	target	luteolin
MOL000006	IL6R	target	luteolin
MOL000006	TP53	target	luteolin
MOL000006	NFKBIA	target	luteolin
MOL000006	APP	target	luteolin
MOL000006	MCL1	target	luteolin
MOL000006	BIRC5	target	luteolin
MOL000006	IL2RA	target	luteolin
MOL000006	CCNB1	target	luteolin
MOL000006	IL4R	target	luteolin
MOL000006	XIAP	target	luteolin
MOL000006	SLC2A4	target	luteolin
MOL007036	SCN5A	target	5,6-dihydroxy-7-isopropyl-1,1-dimethyl-2,3-dihydrophenanthren-4-one
MOL007036	RXRA	target	5,6-dihydroxy-7-isopropyl-1,1-dimethyl-2,3-dihydrophenanthren-4-one
MOL007041	AR	target	2-isopropyl-8-methylphenanthrene-3,4-dione
MOL007041	SCN5A	target	2-isopropyl-8-methylphenanthrene-3,4-dione
MOL007041	RXRA	target	2-isopropyl-8-methylphenanthrene-3,4-dione
MOL007041	CDK2	target	2-isopropyl-8-methylphenanthrene-3,4-dione
MOL007045	SCN5A	target	3α-hydroxytanshinone II a
MOL007045	PRSS1	target	3α-hydroxytanshinone II a
MOL007048	HSP90AA1	target	(E)-3-[2-(3,4-dihydroxyphenyl)-7-hydroxy-benzofuran-4-yl]acrylic acid
MOL007049	AR	target	4-methylenemiltirone
MOL007049	SCN5A	target	4-methylenemiltirone
MOL007049	RXRA	target	4-methylenemiltirone
MOL007049	DRD2	target	4-methylenemiltirone
MOL007050	AR	target	2-(4-hydroxy-3-methoxyphenyl)-5-(3-hydroxypropyl)-7-methoxy-3-benzofurancarboxaldehyde
MOL007050	HSP90AA1	target	2-(4-hydroxy-3-methoxyphenyl)-5-(3-hydroxypropyl)-7-methoxy-3-benzofurancarboxaldehyde
MOL007050	CDK2	target	2-(4-hydroxy-3-methoxyphenyl)-5-(3-hydroxypropyl)-7-methoxy-3-benzofurancarboxaldehyde
MOL007058	AR	target	formyltanshinone

MOL007058	RXRA	target	formyltanshinone
MOL007059	RXRA	target	3-beta-Hydroxymethyllenetanshiquinone
MOL007059	HSP90AA1	target	3-beta-Hydroxymethyllenetanshiquinone
MOL007059	PRSS1	target	3-beta-Hydroxymethyllenetanshiquinone
MOL007061	SCN5A	target	Methylenetanshinquinone
MOL007061	RXRA	target	Methylenetanshinquinone
MOL007061	HSP90AA1	target	Methylenetanshinquinone
MOL007061	PRSS1	target	Methylenetanshinquinone
MOL007068	RXRA	target	Przewaquinone B
MOL007068	HSP90AA1	target	Przewaquinone B
MOL007068	PRSS1	target	Przewaquinone B
MOL007069	SCN5A	target	przewaquinone c
MOL007069	HSP90AA1	target	przewaquinone c
MOL007070	HSP90AA1	target	(6S,7R)-6,7-dihydroxy-1,6-dimethyl-8,9-dihydro-7H-naphtho[8,7-g]benzofuran-10,11-dione
MOL007070	PRSS1	target	(6S,7R)-6,7-dihydroxy-1,6-dimethyl-8,9-dihydro-7H-naphtho[8,7-g]benzofuran-10,11-dione
MOL007071	PRSS1	target	przewaquinone f
MOL007079	PRSS1	target	tanshinaldehyde
MOL007081	HSP90AA1	target	Danshenol B
MOL007082	KCNH2	target	Danshenol A
MOL007082	SCN5A	target	Danshenol A
MOL007082	RXRA	target	Danshenol A
MOL007085	AR	target	Salvilenone
MOL007088	SCN5A	target	cryptotanshinone
MOL007088	RELA	target	cryptotanshinone
MOL007088	STAT3	target	cryptotanshinone
MOL007088	CCND1	target	cryptotanshinone
MOL007088	TNF	target	cryptotanshinone
MOL007088	APP	target	cryptotanshinone
MOL007088	EDN1	target	cryptotanshinone
MOL007088	BIRC5	target	cryptotanshinone
MOL007093	KCNH2	target	dan-shexinkum d
MOL007093	AR	target	dan-shexinkum d
MOL007093	SCN5A	target	dan-shexinkum d
MOL007093	RXRA	target	dan-shexinkum d
MOL007093	CDK2	target	dan-shexinkum d
MOL007093	PRSS1	target	dan-shexinkum d
MOL007094	SCN5A	target	danshenspiroketallactone
MOL007094	RXRA	target	danshenspiroketallactone

MOL007094	HSP90AA1	target	danshenspiroketallactone
MOL007098	AR	target	deoxyneocryptotanshinone
MOL007098	SCN5A	target	deoxyneocryptotanshinone
MOL007098	RXRA	target	deoxyneocryptotanshinone
MOL007098	CDK2	target	deoxyneocryptotanshinone
MOL007100	AR	target	dihydrotanshinlactone
MOL007100	SCN5A	target	dihydrotanshinlactone
MOL007100	RXRA	target	dihydrotanshinlactone
MOL007100	PRSS1	target	dihydrotanshinlactone
MOL007101	SCN5A	target	dihydrotanshinone Ⅰ
MOL007101	RXRA	target	dihydrotanshinone Ⅰ
MOL007101	HSP90AA1	target	dihydrotanshinone Ⅰ
MOL007105	SCN5A	target	epidanshenspiroketallactone
MOL007105	RXRA	target	epidanshenspiroketallactone
MOL007105	HSP90AA1	target	epidanshenspiroketallactone
MOL007105	CDK2	target	epidanshenspiroketallactone
MOL007107	SCN5A	target	C09092
MOL007108	AR	target	isocryptotanshi-none
MOL007108	SCN5A	target	isocryptotanshi-none
MOL007108	RXRA	target	isocryptotanshi-none
MOL007108	DRD2	target	isocryptotanshi-none
MOL007108	CDK2	target	isocryptotanshi-none
MOL007108	PRSS1	target	isocryptotanshi-none
MOL007111	AR	target	Isotanshinone II
MOL007111	SCN5A	target	Isotanshinone II
MOL007111	RXRA	target	Isotanshinone II
MOL007111	CDK2	target	Isotanshinone II
MOL007119	AR	target	miltionone Ⅰ
MOL007119	SCN5A	target	miltionone Ⅰ
MOL007119	RXRA	target	miltionone Ⅰ
MOL007119	CDK2	target	miltionone Ⅰ
MOL007122	AR	target	Miltirone
MOL007122	SCN5A	target	Miltirone
MOL007122	RXRA	target	Miltirone
MOL007124	AR	target	neocryptotanshinone ii
MOL007124	SCN5A	target	neocryptotanshinone ii
MOL007124	RXRA	target	neocryptotanshinone ii
MOL007124	HSP90AA1	target	neocryptotanshinone ii

MOL007124	CDK2	target	neocryptotanshinone ii
MOL007125	SCN5A	target	neocryptotanshinone
MOL007127	SCN5A	target	1-methyl-8,9-dihydro-7H-naphtho[5,6-g]benzofuran-6,10,11-trione
MOL007127	RXRA	target	1-methyl-8,9-dihydro-7H-naphtho[5,6-g]benzofuran-6,10,11-trione
MOL007127	HSP90AA1	target	1-methyl-8,9-dihydro-7H-naphtho[5,6-g]benzofuran-6,10,11-trione
MOL007130	AR	target	prolithospermic acid
MOL007130	HSP90AA1	target	prolithospermic acid
MOL007130	PRSS1	target	prolithospermic acid
MOL007132	AR	target	(2R)-3-(3,4-dihydroxyphenyl)-2-[(Z)-3-(3,4-dihydroxyphenyl)acryloyl]oxy-propionic acid
MOL007132	PRSS1	target	(2R)-3-(3,4-dihydroxyphenyl)-2-[(Z)-3-(3,4-dihydroxyphenyl)acryloyl]oxy-propionic acid
MOL007142	PRSS1	target	salvianolic acid j
MOL007143	RXRA	target	salvilenone Ⅰ
MOL007145	SCN5A	target	salviolone
MOL007145	DRD2	target	salviolone
MOL007150	HSP90AA1	target	(6S)-6-hydroxy-1-methyl-6-methylol-8,9-dihydro-7H-naphtho[8,7-g]benzofuran-10,11-quinone
MOL007150	PRSS1	target	(6S)-6-hydroxy-1-methyl-6-methylol-8,9-dihydro-7H-naphtho[8,7-g]benzofuran-10,11-quinone
MOL007151	HSP90AA1	target	Tanshindiol B
MOL007152	HSP90AA1	target	Przewaquinone E
MOL007154	SCN5A	target	tanshinone iia
MOL007154	RXRA	target	tanshinone iia
MOL007154	RELA	target	tanshinone iia
MOL007154	BCL2	target	tanshinone iia
MOL007154	FOS	target	tanshinone iia
MOL007154	CDKN1A	target	tanshinone iia
MOL007154	TP53	target	tanshinone iia
MOL007154	NFKBIA	target	tanshinone iia
MOL007154	EDNRA	target	tanshinone iia
MOL007154	EDN1	target	tanshinone iia
MOL007154	MYC	target	tanshinone iia
MOL007154	ITGB3	target	tanshinone iia
MOL007155	SCN5A	target	(6S)-6-(hydroxymethyl)-1,6-dimethyl-8,9-dihydro-7H-naphtho[8,7-g]benzofuran-10,11-dione
MOL007155	PRSS1	target	(6S)-6-(hydroxymethyl)-1,6-dimethyl-8,9-dihydro-7H-naphtho[8,7-g]benzofuran-10,11-dione
MOL007156	AR	target	tanshinone Ⅵ
MOL007156	SCN5A	target	tanshinone Ⅵ
MOL007156	HSP90AA1	target	tanshinone Ⅵ
MOL001040	HSP90AA1	target	(2R)-5,7-dihydroxy-2-(4-hydroxyphenyl)chroman-4-one
MOL000422	AR	target	kaempferol
MOL000422	HSP90AA1	target	kaempferol

MOL000422	PRSS1	target	kaempferol
MOL000422	RELA	target	kaempferol
MOL000422	BCL2	target	kaempferol
MOL000422	BAX	target	kaempferol
MOL000422	TNF	target	kaempferol
MOL000422	STAT1	target	kaempferol
MOL000422	CDK1	target	kaempferol
MOL000422	SELE	target	kaempferol
MOL000422	SLC2A4	target	kaempferol
MOL000098	AR	target	quercetin
MOL000098	HSP90AA1	target	quercetin
MOL000098	PRSS1	target	quercetin
MOL000098	KCNH2	target	quercetin
MOL000098	SCN5A	target	quercetin
MOL000098	RXRA	target	quercetin
MOL000098	RELA	target	quercetin
MOL000098	CCND1	target	quercetin
MOL000098	BCL2	target	quercetin
MOL000098	FOS	target	quercetin
MOL000098	CDKN1A	target	quercetin
MOL000098	BAX	target	quercetin
MOL000098	RB1	target	quercetin
MOL000098	TNF	target	quercetin
MOL000098	IL6R	target	quercetin
MOL000098	TP53	target	quercetin
MOL000098	NFKBIA	target	quercetin
MOL000098	STAT1	target	quercetin
MOL000098	CDK1	target	quercetin
MOL000098	CAV1	target	quercetin
MOL000098	MYC	target	quercetin
MOL000098	GJA1	target	quercetin
MOL000098	CCL2	target	quercetin
MOL000098	SELE	target	quercetin
MOL000098	BIRC5	target	quercetin
MOL000098	SULT1E1	target	quercetin
MOL000098	IL2RA	target	quercetin
MOL000098	CCNB1	target	quercetin
MOL000098	COL1A1	target	quercetin

MOL000098	ABCG2	target	quercetin
MOL000098	SLC2A4	target	quercetin
MOL000098	COL3A1	target	quercetin
MOL000098	CXCL11	target	quercetin
MOL000098	CXCL2	target	quercetin
MOL000098	CLDN4	target	quercetin
MOL000098	CXCL10	target	quercetin
MOL000098	SPP1	target	quercetin
MOL000098	RUNX2	target	quercetin
MOL000098	RASSF1	target	quercetin
MOL000239	AR	target	Jaranol
MOL000239	SCN5A	target	Jaranol
MOL000239	HSP90AA1	target	Jaranol
MOL000239	CDK2	target	Jaranol
MOL000239	PRSS1	target	Jaranol
MOL000296	SCN5A	target	hederagenin
MOL000296	RXRA	target	hederagenin
MOL000354	AR	target	isorhamnetin
MOL000354	HSP90AA1	target	isorhamnetin
MOL000354	CDK2	target	isorhamnetin
MOL000354	PRSS1	target	isorhamnetin
MOL000354	RELA	target	isorhamnetin
MOL000371	SCN5A	target	3,9-di-O-methylnissolin
MOL000371	RXRA	target	3,9-di-O-methylnissolin
MOL000371	PRSS1	target	3,9-di-O-methylnissolin
MOL000378	KCNH2	target	7-O-methylisomucronulatol
MOL000378	AR	target	7-O-methylisomucronulatol
MOL000378	SCN5A	target	7-O-methylisomucronulatol
MOL000378	RXRA	target	7-O-methylisomucronulatol
MOL000378	HSP90AA1	target	7-O-methylisomucronulatol
MOL000378	CDK2	target	7-O-methylisomucronulatol
MOL000378	PRSS1	target	7-O-methylisomucronulatol
MOL000380	SCN5A	target	(6aR,11aR)-9,10-dimethoxy-6a,11a-dihydro-6H-benzofurano[3,2-c]chromen-3-ol
MOL000380	RXRA	target	(6aR,11aR)-9,10-dimethoxy-6a,11a-dihydro-6H-benzofurano[3,2-c]chromen-3-ol
MOL000380	HSP90AA1	target	(6aR,11aR)-9,10-dimethoxy-6a,11a-dihydro-6H-benzofurano[3,2-c]chromen-3-ol
MOL000380	PRSS1	target	(6aR,11aR)-9,10-dimethoxy-6a,11a-dihydro-6H-benzofurano[3,2-c]chromen-3-ol
MOL000387	HSP90AA1	target	Bifendate
MOL000392	AR	target	formononetin

MOL000392	RXRA	target	formononetin
MOL000392	HSP90AA1	target	formononetin
MOL000392	CDK2	target	formononetin
MOL000392	PRSS1	target	formononetin
MOL000392	IL4R	target	formononetin
MOL000417	AR	target	Calycosin
MOL000417	RXRA	target	Calycosin
MOL000417	HSP90AA1	target	Calycosin
MOL000417	CDK2	target	Calycosin
MOL000417	PRSS1	target	Calycosin
MOL000433	CDK2	target	FA
MOL000442	RXRA	target	1,7-Dihydroxy-3,9-dimethoxy pterocarpene
MOL000442	HSP90AA1	target	1,7-Dihydroxy-3,9-dimethoxy pterocarpene
MOL000442	PRSS1	target	1,7-Dihydroxy-3,9-dimethoxy pterocarpene
MOL001645	RXRA	target	Linoleyl acetate
MOL001792	RXRA	target	DFV
MOL001792	HSP90AA1	target	DFV
MOL003044	AR	target	Chryseriol
MOL003044	HSP90AA1	target	Chryseriol
MOL003044	CDK2	target	Chryseriol
MOL003044	PRSS1	target	Chryseriol
MOL003542	AR	target	8-Isopentenyl-kaempferol
MOL003542	HSP90AA1	target	8-Isopentenyl-kaempferol
MOL003542	CDK2	target	8-Isopentenyl-kaempferol
MOL003542	PRSS1	target	8-Isopentenyl-kaempferol
MOL003542	SCN5A	target	8-Isopentenyl-kaempferol
MOL003542	RXRA	target	8-Isopentenyl-kaempferol
MOL003542	KCNH2	target	8-Isopentenyl-kaempferol
MOL004367	HSP90AA1	target	olivil
MOL004373	KCNH2	target	Anhydroicaritin
MOL004373	AR	target	Anhydroicaritin
MOL004373	SCN5A	target	Anhydroicaritin
MOL004373	RXRA	target	Anhydroicaritin
MOL004373	HSP90AA1	target	Anhydroicaritin
MOL004373	CDK2	target	Anhydroicaritin
MOL004373	PRSS1	target	Anhydroicaritin
MOL004380	KCNH2	target	C-Homoerythrinan, 1,6-didehydro-3,15,16-trimethoxy-, (3.beta.)-
MOL004380	AR	target	C-Homoerythrinan, 1,6-didehydro-3,15,16-trimethoxy-, (3.beta.)-

MOL004380	SCN5A	target	C-Homoerythrinan, 1,6-didehydro-3,15,16-trimethoxy-, (3.beta.)-
MOL004380	DRD2	target	C-Homoerythrinan, 1,6-didehydro-3,15,16-trimethoxy-, (3.beta.)-
MOL004382	AR	target	Yinyanghuo A
MOL004382	PRSS1	target	Yinyanghuo A
MOL004384	AR	target	Yinyanghuo C
MOL004384	CDK2	target	Yinyanghuo C
MOL004384	PRSS1	target	Yinyanghuo C
MOL004386	AR	target	Yinyanghuo E
MOL004386	PRSS1	target	Yinyanghuo E
MOL004388	HSP90AA1	target	6-hydroxy-11,12-dimethoxy-2,2-dimethyl-1,8-dioxo-2,3,4,8-tetrahydro-1H-isochromeno[3,4-h]isoquinolin-2-ium
MOL004391	AR	target	8-(3-methylbut-2-enyl)-2-phenyl-chromone
MOL004391	SCN5A	target	8-(3-methylbut-2-enyl)-2-phenyl-chromone
MOL004391	RXRA	target	8-(3-methylbut-2-enyl)-2-phenyl-chromone
MOL004391	HSP90AA1	target	8-(3-methylbut-2-enyl)-2-phenyl-chromone
MOL004391	CDK2	target	8-(3-methylbut-2-enyl)-2-phenyl-chromone
MOL004396	SCN5A	target	1,2-bis(4-hydroxy-3-methoxyphenyl)propan-1,3-diol
MOL004396	HSP90AA1	target	1,2-bis(4-hydroxy-3-methoxyphenyl)propan-1,3-diol
MOL004396	CDK2	target	1,2-bis(4-hydroxy-3-methoxyphenyl)propan-1,3-diol
MOL000006	NOX4	target	luteolin
MOL000006	AKR1B1	target	luteolin
MOL000006	XDH	target	luteolin
MOL000006	FLT3	target	luteolin
MOL000006	CA2	target	luteolin
MOL000006	ALOX5	target	luteolin
MOL000006	ADORA1	target	luteolin
MOL000006	GLO1	target	luteolin
MOL000006	SYK	target	luteolin
MOL000006	GSK3B	target	luteolin
MOL000006	PARP1	target	luteolin
MOL000006	TTR	target	luteolin
MOL000006	MMP9	target	luteolin
MOL000006	CA12	target	luteolin
MOL000006	MMP2	target	luteolin
MOL000006	MMP12	target	luteolin
MOL000006	CYP1B1	target	luteolin
MOL000006	ABCG2	target	luteolin
MOL000006	AKR1B10	target	luteolin
MOL000006	TOP1	target	luteolin

MOL000006	ARG1	target	luteolin
MOL000006	CDK6	target	luteolin
MOL000006	PTGS2	target	luteolin
MOL000006	CFTR	target	luteolin
MOL000098	IGF1R	target	quercetin
MOL000098	CYP19A1	target	quercetin
MOL000098	EGFR	target	quercetin
MOL000098	F2	target	quercetin
MOL000098	PIM1	target	quercetin
MOL000098	MPO	target	quercetin
MOL000098	PIK3R1	target	quercetin
MOL000098	ADORA2A	target	quercetin
MOL000098	SRC	target	quercetin
MOL000098	PTK2	target	quercetin
MOL000098	KDR	target	quercetin
MOL000098	MMP13	target	quercetin
MOL000098	MMP3	target	quercetin
MOL000098	ALOX15	target	quercetin
MOL000098	ABCC1	target	quercetin
MOL000098	PLK1	target	quercetin
MOL000098	CA9	target	quercetin
MOL000098	CSNK2A1	target	quercetin
MOL000098	ALOX12	target	quercetin
MOL000098	MET	target	quercetin
MOL000098	CXCR1	target	quercetin
MOL000098	ALK	target	quercetin
MOL000098	AKT1	target	quercetin
MOL000098	ABCB1	target	quercetin
MOL000098	PLA2G1B	target	quercetin
MOL000098	AXL	target	quercetin
MOL000098	NUAK1	target	quercetin
MOL000098	AKR1C3	target	quercetin
MOL000098	AKR1A1	target	quercetin
MOL000098	GPR35	target	quercetin
MOL000098	MAPT	target	quercetin
MOL000098	TOP2A	target	quercetin
MOL000098	INSR	target	quercetin
MOL000098	PIK3CG	target	quercetin

MOL000098	APEX1	target	quercetin
MOL000098	CDK2	target	quercetin
MOL000098	TYR	target	quercetin
MOL000098	AHR	target	quercetin
MOL000296	AR	target	hederagenin
MOL000296	HMGCR	target	hederagenin
MOL000296	CYP51A1	target	hederagenin
MOL000392	IL2	target	formononetin
MOL000422	CTSD	target	kaempferol
MOL000433	TYMS	target	FA
MOL000433	DHFR	target	FA
MOL000449	NR1H3	target	Stigmasterol
MOL002670	DRD2	target	Cavidine
MOL002670	F3	target	Cavidine
MOL004328	SHBG	target	naringenin
MOL004328	ESR1	target	naringenin
MOL004328	ESR2	target	naringenin
MOL004328	PTGS1	target	naringenin
MOL007088	ACHE	target	cryptotanshinone
MOL007101	PTPN11	target	dihydrotanshinone Ⅰ
MOL007132	MMP1	target	(2R)-3-(3,4-dihydroxyphenyl)-2-[(Z)-3-(3,4-dihydroxyphenyl)acryloyl]oxy-propionic acid
MOL007154	TERT	target	tanshinone iia
