# Supplementary material for: An integrated network pharmacology and proteomics approach reveals the anti-fibrotic effect of Fushen Granule on peritoneal fibrosis
Source: BMC Complement Med Ther. 2026 Mar 9;26:143. doi: 10.1186/s12906-026-05333-2 (PMC13085474; doi:10.1186/s12906-026-05333-2)
Supplement: Supplementary file 5 — Supplementary Material 5. [file 12906_2026_5333_MOESM5_ESM.pdf]

Article title: An Integrated Network Pharmacology and Proteomics Approach Reveals the Anti-fibrotic Effect of Fushen Granule on Peritoneal Fibrosis
Author names: Kang Yang, Jie Li, Lin Wang, Hangxing Yu, Xinyue Liu, Zhiqing Gao, Zheng Wang, Linqi Zhang, Hongtao Yang
Affiliation and e-mail address of the corresponding author: First Teaching Hospital of Tianjin University of Traditional Chinese Medicine, tjtcmht@126.com

The 90 mapped active components of "Component-Target network" ranked according to "MOL. score"

name	degree.layout	Banxia	Chenpi	Dahuang	Danggui	Danshen	Guijianyu	Huangqi	Yinyanghuo
MOL000098	77	0	0	0	0	0	1	1	1
MOL000006	44	0	0	0	0	1	0	0	1
MOL007154	13	0	0	0	0	1	0	0	0
MOL000422	12	0	0	0	0	0	1	1	1
MOL004328	11	0	1	0	0	0	0	0	0
MOL002714	11	1	0	0	0	0	0	0	0
MOL005828	10	0	1	0	0	0	0	0	0
MOL007088	9	0	0	0	0	1	0	0	0
MOL000471	8	0	0	1	0	0	0	0	0
MOL004373	7	0	0	0	0	0	0	0	1
MOL003542	7	0	0	0	0	0	0	0	1
MOL000392	7	0	0	0	0	0	0	1	0
MOL000378	7	0	0	0	0	0	0	1	0
MOL007108	6	0	0	0	0	1	0	0	0
MOL007093	6	0	0	0	0	1	0	0	0
MOL002670	6	1	0	0	0	0	0	0	0
MOL004391	5	0	0	0	0	0	0	0	1
MOL000417	5	0	0	0	0	0	0	1	0
MOL000354	5	0	0	0	0	0	0	1	0
MOL000296	5	0	0	0	0	0	0	1	0
MOL000239	5	0	0	0	0	0	0	1	0
MOL007124	5	0	0	0	0	1	0	0	0
MOL000358	5	1	0	1	1	0	1	0	0
MOL004380	4	0	0	0	0	0	0	0	1
MOL003044	4	0	0	0	0	0	0	0	1
MOL000380	4	0	0	0	0	0	0	1	0
MOL007119	4	0	0	0	0	1	0	0	0
MOL007111	4	0	0	0	0	1	0	0	0
MOL007105	4	0	0	0	0	1	0	0	0
MOL007101	4	0	0	0	0	1	0	0	0
MOL007100	4	0	0	0	0	1	0	0	0
MOL007098	4	0	0	0	0	1	0	0	0
MOL007061	4	0	0	0	0	1	0	0	0

MOL007049	4	0	0	0	0	1	0	0	0
MOL007041	4	0	0	0	0	1	0	0	0
MOL002235	4	0	0	1	0	0	0	0	0
MOL004396	3	0	0	0	0	0	0	0	1
MOL004384	3	0	0	0	0	0	0	0	1
MOL000442	3	0	0	0	0	0	0	1	0
MOL000433	3	0	0	0	0	0	0	1	0
MOL000371	3	0	0	0	0	0	0	1	0
MOL007156	3	0	0	0	0	1	0	0	0
MOL007132	3	0	0	0	0	1	0	0	0
MOL007130	3	0	0	0	0	1	0	0	0
MOL007127	3	0	0	0	0	1	0	0	0
MOL007122	3	0	0	0	0	1	0	0	0
MOL007094	3	0	0	0	0	1	0	0	0
MOL007082	3	0	0	0	0	1	0	0	0
MOL007068	3	0	0	0	0	1	0	0	0
MOL007059	3	0	0	0	0	1	0	0	0
MOL007050	3	0	0	0	0	1	0	0	0
MOL001601	3	0	0	0	0	1	0	0	0
MOL005815	3	0	1	0	0	0	0	0	0
MOL000519	3	1	0	0	0	0	0	0	0
MOL000449	3	1	0	0	1	0	0	0	0
MOL004386	2	0	0	0	0	0	0	0	1
MOL004382	2	0	0	0	0	0	0	0	1
MOL001792	2	0	0	0	0	0	0	0	1
MOL007155	2	0	0	0	0	1	0	0	0
MOL007150	2	0	0	0	0	1	0	0	0
MOL007145	2	0	0	0	0	1	0	0	0
MOL007070	2	0	0	0	0	1	0	0	0
MOL007069	2	0	0	0	0	1	0	0	0
MOL007058	2	0	0	0	0	1	0	0	0
MOL007045	2	0	0	0	0	1	0	0	0
MOL007036	2	0	0	0	0	1	0	0	0
MOL002651	2	0	0	0	0	1	0	0	0
MOL002222	2	0	0	0	0	1	0	0	0
MOL000096	2	0	0	1	0	0	0	0	0
MOL005100	2	0	1	0	0	0	1	0	0
MOL004388	1	0	0	0	0	0	0	0	1

MOL004367	1	0	0	0	0	0	0	0	1
MOL001645	1	0	0	0	0	0	0	0	1
MOL000387	1	0	0	0	0	0	0	1	0
MOL001040	1	0	0	0	0	0	1	0	0
MOL007152	1	0	0	0	0	1	0	0	0
MOL007151	1	0	0	0	0	1	0	0	0
MOL007143	1	0	0	0	0	1	0	0	0
MOL007142	1	0	0	0	0	1	0	0	0
MOL007125	1	0	0	0	0	1	0	0	0
MOL007107	1	0	0	0	0	1	0	0	0
MOL007085	1	0	0	0	0	1	0	0	0
MOL007081	1	0	0	0	0	1	0	0	0
MOL007079	1	0	0	0	0	1	0	0	0
MOL007071	1	0	0	0	0	1	0	0	0
MOL007048	1	0	0	0	0	1	0	0	0
MOL000569	1	0	0	0	0	1	0	0	0
MOL002281	1	0	0	1	0	0	0	0	0
MOL002268	1	0	0	1	0	0	0	0	0
MOL006957	1	1	0	0	0	0	0	0	0
