# Supplementary material for: An integrated network pharmacology and proteomics approach reveals the anti-fibrotic effect of Fushen Granule on peritoneal fibrosis
Source: BMC Complement Med Ther. 2026 Mar 9;26:143. doi: 10.1186/s12906-026-05333-2 (PMC13085474; doi:10.1186/s12906-026-05333-2)
Supplement: Supplementary file 6 — Supplementary Material 6. [file 12906_2026_5333_MOESM6_ESM.pdf]

Article title: An Integrated Network Pharmacology and Proteomics Approach Reveals the Anti-fibrotic Effect of Fushen Granule on Peritoneal Fibrosis
Author names: Kang Yang, Jie Li, Lin Wang, Hangxing Yu, Xinyue Liu, Zhiqing Gao, Zheng Wang, Linqi Zhang, Hongtao Yang
Affiliation and e-mail address of the corresponding author: First Teaching Hospital of Tianjin University of Traditional Chinese Medicine, tjtcmht@126.com

GO enrichment analysis of BP, CC, and MF of the 133 overlapped genes

ONTOI	ID	Description	GeneRatio	BgRatio	pvalue	p.adjust	qvalue	geneID	Count
BP	GO:1901653	cellular response to peptide	29/132	374/18870	2.80E-22	1.14E-18	5.23E-19	RELA/FOS/TP53/ADIPOQ/TNF/RB1/APP/SLC2A4/STAT	29
BP	GO:0009410	response to xenobiotic stimulus	30/132	434/18870	1.34E-21	2.74E-18	1.25E-18	KCNH2/HSP90AA1/RELA/BCL2/FOS/TP53/CDK1/ADIP	30
BP	GO:0062197	cellular response to chemical stress	26/132	317/18870	1.26E-20	1.71E-17	7.84E-18	RELA/FOS/TP53/CDK1/BAD/SLC2A4/EDN1/EDNRA/C	26
BP	GO:0006979	response to oxidative stress	28/132	400/18870	2.55E-20	2.60E-17	1.19E-17	RELA/BCL2/FOS/TP53/CDK1/ADIPOQ/APP/EDN1/EDN	28
BP	GO:0043434	response to peptide hormone	28/132	430/18870	1.75E-19	1.43E-16	6.55E-17	RELA/FOS/ADIPOQ/TIMP1/RB1/SLC2A4/STAT3/EDN1/	28
BP	GO:0048511	rhythmic process	24/132	296/18870	5.77E-19	3.93E-16	1.80E-16	TP53/CDK1/MTTP/ADIPOQ/TNF/DRD2/CLDN4/ADOR	24
BP	GO:0051347	positive regulation of transferase activity	27/132	414/18870	8.11E-19	4.34E-16	1.98E-16	HSP90AA1/BAD/ADIPOQ/CDKN1A/TNF/MYC/CCND1/	27
BP	GO:0033674	positive regulation of kinase activity	25/132	337/18870	8.50E-19	4.34E-16	1.98E-16	HSP90AA1/BAD/ADIPOQ/CDKN1A/TNF/CCND1/IL6R/	25
BP	GO:0071375	cellular response to peptide hormone stimulus	24/132	310/18870	1.69E-18	7.67E-16	3.51E-16	RELA/FOS/ADIPOQ/RB1/SLC2A4/STAT3/EDN1/EDNR	24
BP	GO:0048545	response to steroid hormone	24/132	330/18870	7.18E-18	2.93E-15	1.34E-15	RXRA/AR/RELA/BCL2/FOS/ADIPOQ/CDKN1A/TNF/ED	24
BP	GO:0050727	regulation of inflammatory response	26/132	425/18870	1.87E-17	6.93E-15	3.17E-15	RELA/CYP19A1/ADIPOQ/TNF/RB1/NFKBIA/APP/IL2RA	26
BP	GO:1901654	response to ketone	20/132	208/18870	2.20E-17	7.49E-15	3.42E-15	AR/RELA/FOS/CDKN1A/CDK4/EDN1/CAV1/CLDN4/SP	20
BP	GO:0034599	cellular response to oxidative stress	21/132	255/18870	7.98E-17	2.51E-14	1.15E-14	RELA/FOS/TP53/CDK1/EDN1/EDNRA/ALOX5/PARP1/	21
BP	GO:0000302	response to reactive oxygen species	19/132	205/18870	2.92E-16	8.53E-14	3.90E-14	RELA/BCL2/FOS/CDK1/EDN1/STAT1/COL1A1/MMP9/	19
BP	GO:0050673	epithelial cell proliferation	26/132	480/18870	3.57E-16	9.72E-14	4.44E-14	SCN5A/AR/BAX/BAD/CDKN1A/TNF/MYC/DRD2/CCN	26
BP	GO:0009411	response to UV	17/132	152/18870	4.95E-16	1.26E-13	5.78E-14	RELA/BCL2/BAX/TP53/TIMP1/CDKN1A/MYC/CCND1/	17
BP	GO:0050678	regulation of epithelial cell proliferation	24/132	407/18870	8.44E-16	1.92E-13	8.76E-14	SCN5A/AR/BAX/BAD/TNF/MYC/DRD2/CCND1/RB1/C	24
BP	GO:0034764	positive regulation of transmembrane transport	19/132	217/18870	8.45E-16	1.92E-13	8.76E-14	KCNH2/BAX/ADIPOQ/EDN1/EDNRA/CAV1/CCL2/CXC	19
BP	GO:1904645	response to amyloid-beta	12/132	53/18870	1.72E-15	3.71E-13	1.69E-13	TNF/APP/GJA1/GSK3B/PARP1/MMP9/MMP2/MMP12/	12
BP	GO:0009416	response to light stimulus	21/132	318/18870	6.83E-15	1.38E-12	6.30E-13	RELA/BCL2/FOS/BAX/TP53/TIMP1/CDKN1A/MYC/DR	21
BP	GO:0031667	response to nutrient levels	25/132	495/18870	7.09E-15	1.38E-12	6.30E-13	RXRA/RELA/BCL2/FOS/BAX/TP53/ADIPOQ/CDKN1A/T	25
BP	GO:0034614	cellular response to reactive oxygen species	16/132	154/18870	1.23E-14	2.28E-12	1.04E-12	RELA/FOS/CDK1/EDN1/MMP9/MMP2/CYP1B1/EGFR/	16
BP	GO:0070141	response to UV-A	8/132	14/18870	1.34E-14	2.37E-12	1.08E-12	TIMP1/CCND1/MMP9/MMP2/EGFR/MMP3/AKT1/MM	8
BP	GO:0043491	phosphatidylinositol 3-kinase/protein kinase B signal transduction	20/132	290/18870	1.39E-14	2.37E-12	1.08E-12	HSP90AA1/TNF/DRD2/STAT3/EDN1/NOX4/XDH/FLT3	20
BP	GO:0009636	response to toxic substance	19/132	258/18870	2.04E-14	3.33E-12	1.52E-12	BCL2/FOS/BAX/CDK1/CDKN1A/TNF/DRD2/ABCG2/CY	19
BP	GO:0048144	fibroblast proliferation	14/132	107/18870	2.33E-14	3.66E-12	1.67E-12	BAX/TP53/CDK1/CCNB1/CDKN1A/MYC/CDK4/ITGB3/	14
BP	GO:0009314	response to radiation	23/132	428/18870	2.60E-14	3.94E-12	1.80E-12	RELA/BCL2/FOS/BAX/TP53/TIMP1/CDKN1A/MYC/DR	23
BP	GO:0070482	response to oxygen levels	21/132	343/18870	3.05E-14	4.45E-12	2.03E-12	BCL2/FOS/TP53/BAD/ADIPOQ/CDKN1A/TNF/MYC/DR	21
BP	GO:0032102	negative regulation of response to external stimulus	24/132	481/18870	3.42E-14	4.82E-12	2.20E-12	CYP19A1/ADIPOQ/TNF/DRD2/RB1/IL2RA/STAT3/EDN	24
BP	GO:0050900	leukocyte migration	22/132	396/18870	5.13E-14	6.99E-12	3.20E-12	CYP19A1/TNF/IL6R/APP/EDN1/ITGB3/SELE/CCL2/CXC	22
BP	GO:0018108	peptidyl-tyrosine phosphorylation	19/132	276/18870	6.93E-14	9.13E-12	4.18E-12	TP53/ADIPOQ/TNF/IL6R/APP/ITGB3/CAV1/NOX4/FLT	19
BP	GO:0018212	peptidyl-tyrosine modification	19/132	278/18870	7.90E-14	1.01E-11	4.61E-12	TP53/ADIPOQ/TNF/IL6R/APP/ITGB3/CAV1/NOX4/FLT	19
BP	GO:0033002	muscle cell proliferation	18/132	247/18870	1.25E-13	1.54E-11	7.05E-12	FOS/CDK1/ADIPOQ/CDKN1A/TNF/IL6R/EDN1/ITGB3/	18
BP	GO:0009612	response to mechanical stimulus	17/132	213/18870	1.41E-13	1.70E-11	7.77E-12	RELA/FOS/BAD/TNF/DRD2/NFKBIA/EDN1/ITGB3/STAT	17
BP	GO:0048661	positive regulation of smooth muscle cell proliferation	13/132	98/18870	1.71E-13	1.99E-11	9.11E-12	TNF/IL6R/EDN1/ITGB3/STAT1/GJA1/MMP9/MMP2/IG	13
BP	GO:0007584	response to nutrient	15/132	155/18870	2.48E-13	2.82E-11	1.29E-11	RXRA/RELA/ADIPOQ/STAT1/COL1A1/CXCL10/SPP1/R	15
BP	GO:0071216	cellular response to biotic stimulus	18/132	265/18870	4.16E-13	4.60E-11	2.10E-11	RELA/TP53/TNF/CDK4/NFKBIA/CCL2/CXCL11/CXCL2/	18
BP	GO:0048732	gland development	22/132	443/18870	4.94E-13	5.31E-11	2.43E-11	AR/RELA/BCL2/BAX/CYP19A1/TNF/DRD2/CCND1/ED	22
BP	GO:0043281	regulation of cysteine-type endopeptidase activity involved in apoptotic p	15/132	163/18870	5.22E-13	5.47E-11	2.50E-11	BAX/CYCS/BAD/TNF/MYC/XIAP/XDH/SYK/MMP9/SRC	15
BP	GO:0052547	regulation of peptidase activity	19/132	312/18870	6.23E-13	6.37E-11	2.91E-11	BAX/CYCS/BAD/TIMP1/TNF/MYC/XIAP/STAT3/CAV1/	19
BP	GO:0072593	reactive oxygen species metabolic process	17/132	234/18870	6.58E-13	6.55E-11	3.00E-11	BCL2/TP53/CDKN1A/TNF/EDN1/NOX4/XDH/ALOX5/S	17
BP	GO:0036293	response to decreased oxygen levels	19/132	315/18870	7.39E-13	7.19E-11	3.29E-11	BCL2/FOS/TP53/BAD/ADIPOQ/TNF/MYC/DRD2/SLC2	19
BP	GO:0007623	circadian rhythm	16/132	204/18870	1.01E-12	9.59E-11	4.38E-11	TP53/CDK1/MTTP/ADIPOQ/TNF/DRD2/CLDN4/ADOR	16
BP	GO:0048660	regulation of smooth muscle cell proliferation	15/132	171/18870	1.06E-12	9.80E-11	4.48E-11	ADIPOQ/CDKN1A/TNF/IL6R/EDN1/ITGB3/STAT1/GJA1	15

BP	GO:0048145	regulation of fibroblast proliferation	12/132	89/18870	1.24E-12	1.13E-10	5.15E-11	BAX/TP53/CCNB1/CDKN1A/MYC/CDK4/ITGB3/CAV1/	12
BP	GO:0002237	response to molecule of bacterial origin	20/132	369/18870	1.27E-12	1.13E-10	5.15E-11	RELA/FOS/TNF/CDK4/NFKBIA/EDN1/SELE/CCL2/CXCL	20
BP	GO:0048659	smooth muscle cell proliferation	15/132	175/18870	1.48E-12	1.29E-10	5.88E-11	ADIPOQ/CDKN1A/TNF/IL6R/EDN1/ITGB3/STAT1/GJA1	15
BP	GO:1904019	epithelial cell apoptotic process	14/132	144/18870	1.52E-12	1.29E-10	5.90E-11	BCL2/BAX/TNF/RB1/EDNRA/ITGB3/CCL2/IGF1R/KDR/	14
BP	GO:0042060	wound healing	21/132	423/18870	1.78E-12	1.48E-10	6.77E-11	TIMP1/CDKN1A/TNF/EDN1/ITGB3/CAV1/COL3A1/CLD	21
BP	GO:0043410	positive regulation of MAPK cascade	22/132	474/18870	1.90E-12	1.55E-10	7.09E-11	AR/TNF/DRD2/APP/XIAP/EDN1/ITGB3/CCL2/NOX4/XD	22
BP	GO:0031960	response to corticosteroid	14/132	149/18870	2.43E-12	1.95E-10	8.89E-11	BCL2/FOS/ADIPOQ/CDKN1A/TNF/EDN1/FLT3/PARP1/	14
BP	GO:0097305	response to alcohol	17/132	254/18870	2.48E-12	1.95E-10	8.89E-11	FOS/CDK1/ADIPOQ/CDKN1A/TNF/DRD2/CDK4/SLC2	17
BP	GO:0001666	response to hypoxia	18/132	298/18870	3.04E-12	2.34E-10	1.07E-10	BCL2/FOS/TP53/BAD/ADIPOQ/TNF/MYC/DRD2/SLC2	18
BP	GO:0034612	response to tumor necrosis factor	17/132	259/18870	3.39E-12	2.56E-10	1.17E-10	RELA/FOS/TP53/ADIPOQ/TNF/NFKBIA/XIAP/SLC2A4/	17
BP	GO:0042391	regulation of membrane potential	21/132	440/18870	3.76E-12	2.79E-10	1.28E-10	KCNH2/SCN5A/BCL2/BAX/BAD/TNF/DRD2/APP/EDN1	21
BP	GO:0032496	response to lipopolysaccharide	19/132	348/18870	4.28E-12	3.12E-10	1.43E-10	RELA/FOS/TNF/CDK4/NFKBIA/EDN1/SELE/CCL2/CXCL	19
BP	GO:2001233	regulation of apoptotic signaling pathway	20/132	398/18870	5.07E-12	3.63E-10	1.66E-10	AR/RELA/BCL2/BAX/TP53/BAD/TNF/MYC/RB1/MCL1/	20
BP	GO:0001819	positive regulation of cytokine production	22/132	499/18870	5.23E-12	3.69E-10	1.68E-10	HSP90AA1/RELA/ADIPOQ/TNF/DRD2/IL6R/APP/IL4R/	22
BP	GO:0034504	protein localization to nucleus	18/132	310/18870	5.89E-12	4.08E-10	1.86E-10	HSP90AA1/TP53/CDK1/CDKN1A/NFKBIA/STAT3/COL1	18
BP	GO:0032868	response to insulin	17/132	269/18870	6.21E-12	4.23E-10	1.93E-10	RELA/FOS/ADIPOQ/RB1/SLC2A4/STAT1/COL1A1/GSK	17
BP	GO:0032570	response to progesterone	9/132	40/18870	6.93E-12	4.64E-10	2.12E-10	RELA/FOS/CAV1/CLDN4/CYP1B1/IGF1R/SRC/NR1H3/	9
BP	GO:2000116	regulation of cysteine-type endopeptidase activity	15/132	195/18870	7.12E-12	4.69E-10	2.14E-10	BAX/CYCS/BAD/TNF/MYC/XIAP/XDH/SYK/MMP9/SRC	15
BP	GO:0050878	regulation of body fluid levels	19/132	365/18870	9.83E-12	6.37E-10	2.91E-10	DRD2/CCND1/EDN1/ITGB3/CAV1/COL3A1/AKR1B1/X	19
BP	GO:0031348	negative regulation of defense response	18/132	321/18870	1.05E-11	6.73E-10	3.07E-10	CYP19A1/ADIPOQ/DRD2/RB1/IL2RA/STAT3/ALOX5/A	18
BP	GO:0045860	positive regulation of protein kinase activity	17/132	282/18870	1.32E-11	8.27E-10	3.78E-10	HSP90AA1/ADIPOQ/CDKN1A/TNF/CCND1/IL6R/EDN1	17
BP	GO:0032869	cellular response to insulin stimulus	15/132	206/18870	1.57E-11	9.56E-10	4.37E-10	RELA/ADIPOQ/RB1/SLC2A4/STAT1/GSK3B/PARP1/IGF	15
BP	GO:0044703	multi-organism reproductive process	15/132	206/18870	1.57E-11	9.56E-10	4.37E-10	AR/BCL2/FOS/TIMP1/EDN1/ITGB3/GJA1/CLDN4/SPP1	15
BP	GO:0051897	positive regulation of phosphatidylinositol 3-kinase/protein kinase B signa	14/132	171/18870	1.60E-11	9.59E-10	4.38E-10	HSP90AA1/TNF/NOX4/FLT3/IGF1R/EGFR/SRC/PTK2/K	14
BP	GO:0052548	regulation of endopeptidase activity	17/132	288/18870	1.84E-11	1.09E-09	4.97E-10	BAX/CYCS/BAD/TIMP1/TNF/MYC/XIAP/STAT3/XDH/S	17
BP	GO:0051896	regulation of phosphatidylinositol 3-kinase/protein kinase B signal transdu	16/132	248/18870	1.98E-11	1.14E-09	5.21E-10	HSP90AA1/TNF/DRD2/NOX4/XDH/FLT3/IGF1R/EGFR/	16
BP	GO:0033273	response to vitamin	11/132	86/18870	2.02E-11	1.14E-09	5.21E-10	RXRA/RELA/COL1A1/CXCL10/SPP1/RUNX2/IGF1R/PIM	11
BP	GO:0071214	cellular response to abiotic stimulus	18/132	334/18870	2.04E-11	1.14E-09	5.21E-10	BAX/TP53/BAD/TIMP1/CDKN1A/MYC/SLC2A4/ITGB3/	18
BP	GO:0104004	cellular response to environmental stimulus	18/132	334/18870	2.04E-11	1.14E-09	5.21E-10	BAX/TP53/BAD/TIMP1/CDKN1A/MYC/SLC2A4/ITGB3/	18
BP	GO:1904035	regulation of epithelial cell apoptotic process	12/132	113/18870	2.24E-11	1.24E-09	5.66E-10	BCL2/BAX/TNF/RB1/ITGB3/CCL2/IGF1R/KDR/PLA2G1B	12
BP	GO:0014074	response to purine-containing compound	13/132	143/18870	2.33E-11	1.27E-09	5.81E-10	RELA/FOS/ADIPOQ/STAT1/COL1A1/ADORA1/CYP1B1	13
BP	GO:0002064	epithelial cell development	15/132	214/18870	2.70E-11	1.43E-09	6.55E-10	AR/BAD/ADIPOQ/CDKN1A/TNF/EDNRA/AKR1B1/GSK	15
BP	GO:0043270	positive regulation of monoatomic ion transport	15/132	214/18870	2.70E-11	1.43E-09	6.55E-10	KCNH2/SCN5A/BAX/EDN1/EDNRA/CAV1/CCL2/CXCL	15
BP	GO:0044706	multi-multicellular organism process	15/132	216/18870	3.09E-11	1.62E-09	7.39E-10	AR/BCL2/FOS/TIMP1/EDN1/ITGB3/GJA1/CLDN4/SPP1	15
BP	GO:2000377	regulation of reactive oxygen species metabolic process	13/132	147/18870	3.31E-11	1.71E-09	7.82E-10	BCL2/TP53/CDKN1A/TNF/XDH/ALOX5/SYK/CYP1B1/F	13
BP	GO:0034644	cellular response to UV	11/132	90/18870	3.35E-11	1.71E-09	7.82E-10	BAX/TP53/TIMP1/CDKN1A/MYC/PARP1/MMP9/MMP	11
BP	GO:0001659	temperature homeostasis	14/132	183/18870	3.99E-11	2.01E-09	9.20E-10	ADIPOQ/TNF/DRD2/RB1/IL4R/STAT3/CAV1/GJA1/AD	14
BP	GO:0048771	tissue remodeling	14/132	184/18870	4.29E-11	2.14E-09	9.77E-10	BAX/TP53/TIMP1/EDNRA/ITGB3/CAV1/GJA1/SPP1/NC	14
BP	GO:0045862	positive regulation of proteolysis	18/132	350/18870	4.40E-11	2.17E-09	9.90E-10	BAX/CYCS/BAD/TNF/MYC/STAT3/CAV1/CLDN4/XDH/	18
BP	GO:0007565	female pregnancy	14/132	186/18870	4.96E-11	2.41E-09	1.10E-09	AR/BCL2/FOS/TIMP1/ITGB3/GJA1/CLDN4/SPP1/PARP	14
BP	GO:0006869	lipid transport	20/132	453/18870	5.22E-11	2.51E-09	1.15E-09	RXRA/MTTP/CYP19A1/ADIPOQ/DRD2/NFKBIA/EDN1/	20
BP	GO:1902893	regulation of miRNA transcription	10/132	70/18870	5.52E-11	2.62E-09	1.20E-09	AR/RELA/FOS/TP53/TNF/MYC/STAT3/EGFR/TERT/ESR	10
BP	GO:0061614	miRNA transcription	10/132	71/18870	6.39E-11	3.00E-09	1.37E-09	AR/RELA/FOS/TP53/TNF/MYC/STAT3/EGFR/TERT/ESR	10
BP	GO:0097191	extrinsic apoptotic signaling pathway	15/132	229/18870	7.07E-11	3.28E-09	1.50E-09	AR/RELA/BCL2/BAX/BAD/TNF/IL6R/MCL1/CAV1/GSK3	15
BP	GO:0006690	icosanoid metabolic process	12/132	125/18870	7.45E-11	3.42E-09	1.56E-09	EDN1/AKR1B1/ALOX5/SYK/CYP1B1/ALOX15/ABCC1/	12
BP	GO:1902895	positive regulation of miRNA transcription	9/132	52/18870	8.69E-11	3.94E-09	1.80E-09	AR/RELA/FOS/TP53/TNF/MYC/STAT3/EGFR/TERT	9
BP	GO:0046683	response to organophosphorus	12/132	127/18870	8.98E-11	4.02E-09	1.84E-09	RELA/FOS/ADIPOQ/STAT1/COL1A1/CYP1B1/TYMS/PI	12
BP	GO:0097193	intrinsic apoptotic signaling pathway	17/132	319/18870	9.14E-11	4.02E-09	1.84E-09	BCL2/BAX/TP53/CYCS/BAD/CDKN1A/TNF/MYC/MCL1	17
BP	GO:1902074	response to salt	18/132	366/18870	9.14E-11	4.02E-09	1.84E-09	SCN5A/HSP90AA1/FOS/MTTP/ADIPOQ/TNF/DRD2/E	18

BP	GO:0048146	positive regulation of fibroblast proliferation	9/132	53/18870	1.04E-10	4.52E-09	2.07E-09	CCNB1/CDKN1A/MYC/CDK4/ITGB3/EGFR/PLA2G1B/C	9
BP	GO:0071356	cellular response to tumor necrosis factor	15/132	238/18870	1.22E-10	5.20E-09	2.38E-09	RELA/FOS/TP53/ADIPOQ/TNF/NFKBIA/XIAP/SLC2A4/E	15
BP	GO:0060326	cell chemotaxis	17/132	325/18870	1.22E-10	5.20E-09	2.38E-09	CYP19A1/IL6R/EDN1/CCL2/CXCL11/CXCL2/CXCL10/A	17
BP	GO:0071219	cellular response to molecule of bacterial origin	15/132	239/18870	1.29E-10	5.44E-09	2.49E-09	RELA/TNF/CDK4/NFKBIA/CCL2/CXCL11/CXCL2/CXCL1	15
BP	GO:1901655	cellular response to ketone	11/132	103/18870	1.49E-10	6.20E-09	2.83E-09	AR/FOS/CDK4/SPP1/CYP1B1/IGF1R/SRC/AKT1/AKR1C	11
BP	GO:0010827	regulation of glucose transmembrane transport	10/132	78/18870	1.67E-10	6.89E-09	3.15E-09	ADIPOQ/TNF/EDN1/EDNRA/PIK3R1/AKT1/PLA2G1B/P	10
BP	GO:0070371	ERK1 and ERK2 cascade	17/132	336/18870	2.05E-10	8.36E-09	3.82E-09	CDK1/ADIPOQ/TNF/MYC/DRD2/APP/EDN1/ITGB3/CC	17
BP	GO:0071453	cellular response to oxygen levels	13/132	174/18870	2.76E-10	1.12E-08	5.10E-09	BCL2/FOS/TP53/BAD/MYC/DRD2/SLC2A4/EDN1/CAV	13
BP	GO:0001503	ossification	19/132	444/18870	2.80E-10	1.12E-08	5.13E-09	RXRA/BCL2/TNF/IL6R/COL1A1/SPP1/RUNX2/ALOX5/G	19
BP	GO:0010959	regulation of metal ion transport	18/132	398/18870	3.55E-10	1.41E-08	6.44E-09	KCNH2/SCN5A/BCL2/BAX/DRD2/ITGB3/CAV1/CCL2/C	18
BP	GO:0051591	response to cAMP	10/132	85/18870	3.99E-10	1.57E-08	7.16E-09	RELA/FOS/ADIPOQ/STAT1/COL1A1/CYP1B1/PIK3CG/T	10
BP	GO:0034765	regulation of monoatomic ion transmembrane transport	19/132	454/18870	4.08E-10	1.58E-08	7.21E-09	KCNH2/SCN5A/BCL2/BAX/DRD2/EDN1/EDNRA/ITGB3	19
BP	GO:0050679	positive regulation of epithelial cell proliferation	14/132	218/18870	4.09E-10	1.58E-08	7.21E-09	SCN5A/AR/BAD/TNF/MYC/CCND1/STAT3/ITGB3/RUN	14
BP	GO:0030522	intracellular receptor signaling pathway	17/132	353/18870	4.38E-10	1.67E-08	7.64E-09	RXRA/AR/RELA/TNF/NFKBIA/XIAP/STAT3/CAV1/PARP	17
BP	GO:2000628	regulation of miRNA metabolic process	10/132	86/18870	4.49E-10	1.70E-08	7.75E-09	AR/RELA/FOS/TP53/TNF/MYC/STAT3/EGFR/TERT/ESR	10
BP	GO:0090068	positive regulation of cell cycle process	15/132	262/18870	4.66E-10	1.75E-08	7.98E-09	CDK1/CCNB1/TNF/DRD2/CCND1/RB1/CDK4/APP/BIR	15
BP	GO:1901990	regulation of mitotic cell cycle phase transition	17/132	355/18870	4.77E-10	1.77E-08	8.10E-09	BCL2/TP53/CDK1/CCNB1/CDK2/CDKN1A/CCND1/RB1	17
BP	GO:1905952	regulation of lipid localization	13/132	183/18870	5.16E-10	1.90E-08	8.68E-09	RXRA/CYP19A1/ADIPOQ/TNF/NFKBIA/EDN1/ITGB3/C	13
BP	GO:0010038	response to metal ion	17/132	359/18870	5.66E-10	2.07E-08	9.44E-09	SCN5A/BCL2/FOS/CDK1/MTTP/DRD2/EDN1/CAV1/PA	17
BP	GO:0007162	negative regulation of cell adhesion	16/132	312/18870	5.95E-10	2.13E-08	9.75E-09	ADIPOQ/IL2RA/IL4R/COL1A1/MMP2/MMP12/CYP1B1	16
BP	GO:1904659	glucose transmembrane transport	11/132	117/18870	5.95E-10	2.13E-08	9.75E-09	ADIPOQ/TNF/SLC2A4/EDN1/EDNRA/PIK3R1/AKT1/PL	11
BP	GO:2000630	positive regulation of miRNA metabolic process	9/132	64/18870	6.06E-10	2.15E-08	9.84E-09	AR/RELA/FOS/TP53/TNF/MYC/STAT3/EGFR/TERT	9
BP	GO:0071222	cellular response to lipopolysaccharide	14/132	226/18870	6.56E-10	2.31E-08	1.06E-08	RELA/TNF/CDK4/NFKBIA/CCL2/CXCL11/CXCL2/CXCL1	14
BP	GO:0006935	chemotaxis	19/132	468/18870	6.77E-10	2.36E-08	1.08E-08	CYP19A1/IL6R/EDN1/ITGB3/CCL2/CXCL11/CXCL2/CX	19
BP	GO:1904062	regulation of monoatomic cation transmembrane transport	16/132	315/18870	6.84E-10	2.37E-08	1.08E-08	KCNH2/SCN5A/BCL2/BAX/DRD2/EDN1/EDNRA/ITGB3	16
BP	GO:0051051	negative regulation of transport	19/132	469/18870	7.02E-10	2.41E-08	1.10E-08	KCNH2/BCL2/ADIPOQ/TNF/KLF7/DRD2/EDN1/ITGB3/	19
BP	GO:0042330	taxis	19/132	470/18870	7.27E-10	2.45E-08	1.12E-08	CYP19A1/IL6R/EDN1/ITGB3/CCL2/CXCL11/CXCL2/CX	19
BP	GO:0044772	mitotic cell cycle phase transition	19/132	470/18870	7.27E-10	2.45E-08	1.12E-08	BCL2/TP53/CDK1/CCNB1/CDK2/CDKN1A/MYC/CCND	19
BP	GO:0032409	regulation of transporter activity	15/132	271/18870	7.44E-10	2.49E-08	1.14E-08	RXRA/BCL2/ADIPOQ/DRD2/APP/EDN1/EDNRA/CAV1/	15
BP	GO:0008645	hexose transmembrane transport	11/132	120/18870	7.83E-10	2.60E-08	1.19E-08	ADIPOQ/TNF/SLC2A4/EDN1/EDNRA/PIK3R1/AKT1/PL	11
BP	GO:0032368	regulation of lipid transport	12/132	153/18870	7.91E-10	2.61E-08	1.19E-08	RXRA/CYP19A1/ADIPOQ/NFKBIA/EDN1/ITGB3/CAV1/	12
BP	GO:0050730	regulation of peptidyl-tyrosine phosphorylation	14/132	231/18870	8.74E-10	2.86E-08	1.31E-08	TP53/ADIPOQ/TNF/IL6R/APP/ITGB3/CAV1/NOX4/FLT	14
BP	GO:0140747	regulation of ncRNA transcription	10/132	92/18870	8.82E-10	2.86E-08	1.31E-08	AR/RELA/FOS/TP53/TNF/MYC/STAT3/EGFR/TERT/ESR	10
BP	GO:1903829	positive regulation of protein localization	19/132	476/18870	8.98E-10	2.89E-08	1.32E-08	HSP90AA1/CDK1/BAD/TNF/GSK3B/PARP1/EGFR/F2/PI	19
BP	GO:0038034	signal transduction in absence of ligand	9/132	67/18870	9.24E-10	2.93E-08	1.34E-08	BCL2/BAX/BAD/TNF/MCL1/GSK3B/AKT1/IL2/TERT	9
BP	GO:0097192	extrinsic apoptotic signaling pathway in absence of ligand	9/132	67/18870	9.24E-10	2.93E-08	1.34E-08	BCL2/BAX/BAD/TNF/MCL1/GSK3B/AKT1/IL2/TERT	9
BP	GO:1900182	positive regulation of protein localization to nucleus	10/132	93/18870	9.82E-10	3.09E-08	1.41E-08	HSP90AA1/CDK1/PARP1/F2/PIK3R1/SRC/PLK1/AKT1/T	10
BP	GO:0015749	monosaccharide transmembrane transport	11/132	123/18870	1.02E-09	3.16E-08	1.44E-08	ADIPOQ/TNF/SLC2A4/EDN1/EDNRA/PIK3R1/AKT1/PL	11
BP	GO:0071482	cellular response to light stimulus	11/132	123/18870	1.02E-09	3.16E-08	1.44E-08	BAX/TP53/TIMP1/CDKN1A/MYC/PARP1/MMP9/MMP2	11
BP	GO:0051402	neuron apoptotic process	15/132	278/18870	1.06E-09	3.25E-08	1.48E-08	BCL2/BAX/TP53/TNF/CCND1/RB1/APP/MCL1/XIAP/CC	15
BP	GO:0045785	positive regulation of cell adhesion	19/132	482/18870	1.10E-09	3.37E-08	1.54E-08	RELA/BAD/TNF/IL2RA/IL4R/ITGB3/SELE/CAV1/CCL2/A	19
BP	GO:0034767	positive regulation of monoatomic ion transmembrane transport	12/132	158/18870	1.15E-09	3.44E-08	1.57E-08	KCNH2/BAX/EDN1/EDNRA/CAV1/CCL2/CXCL11/CXCL	12
BP	GO:0036294	cellular response to decreased oxygen levels	12/132	158/18870	1.15E-09	3.44E-08	1.57E-08	BCL2/FOS/TP53/BAD/MYC/DRD2/SLC2A4/EDN1/SRC/	12
BP	GO:0003014	renal system process	11/132	126/18870	1.32E-09	3.94E-08	1.80E-08	BCL2/ADIPOQ/DRD2/EDN1/EDNRA/ABCG2/CLDN4/A	11
BP	GO:0030595	leukocyte chemotaxis	14/132	240/18870	1.44E-09	4.22E-08	1.93E-08	CYP19A1/IL6R/EDN1/CCL2/CXCL11/CXCL2/CXCL10/A	14
BP	GO:0051384	response to glucocorticoid	11/132	127/18870	1.44E-09	4.22E-08	1.93E-08	BCL2/FOS/ADIPOQ/CDKN1A/TNF/EDN1/FLT3/CYP1B1	11
BP	GO:0046777	protein autophosphorylation	13/132	199/18870	1.45E-09	4.22E-08	1.93E-08	ADIPOQ/CAV1/FLT3/GSK3B/IGF1R/EGFR/PIM1/SRC/P	13
BP	GO:0071900	regulation of protein serine/threonine kinase activity	15/132	285/18870	1.49E-09	4.31E-08	1.97E-08	CCNB1/ADIPOQ/CDKN1A/TNF/CCND1/RB1/EDN1/FL	15
BP	GO:0045787	positive regulation of cell cycle	16/132	334/18870	1.60E-09	4.55E-08	2.08E-08	CDK1/CCNB1/TNF/DRD2/CCND1/RB1/CDK4/APP/BIR	16

BP	GO:0097529	myeloid leukocyte migration	14/132	242/18870	1.60E-09	4.55E-08	2.08E-08	CYP19A1/IL6R/EDN1/CCL2/CXCL11/CXCL2/CXCL10/SY	14
BP	GO:0022407	regulation of cell-cell adhesion	19/132	493/18870	1.60E-09	4.55E-08	2.08E-08	RELA/BAD/ADIPOQ/TNF/IL2RA/IL4R/SELE/CAV1/CCL2	19
BP	GO:2001234	negative regulation of apoptotic signaling pathway	14/132	243/18870	1.69E-09	4.76E-08	2.17E-08	AR/RELA/BCL2/BAX/TNF/RB1/MCL1/GSK3B/MMP9/SR	14
BP	GO:0050728	negative regulation of inflammatory response	13/132	202/18870	1.74E-09	4.86E-08	2.22E-08	CYP19A1/ADIPOQ/RB1/IL2RA/STAT3/ALOX5/ADORA1	13
BP	GO:0051881	regulation of mitochondrial membrane potential	9/132	73/18870	2.03E-09	5.63E-08	2.57E-08	BCL2/BAX/BAD/PARP1/ADORA2A/SRC/KDR/AKT1/MA	9
BP	GO:0050777	negative regulation of immune response	13/132	205/18870	2.08E-09	5.74E-08	2.62E-08	DRD2/IL4R/COL3A1/SYK/PARP1/MMP12/ARG1/SRC/A	13
BP	GO:0042542	response to hydrogen peroxide	10/132	101/18870	2.23E-09	6.11E-08	2.79E-08	RELA/BCL2/CDK1/EDN1/STAT1/COL1A1/MMP2/CYP1I	10
BP	GO:0035094	response to nicotine	8/132	51/18870	2.29E-09	6.23E-08	2.85E-08	RELA/BCL2/BAD/TNF/DRD2/EDN1/MMP2/IGF1R	8
BP	GO:0018105	peptidyl-serine phosphorylation	15/132	295/18870	2.38E-09	6.45E-08	2.95E-08	HSP90AA1/BCL2/BAX/CDK1/CDK2/TNF/APP/CAV1/GS	15
BP	GO:0046883	regulation of hormone secretion	14/132	251/18870	2.57E-09	6.85E-08	3.13E-08	BAD/CYP19A1/ADIPOQ/TNF/KLF7/DRD2/EDN1/GJA1/	14
BP	GO:0051924	regulation of calcium ion transport	14/132	251/18870	2.57E-09	6.85E-08	3.13E-08	BCL2/BAX/DRD2/ITGB3/CAV1/CCL2/CXCL11/CXCL10/	14
BP	GO:0010952	positive regulation of peptidase activity	12/132	170/18870	2.65E-09	7.03E-08	3.21E-08	BAX/CYCS/BAD/TNF/MYC/STAT3/CLDN4/XDH/SYK/CT	12
BP	GO:0000082	G1/S transition of mitotic cell cycle	14/132	252/18870	2.70E-09	7.12E-08	3.25E-08	BCL2/TP53/CDK1/CDK2/CDKN1A/MYC/CCND1/RB1/C	14
BP	GO:0034219	carbohydrate transmembrane transport	11/132	136/18870	2.99E-09	7.81E-08	3.57E-08	ADIPOQ/TNF/SLC2A4/EDN1/EDNRA/PIK3R1/AKT1/PL/	11
BP	GO:0009895	negative regulation of catabolic process	16/132	349/18870	3.00E-09	7.81E-08	3.57E-08	RELA/BCL2/TP53/TIMP1/TNF/MCL1/STAT3/ADORA1/E	16
BP	GO:1901987	regulation of cell cycle phase transition	18/132	456/18870	3.09E-09	7.98E-08	3.65E-08	BCL2/TP53/CDK1/CCNB1/CDK2/CDKN1A/CCND1/RB1	18
BP	GO:1903034	regulation of response to wounding	12/132	173/18870	3.23E-09	8.31E-08	3.80E-08	TNF/STAT3/EDN1/CAV1/CLDN4/SPP1/ALOX5/IGF1R/F	12
BP	GO:0010586	miRNA metabolic process	10/132	105/18870	3.27E-09	8.34E-08	3.81E-08	AR/RELA/FOS/TP53/TNF/MYC/STAT3/EGFR/TERT/ESR1	10
BP	GO:0046323	glucose import	9/132	77/18870	3.29E-09	8.34E-08	3.81E-08	ADIPOQ/TNF/SLC2A4/PIK3R1/AKT1/PLA2G1B/PTPN11	9
BP	GO:0051090	regulation of DNA-binding transcription factor activity	17/132	406/18870	3.65E-09	9.19E-08	4.20E-08	AR/RELA/TNF/RB1/NFKBIA/APP/STAT3/EDN1/CAV1/SY	17
BP	GO:0042886	amide transport	16/132	354/18870	3.68E-09	9.22E-08	4.22E-08	BAD/MTTP/TNF/KLF7/DRD2/EDN1/GJA1/ABCG2/CA2/	16
BP	GO:0071902	positive regulation of protein serine/threonine kinase activity	11/132	140/18870	4.06E-09	1.01E-07	4.63E-08	ADIPOQ/TNF/CCND1/EDN1/FLT3/EGFR/SRC/AKT1/PL	11
BP	GO:0018209	peptidyl-serine modification	15/132	308/18870	4.28E-09	1.06E-07	4.85E-08	HSP90AA1/BCL2/BAX/CDK1/CDK2/TNF/APP/CAV1/GS	15
BP	GO:0051899	membrane depolarization	9/132	80/18870	4.64E-09	1.14E-07	5.22E-08	KCNH2/SCN5A/BCL2/EDN1/CAV1/PARP1/ADORA2A/S	9
BP	GO:1900180	regulation of protein localization to nucleus	11/132	143/18870	5.08E-09	1.24E-07	5.68E-08	HSP90AA1/CDK1/GSK3B/PARP1/F2/PIK3R1/SRC/PLK1/	11
BP	GO:0006970	response to osmotic stress	9/132	81/18870	5.19E-09	1.26E-07	5.77E-08	HSP90AA1/BAX/TP53/BAD/TNF/SLC2A4/AKR1B1/ABC	9
BP	GO:0050708	regulation of protein secretion	14/132	266/18870	5.41E-09	1.31E-07	5.98E-08	BAD/TNF/KLF7/DRD2/ALOX5/F2/ADORA2A/PLA2G1B/	14
BP	GO:0043280	positive regulation of cysteine-type endopeptidase activity involved in apo	10/132	111/18870	5.63E-09	1.35E-07	6.19E-08	BAX/CYCS/BAD/TNF/MYC/XDH/SYK/CTSD/MAPT/F3	10
BP	GO:0003018	vascular process in circulatory system	14/132	269/18870	6.24E-09	1.49E-07	6.82E-08	TNF/SLC2A4/EDN1/EDNRA/CAV1/ABCG2/ADORA1/M	14
BP	GO:0032355	response to estradiol	10/132	113/18870	6.71E-09	1.59E-07	7.28E-08	CYP19A1/STAT3/COL1A1/MMP2/CYP1B1/IGF1R/EGFR	10
BP	GO:0071456	cellular response to hypoxia	11/132	147/18870	6.80E-09	1.61E-07	7.34E-08	BCL2/FOS/TP53/BAD/MYC/DRD2/SLC2A4/EDN1/SRC/	11
BP	GO:0031349	positive regulation of defense response	18/132	480/18870	6.87E-09	1.61E-07	7.37E-08	HSP90AA1/RELA/TNF/NFKBIA/APP/XIAP/CAV1/SYK/M	18
BP	GO:0071492	cellular response to UV-A	5/132	11/18870	6.93E-09	1.62E-07	7.40E-08	TIMP1/MMP9/MMP2/MMP3/MMP1	5
BP	GO:0048678	response to axon injury	9/132	84/18870	7.20E-09	1.66E-07	7.61E-08	BCL2/BAX/CDK1/DRD2/SPP1/MMP2/IGF1R/AXL/DHFR	9
BP	GO:0045088	regulation of innate immune response	17/132	425/18870	7.21E-09	1.66E-07	7.61E-08	HSP90AA1/RELA/TNF/DRD2/NFKBIA/XIAP/CAV1/SYK/	17
BP	GO:1990776	response to angiotensin	7/132	38/18870	7.36E-09	1.69E-07	7.72E-08	RELA/CAV1/COL3A1/CA2/IGF1R/SRC/PTGS2	7
BP	GO:0030198	extracellular matrix organization	15/132	321/18870	7.48E-09	1.71E-07	7.81E-08	PRSS1/TNF/RB1/APP/ITGB3/CAV1/COL1A1/COL3A1/N	15
BP	GO:0043062	extracellular structure organization	15/132	322/18870	7.80E-09	1.77E-07	8.09E-08	PRSS1/TNF/RB1/APP/ITGB3/CAV1/COL1A1/COL3A1/N	15
BP	GO:0045229	external encapsulating structure organization	15/132	323/18870	8.13E-09	1.84E-07	8.39E-08	PRSS1/TNF/RB1/APP/ITGB3/CAV1/COL1A1/COL3A1/N	15
BP	GO:1903039	positive regulation of leukocyte cell-cell adhesion	14/132	275/18870	8.27E-09	1.86E-07	8.48E-08	RELA/BAD/TNF/IL2RA/IL4R/SELE/CAV1/CCL2/ALOX5/S	14
BP	GO:0002685	regulation of leukocyte migration	13/132	230/18870	8.36E-09	1.87E-07	8.53E-08	CYP19A1/TNF/IL6R/APP/EDN1/ITGB3/SELE/CCL2/CXC	13
BP	GO:1902749	regulation of cell cycle G2/M phase transition	10/132	116/18870	8.66E-09	1.92E-07	8.79E-08	TP53/CDK1/CCNB1/CDK2/CDKN1A/CCND1/CDK4/API	10
BP	GO:0046324	regulation of glucose import	8/132	60/18870	8.73E-09	1.93E-07	8.81E-08	ADIPOQ/TNF/PIK3R1/AKT1/PLA2G1B/PTPN11/TERT/IN	8
BP	GO:2001242	regulation of intrinsic apoptotic signaling pathway	12/132	190/18870	9.34E-09	2.05E-07	9.37E-08	BCL2/BAX/TP53/BAD/MYC/MCL1/CAV1/PARP1/MMP9	12
BP	GO:0031099	regeneration	12/132	191/18870	9.90E-09	2.16E-07	9.87E-08	BCL2/CDK1/CDKN1A/TNF/CCND1/SPP1/FLT3/MMP2/	12
BP	GO:0044843	cell cycle G1/S phase transition	14/132	279/18870	9.93E-09	2.16E-07	9.87E-08	BCL2/TP53/CDK1/CDK2/CDKN1A/MYC/CCND1/RB1/C	14
BP	GO:1903037	regulation of leukocyte cell-cell adhesion	16/132	382/18870	1.08E-08	2.34E-07	1.07E-07	RELA/BAD/TNF/IL2RA/IL4R/SELE/CAV1/CCL2/ALOX5/S	16
BP	GO:0016049	cell growth	18/132	497/18870	1.18E-08	2.52E-07	1.15E-07	HSP90AA1/BCL2/TP53/CDKN1A/RB1/APP/EDN1/EDNI	18
BP	GO:0022411	cellular component disassembly	18/132	497/18870	1.18E-08	2.52E-07	1.15E-07	PRSS1/BAX/TP53/CDK1/TNF/MYC/GSK3B/MMP9/MMI	18

BP	GO:0044839	cell cycle G2/M phase transition	11/132	155/18870	1.19E-08	2.52E-07	1.15E-07	TP53/CDK1/CCNB1/CDK2/CDKN1A/CCND1/CDK4/API	11
BP	GO:0006909	phagocytosis	13/132	237/18870	1.20E-08	2.53E-07	1.16E-07	ADIPOQ/TNF/ITGB3/CCL2/ADORA1/SYK/ADORA2A/S	13
BP	GO:0048469	cell maturation	12/132	195/18870	1.25E-08	2.63E-07	1.20E-07	BCL2/CDKN1A/RB1/APP/EDN1/EDNRA/RUNX2/AKR1E	12
BP	GO:0010950	positive regulation of endopeptidase activity	11/132	156/18870	1.27E-08	2.66E-07	1.21E-07	BAX/CYCS/BAD/TNF/MYC/STAT3/XDH/SYK/CTSD/MA	11
BP	GO:0006816	calcium ion transport	17/132	445/18870	1.42E-08	2.96E-07	1.36E-07	SCN5A/BCL2/BAX/DRD2/EDN1/EDNRA/ITGB3/CAV1/(	17
BP	GO:0031334	positive regulation of protein-containing complex assembly	12/132	198/18870	1.48E-08	3.07E-07	1.41E-07	HSP90AA1/BAX/TP53/TNF/SYK/GSK3B/SRC/MMP3/AL	12
BP	GO:0042063	gliogenesis	15/132	338/18870	1.49E-08	3.08E-07	1.41E-07	RELA/TP53/CDK1/TNF/RB1/APP/STAT3/CCL2/COL3A1	15
BP	GO:0008643	carbohydrate transport	11/132	159/18870	1.55E-08	3.17E-07	1.45E-07	ADIPOQ/TNF/SLC2A4/EDN1/EDNRA/PIK3R1/AKT1/PL/	11
BP	GO:0006606	protein import into nucleus	11/132	161/18870	1.76E-08	3.60E-07	1.64E-07	HSP90AA1/TP53/CDK1/CDKN1A/NFKBIA/STAT3/SYK/I	11
BP	GO:1904705	regulation of vascular associated smooth muscle cell proliferation	9/132	93/18870	1.78E-08	3.63E-07	1.66E-07	ADIPOQ/CDKN1A/TNF/EDN1/GJA1/MMP9/MMP2/SR(	9
BP	GO:1904646	cellular response to amyloid-beta	7/132	43/18870	1.82E-08	3.69E-07	1.69E-07	TNF/APP/GJA1/GSK3B/PARP1/IGF1R/ABCC1	7
BP	GO:1990874	vascular associated smooth muscle cell proliferation	9/132	95/18870	2.15E-08	4.33E-07	1.98E-07	ADIPOQ/CDKN1A/TNF/EDN1/GJA1/MMP9/MMP2/SR(	9
BP	GO:0045931	positive regulation of mitotic cell cycle	10/132	128/18870	2.24E-08	4.49E-07	2.05E-07	CDK1/CCNB1/CCND1/RB1/CDK4/APP/EGFR/AKT1/PTF	10
BP	GO:0042593	glucose homeostasis	13/132	251/18870	2.37E-08	4.72E-07	2.16E-07	BAD/ADIPOQ/KLF7/SLC2A4/STAT3/NOX4/ALOX5/IGF	13
BP	GO:2000379	positive regulation of reactive oxygen species metabolic process	8/132	68/18870	2.41E-08	4.75E-07	2.17E-07	TP53/CDKN1A/XDH/SYK/CYP1B1/F2/AKR1C3/MAPT	8
BP	GO:2001056	positive regulation of cysteine-type endopeptidase activity	10/132	129/18870	2.42E-08	4.75E-07	2.17E-07	BAX/CYCS/BAD/TNF/MYC/XDH/SYK/CTSD/MAPT/F3	10
BP	GO:0051170	import into nucleus	11/132	166/18870	2.42E-08	4.75E-07	2.17E-07	HSP90AA1/TP53/CDK1/CDKN1A/NFKBIA/STAT3/SYK/I	11
BP	GO:0071674	mononuclear cell migration	12/132	207/18870	2.43E-08	4.76E-07	2.17E-07	TNF/IL6R/APP/ITGB3/CCL2/CXCL11/CXCL10/ALOX5/P	12
BP	GO:0033500	carbohydrate homeostasis	13/132	252/18870	2.48E-08	4.83E-07	2.21E-07	BAD/ADIPOQ/KLF7/SLC2A4/STAT3/NOX4/ALOX5/IGF	13
BP	GO:0071621	granulocyte chemotaxis	10/132	130/18870	2.60E-08	5.04E-07	2.30E-07	EDN1/CCL2/CXCL11/CXCL2/CXCL10/SYK/PTK2/CXCR1	10
BP	GO:0050731	positive regulation of peptidyl-tyrosine phosphorylation	11/132	168/18870	2.74E-08	5.28E-07	2.41E-07	TP53/ADIPOQ/TNF/IL6R/ITGB3/NOX4/FLT3/SYK/SRC/I	11
BP	GO:1901992	positive regulation of mitotic cell cycle phase transition	9/132	98/18870	2.83E-08	5.44E-07	2.49E-07	CDK1/CCNB1/CCND1/RB1/CDK4/APP/EGFR/AKT1/TEF	9
BP	GO:0003015	heart process	13/132	255/18870	2.86E-08	5.45E-07	2.49E-07	KCNH2/SCN5A/HSP90AA1/TNF/DRD2/EDN1/EDNRA/	13
BP	GO:2001237	negative regulation of extrinsic apoptotic signaling pathway	9/132	99/18870	3.10E-08	5.89E-07	2.69E-07	AR/RELA/BCL2/TNF/MCL1/GSK3B/SRC/AKT1/TERT	9
BP	GO:1903522	regulation of blood circulation	13/132	258/18870	3.28E-08	6.20E-07	2.83E-07	KCNH2/SCN5A/HSP90AA1/TNF/DRD2/EDN1/EDNRA/	13
BP	GO:0015850	organic hydroxy compound transport	14/132	308/18870	3.45E-08	6.47E-07	2.96E-07	RXRA/MTTP/CYP19A1/ADIPOQ/DRD2/NFKBIA/ITGB3/	14
BP	GO:0046879	hormone secretion	14/132	308/18870	3.45E-08	6.47E-07	2.96E-07	BAD/CYP19A1/ADIPOQ/TNF/KLF7/DRD2/EDN1/GJA1/	14
BP	GO:0051048	negative regulation of secretion	11/132	172/18870	3.49E-08	6.52E-07	2.98E-07	ADIPOQ/TNF/KLF7/DRD2/EDN1/GJA1/ADORA1/PTPN	11
BP	GO:1903706	regulation of hemopoiesis	16/132	417/18870	3.68E-08	6.84E-07	3.13E-07	FOS/BAD/ADIPOQ/TNF/MYC/RB1/NFKBIA/IL2RA/IL4R/	16
BP	GO:0007204	positive regulation of cytosolic calcium ion concentration	11/132	173/18870	3.71E-08	6.86E-07	3.13E-07	BCL2/BAX/EDN1/EDNRA/CAV1/CXCR1/PLA2G1B/GPR	11
BP	GO:0051047	positive regulation of secretion	14/132	310/18870	3.74E-08	6.88E-07	3.15E-07	BAD/CYP19A1/DRD2/IL4R/EDN1/SPP1/ADORA1/SYK/I	14
BP	GO:0071695	anatomical structure maturation	13/132	261/18870	3.76E-08	6.88E-07	3.15E-07	BCL2/CDKN1A/RB1/APP/EDN1/EDNRA/RUNX2/AKR1E	13
BP	GO:0051098	regulation of binding	13/132	262/18870	3.93E-08	7.12E-07	3.25E-07	BAX/ADIPOQ/CDKN1A/RB1/NFKBIA/APP/CAV1/GSK3E	13
BP	GO:0007159	leukocyte cell-cell adhesion	16/132	419/18870	3.94E-08	7.12E-07	3.25E-07	RELA/BAD/TNF/IL2RA/IL4R/SELE/CAV1/CCL2/ALOX5/S	16
BP	GO:1903169	regulation of calcium ion transmembrane transport	11/132	174/18870	3.94E-08	7.12E-07	3.25E-07	BCL2/BAX/DRD2/ITGB3/CAV1/CXCL11/CXCL10/F2/PL/	11
BP	GO:0098781	ncRNA transcription	10/132	136/18870	4.01E-08	7.22E-07	3.30E-07	AR/RELA/FOS/TP53/TNF/MYC/STAT3/EGFR/TERT/ESR	10
BP	GO:0070372	regulation of ERK1 and ERK2 cascade	14/132	313/18870	4.22E-08	7.56E-07	3.46E-07	ADIPOQ/TNF/DRD2/APP/ITGB3/CCL2/NOX4/SYK/EGF	14
BP	GO:0006809	nitric oxide biosynthetic process	8/132	73/18870	4.25E-08	7.58E-07	3.47E-07	HSP90AA1/TNF/EDN1/CAV1/CYP1B1/AKT1/INSR/PTG	8
BP	GO:1903131	mononuclear cell differentiation	17/132	481/18870	4.43E-08	7.86E-07	3.59E-07	BCL2/FOS/BAX/TP53/BAD/MYC/IL6R/IL2RA/IL4R/STAT	17
BP	GO:0009306	protein secretion	15/132	368/18870	4.60E-08	8.14E-07	3.72E-07	BAD/MTTP/TNF/KLF7/DRD2/ALOX5/F2/ADORA2A/PL/	15
BP	GO:0035592	establishment of protein localization to extracellular region	15/132	369/18870	4.77E-08	8.38E-07	3.83E-07	BAD/MTTP/TNF/KLF7/DRD2/ALOX5/F2/ADORA2A/PL/	15
BP	GO:0010389	regulation of G2/M transition of mitotic cell cycle	9/132	104/18870	4.78E-08	8.38E-07	3.83E-07	CDK1/CCNB1/CDK2/CDKN1A/CCND1/CDK4/APP/PLK	9
BP	GO:0023061	signal release	17/132	484/18870	4.84E-08	8.45E-07	3.86E-07	BAD/CYP19A1/ADIPOQ/TNF/KLF7/DRD2/EDN1/GJA1/	17
BP	GO:0035270	endocrine system development	10/132	139/18870	4.94E-08	8.58E-07	3.92E-07	BAD/DRD2/IL6R/EDN1/EDNRA/GSK3B/CYP1B1/AKT1/I	10
BP	GO:0000086	G2/M transition of mitotic cell cycle	10/132	140/18870	5.28E-08	9.15E-07	4.18E-07	CDK1/CCNB1/CDK2/CDKN1A/CCND1/CDK4/APP/PLK	10
BP	GO:0009914	hormone transport	14/132	319/18870	5.34E-08	9.21E-07	4.21E-07	BAD/CYP19A1/ADIPOQ/TNF/KLF7/DRD2/EDN1/GJA1/	14
BP	GO:0030099	myeloid cell differentiation	16/132	430/18870	5.63E-08	9.66E-07	4.42E-07	FOS/ADIPOQ/TNF/MYC/RB1/NFKBIA/APP/STAT3/STA	16
BP	GO:0007596	blood coagulation	12/132	224/18870	5.82E-08	9.94E-07	4.55E-07	EDN1/ITGB3/CAV1/COL3A1/SYK/F2/ADORA2A/SRC/A	12
BP	GO:0001936	regulation of endothelial cell proliferation	11/132	181/18870	5.90E-08	1.00E-06	4.57E-07	TNF/STAT3/ITGB3/STAT1/CAV1/CCL2/XDH/ALOX5/KD	11

BP	GO:0071478	cellular response to radiation	11/132	181/18870	5.90E-08	1.00E-06	4.57E-07	BAX/TP53/TIMP1/CDKN1A/MYC/PARP1/MMP9/MMP2	11
BP	GO:0022409	positive regulation of cell-cell adhesion	14/132	322/18870	6.00E-08	1.01E-06	4.63E-07	RELA/BAD/TNF/IL2RA/IL4R/SELE/CAV1/CCL2/ALOX5/S	14
BP	GO:0043393	regulation of protein binding	10/132	142/18870	6.05E-08	1.02E-06	4.65E-07	BAX/ADIPOQ/CDKN1A/APP/CAV1/GSK3B/MMP9/PLK1	10
BP	GO:0045936	negative regulation of phosphate metabolic process	15/132	376/18870	6.10E-08	1.02E-06	4.67E-07	BAX/TP53/ADIPOQ/CDKN1A/TNF/DRD2/RB1/CAV1/XI	15
BP	GO:0030593	neutrophil chemotaxis	9/132	107/18870	6.13E-08	1.02E-06	4.67E-07	EDN1/CCL2/CXCL11/CXCL2/CXCL10/SYK/CXCR1/PLA2	9
BP	GO:0010332	response to gamma radiation	7/132	51/18870	6.26E-08	1.04E-06	4.75E-07	BCL2/BAX/TP53/CDKN1A/MYC/CXCL10/PARP1	7
BP	GO:0010563	negative regulation of phosphorus metabolic process	15/132	377/18870	6.31E-08	1.04E-06	4.75E-07	BAX/TP53/ADIPOQ/CDKN1A/TNF/DRD2/RB1/CAV1/XI	15
BP	GO:0071692	protein localization to extracellular region	15/132	377/18870	6.31E-08	1.04E-06	4.75E-07	BAD/MTTP/TNF/KLF7/DRD2/ALOX5/F2/ADORA2A/PL/	15
BP	GO:0002066	columnar/cuboidal epithelial cell development	7/132	52/18870	7.19E-08	1.17E-06	5.36E-07	BAD/CDKN1A/GSK3B/SRC/AKT1/TYMS/CDK6	7
BP	GO:1904707	positive regulation of vascular associated smooth muscle cell proliferation	7/132	52/18870	7.19E-08	1.17E-06	5.36E-07	TNF/EDN1/GJA1/MMP9/MMP2/SRC/TERT	7
BP	GO:0046209	nitric oxide metabolic process	8/132	78/18870	7.20E-08	1.17E-06	5.36E-07	HSP90AA1/TNF/EDN1/CAV1/CYP1B1/AKT1/INSR/PTG	8
BP	GO:0045834	positive regulation of lipid metabolic process	10/132	145/18870	7.37E-08	1.19E-06	5.45E-07	ADIPOQ/TNF/FLT3/ADORA1/IGF1R/F2/AKT1/NR1H3/N	10
BP	GO:0050817	coagulation	12/132	229/18870	7.41E-08	1.19E-06	5.45E-07	EDN1/ITGB3/CAV1/COL3A1/SYK/F2/ADORA2A/SRC/A	12
BP	GO:0071241	cellular response to inorganic substance	12/132	229/18870	7.41E-08	1.19E-06	5.45E-07	SCN5A/FOS/CDK2/EDN1/PARP1/MMP9/EGFR/MMP3/	12
BP	GO:0048638	regulation of developmental growth	14/132	328/18870	7.54E-08	1.21E-06	5.52E-07	AR/BCL2/CDK1/CDKN1A/DRD2/APP/STAT3/EDN1/SPI	14
BP	GO:2001057	reactive nitrogen species metabolic process	8/132	79/18870	7.96E-08	1.27E-06	5.81E-07	HSP90AA1/TNF/EDN1/CAV1/CYP1B1/AKT1/INSR/PTG	8
BP	GO:0007599	hemostasis	12/132	231/18870	8.15E-08	1.29E-06	5.91E-07	EDN1/ITGB3/CAV1/COL3A1/SYK/F2/ADORA2A/SRC/A	12
BP	GO:0001894	tissue homeostasis	13/132	279/18870	8.20E-08	1.29E-06	5.91E-07	BCL2/BAX/RB1/XIAP/ITGB3/GJA1/COL3A1/SPP1/NOX	13
BP	GO:0060249	anatomical structure homeostasis	13/132	279/18870	8.20E-08	1.29E-06	5.91E-07	BCL2/BAX/RB1/XIAP/ITGB3/GJA1/COL3A1/SPP1/NOX	13
BP	GO:0015711	organic anion transport	16/132	443/18870	8.47E-08	1.33E-06	6.08E-07	MTTP/TNF/DRD2/SLC2A4/EDN1/GJA1/ABCG2/ADOR/	16
BP	GO:0043523	regulation of neuron apoptotic process	12/132	232/18870	8.54E-08	1.34E-06	6.11E-07	BCL2/BAX/TP53/TNF/CCND1/MCL1/CCL2/PARP1/ADO	12
BP	GO:0002573	myeloid leukocyte differentiation	12/132	235/18870	9.83E-08	1.53E-06	7.01E-07	FOS/ADIPOQ/TNF/MYC/RB1/APP/GLO1/PARP1/MMP	12
BP	GO:0061448	connective tissue development	13/132	285/18870	1.05E-07	1.63E-06	7.44E-07	TIMP1/KLF7/RB1/IL6R/EDN1/ITGB3/COL1A1/COL3A1/	13
BP	GO:0062012	regulation of small molecule metabolic process	14/132	337/18870	1.05E-07	1.63E-06	7.44E-07	TP53/BAD/ADIPOQ/TNF/APP/STAT3/CAV1/PARP1/SR	14
BP	GO:0042752	regulation of circadian rhythm	9/132	114/18870	1.06E-07	1.64E-06	7.49E-07	TP53/CDK1/DRD2/ADORA1/GSK3B/PARP1/ADORA2A/	9
BP	GO:0071466	cellular response to xenobiotic stimulus	11/132	192/18870	1.08E-07	1.66E-06	7.57E-07	KCNH2/TP53/ADIPOQ/MYC/RB1/EDN1/ITGB3/CYP1B1	11
BP	GO:0033559	unsaturated fatty acid metabolic process	9/132	115/18870	1.15E-07	1.75E-06	8.02E-07	EDN1/AKR1B1/ALOX5/CYP1B1/ALOX15/ALOX12/AKR1	9
BP	GO:1903531	negative regulation of secretion by cell	10/132	152/18870	1.15E-07	1.75E-06	8.02E-07	ADIPOQ/KLF7/DRD2/EDN1/GJA1/ADORA1/PTPN11/N	10
BP	GO:0022898	regulation of transmembrane transporter activity	12/132	239/18870	1.18E-07	1.79E-06	8.17E-07	BCL2/DRD2/APP/EDN1/EDNRA/CAV1/CCL2/MMP9/PI	12
BP	GO:0007548	sex differentiation	13/132	288/18870	1.19E-07	1.79E-06	8.17E-07	AR/BCL2/BAX/CYP19A1/MMP2/CYP1B1/SRC/KDR/AXL	13
BP	GO:1903532	positive regulation of secretion by cell	13/132	288/18870	1.19E-07	1.79E-06	8.17E-07	BAD/CYP19A1/DRD2/IL4R/EDN1/SPP1/SYK/F2/ADOR/	13
BP	GO:0043276	anoikis	6/132	34/18870	1.20E-07	1.79E-06	8.19E-07	BCL2/MCL1/CAV1/SRC/PTK2/AKT1	6
BP	GO:0071897	DNA biosynthetic process	11/132	194/18870	1.20E-07	1.79E-06	8.19E-07	HSP90AA1/TP53/ADIPOQ/CDKN1A/TNF/MYC/NOX4/(	11
BP	GO:0001836	release of cytochrome c from mitochondria	7/132	56/18870	1.22E-07	1.82E-06	8.30E-07	BCL2/BAX/TP53/BAD/MCL1/MMP9/AKT1	7
BP	GO:0038083	peptidyl-tyrosine autophosphorylation	5/132	18/18870	1.24E-07	1.83E-06	8.36E-07	CAV1/IGF1R/EGFR/KDR/ALK	5
BP	GO:2000811	negative regulation of anoikis	5/132	18/18870	1.24E-07	1.83E-06	8.36E-07	BCL2/MCL1/CAV1/SRC/PTK2	5
BP	GO:0019216	regulation of lipid metabolic process	14/132	342/18870	1.26E-07	1.86E-06	8.50E-07	ADIPOQ/TNF/CAV1/FLT3/ADORA1/IGF1R/F2/ALK/AKT	14
BP	GO:1901991	negative regulation of mitotic cell cycle phase transition	11/132	196/18870	1.33E-07	1.95E-06	8.90E-07	BCL2/TP53/CDK1/CCNB1/CDK2/CDKN1A/CCND1/RB1	11
BP	GO:2000278	regulation of DNA biosynthetic process	9/132	117/18870	1.33E-07	1.95E-06	8.90E-07	HSP90AA1/TP53/ADIPOQ/CDKN1A/TNF/MYC/NOX4/(	9
BP	GO:0030100	regulation of endocytosis	13/132	291/18870	1.34E-07	1.95E-06	8.92E-07	ADIPOQ/TNF/DRD2/ITGB3/SELE/CAV1/CCL2/SYK/SRC	13
BP	GO:0019748	secondary metabolic process	7/132	57/18870	1.38E-07	2.01E-06	9.17E-07	BCL2/AKR1B1/CYP1B1/AKR1B10/AKR1C3/AKR1A1/TYI	7
BP	GO:0071496	cellular response to external stimulus	14/132	346/18870	1.45E-07	2.11E-06	9.63E-07	RXRA/BCL2/FOS/TP53/BAD/CDKN1A/ITGB3/COL1A1/I	14
BP	GO:0048754	branching morphogenesis of an epithelial tube	10/132	156/18870	1.47E-07	2.11E-06	9.66E-07	AR/BCL2/TNF/MYC/EDN1/EDNRA/SRC/KDR/MET/ESR1	10
BP	GO:0097530	granulocyte migration	10/132	156/18870	1.47E-07	2.11E-06	9.66E-07	EDN1/CCL2/CXCL11/CXCL2/CXCL10/SYK/PTK2/CXCR1	10
BP	GO:0051091	positive regulation of DNA-binding transcription factor activity	12/132	244/18870	1.48E-07	2.12E-06	9.69E-07	AR/RELA/TNF/APP/STAT3/EDN1/CAV1/ALK/AKT1/PLA	12
BP	GO:1901989	positive regulation of cell cycle phase transition	9/132	119/18870	1.54E-07	2.20E-06	1.01E-06	CDK1/CCNB1/CCND1/RB1/CDK4/APP/EGFR/AKT1/TEF	9
BP	GO:0032370	positive regulation of lipid transport	8/132	86/18870	1.55E-07	2.21E-06	1.01E-06	RXRA/CYP19A1/ADIPOQ/NFKBIA/EDN1/CAV1/SPP1/N	8
BP	GO:0019369	arachidonic acid metabolic process	7/132	58/18870	1.56E-07	2.21E-06	1.01E-06	ALOX5/CYP1B1/ALOX15/ALOX12/AKR1C3/PTGS1/PTG	7
BP	GO:0045428	regulation of nitric oxide biosynthetic process	7/132	58/18870	1.56E-07	2.21E-06	1.01E-06	HSP90AA1/TNF/EDN1/CAV1/AKT1/INSR/PTGS2	7

BP	GO:0046660	female sex differentiation	9/132	120/18870	1.66E-07	2.32E-06	1.06E-06	BCL2/BAX/CYP19A1/MMP2/SRC/KDR/AXL/INSR/ESR1	9
BP	GO:2001236	regulation of extrinsic apoptotic signaling pathway	10/132	158/18870	1.66E-07	2.32E-06	1.06E-06	AR/RELA/BCL2/TNF/MCL1/CAV1/GSK3B/SRC/AKT1/TE	10
BP	GO:0048708	astrocyte differentiation	8/132	87/18870	1.70E-07	2.38E-06	1.09E-06	TNF/APP/STAT3/F2/ADORA2A/PTPN11/MAPT/CDK6	8
BP	GO:0045786	negative regulation of cell cycle	15/132	407/18870	1.70E-07	2.38E-06	1.09E-06	BCL2/TP53/CDK1/CCNB1/CDK2/CDKN1A/TNF/CCND1	15
BP	GO:0051054	positive regulation of DNA metabolic process	13/132	298/18870	1.76E-07	2.44E-06	1.12E-06	HSP90AA1/BAX/CDK1/CDK2/TNF/MYC/NOX4/PARP1/	13
BP	GO:0045930	negative regulation of mitotic cell cycle	12/132	248/18870	1.76E-07	2.44E-06	1.12E-06	BCL2/TP53/CDK1/CCNB1/CDK2/CDKN1A/TNF/CCND1	12
BP	GO:0045471	response to ethanol	9/132	121/18870	1.78E-07	2.45E-06	1.12E-06	FOS/CDK1/ADIPOQ/TNF/DRD2/SLC2A4/IGF1R/IL2/TYI	9
BP	GO:0070588	calcium ion transmembrane transport	14/132	352/18870	1.79E-07	2.47E-06	1.13E-06	SCN5A/BCL2/BAX/DRD2/EDN1/EDNRA/ITGB3/CAV1/(	14
BP	GO:0001935	endothelial cell proliferation	11/132	202/18870	1.80E-07	2.47E-06	1.13E-06	TNF/STAT3/ITGB3/STAT1/CAV1/CCL2/XDH/ALOX5/KD	11
BP	GO:0001763	morphogenesis of a branching structure	11/132	203/18870	1.89E-07	2.59E-06	1.18E-06	AR/BCL2/TNF/MYC/DRD2/EDN1/EDNRA/SRC/KDR/ME	11
BP	GO:0022612	gland morphogenesis	9/132	122/18870	1.91E-07	2.60E-06	1.19E-06	AR/BCL2/BAX/TNF/CAV1/MMP2/EGFR/SRC/ESR1	9
BP	GO:0007566	embryo implantation	7/132	60/18870	1.98E-07	2.68E-06	1.23E-06	TIMP1/ITGB3/GJA1/SPP1/MMP9/MMP2/PTGS2	7
BP	GO:0080164	regulation of nitric oxide metabolic process	7/132	60/18870	1.98E-07	2.68E-06	1.23E-06	HSP90AA1/TNF/EDN1/CAV1/AKT1/INSR/PTGS2	7
BP	GO:0033280	response to vitamin D	6/132	37/18870	2.03E-07	2.72E-06	1.25E-06	RXRA/CXCL10/SPP1/PIM1/TYR/PTGS2	6
BP	GO:0046326	positive regulation of glucose import	6/132	37/18870	2.03E-07	2.72E-06	1.25E-06	ADIPOQ/PIK3R1/AKT1/PTPN11/TERT/INSR	6
BP	GO:0051385	response to mineralocorticoid	6/132	37/18870	2.03E-07	2.72E-06	1.25E-06	FOS/CDKN1A/EDN1/PARP1/IGF1R/SRC	6
BP	GO:0120254	olefinic compound metabolic process	10/132	162/18870	2.09E-07	2.79E-06	1.28E-06	CYP19A1/AKR1B1/ALOX5/CYP1B1/AKR1B10/ALOX15/	10
BP	GO:0046456	icosanoid biosynthetic process	7/132	61/18870	2.23E-07	2.96E-06	1.35E-06	EDN1/ALOX5/SYK/PLA2G1B/AKR1C3/PTGS1/PTGS2	7
BP	GO:0048608	reproductive structure development	13/132	305/18870	2.30E-07	3.05E-06	1.39E-06	AR/BCL2/BAX/CYP19A1/MMP2/CYP1B1/SRC/KDR/AXL	13
BP	GO:0001558	regulation of cell growth	15/132	417/18870	2.33E-07	3.08E-06	1.41E-06	BCL2/TP53/CDKN1A/RB1/EDN1/GJA1/SPP1/GSK3B/EG	15
BP	GO:0001952	regulation of cell-matrix adhesion	9/132	125/18870	2.35E-07	3.09E-06	1.41E-06	BCL2/ITGB3/GSK3B/MMP12/PIK3R1/SRC/PTK2/KDR/CI	9
BP	GO:0008286	insulin receptor signaling pathway	9/132	125/18870	2.35E-07	3.09E-06	1.41E-06	RELA/GSK3B/IGF1R/PIK3R1/SRC/AKT1/PTPN11/CTSD/	9
BP	GO:0045429	positive regulation of nitric oxide biosynthetic process	6/132	38/18870	2.40E-07	3.14E-06	1.44E-06	HSP90AA1/TNF/EDN1/AKT1/INSR/PTGS2	6
BP	GO:0008016	regulation of heart contraction	11/132	208/18870	2.42E-07	3.15E-06	1.44E-06	KCNH2/SCN5A/HSP90AA1/TNF/DRD2/EDN1/EDNRA/	11
BP	GO:0071383	cellular response to steroid hormone stimulus	11/132	208/18870	2.42E-07	3.15E-06	1.44E-06	RXRA/AR/EDN1/FLT3/PARP1/CYP1B1/IGF1R/SRC/AKR	11
BP	GO:0045927	positive regulation of growth	12/132	256/18870	2.48E-07	3.22E-06	1.47E-06	BCL2/CDK1/DRD2/EDN1/EGFR/F2/PIM1/CSNK2A1/AK	12
BP	GO:0022617	extracellular matrix disassembly	7/132	62/18870	2.49E-07	3.22E-06	1.47E-06	PRSS1/MMP9/MMP2/MMP12/MMP13/MMP3/MMP1	7
BP	GO:0043086	negative regulation of catalytic activity	16/132	480/18870	2.51E-07	3.23E-06	1.48E-06	TP53/ADIPOQ/TIMP1/CDKN1A/TNF/DRD2/RB1/XIAP/(	16
BP	GO:0043200	response to amino acid	9/132	126/18870	2.52E-07	3.23E-06	1.48E-06	RELA/BCL2/TNF/EDN1/COL1A1/COL3A1/MMP2/IGF1F	9
BP	GO:0051222	positive regulation of protein transport	13/132	308/18870	2.57E-07	3.29E-06	1.50E-06	HSP90AA1/CDK1/BAD/TNF/GSK3B/F2/PIK3R1/ADORA	13
BP	GO:0061458	reproductive system development	13/132	309/18870	2.67E-07	3.40E-06	1.56E-06	AR/BCL2/BAX/CYP19A1/MMP2/CYP1B1/SRC/KDR/AXL	13
BP	GO:0032757	positive regulation of interleukin-8 production	7/132	63/18870	2.79E-07	3.55E-06	1.62E-06	RELA/ADIPOQ/TNF/STAT3/SYK/PLA2G1B/F3	7
BP	GO:1904407	positive regulation of nitric oxide metabolic process	6/132	39/18870	2.82E-07	3.58E-06	1.64E-06	HSP90AA1/TNF/EDN1/AKT1/INSR/PTGS2	6
BP	GO:0097237	cellular response to toxic substance	9/132	128/18870	2.88E-07	3.64E-06	1.66E-06	TNF/ABCG2/AKR1B10/PIM1/MPO/AKR1A1/PTGS1/PT(	9
BP	GO:1990266	neutrophil migration	9/132	129/18870	3.08E-07	3.88E-06	1.77E-06	EDN1/CCL2/CXCL11/CXCL2/CXCL10/SYK/CXCR1/PLA2	9
BP	GO:0032411	positive regulation of transporter activity	8/132	94/18870	3.11E-07	3.90E-06	1.78E-06	RXRA/ADIPOQ/EDN1/EDNRA/CCL2/ABCB1/NR1H3/CI	8
BP	GO:0031100	animal organ regeneration	7/132	64/18870	3.11E-07	3.90E-06	1.78E-06	CDK1/CDKN1A/TNF/CCND1/FLT3/AXL/TYMS	7
BP	GO:0030098	lymphocyte differentiation	15/132	429/18870	3.34E-07	4.18E-06	1.91E-06	BCL2/BAX/TP53/BAD/IL6R/IL2RA/IL4R/STAT3/RUNX2/	15
BP	GO:0048872	homeostasis of number of cells	13/132	316/18870	3.44E-07	4.29E-06	1.96E-06	BCL2/BAX/RB1/IL2RA/XIAP/STAT3/STAT1/FLT3/AKT1/	13
BP	GO:0001959	regulation of cytokine-mediated signaling pathway	10/132	171/18870	3.46E-07	4.29E-06	1.96E-06	ADIPOQ/XIAP/EDN1/CAV1/SYK/MMP12/ARG1/AXL/P	10
BP	GO:0050680	negative regulation of epithelial cell proliferation	10/132	173/18870	3.85E-07	4.77E-06	2.18E-06	AR/TNF/DRD2/RB1/STAT1/CAV1/CCL2/XDH/ALOX5/C	10
BP	GO:0070374	positive regulation of ERK1 and ERK2 cascade	11/132	218/18870	3.87E-07	4.78E-06	2.19E-06	TNF/DRD2/APP/ITGB3/CCL2/NOX4/EGFR/SRC/KDR/AI	11
BP	GO:0030879	mammary gland development	9/132	133/18870	3.99E-07	4.89E-06	2.24E-06	AR/BAX/CYP19A1/CCND1/CAV1/XDH/SRC/AKT1/ESR1	9
BP	GO:0061041	regulation of wound healing	9/132	133/18870	3.99E-07	4.89E-06	2.24E-06	TNF/EDN1/CAV1/CLDN4/ALOX5/F2/PTK2/ALOX12/F3	9
BP	GO:0001667	ameboidal-type cell migration	16/132	497/18870	4.00E-07	4.89E-06	2.24E-06	TIMP1/TNF/EDN1/EDNRA/ITGB3/GJA1/MMP9/CYP1B1	16
BP	GO:0007259	receptor signaling pathway via JAK-STAT	10/132	174/18870	4.06E-07	4.94E-06	2.26E-06	TNF/IL6R/STAT3/STAT1/CAV1/CCL2/FLT3/CYP1B1/F2/	10
BP	GO:0050806	positive regulation of synaptic transmission	10/132	174/18870	4.06E-07	4.94E-06	2.26E-06	TNF/DRD2/APP/CCL2/CA2/ADORA1/GSK3B/ADORA2/	10
BP	GO:1902105	regulation of leukocyte differentiation	13/132	321/18870	4.11E-07	4.99E-06	2.28E-06	FOS/BAD/ADIPOQ/TNF/MYC/RB1/IL2RA/IL4R/SYK/PIK	13
BP	GO:0010810	regulation of cell-substrate adhesion	11/132	220/18870	4.24E-07	5.13E-06	2.34E-06	BCL2/ITGB3/COL1A1/GSK3B/MMP12/PIK3R1/SRC/PTK	11

BP	GO:0046888	negative regulation of hormone secretion	7/132	67/18870	4.28E-07	5.16E-06	2.36E-06	ADIPOQ/KLF7/DRD2/EDN1/GJA1/ADORA1/PTPN11	7
BP	GO:0010634	positive regulation of epithelial cell migration	10/132	176/18870	4.51E-07	5.42E-06	2.48E-06	EDN1/ITGB3/MMP9/SRC/PTK2/KDR/MET/AKT1/PIK3C	10
BP	GO:0002696	positive regulation of leukocyte activation	14/132	380/18870	4.53E-07	5.42E-06	2.48E-06	BCL2/BAD/CDKN1A/TNF/IL2RA/IL4R/CAV1/CCL2/SYK/	14
BP	GO:1904951	positive regulation of establishment of protein localization	13/132	324/18870	4.57E-07	5.46E-06	2.50E-06	HSP90AA1/CDK1/BAD/TNF/GSK3B/F2/PIK3R1/ADORA	13
BP	GO:0030638	polyketide metabolic process	4/132	10/18870	4.65E-07	5.51E-06	2.52E-06	AKR1B1/AKR1B10/AKR1C3/AKR1A1	4
BP	GO:0030647	aminoglycoside antibiotic metabolic process	4/132	10/18870	4.65E-07	5.51E-06	2.52E-06	AKR1B1/AKR1B10/AKR1C3/AKR1A1	4
BP	GO:0044598	doxorubicin metabolic process	4/132	10/18870	4.65E-07	5.51E-06	2.52E-06	AKR1B1/AKR1B10/AKR1C3/AKR1A1	4
BP	GO:0006919	activation of cysteine-type endopeptidase activity involved in apoptotic pr	7/132	68/18870	4.75E-07	5.60E-06	2.56E-06	BAX/CYCS/BAD/TNF/XDH/MAPT/F3	7
BP	GO:0035265	organ growth	10/132	177/18870	4.76E-07	5.60E-06	2.56E-06	AR/BCL2/CDK1/CYP19A1/EDN1/PIM1/MMP13/AKT1/F	10
BP	GO:0008202	steroid metabolic process	13/132	327/18870	5.08E-07	5.96E-06	2.72E-06	CYP19A1/TNF/APP/SULT1E1/SPP1/AKR1B1/CYP1B1/IG	13
BP	GO:0008585	female gonad development	8/132	101/18870	5.42E-07	6.35E-06	2.90E-06	BCL2/BAX/CYP19A1/MMP2/SRC/KDR/INSR/ESR1	8
BP	GO:0033135	regulation of peptidyl-serine phosphorylation	9/132	138/18870	5.46E-07	6.37E-06	2.91E-06	HSP90AA1/BCL2/BAX/TNF/APP/CAV1/EGFR/AKT1/PT	9
BP	GO:0042180	cellular ketone metabolic process	11/132	226/18870	5.54E-07	6.45E-06	2.95E-06	CYP19A1/ADIPOQ/CAV1/AKR1B1/GLO1/AKR1B10/AK	11
BP	GO:0006913	nucleocytoplasmic transport	13/132	330/18870	5.63E-07	6.48E-06	2.96E-06	HSP90AA1/TP53/CDK1/CDKN1A/NFKBIA/STAT3/SYK/	13
BP	GO:0021700	developmental maturation	13/132	330/18870	5.63E-07	6.48E-06	2.96E-06	BCL2/CDKN1A/RB1/APP/EDN1/EDNRA/RUNX2/AKR1E	13
BP	GO:0051169	nuclear transport	13/132	330/18870	5.63E-07	6.48E-06	2.96E-06	HSP90AA1/TP53/CDK1/CDKN1A/NFKBIA/STAT3/SYK/	13
BP	GO:0051251	positive regulation of lymphocyte activation	13/132	330/18870	5.63E-07	6.48E-06	2.96E-06	BCL2/BAD/CDKN1A/IL2RA/IL4R/CAV1/CCL2/SYK/SRC/	13
BP	GO:1901988	negative regulation of cell cycle phase transition	12/132	277/18870	5.77E-07	6.62E-06	3.02E-06	BCL2/TP53/CDK1/CCNB1/CDK2/CDKN1A/CCND1/RB1	12
BP	GO:0043467	regulation of generation of precursor metabolites and energy	9/132	139/18870	5.80E-07	6.64E-06	3.03E-06	TP53/CDK1/CCNB1/TNF/APP/STAT3/GSK3B/AKT1/INS	9
BP	GO:0055074	calcium ion homeostasis	13/132	331/18870	5.82E-07	6.64E-06	3.04E-06	BCL2/BAX/DRD2/APP/EDN1/EDNRA/ITGB3/CAV1/CXC	13
BP	GO:0008630	intrinsic apoptotic signaling pathway in response to DNA damage	8/132	102/18870	5.85E-07	6.66E-06	3.04E-06	BCL2/BAX/TP53/BAD/CDKN1A/TNF/MCL1/PIK3R1	8
BP	GO:0009651	response to salt stress	5/132	24/18870	5.93E-07	6.69E-06	3.06E-06	HSP90AA1/BAX/TP53/TNF/AKR1B1	5
BP	GO:2000209	regulation of anoikis	5/132	24/18870	5.93E-07	6.69E-06	3.06E-06	BCL2/MCL1/CAV1/SRC/PTK2	5
BP	GO:0010828	positive regulation of glucose transmembrane transport	6/132	44/18870	5.93E-07	6.69E-06	3.06E-06	ADIPOQ/PIK3R1/AKT1/PTPN11/TERT/INSR	6
BP	GO:0097306	cellular response to alcohol	8/132	103/18870	6.31E-07	7.10E-06	3.24E-06	FOS/CDK4/CYP1B1/IGF1R/AKT1/AKR1C3/AHR/CFTR	8
BP	GO:0042698	ovulation cycle	7/132	71/18870	6.40E-07	7.17E-06	3.28E-06	CA12/MMP2/CYP1B1/IGF1R/SRC/AXL/ESR1	7
BP	GO:0016042	lipid catabolic process	13/132	334/18870	6.45E-07	7.17E-06	3.28E-06	CYP19A1/ADIPOQ/TNF/SULT1E1/SPP1/ADORA1/CYP1	13
BP	GO:0046394	carboxylic acid biosynthetic process	13/132	334/18870	6.45E-07	7.17E-06	3.28E-06	EDN1/AKR1B1/ALOX5/SYK/ALOX15/ALOX12/PLA2G1E	13
BP	GO:0060759	regulation of response to cytokine stimulus	10/132	183/18870	6.46E-07	7.17E-06	3.28E-06	ADIPOQ/XIAP/EDN1/CAV1/SYK/MMP12/ARG1/AXL/P	10
BP	GO:2000045	regulation of G1/S transition of mitotic cell cycle	10/132	183/18870	6.46E-07	7.17E-06	3.28E-06	BCL2/TP53/CDK2/CDKN1A/CCND1/RB1/CCL2/EGFR/A	10
BP	GO:0001101	response to acid chemical	9/132	141/18870	6.54E-07	7.23E-06	3.30E-06	RELA/BCL2/TNF/EDN1/COL1A1/COL3A1/MMP2/IGF1F	9
BP	GO:1904064	positive regulation of cation transmembrane transport	9/132	141/18870	6.54E-07	7.23E-06	3.30E-06	KCNH2/BAX/EDN1/EDNRA/CAV1/CXCL11/CXCL10/F2	9
BP	GO:0033138	positive regulation of peptidyl-serine phosphorylation	8/132	104/18870	6.79E-07	7.47E-06	3.42E-06	HSP90AA1/BCL2/TNF/APP/CAV1/EGFR/AKT1/PTGS2	8
BP	GO:0030574	collagen catabolic process	6/132	45/18870	6.81E-07	7.47E-06	3.42E-06	MMP9/MMP2/MMP12/MMP13/MMP3/MMP1	6
BP	GO:0006801	superoxide metabolic process	7/132	72/18870	7.05E-07	7.70E-06	3.52E-06	EDN1/NOX4/SYK/MPO/ALOX12/MAPT/DHFR	7
BP	GO:0014823	response to activity	7/132	72/18870	7.05E-07	7.70E-06	3.52E-06	FOS/CDK1/ADIPOQ/TNF/EDN1/ITGB3/MMP2	7
BP	GO:0016053	organic acid biosynthetic process	13/132	337/18870	7.13E-07	7.77E-06	3.55E-06	EDN1/AKR1B1/ALOX5/SYK/ALOX15/ALOX12/PLA2G1E	13
BP	GO:0046545	development of primary female sexual characteristics	8/132	105/18870	7.31E-07	7.94E-06	3.63E-06	BCL2/BAX/CYP19A1/MMP2/SRC/KDR/INSR/ESR1	8
BP	GO:0035994	response to muscle stretch	5/132	25/18870	7.37E-07	7.99E-06	3.65E-06	RELA/FOS/NFKBIA/EDN1/PTK2	5
BP	GO:0050867	positive regulation of cell activation	14/132	396/18870	7.41E-07	8.01E-06	3.66E-06	BCL2/BAD/CDKN1A/TNF/IL2RA/IL4R/CAV1/CCL2/SYK/	14
BP	GO:0008406	gonad development	11/132	233/18870	7.50E-07	8.08E-06	3.69E-06	AR/BCL2/BAX/CYP19A1/MMP2/CYP1B1/SRC/KDR/AKF	11
BP	GO:0032963	collagen metabolic process	8/132	106/18870	7.86E-07	8.45E-06	3.86E-06	COL1A1/MMP9/MMP2/MMP12/F2/MMP13/MMP3/MI	8
BP	GO:0061138	morphogenesis of a branching epithelium	10/132	187/18870	7.88E-07	8.45E-06	3.86E-06	AR/BCL2/TNF/MYC/EDN1/EDNRA/SRC/KDR/MET/ESR1	10
BP	GO:0097696	receptor signaling pathway via STAT	10/132	188/18870	8.27E-07	8.85E-06	4.04E-06	TNF/IL6R/STAT3/STAT1/CAV1/CCL2/FLT3/CYP1B1/F2/	10
BP	GO:0010721	negative regulation of cell development	12/132	287/18870	8.39E-07	8.95E-06	4.09E-06	TP53/ADIPOQ/TNF/MYC/RB1/IL4R/SPP1/GSK3B/F2/PI	12
BP	GO:0002526	acute inflammatory response	8/132	107/18870	8.45E-07	8.99E-06	4.11E-06	CD163/TNF/IL6R/ADORA1/F2/PIK3CG/PTGS2/F3	8
BP	GO:0006631	fatty acid metabolic process	14/132	401/18870	8.60E-07	9.13E-06	4.17E-06	ADIPOQ/EDN1/CAV1/AKR1B1/ALOX5/CYP1B1/ALOX1	14
BP	GO:0055123	digestive system development	9/132	146/18870	8.78E-07	9.29E-06	4.25E-06	BCL2/CDKN1A/TNF/RB1/COL3A1/EGFR/SRC/TYMS/IN	9
BP	GO:0048806	genitalia development	6/132	47/18870	8.87E-07	9.36E-06	4.28E-06	AR/BAX/CYP19A1/AXL/PTPN11/ESR1	6

BP	GO:0045137	development of primary sexual characteristics	11/132	238/18870	9.24E-07	9.73E-06	4.45E-06	AR/BCL2/BAX/CYP19A1/MMP2/CYP1B1/SRC/KDR/AKT	11
BP	GO:0002687	positive regulation of leukocyte migration	9/132	149/18870	1.04E-06	1.09E-05	5.00E-06	TNF/IL6R/APP/EDN1/ITGB3/SELE/CXCL10/PIK3R1/PTK2	9
BP	GO:0008637	apoptotic mitochondrial changes	8/132	110/18870	1.04E-06	1.09E-05	5.00E-06	BCL2/BAX/TP53/BAD/MCL1/GSK3B/MMP9/AKT1	8
BP	GO:0120161	regulation of cold-induced thermogenesis	9/132	150/18870	1.10E-06	1.15E-05	5.26E-06	ADIPOQ/RB1/IL4R/CAV1/GJA1/SYK/IGF1R/ACHE/NR1I	9
BP	GO:0010875	positive regulation of cholesterol efflux	5/132	27/18870	1.11E-06	1.15E-05	5.26E-06	RXRA/ADIPOQ/NFKBIA/CAV1/NR1H3	5
BP	GO:0050995	negative regulation of lipid catabolic process	5/132	27/18870	1.11E-06	1.15E-05	5.26E-06	TNF/ADORA1/ALK/AKT1/PIK3CG	5
BP	GO:0033209	tumor necrosis factor-mediated signaling pathway	8/132	111/18870	1.12E-06	1.16E-05	5.28E-06	RELA/TP53/ADIPOQ/TNF/NFKBIA/XIAP/STAT1/SYK	8
BP	GO:1905954	positive regulation of lipid localization	8/132	111/18870	1.12E-06	1.16E-05	5.28E-06	RXRA/CYP19A1/ADIPOQ/NFKBIA/EDN1/CAV1/SPP1/N	8
BP	GO:0046942	carboxylic acid transport	13/132	351/18870	1.12E-06	1.16E-05	5.30E-06	TNF/DRD2/EDN1/GJA1/ABCG2/ADORA1/SYK/ADORA	13
BP	GO:0060047	heart contraction	11/132	243/18870	1.13E-06	1.17E-05	5.33E-06	KCNH2/SCN5A/HSP90AA1/TNF/DRD2/EDN1/EDNRA/	11
BP	GO:0033628	regulation of cell adhesion mediated by integrin	6/132	49/18870	1.14E-06	1.17E-05	5.36E-06	ITGB3/SYK/CYP1B1/PTK2/PTPN11/PIK3CG	6
BP	GO:0015849	organic acid transport	13/132	352/18870	1.16E-06	1.19E-05	5.43E-06	TNF/DRD2/EDN1/GJA1/ABCG2/ADORA1/SYK/ADORA	13
BP	GO:0106106	cold-induced thermogenesis	9/132	151/18870	1.16E-06	1.19E-05	5.43E-06	ADIPOQ/RB1/IL4R/CAV1/GJA1/SYK/IGF1R/ACHE/NR1I	9
BP	GO:0031589	cell-substrate adhesion	13/132	356/18870	1.32E-06	1.34E-05	6.13E-06	BCL2/ITGB3/COL1A1/COL3A1/GSK3B/MMP12/PIK3R1/	13
BP	GO:0033044	regulation of chromosome organization	11/132	247/18870	1.33E-06	1.35E-05	6.17E-06	CDK1/CCNB1/CDK2/MYC/RB1/BIRC5/PARP1/SRC/PLK	11
BP	GO:0031016	pancreas development	7/132	79/18870	1.33E-06	1.35E-05	6.17E-06	BAD/IL6R/GSK3B/MET/AKT1/INSR/CDK6	7
BP	GO:0007249	canonical NF-kappaB signal transduction	12/132	300/18870	1.34E-06	1.35E-05	6.17E-06	RELA/ADIPOQ/TNF/NFKBIA/XIAP/EDN1/EDNRA/STAT	12
BP	GO:0043254	regulation of protein-containing complex assembly	14/132	419/18870	1.44E-06	1.46E-05	6.66E-06	HSP90AA1/BAX/TP53/TNF/RB1/SYK/GSK3B/SRC/MMP	14
BP	GO:0071248	cellular response to metal ion	10/132	200/18870	1.45E-06	1.46E-05	6.66E-06	SCN5A/FOS/EDN1/PARP1/MMP9/EGFR/ALOX15/AKT1	10
BP	GO:0090087	regulation of peptide transport	10/132	200/18870	1.45E-06	1.46E-05	6.66E-06	BAD/TNF/KLF7/DRD2/CA2/ALOX5/ADORA1/F2/PTPN1	10
BP	GO:0010971	positive regulation of G2/M transition of mitotic cell cycle	5/132	29/18870	1.61E-06	1.61E-05	7.37E-06	CDK1/CCNB1/CCND1/CDK4/APP	5
BP	GO:0010001	glial cell differentiation	11/132	252/18870	1.62E-06	1.61E-05	7.38E-06	RELA/CDK1/TNF/APP/STAT3/F2/ADORA2A/AKT1/PTPI	11
BP	GO:0006636	unsaturated fatty acid biosynthetic process	6/132	52/18870	1.63E-06	1.62E-05	7.43E-06	EDN1/ALOX15/ALOX12/AKR1C3/PTGS1/PTGS2	6
BP	GO:0043154	negative regulation of cysteine-type endopeptidase activity involved in ap	6/132	52/18870	1.63E-06	1.62E-05	7.43E-06	TNF/XIAP/MMP9/SRC/CSNK2A1/AKT1	6
BP	GO:0042176	regulation of protein catabolic process	13/132	364/18870	1.68E-06	1.67E-05	7.64E-06	HSP90AA1/RELA/CDK2/TIMP1/TNF/CAV1/GSK3B/EGFI	13
BP	GO:0098754	detoxification	9/132	158/18870	1.70E-06	1.68E-05	7.66E-06	ABCG2/AKR1B10/PIM1/MPO/ABCB1/AKR1A1/PTGS1/	9
BP	GO:0030217	T cell differentiation	12/132	307/18870	1.70E-06	1.68E-05	7.66E-06	BCL2/TP53/BAD/IL6R/IL2RA/IL4R/STAT3/RUNX2/SYK/	12
BP	GO:0150076	neuroinflammatory response	7/132	82/18870	1.72E-06	1.69E-05	7.73E-06	TNF/APP/MMP9/ADORA2A/MMP3/MAPT/PTGS2	7
BP	GO:0006874	intracellular calcium ion homeostasis	12/132	308/18870	1.76E-06	1.73E-05	7.89E-06	BCL2/BAX/DRD2/APP/EDN1/EDNRA/ITGB3/CAV1/CXC	12
BP	GO:0097553	calcium ion transmembrane import into cytosol	10/132	205/18870	1.82E-06	1.78E-05	8.13E-06	SCN5A/BCL2/BAX/DRD2/ITGB3/CAV1/CXCL11/CXCL1	10
BP	GO:1990748	cellular detoxification	8/132	119/18870	1.89E-06	1.85E-05	8.46E-06	ABCG2/AKR1B10/PIM1/MPO/AKR1A1/PTGS1/PTGS2/	8
BP	GO:0002833	positive regulation of response to biotic stimulus	13/132	369/18870	1.96E-06	1.91E-05	8.73E-06	HSP90AA1/RELA/TNF/NFKBIA/XIAP/CAV1/SYK/MMP1	13
BP	GO:0050805	negative regulation of synaptic transmission	6/132	54/18870	2.05E-06	1.99E-05	9.11E-06	ADIPOQ/DRD2/ADORA1/ACHE/MAPT/PTGS2	6
BP	GO:0016032	viral process	14/132	432/18870	2.07E-06	2.00E-05	9.16E-06	BCL2/TP53/CDK1/TNF/ITGB3/STAT1/CAV1/CCL2/GSK	14
BP	GO:0010631	epithelial cell migration	13/132	372/18870	2.14E-06	2.07E-05	9.48E-06	TNF/EDN1/ITGB3/MMP9/CYP1B1/SRC/PTK2/KDR/MET	13
BP	GO:1902806	regulation of cell cycle G1/S phase transition	10/132	209/18870	2.16E-06	2.09E-05	9.54E-06	BCL2/TP53/CDK2/CDKN1A/CCND1/RB1/CCL2/EGFR/A	10
BP	GO:0010745	negative regulation of macrophage derived foam cell differentiation	4/132	14/18870	2.17E-06	2.09E-05	9.55E-06	ADIPOQ/NFKBIA/ITGB3/NR1H3	4
BP	GO:0030336	negative regulation of cell migration	13/132	373/18870	2.21E-06	2.12E-05	9.69E-06	BCL2/CYP19A1/ADIPOQ/TIMP1/TNF/DRD2/STAT3/GJ	13
BP	GO:0090132	epithelium migration	13/132	375/18870	2.34E-06	2.24E-05	1.03E-05	TNF/EDN1/ITGB3/MMP9/CYP1B1/SRC/PTK2/KDR/MET	13
BP	GO:0015833	peptide transport	11/132	262/18870	2.36E-06	2.25E-05	1.03E-05	BAD/TNF/KLF7/DRD2/EDN1/CA2/ALOX5/ADORA1/F2/	11
BP	GO:0031400	negative regulation of protein modification process	14/132	437/18870	2.36E-06	2.25E-05	1.03E-05	RELA/BAX/ADIPOQ/CDKN1A/TNF/DRD2/RB1/CAV1/X	14
BP	GO:0050890	cognition	12/132	317/18870	2.37E-06	2.25E-05	1.03E-05	FOS/TNF/DRD2/APP/SLC2A4/ADORA1/EGFR/SRC/HM	12
BP	GO:2000134	negative regulation of G1/S transition of mitotic cell cycle	7/132	86/18870	2.37E-06	2.25E-05	1.03E-05	BCL2/TP53/CDK2/CDKN1A/CCND1/RB1/CCL2	7
BP	GO:0002065	columnar/cuboidal epithelial cell differentiation	8/132	123/18870	2.43E-06	2.30E-05	1.05E-05	BAD/CDKN1A/CAV1/GSK3B/SRC/AKT1/TYMS/CDK6	8
BP	GO:0001541	ovarian follicle development	6/132	56/18870	2.55E-06	2.40E-05	1.10E-05	BCL2/BAX/MMP2/SRC/KDR/ESR1	6
BP	GO:0070542	response to fatty acid	6/132	56/18870	2.55E-06	2.40E-05	1.10E-05	ADIPOQ/CDK4/EDN1/SRC/AKR1C3/PTGS2	6
BP	GO:0045637	regulation of myeloid cell differentiation	10/132	213/18870	2.56E-06	2.41E-05	1.10E-05	FOS/ADIPOQ/TNF/MYC/RB1/NFKBIA/STAT3/STAT1/PI	10
BP	GO:0010948	negative regulation of cell cycle process	12/132	320/18870	2.61E-06	2.45E-05	1.12E-05	BCL2/TP53/CDK1/CCNB1/CDK2/CDKN1A/CCND1/RB1	12
BP	GO:1902751	positive regulation of cell cycle G2/M phase transition	5/132	32/18870	2.69E-06	2.52E-05	1.15E-05	CDK1/CCNB1/CCND1/CDK4/APP	5

BP	GO:0090130	tissue migration	13/132	380/18870	2.71E-06	2.53E-05	1.16E-05	TNF/EDN1/ITGB3/MMP9/CYP1B1/SRC/PTK2/KDR/MET	13
BP	GO:0034976	response to endoplasmic reticulum stress	11/132	266/18870	2.73E-06	2.55E-05	1.16E-05	BCL2/BAX/TP53/CCND1/CAV1/ALOX5/GSK3B/PIK3R1/	11
BP	GO:0043406	positive regulation of MAP kinase activity	7/132	88/18870	2.77E-06	2.58E-05	1.18E-05	TNF/EDN1/FLT3/EGFR/PLA2G1B/INSR/PIK3CG	7
BP	GO:0006633	fatty acid biosynthetic process	9/132	168/18870	2.82E-06	2.61E-05	1.19E-05	EDN1/ALOX5/ALOX15/ALOX12/PLA2G1B/AKR1C3/NR	9
BP	GO:1990845	adaptive thermogenesis	9/132	168/18870	2.82E-06	2.61E-05	1.19E-05	ADIPOQ/RB1/IL4R/CAV1/GJA1/SYK/IGF1R/ACHE/NR1I	9
BP	GO:0010720	positive regulation of cell development	14/132	444/18870	2.84E-06	2.63E-05	1.20E-05	RELA/BCL2/FOS/BAD/ADIPOQ/TNF/DRD2/RB1/IL2RA/	14
BP	GO:0045187	regulation of circadian sleep/wake cycle, sleep	4/132	15/18870	2.94E-06	2.71E-05	1.24E-05	DRD2/ADORA1/PARP1/ADORA2A	4
BP	GO:1903828	negative regulation of protein localization	10/132	218/18870	3.15E-06	2.90E-05	1.33E-05	ADIPOQ/KLF7/DRD2/GSK3B/AKT1/PTPN11/NR1H3/HN	10
BP	GO:0009266	response to temperature stimulus	9/132	171/18870	3.26E-06	2.99E-05	1.37E-05	HSP90AA1/CDKN1A/NFKBIA/CXCL10/ADORA1/GSK3E	9
BP	GO:0031623	receptor internalization	8/132	128/18870	3.28E-06	3.00E-05	1.37E-05	DRD2/ITGB3/SELE/CAV1/SYK/CXCR1/ACHE/INSR	8
BP	GO:2000146	negative regulation of cell motility	13/132	388/18870	3.40E-06	3.11E-05	1.42E-05	BCL2/CYP19A1/ADIPOQ/TIMP1/TNF/DRD2/STAT3/GJ/	13
BP	GO:0070098	chemokine-mediated signaling pathway	7/132	91/18870	3.47E-06	3.15E-05	1.44E-05	EDN1/CCL2/CXCL11/CXCL2/CXCL10/CXCR1/GPR35	7
BP	GO:0046686	response to cadmium ion	6/132	59/18870	3.48E-06	3.15E-05	1.44E-05	FOS/CDK1/MMP9/EGFR/AKT1/TERT	6
BP	GO:0060135	maternal process involved in female pregnancy	6/132	59/18870	3.48E-06	3.15E-05	1.44E-05	AR/SPP1/PARP1/AKT1/ESR1/PTGS2	6
BP	GO:0006691	leukotriene metabolic process	5/132	34/18870	3.67E-06	3.31E-05	1.51E-05	ALOX5/SYK/ABCC1/ALOX12/PLA2G1B	5
BP	GO:0070293	renal absorption	5/132	34/18870	3.67E-06	3.31E-05	1.51E-05	ADIPOQ/EDN1/EDNRA/CLDN4/AKR1C3	5
BP	GO:1904385	cellular response to angiotensin	5/132	34/18870	3.67E-06	3.31E-05	1.51E-05	RELA/CAV1/CA2/IGF1R/SRC	5
BP	GO:0060193	positive regulation of lipase activity	6/132	60/18870	3.84E-06	3.45E-05	1.58E-05	EDNRA/SELE/EGFR/PLA2G1B/NR1H3/ESR1	6
BP	GO:0002218	activation of innate immune response	11/132	276/18870	3.89E-06	3.49E-05	1.60E-05	HSP90AA1/RELA/TNF/NFKBIA/XIAP/CAV1/SYK/PIK3R1	11
BP	GO:0050802	circadian sleep/wake cycle, sleep	4/132	16/18870	3.90E-06	3.49E-05	1.60E-05	DRD2/ADORA1/PARP1/ADORA2A	4
BP	GO:0072594	establishment of protein localization to organelle	14/132	459/18870	4.17E-06	3.73E-05	1.70E-05	HSP90AA1/BAX/TP53/CDK1/CDKN1A/NFKBIA/STAT3/	14
BP	GO:0051348	negative regulation of transferase activity	10/132	225/18870	4.18E-06	3.73E-05	1.70E-05	TP53/ADIPOQ/CDKN1A/RB1/CAV1/GSK3B/SRC/PLK1/	10
BP	GO:2000351	regulation of endothelial cell apoptotic process	6/132	61/18870	4.23E-06	3.76E-05	1.72E-05	TNF/ITGB3/CCL2/KDR/AKR1C3/TERT	6
BP	GO:0070633	transepithelial transport	5/132	35/18870	4.26E-06	3.76E-05	1.72E-05	EDN1/ABCG2/ABCC1/ABCB1/CFTR	5
BP	GO:1901099	negative regulation of signal transduction in absence of ligand	5/132	35/18870	4.26E-06	3.76E-05	1.72E-05	BCL2/TNF/MCL1/AKT1/TERT	5
BP	GO:2001240	negative regulation of extrinsic apoptotic signaling pathway in absence of	5/132	35/18870	4.26E-06	3.76E-05	1.72E-05	BCL2/TNF/MCL1/AKT1/TERT	5
BP	GO:0030168	platelet activation	8/132	133/18870	4.36E-06	3.84E-05	1.76E-05	ITGB3/COL3A1/SYK/F2/SRC/ALOX12/AXL/PIK3CG	8
BP	GO:1904950	negative regulation of establishment of protein localization	8/132	133/18870	4.36E-06	3.84E-05	1.76E-05	ADIPOQ/KLF7/DRD2/PTPN11/NR1H3/HMGCR/CYP51/	8
BP	GO:0048565	digestive tract development	8/132	134/18870	4.61E-06	4.05E-05	1.85E-05	BCL2/CDKN1A/TNF/RB1/COL3A1/EGFR/SRC/TYMS	8
BP	GO:0031668	cellular response to extracellular stimulus	11/132	281/18870	4.62E-06	4.05E-05	1.85E-05	RXRA/BCL2/FOS/TP53/CDKN1A/COL1A1/IGF1R/PIM1/	11
BP	GO:1902807	negative regulation of cell cycle G1/S phase transition	7/132	95/18870	4.63E-06	4.05E-05	1.85E-05	BCL2/TP53/CDK2/CDKN1A/CCND1/RB1/CCL2	7
BP	GO:0071715	icosanoid transport	6/132	63/18870	5.12E-06	4.47E-05	2.04E-05	DRD2/EDN1/SYK/ABCC1/PLA2G1B/PTGS2	6
BP	GO:0032412	regulation of monoatomic ion transmembrane transporter activity	10/132	231/18870	5.27E-06	4.59E-05	2.10E-05	DRD2/APP/EDN1/EDNRA/CAV1/CCL2/MMP9/ABCB1/	10
BP	GO:0045089	positive regulation of innate immune response	12/132	343/18870	5.32E-06	4.61E-05	2.11E-05	HSP90AA1/RELA/TNF/NFKBIA/XIAP/CAV1/SYK/MMP1	12
BP	GO:1903035	negative regulation of response to wounding	7/132	97/18870	5.32E-06	4.61E-05	2.11E-05	TNF/STAT3/EDN1/SPP1/ALOX5/F2/ALOX12	7
BP	GO:1904036	negative regulation of epithelial cell apoptotic process	6/132	64/18870	5.62E-06	4.85E-05	2.22E-05	BCL2/RB1/ITGB3/IGF1R/KDR/TERT	6
BP	GO:2001244	positive regulation of intrinsic apoptotic signaling pathway	6/132	64/18870	5.62E-06	4.85E-05	2.22E-05	BAX/TP53/BAD/MYC/MCL1/CAV1	6
BP	GO:0071560	cellular response to transforming growth factor beta stimulus	11/132	287/18870	5.65E-06	4.87E-05	2.23E-05	FOS/TP53/STAT3/EDN1/CAV1/COL1A1/COL3A1/PARF	11
BP	GO:1990868	response to chemokine	7/132	99/18870	6.09E-06	5.23E-05	2.39E-05	EDN1/CCL2/CXCL11/CXCL2/CXCL10/CXCR1/GPR35	7
BP	GO:1990869	cellular response to chemokine	7/132	99/18870	6.09E-06	5.23E-05	2.39E-05	EDN1/CCL2/CXCL11/CXCL2/CXCL10/CXCR1/GPR35	7
BP	GO:0045861	negative regulation of proteolysis	10/132	235/18870	6.14E-06	5.25E-05	2.40E-05	TP53/TIMP1/TNF/XIAP/MMP9/F2/SRC/CSNK2A1/AKT1	10
BP	GO:2000117	negative regulation of cysteine-type endopeptidase activity	6/132	65/18870	6.16E-06	5.26E-05	2.40E-05	TNF/XIAP/MMP9/SRC/CSNK2A1/AKT1	6
BP	GO:1902107	positive regulation of leukocyte differentiation	9/132	185/18870	6.20E-06	5.28E-05	2.41E-05	FOS/BAD/TNF/RB1/IL2RA/IL4R/SYK/AXL/IL2	9
BP	GO:1903708	positive regulation of hemopoiesis	9/132	185/18870	6.20E-06	5.28E-05	2.41E-05	FOS/BAD/TNF/RB1/IL2RA/IL4R/SYK/AXL/IL2	9
BP	GO:0070555	response to interleukin-1	8/132	140/18870	6.38E-06	5.42E-05	2.48E-05	RELA/NFKBIA/APP/EDN1/SELE/CCL2/MMP2/SRC	8
BP	GO:0032373	positive regulation of sterol transport	5/132	38/18870	6.47E-06	5.44E-05	2.49E-05	RXRA/ADIPOQ/NFKBIA/CAV1/NR1H3	5
BP	GO:0032376	positive regulation of cholesterol transport	5/132	38/18870	6.47E-06	5.44E-05	2.49E-05	RXRA/ADIPOQ/NFKBIA/CAV1/NR1H3	5
BP	GO:0071392	cellular response to estradiol stimulus	5/132	38/18870	6.47E-06	5.44E-05	2.49E-05	MMP2/IGF1R/EGFR/ESR1/ESR2	5
BP	GO:0010522	regulation of calcium ion transport into cytosol	4/132	18/18870	6.49E-06	5.44E-05	2.49E-05	BCL2/BAX/CAV1/PLA2G1B	4

BP	GO:0045780	positive regulation of bone resorption	4/132	18/18870	6.49E-06	5.44E-05	2.49E-05	ITGB3/SPP1/SYK/SRC	4
BP	GO:0086103	G protein-coupled receptor signaling pathway involved in heart process	4/132	18/18870	6.49E-06	5.44E-05	2.49E-05	EDN1/EDNRA/CAV1/SRC	4
BP	GO:0070661	leukocyte proliferation	12/132	350/18870	6.53E-06	5.46E-05	2.50E-05	BCL2/BAX/TP53/CDKN1A/IL2RA/FLT3/SYK/ARG1/PTK2	12
BP	GO:0006936	muscle contraction	12/132	351/18870	6.72E-06	5.58E-05	2.55E-05	KCNH2/SCN5A/HSP90AA1/TNF/DRD2/EDN1/EDNRA/	12
BP	GO:0046887	positive regulation of hormone secretion	8/132	141/18870	6.73E-06	5.58E-05	2.55E-05	BAD/CYP19A1/DRD2/EDN1/SPP1/F2/PTPN11/CFTR	8
BP	GO:0034605	cellular response to heat	6/132	66/18870	6.73E-06	5.58E-05	2.55E-05	HSP90AA1/CDKN1A/CXCL10/GSK3B/MAPT/PTGS2	6
BP	GO:0061180	mammary gland epithelium development	6/132	66/18870	6.73E-06	5.58E-05	2.55E-05	AR/BAX/CCND1/SRC/AKT1/ESR1	6
BP	GO:1905953	negative regulation of lipid localization	6/132	66/18870	6.73E-06	5.58E-05	2.55E-05	TNF/NFKBIA/ITGB3/AKT1/PTPN11/NR1H3	6
BP	GO:0008217	regulation of blood pressure	9/132	187/18870	6.77E-06	5.60E-05	2.56E-05	AR/ADIPOQ/TNF/DRD2/EDN1/EDNRA/ADORA1/PTGS	9
BP	GO:0071559	response to transforming growth factor beta	11/132	293/18870	6.87E-06	5.67E-05	2.59E-05	FOS/TP53/STAT3/EDN1/CAV1/COL1A1/COL3A1/PARF	11
BP	GO:0032755	positive regulation of interleukin-6 production	7/132	101/18870	6.96E-06	5.72E-05	2.61E-05	RELA/TNF/IL6R/APP/STAT3/SYK/PTPN11	7
BP	GO:0120162	positive regulation of cold-induced thermogenesis	7/132	101/18870	6.96E-06	5.72E-05	2.61E-05	ADIPOQ/IL4R/CAV1/GJA1/SYK/IGF1R/ACHE	7
BP	GO:0006109	regulation of carbohydrate metabolic process	9/132	188/18870	7.07E-06	5.79E-05	2.65E-05	TP53/BAD/ADIPOQ/APP/STAT3/GSK3B/SRC/AKT1/INS	9
BP	GO:0007093	mitotic cell cycle checkpoint signaling	8/132	142/18870	7.09E-06	5.79E-05	2.65E-05	TP53/CDK1/CCNB1/CDK2/CDKN1A/CCND1/BIRC5/PLI	8
BP	GO:0043405	regulation of MAP kinase activity	8/132	142/18870	7.09E-06	5.79E-05	2.65E-05	ADIPOQ/TNF/EDN1/FLT3/EGFR/PLA2G1B/INSR/PIK3C	8
BP	GO:0007160	cell-matrix adhesion	10/132	239/18870	7.12E-06	5.80E-05	2.65E-05	BCL2/ITGB3/COL3A1/GSK3B/MMP12/PIK3R1/SRC/PTK	10
BP	GO:0010632	regulation of epithelial cell migration	11/132	295/18870	7.33E-06	5.96E-05	2.72E-05	TNF/EDN1/ITGB3/MMP9/SRC/PTK2/KDR/MET/AKT1/P	11
BP	GO:0070301	cellular response to hydrogen peroxide	6/132	67/18870	7.35E-06	5.96E-05	2.72E-05	RELA/CDK1/EDN1/CYP1B1/SRC/AXL	6
BP	GO:0072577	endothelial cell apoptotic process	6/132	67/18870	7.35E-06	5.96E-05	2.72E-05	TNF/ITGB3/CCL2/KDR/AKR1C3/TERT	6
BP	GO:0043550	regulation of lipid kinase activity	5/132	39/18870	7.38E-06	5.96E-05	2.73E-05	RB1/FLT3/F2/PIK3R1/MAPT	5
BP	GO:0097242	amyloid-beta clearance	5/132	39/18870	7.38E-06	5.96E-05	2.73E-05	TNF/IGF1R/HMGCR/CYP51A1/INSR	5
BP	GO:0002027	regulation of heart rate	7/132	102/18870	7.43E-06	5.98E-05	2.74E-05	KCNH2/SCN5A/TNF/DRD2/EDN1/EDNRA/CAV1	7
BP	GO:0042445	hormone metabolic process	10/132	241/18870	7.66E-06	6.16E-05	2.82E-05	CYP19A1/SULT1E1/SPP1/AKR1B1/CYP1B1/AKR1B10/I(	10
BP	GO:0034763	negative regulation of transmembrane transport	8/132	144/18870	7.85E-06	6.30E-05	2.88E-05	KCNH2/BCL2/TNF/DRD2/CAV1/MMP9/AKT1/GPR35	8
BP	GO:0009408	response to heat	7/132	103/18870	7.92E-06	6.33E-05	2.90E-05	HSP90AA1/CDKN1A/CXCL10/GSK3B/AKT1/MAPT/PTG	7
BP	GO:0032677	regulation of interleukin-8 production	7/132	103/18870	7.92E-06	6.33E-05	2.90E-05	RELA/ADIPOQ/TNF/STAT3/SYK/PLA2G1B/F3	7
BP	GO:0022410	circadian sleep/wake cycle process	4/132	19/18870	8.17E-06	6.50E-05	2.97E-05	DRD2/ADORA1/PARP1/ADORA2A	4
BP	GO:0030540	female genitalia development	4/132	19/18870	8.17E-06	6.50E-05	2.97E-05	BAX/CYP19A1/AXL/ESR1	4
BP	GO:0042749	regulation of circadian sleep/wake cycle	4/132	19/18870	8.17E-06	6.50E-05	2.97E-05	DRD2/ADORA1/PARP1/ADORA2A	4
BP	GO:2001235	positive regulation of apoptotic signaling pathway	8/132	145/18870	8.26E-06	6.56E-05	3.00E-05	BAX/TP53/BAD/TNF/MYC/MCL1/CAV1/ADORA2A	8
BP	GO:0000075	cell cycle checkpoint signaling	9/132	192/18870	8.38E-06	6.62E-05	3.03E-05	TP53/CDK1/CCNB1/CDK2/CDKN1A/CCND1/BIRC5/PLI	9
BP	GO:0006730	one-carbon metabolic process	5/132	40/18870	8.39E-06	6.62E-05	3.03E-05	CA2/CA12/CA9/TYMS/DHFR	5
BP	GO:0010742	macrophage derived foam cell differentiation	5/132	40/18870	8.39E-06	6.62E-05	3.03E-05	ADIPOQ/NFKBIA/ITGB3/STAT1/NR1H3	5
BP	GO:0032637	interleukin-8 production	7/132	104/18870	8.45E-06	6.65E-05	3.04E-05	RELA/ADIPOQ/TNF/STAT3/SYK/PLA2G1B/F3	7
BP	GO:0050804	modulation of chemical synaptic transmission	14/132	489/18870	8.58E-06	6.74E-05	3.08E-05	ADIPOQ/TNF/DRD2/APP/EDN1/CCL2/CA2/ADORA1/(	14
BP	GO:0040013	negative regulation of locomotion	13/132	423/18870	8.64E-06	6.77E-05	3.10E-05	BCL2/CYP19A1/ADIPOQ/TIMP1/TNF/DRD2/STAT3/GJ/	13
BP	GO:0035296	regulation of tube diameter	8/132	146/18870	8.69E-06	6.79E-05	3.10E-05	TNF/EDN1/EDNRA/CAV1/ADORA1/MMP2/ADORA2A/	8
BP	GO:0097746	blood vessel diameter maintenance	8/132	146/18870	8.69E-06	6.79E-05	3.10E-05	TNF/EDN1/EDNRA/CAV1/ADORA1/MMP2/ADORA2A/	8
BP	GO:0043627	response to estrogen	6/132	69/18870	8.73E-06	6.81E-05	3.11E-05	HSP90AA1/AR/CAV1/MMP2/ESR1/ESR2	6
BP	GO:0099177	regulation of trans-synaptic signaling	14/132	490/18870	8.78E-06	6.83E-05	3.12E-05	ADIPOQ/TNF/DRD2/APP/EDN1/CCL2/CA2/ADORA1/(	14
BP	GO:0035150	regulation of tube size	8/132	147/18870	9.14E-06	7.07E-05	3.23E-05	TNF/EDN1/EDNRA/CAV1/ADORA1/MMP2/ADORA2A/	8
BP	GO:0035264	multicellular organism growth	8/132	147/18870	9.14E-06	7.07E-05	3.23E-05	AR/BCL2/TP53/DRD2/APP/STAT3/COL3A1/PTPN11	8
BP	GO:0062013	positive regulation of small molecule metabolic process	8/132	147/18870	9.14E-06	7.07E-05	3.23E-05	ADIPOQ/TNF/APP/SRC/AKT1/NR1H3/INSR/PTGS2	8
BP	GO:0002790	peptide secretion	10/132	246/18870	9.17E-06	7.08E-05	3.24E-05	BAD/TNF/KLF7/DRD2/EDN1/ALOX5/ADORA1/F2/PTPN	10
BP	GO:0042554	superoxide anion generation	5/132	41/18870	9.50E-06	7.31E-05	3.34E-05	EDN1/NOX4/SYK/ALOX12/MAPT	5
BP	GO:0090077	foam cell differentiation	5/132	41/18870	9.50E-06	7.31E-05	3.34E-05	ADIPOQ/NFKBIA/ITGB3/STAT1/NR1H3	5
BP	GO:0038061	non-canonical NF-kappaB signal transduction	8/132	148/18870	9.61E-06	7.38E-05	3.37E-05	RELA/TNF/NFKBIA/APP/EDN1/EGFR/ALK/AKT1	8
BP	GO:0051767	nitric-oxide synthase biosynthetic process	4/132	20/18870	1.02E-05	7.74E-05	3.54E-05	EDN1/STAT1/CCL2/KDR	4
BP	GO:0051769	regulation of nitric-oxide synthase biosynthetic process	4/132	20/18870	1.02E-05	7.74E-05	3.54E-05	EDN1/STAT1/CCL2/KDR	4

BP	GO:0051900	regulation of mitochondrial depolarization	4/132	20/18870	1.02E-05	7.74E-05	3.54E-05	BCL2/PARP1/SRC/KDR	4
BP	GO:0055093	response to hyperoxia	4/132	20/18870	1.02E-05	7.74E-05	3.54E-05	CDKN1A/CAV1/COL1A1/MMP2	4
BP	GO:0046651	lymphocyte proliferation	11/132	307/18870	1.07E-05	8.12E-05	3.71E-05	BCL2/BAX/TP53/CDKN1A/IL2RA/FLT3/SYK/ARG1/IL2/F	11
BP	GO:0048512	circadian behavior	5/132	42/18870	1.07E-05	8.12E-05	3.71E-05	TP53/DRD2/ADORA1/PARP1/ADORA2A	5
BP	GO:0071364	cellular response to epidermal growth factor stimulus	5/132	42/18870	1.07E-05	8.12E-05	3.71E-05	FOS/COL1A1/EGFR/AKT1/PTPN11	5
BP	GO:0002791	regulation of peptide secretion	9/132	198/18870	1.07E-05	8.12E-05	3.71E-05	BAD/TNF/KLF7/DRD2/ALOX5/ADORA1/F2/PTPN11/CF	9
BP	GO:0031669	cellular response to nutrient levels	10/132	251/18870	1.09E-05	8.22E-05	3.76E-05	RXRA/BCL2/FOS/TP53/CDKN1A/COL1A1/IGF1R/PIM1/	10
BP	GO:0050870	positive regulation of T cell activation	10/132	251/18870	1.09E-05	8.22E-05	3.76E-05	BAD/IL2RA/IL4R/CAV1/CCL2/SYK/SRC/AKT1/IL2/PTPN	10
BP	GO:1901617	organic hydroxy compound biosynthetic process	10/132	251/18870	1.09E-05	8.22E-05	3.76E-05	CYP19A1/TNF/ALOX15/ALOX12/AKR1C3/HMGCR/CYF	10
BP	GO:0032481	positive regulation of type I interferon production	6/132	72/18870	1.12E-05	8.39E-05	3.83E-05	HSP90AA1/XIAP/STAT1/SYK/MMP12/PTPN11	6
BP	GO:0032722	positive regulation of chemokine production	6/132	72/18870	1.12E-05	8.39E-05	3.83E-05	ADIPOQ/TNF/IL6R/APP/IL4R/SYK	6
BP	GO:0001776	leukocyte homeostasis	7/132	109/18870	1.15E-05	8.61E-05	3.94E-05	BCL2/BAX/IL2RA/FLT3/AKT1/AXL/IL2	7
BP	GO:0010821	regulation of mitochondrion organization	8/132	152/18870	1.17E-05	8.72E-05	3.99E-05	BAX/TP53/BAD/GSK3B/MMP9/KDR/AKT1/MAPT	8
BP	GO:0014002	astrocyte development	5/132	43/18870	1.21E-05	8.98E-05	4.11E-05	TNF/APP/ADORA2A/MAPT/CDK6	5
BP	GO:0060443	mammary gland morphogenesis	5/132	43/18870	1.21E-05	8.98E-05	4.11E-05	AR/BAX/CAV1/SRC/ESR1	5
BP	GO:0072678	T cell migration	6/132	73/18870	1.21E-05	9.00E-05	4.11E-05	APP/ITGB3/CCL2/CXCL11/CXCL10/PIK3CG	6
BP	GO:0001676	long-chain fatty acid metabolic process	7/132	110/18870	1.22E-05	9.04E-05	4.13E-05	ALOX5/CYP1B1/ALOX15/ALOX12/AKR1C3/PTGS1/PTC	7
BP	GO:0090398	cellular senescence	7/132	110/18870	1.22E-05	9.04E-05	4.13E-05	TP53/CDK2/CDKN1A/IGF1R/NUAK1/TERT/CDK6	7
BP	GO:0030183	B cell differentiation	8/132	153/18870	1.22E-05	9.05E-05	4.14E-05	BCL2/BAX/TP53/BAD/FLT3/SYK/PIK3R1/IL2	8
BP	GO:0016137	glycoside metabolic process	4/132	21/18870	1.25E-05	9.17E-05	4.19E-05	AKR1B1/AKR1B10/AKR1C3/AKR1A1	4
BP	GO:0035809	regulation of urine volume	4/132	21/18870	1.25E-05	9.17E-05	4.19E-05	DRD2/EDN1/AKR1B1/ADORA2A	4
BP	GO:0042745	circadian sleep/wake cycle	4/132	21/18870	1.25E-05	9.17E-05	4.19E-05	DRD2/ADORA1/PARP1/ADORA2A	4
BP	GO:0002758	innate immune response-activating signaling pathway	10/132	255/18870	1.25E-05	9.19E-05	4.20E-05	RELA/TNF/NFKBIA/XIAP/CAV1/SYK/PIK3R1/SRC/NR1H	10
BP	GO:0007229	integrin-mediated signaling pathway	7/132	111/18870	1.30E-05	9.49E-05	4.34E-05	TIMP1/ITGB3/COL3A1/SYK/SRC/PTK2/PTPN11	7
BP	GO:0050709	negative regulation of protein secretion	6/132	74/18870	1.31E-05	9.58E-05	4.38E-05	KLF7/DRD2/PTPN11/NR1H3/HMGCR/CYP51A1	6
BP	GO:0032943	mononuclear cell proliferation	11/132	314/18870	1.32E-05	9.61E-05	4.39E-05	BCL2/BAX/TP53/CDKN1A/IL2RA/FLT3/SYK/ARG1/IL2/F	11
BP	GO:0001649	osteoblast differentiation	10/132	257/18870	1.34E-05	9.77E-05	4.47E-05	TNF/IL6R/COL1A1/SPP1/RUNX2/GSK3B/PTK2/AKT1/A(	10
BP	GO:0007622	rhythmic behavior	5/132	44/18870	1.35E-05	9.84E-05	4.50E-05	TP53/DRD2/ADORA1/PARP1/ADORA2A	5
BP	GO:0022408	negative regulation of cell-cell adhesion	9/132	205/18870	1.42E-05	0.00010266	4.69E-05	ADIPOQ/IL2RA/IL4R/ARG1/ADORA2A/PTK2/ALOX12/	9
BP	GO:0010822	positive regulation of mitochondrion organization	6/132	75/18870	1.42E-05	0.00010266	4.69E-05	BAX/TP53/BAD/GSK3B/MMP9/KDR	6
BP	GO:0043588	skin development	11/132	317/18870	1.44E-05	0.00010406	4.76E-05	RELA/BCL2/CDKN1A/TNF/COL1A1/COL3A1/CLDN4/E(	11
BP	GO:0034284	response to monosaccharide	9/132	206/18870	1.47E-05	0.00010631	4.86E-05	BAD/ADIPOQ/TNF/KLF7/RUNX2/NOX4/IGF1R/PTGS2/	9
BP	GO:0033574	response to testosterone	5/132	45/18870	1.52E-05	0.00010819	4.95E-05	AR/EDN1/SPP1/IGF1R/CA9	5
BP	GO:0090199	regulation of release of cytochrome c from mitochondria	5/132	45/18870	1.52E-05	0.00010819	4.95E-05	BAX/TP53/BAD/MMP9/AKT1	5
BP	GO:0010888	negative regulation of lipid storage	4/132	22/18870	1.52E-05	0.00010819	4.95E-05	TNF/NFKBIA/ITGB3/NR1H3	4
BP	GO:0030431	sleep	4/132	22/18870	1.52E-05	0.00010819	4.95E-05	DRD2/ADORA1/PARP1/ADORA2A	4
BP	GO:0072111	cell proliferation involved in kidney development	4/132	22/18870	1.52E-05	0.00010819	4.95E-05	MYC/IL6R/ITGB3/STAT1	4
BP	GO:0098801	regulation of renal system process	4/132	22/18870	1.52E-05	0.00010819	4.95E-05	ADIPOQ/DRD2/EDN1/ADORA1	4
BP	GO:1902644	tertiary alcohol metabolic process	4/132	22/18870	1.52E-05	0.00010819	4.95E-05	AKR1B1/AKR1B10/AKR1C3/AKR1A1	4
BP	GO:0050863	regulation of T cell activation	12/132	381/18870	1.53E-05	0.00010877	4.97E-05	BAD/IL2RA/IL4R/CAV1/CCL2/SYK/ARG1/ADORA2A/SR	12
BP	GO:0051216	cartilage development	9/132	207/18870	1.53E-05	0.00010877	4.97E-05	TIMP1/RB1/EDN1/COL1A1/COL3A1/RUNX2/MMP13/F	9
BP	GO:0007409	axonogenesis	13/132	448/18870	1.59E-05	0.00011269	5.15E-05	HSP90AA1/BCL2/KLF7/DRD2/APP/EDN1/EDNRA/SPP1	13
BP	GO:0001937	negative regulation of endothelial cell proliferation	6/132	77/18870	1.65E-05	0.00011641	5.32E-05	TNF/STAT1/CAV1/CCL2/XDH/ALOX5	6
BP	GO:0032091	negative regulation of protein binding	6/132	77/18870	1.65E-05	0.00011641	5.32E-05	BAX/ADIPOQ/CDKN1A/CAV1/GSK3B/AKT1	6
BP	GO:0032890	regulation of organic acid transport	6/132	77/18870	1.65E-05	0.00011641	5.32E-05	TNF/EDN1/ADORA1/SYK/ADORA2A/AKT1	6
BP	GO:0031018	endocrine pancreas development	5/132	46/18870	1.69E-05	0.00011804	5.40E-05	BAD/IL6R/GSK3B/AKT1/CDK6	5
BP	GO:0032309	icosanoid secretion	5/132	46/18870	1.69E-05	0.00011804	5.40E-05	DRD2/EDN1/SYK/PLA2G1B/PTGS2	5
BP	GO:0032768	regulation of monooxygenase activity	5/132	46/18870	1.69E-05	0.00011804	5.40E-05	TNF/EGFR/AKT1/TERT/DHFR	5
BP	GO:0070849	response to epidermal growth factor	5/132	46/18870	1.69E-05	0.00011804	5.40E-05	FOS/COL1A1/EGFR/AKT1/PTPN11	5

BP	GO:1900271	regulation of long-term synaptic potentiation	5/132	46/18870	1.69E-05	0.00011804	5.40E-05	DRD2/APP/ADORA1/GSK3B/ADORA2A	5
BP	GO:2001239	regulation of extrinsic apoptotic signaling pathway in absence of ligand	5/132	46/18870	1.69E-05	0.00011804	5.40E-05	BCL2/TNF/MCL1/AKT1/TERT	5
BP	GO:0003012	muscle system process	13/132	452/18870	1.74E-05	0.00012162	5.56E-05	KCNH2/SCN5A/HSP90AA1/TNF/DRD2/EDN1/EDNRA/	13
BP	GO:0060191	regulation of lipase activity	6/132	78/18870	1.78E-05	0.00012368	5.65E-05	EDNRA/SELE/EGFR/PLA2G1B/NR1H3/ESR1	6
BP	GO:0051882	mitochondrial depolarization	4/132	23/18870	1.83E-05	0.00012694	5.80E-05	BCL2/PARP1/SRC/KDR	4
BP	GO:0003254	regulation of membrane depolarization	5/132	47/18870	1.88E-05	0.00012958	5.92E-05	SCN5A/BCL2/PARP1/SRC/KDR	5
BP	GO:0014075	response to amine	5/132	47/18870	1.88E-05	0.00012958	5.92E-05	CDK1/DRD2/EDN1/EDNRA/ADORA2A	5
BP	GO:0043029	T cell homeostasis	5/132	47/18870	1.88E-05	0.00012958	5.92E-05	BCL2/BAX/IL2RA/AKT1/IL2	5
BP	GO:0051353	positive regulation of oxidoreductase activity	5/132	47/18870	1.88E-05	0.00012958	5.92E-05	TNF/EDN1/AKT1/TERT/DHFR	5
BP	GO:0140353	lipid export from cell	5/132	47/18870	1.88E-05	0.00012958	5.92E-05	CYP19A1/EDN1/SPP1/PTPN11/PTGS2	5
BP	GO:0051966	regulation of synaptic transmission, glutamatergic	6/132	79/18870	1.91E-05	0.00013152	6.01E-05	TNF/DRD2/CCL2/ADORA1/ADORA2A/PTGS2	6
BP	GO:0042326	negative regulation of phosphorylation	11/132	327/18870	1.92E-05	0.0001318	6.02E-05	BAX/ADIPOQ/CDKN1A/DRD2/RB1/CAV1/XDH/PLK1/A	11
BP	GO:0007088	regulation of mitotic nuclear division	7/132	118/18870	1.93E-05	0.00013207	6.04E-05	CCNB1/TNF/RB1/BIRC5/EDN1/PLK1/INSR	7
BP	GO:0051928	positive regulation of calcium ion transport	7/132	118/18870	1.93E-05	0.00013207	6.04E-05	BAX/CAV1/CCL2/CXCL11/CXCL10/F2/PLA2G1B	7
BP	GO:0007292	female gamete generation	8/132	163/18870	1.94E-05	0.00013207	6.04E-05	BCL2/EDN1/EDNRA/MMP2/SRC/PLK1/AKT1/TOP2A	8
BP	GO:0030307	positive regulation of cell growth	8/132	163/18870	1.94E-05	0.00013207	6.04E-05	BCL2/EDN1/EGFR/F2/CSNK2A1/AKT1/IL2/MAPT	8
BP	GO:0043524	negative regulation of neuron apoptotic process	8/132	163/18870	1.94E-05	0.00013207	6.04E-05	BCL2/BAX/CCND1/CCL2/ADORA2A/KDR/AXL/TERT	8
BP	GO:0016055	Wnt signaling pathway	13/132	459/18870	2.05E-05	0.00013931	6.37E-05	DRD2/CCND1/APP/XIAP/EDN1/EDNRA/CAV1/COL1A1	13
BP	GO:0006953	acute-phase response	5/132	48/18870	2.09E-05	0.00014145	6.47E-05	CD163/TNF/IL6R/F2/PTGS2	5
BP	GO:0043277	apoptotic cell clearance	5/132	48/18870	2.09E-05	0.00014145	6.47E-05	ITGB3/CCL2/ALOX15/AXL/NR1H3	5
BP	GO:0050796	regulation of insulin secretion	8/132	165/18870	2.12E-05	0.00014327	6.55E-05	BAD/TNF/KLF7/DRD2/ALOX5/F2/PTPN11/CFTR	8
BP	GO:0198738	cell-cell signaling by wnt	13/132	461/18870	2.14E-05	0.00014483	6.62E-05	DRD2/CCND1/APP/XIAP/EDN1/EDNRA/CAV1/COL1A1	13
BP	GO:0071346	cellular response to type II interferon	7/132	120/18870	2.16E-05	0.00014535	6.64E-05	TP53/TNF/EDN1/STAT1/CCL2/ARG1/NR1H3	7
BP	GO:0002070	epithelial cell maturation	4/132	24/18870	2.18E-05	0.00014652	6.70E-05	CDKN1A/AKR1B1/KDR/TYMS	4
BP	GO:0003323	type B pancreatic cell development	4/132	24/18870	2.18E-05	0.00014652	6.70E-05	BAD/GSK3B/AKT1/CDK6	4
BP	GO:0051100	negative regulation of binding	7/132	121/18870	2.28E-05	0.00015237	6.97E-05	BAX/ADIPOQ/CDKN1A/NFKBIA/CAV1/GSK3B/AKT1	7
BP	GO:0071887	leukocyte apoptotic process	7/132	121/18870	2.28E-05	0.00015237	6.97E-05	BCL2/BAX/TP53/IL2RA/AKT1/AXL/IL2	7
BP	GO:0048639	positive regulation of developmental growth	8/132	167/18870	2.31E-05	0.00015409	7.04E-05	BCL2/CDK1/DRD2/EDN1/PIM1/AKT1/MAPT/INSR	8
BP	GO:0010518	positive regulation of phospholipase activity	5/132	49/18870	2.31E-05	0.00015409	7.04E-05	EDNRA/SELE/EGFR/PLA2G1B/ESR1	5
BP	GO:0014911	positive regulation of smooth muscle cell migration	5/132	49/18870	2.31E-05	0.00015409	7.04E-05	BCL2/ITGB3/CYP1B1/SRC/TERT	5
BP	GO:0007611	learning or memory	10/132	274/18870	2.33E-05	0.00015494	7.08E-05	FOS/DRD2/APP/SLC2A4/EGFR/SRC/HMGCR/MAPT/INS	10
BP	GO:0060562	epithelial tube morphogenesis	11/132	334/18870	2.33E-05	0.00015501	7.09E-05	AR/BCL2/TNF/MYC/EDN1/EDNRA/CXCL10/SRC/KDR/N	11
BP	GO:0000079	regulation of cyclin-dependent protein serine/threonine kinase activity	6/132	82/18870	2.37E-05	0.00015654	7.16E-05	CCNB1/CDKN1A/CCND1/EGFR/PLK1/AKT1	6
BP	GO:0051341	regulation of oxidoreductase activity	6/132	82/18870	2.37E-05	0.00015654	7.16E-05	TNF/EDN1/EGFR/AKT1/TERT/DHFR	6
BP	GO:0070227	lymphocyte apoptotic process	6/132	82/18870	2.37E-05	0.00015654	7.16E-05	BCL2/BAX/TP53/IL2RA/AKT1/IL2	6
BP	GO:0044773	mitotic DNA damage checkpoint signaling	6/132	83/18870	2.54E-05	0.0001675	7.66E-05	TP53/CDK1/CDK2/CDKN1A/CCND1/PLK1	6
BP	GO:0006693	prostaglandin metabolic process	5/132	50/18870	2.55E-05	0.00016807	7.68E-05	EDN1/AKR1B1/AKR1C3/PTGS1/PTGS2	5
BP	GO:0071320	cellular response to cAMP	5/132	50/18870	2.55E-05	0.00016807	7.68E-05	ADIPOQ/CYP1B1/PIK3CG/AHR/CFTR	5
BP	GO:0048143	astrocyte activation	4/132	25/18870	2.58E-05	0.00016931	7.74E-05	TNF/APP/ADORA2A/MAPT	4
BP	GO:0090200	positive regulation of release of cytochrome c from mitochondria	4/132	25/18870	2.58E-05	0.00016931	7.74E-05	BAX/TP53/BAD/MMP9	4
BP	GO:0042113	B cell activation	10/132	278/18870	2.64E-05	0.00017255	7.89E-05	BCL2/BAX/TP53/BAD/CDKN1A/FLT3/SYK/PIK3R1/IL2/A	10
BP	GO:0002761	regulation of myeloid leukocyte differentiation	7/132	124/18870	2.67E-05	0.00017397	7.95E-05	FOS/ADIPOQ/TNF/MYC/RB1/PIK3R1/CDK6	7
BP	GO:0072676	lymphocyte migration	7/132	124/18870	2.67E-05	0.00017397	7.95E-05	APP/ITGB3/CCL2/CXCL11/CXCL10/AKT1/PIK3CG	7
BP	GO:1904427	positive regulation of calcium ion transmembrane transport	6/132	84/18870	2.72E-05	0.00017707	8.09E-05	BAX/CAV1/CXCL11/CXCL10/F2/PLA2G1B	6
BP	GO:0030856	regulation of epithelial cell differentiation	8/132	171/18870	2.74E-05	0.00017815	8.14E-05	BAD/TNF/KLF7/CCND1/STAT1/CAV1/XDH/MMP9	8
BP	GO:0050866	negative regulation of cell activation	9/132	223/18870	2.76E-05	0.0001792	8.19E-05	IL2RA/IL4R/ARG1/F2/ADORA2A/ALOX12/AXL/IL2/NR1	9
BP	GO:0002757	immune response-activating signaling pathway	13/132	473/18870	2.80E-05	0.00018149	8.30E-05	RELA/BCL2/BAX/TNF/NFKBIA/XIAP/CAV1/SYK/PIK3R1/	13
BP	GO:0071675	regulation of mononuclear cell migration	7/132	125/18870	2.81E-05	0.00018149	8.30E-05	TNF/APP/ITGB3/CCL2/CXCL10/PTK2/AKT1	7
BP	GO:0072089	stem cell proliferation	7/132	125/18870	2.81E-05	0.00018149	8.30E-05	TP53/GJA1/RUNX2/PIM1/KDR/ABCB1/TERT	7

BP	GO:0006692	prostanoid metabolic process	5/132	51/18870	2.82E-05	0.0001815	8.30E-05	EDN1/AKR1B1/AKR1C3/PTGS1/PTGS2	5
BP	GO:0048013	ephrin receptor signaling pathway	5/132	51/18870	2.82E-05	0.0001815	8.30E-05	MMP9/MMP2/SRC/PTK2/PTPN11	5
BP	GO:0034103	regulation of tissue remodeling	6/132	85/18870	2.91E-05	0.00018676	8.54E-05	TP53/ITGB3/SPP1/SYK/SRC/IL2	6
BP	GO:1904029	regulation of cyclin-dependent protein kinase activity	6/132	85/18870	2.91E-05	0.00018676	8.54E-05	CCNB1/CDKN1A/CCND1/EGFR/PLK1/AKT1	6
BP	GO:0030900	forebrain development	12/132	407/18870	2.93E-05	0.00018777	8.58E-05	SCN5A/BAX/DRD2/APP/COL3A1/GSK3B/IGF1R/EGFR/!	12
BP	GO:0000077	DNA damage checkpoint signaling	7/132	126/18870	2.96E-05	0.00018926	8.65E-05	TP53/CDK1/CDK2/CDKN1A/CCND1/PLK1/PTPN11	7
BP	GO:0060333	type II interferon-mediated signaling pathway	4/132	26/18870	3.04E-05	0.00019372	8.86E-05	TP53/STAT1/ARG1/NR1H3	4
BP	GO:1904996	positive regulation of leukocyte adhesion to vascular endothelial cell	4/132	26/18870	3.04E-05	0.00019372	8.86E-05	RELA/TNF/SELE/ALOX5	4
BP	GO:0072330	monocarboxylic acid biosynthetic process	9/132	226/18870	3.06E-05	0.00019529	8.93E-05	EDN1/ALOX5/ALOX15/ALOX12/PLA2G1B/AKR1C3/NR	9
BP	GO:0031103	axon regeneration	5/132	52/18870	3.10E-05	0.0001972	9.01E-05	BCL2/SPP1/MMP2/IGF1R/DHFR	5
BP	GO:0008625	extrinsic apoptotic signaling pathway via death domain receptors	6/132	86/18870	3.11E-05	0.00019729	9.02E-05	BCL2/BAX/BAD/TNF/GSK3B/PIK3R1	6
BP	GO:0051099	positive regulation of binding	7/132	127/18870	3.11E-05	0.00019729	9.02E-05	RB1/APP/CAV1/GSK3B/MMP9/MET/TERT	7
BP	GO:0043542	endothelial cell migration	10/132	284/18870	3.16E-05	0.0002002	9.15E-05	TNF/EDN1/ITGB3/CYP1B1/PTK2/KDR/MET/AKT1/PIK3(	10
BP	GO:0031214	biomineral tissue development	8/132	175/18870	3.23E-05	0.00020436	9.34E-05	RXRA/COL1A1/SPP1/ALOX5/MMP13/ALOX15/PTGS2/	8
BP	GO:0010951	negative regulation of endopeptidase activity	7/132	128/18870	3.27E-05	0.00020622	9.43E-05	TIMP1/TNF/XIAP/MMP9/SRC/CSNK2A1/AKT1	7
BP	GO:0098773	skin epidermis development	7/132	128/18870	3.27E-05	0.00020622	9.43E-05	RELA/BCL2/TNF/CLDN4/EGFR/ALOX12/MET	7
BP	GO:0044774	mitotic DNA integrity checkpoint signaling	6/132	87/18870	3.32E-05	0.00020892	9.55E-05	TP53/CDK1/CDK2/CDKN1A/CCND1/PLK1	6
BP	GO:0045165	cell fate commitment	10/132	286/18870	3.35E-05	0.00021085	9.64E-05	AR/BCL2/TP53/IL6R/MCL1/STAT3/EDN1/EDNRA/RUN)	10
BP	GO:0051224	negative regulation of protein transport	7/132	129/18870	3.44E-05	0.0002158	9.86E-05	ADIPOQ/KLF7/DRD2/PTPN11/NR1H3/HMGCR/CYP51/	7
BP	GO:0045765	regulation of angiogenesis	11/132	349/18870	3.49E-05	0.00021856	9.99E-05	TNF/STAT3/ITGB3/STAT1/CXCL10/ALOX5/CYP1B1/KD	11
BP	GO:0009743	response to carbohydrate	9/132	230/18870	3.52E-05	0.00021991	0.00010053	BAD/ADIPOQ/TNF/KLF7/RUNX2/NOX4/IGF1R/PTGS2/	9
BP	GO:0033627	cell adhesion mediated by integrin	6/132	88/18870	3.54E-05	0.00022067	0.00010087	ITGB3/SYK/CYP1B1/PTK2/PTPN11/PIK3CG	6
BP	GO:0002092	positive regulation of receptor internalization	4/132	27/18870	3.54E-05	0.00022067	0.00010087	DRD2/SELE/SYK/INSR	4
BP	GO:0036296	response to increased oxygen levels	4/132	27/18870	3.54E-05	0.00022067	0.00010087	CDKN1A/CAV1/COL1A1/MMP2	4
BP	GO:0051235	maintenance of location	11/132	350/18870	3.58E-05	0.00022262	0.00010177	BAX/TNF/DRD2/NFKBIA/ITGB3/CAV1/CXCL11/CXCL1(	11
BP	GO:0010595	positive regulation of endothelial cell migration	7/132	130/18870	3.61E-05	0.00022436	0.00010256	EDN1/ITGB3/KDR/MET/AKT1/PIK3CG/PTGS2	7
BP	GO:0010594	regulation of endothelial cell migration	9/132	231/18870	3.64E-05	0.00022542	0.00010305	TNF/EDN1/ITGB3/PTK2/KDR/MET/AKT1/PIK3CG/PTGS.	9
BP	GO:0010874	regulation of cholesterol efflux	5/132	54/18870	3.73E-05	0.00023046	0.00010535	RXRA/ADIPOQ/NFKBIA/CAV1/NR1H3	5
BP	GO:0030520	intracellular estrogen receptor signaling pathway	5/132	54/18870	3.73E-05	0.00023046	0.00010535	AR/PARP1/SRC/ESR1/ESR2	5
BP	GO:0050920	regulation of chemotaxis	9/132	232/18870	3.76E-05	0.00023197	0.00010604	CYP19A1/IL6R/EDN1/CCL2/CXCL10/PTK2/KDR/MET/F:	9
BP	GO:0001960	negative regulation of cytokine-mediated signaling pathway	6/132	89/18870	3.78E-05	0.00023197	0.00010604	ADIPOQ/XIAP/CAV1/MMP12/ARG1/NR1H3	6
BP	GO:0014910	regulation of smooth muscle cell migration	6/132	89/18870	3.78E-05	0.00023197	0.00010604	BCL2/ADIPOQ/ITGB3/CYP1B1/SRC/TERT	6
BP	GO:0046849	bone remodeling	6/132	89/18870	3.78E-05	0.00023197	0.00010604	ITGB3/GJA1/SPP1/NOX4/SYK/SRC	6
BP	GO:0015718	monocarboxylic acid transport	8/132	179/18870	3.80E-05	0.00023289	0.00010646	TNF/DRD2/EDN1/ABCG2/SYK/AKT1/PLA2G1B/PTGS2	8
BP	GO:0060068	vagina development	3/132	10/18870	3.87E-05	0.00023723	0.00010845	BAX/AXL/ESR1	3
BP	GO:1901342	regulation of vasculature development	11/132	354/18870	3.97E-05	0.00024276	0.00011097	TNF/STAT3/ITGB3/STAT1/CXCL10/ALOX5/CYP1B1/KD	11
BP	GO:0042476	odontogenesis	7/132	132/18870	3.99E-05	0.00024335	0.00011124	SCN5A/BAX/EDN1/COL1A1/RUNX2/SRC/CFTR	7
BP	GO:0007589	body fluid secretion	6/132	90/18870	4.02E-05	0.00024491	0.00011196	CCND1/EDN1/CAV1/XDH/ADORA1/NR1H3	6
BP	GO:0010507	negative regulation of autophagy	6/132	90/18870	4.02E-05	0.00024491	0.00011196	BCL2/TP53/MCL1/STAT3/MET/AKT1	6
BP	GO:0046631	alpha-beta T cell activation	8/132	181/18870	4.11E-05	0.00024922	0.00011392	BCL2/IL6R/IL2RA/IL4R/STAT3/SYK/ADORA2A/IL2	8
BP	GO:0060402	calcium ion transport into cytosol	4/132	28/18870	4.11E-05	0.00024922	0.00011392	BCL2/BAX/CAV1/PLA2G1B	4
BP	GO:0090594	inflammatory response to wounding	4/132	28/18870	4.11E-05	0.00024922	0.00011392	TIMP1/TNF/STAT3/ALOX5	4
BP	GO:0043271	negative regulation of monoatomic ion transport	7/132	133/18870	4.18E-05	0.00025312	0.00011571	KCNH2/BCL2/DRD2/CAV1/MMP9/GPR35/PTGS2	7
BP	GO:0002220	innate immune response activating cell surface receptor signaling pathway	6/132	91/18870	4.28E-05	0.0002584	0.00011812	RELA/NFKBIA/SYK/PIK3R1/SRC/NR1H3	6
BP	GO:0045824	negative regulation of innate immune response	6/132	91/18870	4.28E-05	0.0002584	0.00011812	DRD2/PARP1/MMP12/ARG1/AKT1/NR1H3	6
BP	GO:0002221	pattern recognition receptor signaling pathway	9/132	236/18870	4.30E-05	0.00025888	0.00011834	RELA/TNF/NFKBIA/XIAP/CAV1/PIK3R1/SRC/NR1H3/ES	9
BP	GO:0030857	negative regulation of epithelial cell differentiation	5/132	56/18870	4.45E-05	0.00026797	0.0001225	CCND1/STAT1/CAV1/XDH/MMP9	5
BP	GO:1904063	negative regulation of cation transmembrane transport	6/132	92/18870	4.56E-05	0.00027364	0.00012509	KCNH2/BCL2/DRD2/CAV1/MMP9/GPR35	6
BP	GO:0031570	DNA integrity checkpoint signaling	7/132	135/18870	4.60E-05	0.00027599	0.00012616	TP53/CDK1/CDK2/CDKN1A/CCND1/PLK1/PTPN11	7

BP	GO:0006694	steroid biosynthetic process	8/132	184/18870	4.62E-05	0.00027657	0.00012643	CYP19A1/TNF/AKR1B1/IGF1R/AKR1C3/HMGCR/CYP51	8
BP	GO:0031571	mitotic G1 DNA damage checkpoint signaling	4/132	29/18870	4.74E-05	0.00028291	0.00012933	TP53/CDK2/CDKN1A/CCND1	4
BP	GO:0032770	positive regulation of monooxygenase activity	4/132	29/18870	4.74E-05	0.00028291	0.00012933	TNF/AKT1/TERT/DHFR	4
BP	GO:0044819	mitotic G1/S transition checkpoint signaling	4/132	29/18870	4.74E-05	0.00028291	0.00012933	TP53/CDK2/CDKN1A/CCND1	4
BP	GO:0002764	immune response-regulating signaling pathway	13/132	500/18870	4.96E-05	0.00029546	0.00013506	RELA/BCL2/BAX/TNF/NFKBIA/XIAP/CAV1/SYK/PIK3R1/	13
BP	GO:0010639	negative regulation of organelle organization	11/132	363/18870	4.98E-05	0.00029596	0.00013529	TP53/CCNB1/BIRC5/PARP1/PIK3R1/SRC/PLK1/MET/AK	11
BP	GO:0006941	striated muscle contraction	8/132	186/18870	4.98E-05	0.00029596	0.00013529	KCNH2/SCN5A/HSP90AA1/TNF/CAV1/GJA1/ADORA1/	8
BP	GO:0010466	negative regulation of peptidase activity	7/132	137/18870	5.05E-05	0.0002993	0.00013682	TIMP1/TNF/XIAP/MMP9/SRC/CSNK2A1/AKT1	7
BP	GO:0030072	peptide hormone secretion	9/132	241/18870	5.06E-05	0.0002993	0.00013682	BAD/TNF/KLF7/DRD2/EDN1/ALOX5/F2/PTPN11/CFTR	9
BP	GO:0060761	negative regulation of response to cytokine stimulus	6/132	94/18870	5.14E-05	0.00030356	0.00013877	ADIPOQ/XIAP/CAV1/MMP12/ARG1/NR1H3	6
BP	GO:2001251	negative regulation of chromosome organization	6/132	94/18870	5.14E-05	0.00030356	0.00013877	CCNB1/BIRC5/PARP1/SRC/PLK1/TOP2A	6
BP	GO:0031102	neuron projection regeneration	5/132	58/18870	5.28E-05	0.00030835	0.00014095	BCL2/SPP1/MMP2/IGF1R/DHFR	5
BP	GO:0048008	platelet-derived growth factor receptor signaling pathway	5/132	58/18870	5.28E-05	0.00030835	0.00014095	ADIPOQ/ITGB3/SRC/PTPN11/F3	5
BP	GO:0070231	T cell apoptotic process	5/132	58/18870	5.28E-05	0.00030835	0.00014095	BCL2/BAX/TP53/IL2RA/AKT1	5
BP	GO:0010212	response to ionizing radiation	7/132	138/18870	5.29E-05	0.00030835	0.00014095	BCL2/BAX/TP53/CDKN1A/MYC/CXCL10/PARP1	7
BP	GO:0019372	lipoxygenase pathway	3/132	11/18870	5.30E-05	0.00030835	0.00014095	ALOX5/ALOX15/ALOX12	3
BP	GO:0042447	hormone catabolic process	3/132	11/18870	5.30E-05	0.00030835	0.00014095	CYP19A1/SULT1E1/SPP1	3
BP	GO:0060736	prostate gland growth	3/132	11/18870	5.30E-05	0.00030835	0.00014095	AR/CYP19A1/ESR1	3
BP	GO:0070669	response to interleukin-2	3/132	11/18870	5.30E-05	0.00030835	0.00014095	IL2RA/STAT3/IL2	3
BP	GO:1900222	negative regulation of amyloid-beta clearance	3/132	11/18870	5.30E-05	0.00030835	0.00014095	TNF/HMGCR/CYP51A1	3
BP	GO:2001223	negative regulation of neuron migration	3/132	11/18870	5.30E-05	0.00030835	0.00014095	DRD2/STAT3/COL3A1	3
BP	GO:0060537	muscle tissue development	12/132	433/18870	5.33E-05	0.00030985	0.00014164	SCN5A/BCL2/FOS/CDK1/RB1/EDN1/EDNRA/CAV1/GJ/	12
BP	GO:0003309	type B pancreatic cell differentiation	4/132	30/18870	5.44E-05	0.00031591	0.00014441	BAD/GSK3B/AKT1/CDK6	4
BP	GO:0072091	regulation of stem cell proliferation	6/132	95/18870	5.46E-05	0.00031624	0.00014456	TP53/GJA1/RUNX2/PIM1/KDR/TERT	6
BP	GO:0060048	cardiac muscle contraction	7/132	139/18870	5.54E-05	0.00032061	0.00014656	KCNH2/SCN5A/HSP90AA1/CAV1/GJA1/ADORA1/PIK3	7
BP	GO:0001933	negative regulation of protein phosphorylation	10/132	304/18870	5.61E-05	0.0003241	0.00014816	BAX/ADIPOQ/CDKN1A/DRD2/RB1/CAV1/XDH/PLK1/A	10
BP	GO:0010517	regulation of phospholipase activity	5/132	59/18870	5.74E-05	0.00033123	0.00015141	EDNRA/SELE/EGFR/PLA2G1B/ESR1	5
BP	GO:0014909	smooth muscle cell migration	6/132	96/18870	5.79E-05	0.00033352	0.00015246	BCL2/ADIPOQ/ITGB3/CYP1B1/SRC/TERT	6
BP	GO:0048863	stem cell differentiation	9/132	246/18870	5.92E-05	0.00034086	0.00015582	BCL2/TP53/STAT3/EDN1/EDNRA/RUNX2/GSK3B/CDK(	9
BP	GO:0015918	sterol transport	7/132	141/18870	6.07E-05	0.00034857	0.00015934	RXRA/MTTP/ADIPOQ/NFKBIA/CAV1/NR1H3/CFTR	7
BP	GO:0001975	response to amphetamine	4/132	31/18870	6.22E-05	0.00035581	0.00016265	DRD2/EDN1/EDNRA/ADORA2A	4
BP	GO:0050999	regulation of nitric-oxide synthase activity	4/132	31/18870	6.22E-05	0.00035581	0.00016265	EGFR/AKT1/TERT/DHFR	4
BP	GO:0071384	cellular response to corticosteroid stimulus	5/132	60/18870	6.23E-05	0.00035581	0.00016265	EDN1/FLT3/CYP1B1/IGF1R/AKR1C3	5
BP	GO:0097300	programmed necrotic cell death	5/132	60/18870	6.23E-05	0.00035581	0.00016265	BAX/TP53/TNF/CAV1/PARP1	5
BP	GO:0045444	fat cell differentiation	9/132	248/18870	6.31E-05	0.00035976	0.00016446	ADIPOQ/TNF/CCND1/SLC2A4/SULT1E1/ALOX5/PIM1/	9
BP	GO:0006066	alcohol metabolic process	11/132	373/18870	6.36E-05	0.00036227	0.0001656	APP/SULT1E1/AKR1B1/CYP1B1/AKR1B10/AKR1C3/AKF	11
BP	GO:0009755	hormone-mediated signaling pathway	8/132	193/18870	6.47E-05	0.00036795	0.0001682	RXRA/AR/PARP1/SRC/PTPN11/NR1H3/ESR1/ESR2	8
BP	GO:0019932	second-messenger-mediated signaling	10/132	310/18870	6.60E-05	0.00037514	0.00017149	TNF/EDN1/SELE/SYK/GSK3B/EGFR/KDR/CXCR1/MAPT/	10
BP	GO:0034341	response to type II interferon	7/132	143/18870	6.63E-05	0.00037633	0.00017203	TP53/TNF/EDN1/STAT1/CCL2/ARG1/NR1H3	7
BP	GO:0032092	positive regulation of protein binding	5/132	61/18870	6.75E-05	0.00038062	0.00017399	APP/CAV1/GSK3B/MMP9/TERT	5
BP	GO:0046902	regulation of mitochondrial membrane permeability	5/132	61/18870	6.75E-05	0.00038062	0.00017399	BCL2/BAX/TP53/BAD/GSK3B	5
BP	GO:0048010	vascular endothelial growth factor receptor signaling pathway	5/132	61/18870	6.75E-05	0.00038062	0.00017399	ITGB3/SRC/PTK2/KDR/AXL	5
BP	GO:0050994	regulation of lipid catabolic process	5/132	61/18870	6.75E-05	0.00038062	0.00017399	TNF/ADORA1/ALK/AKT1/PIK3CG	5
BP	GO:0051592	response to calcium ion	7/132	144/18870	6.93E-05	0.00039056	0.00017854	SCN5A/FOS/MTTP/EDN1/CAV1/ALOX15/AKR1C3	7
BP	GO:0090276	regulation of peptide hormone secretion	8/132	195/18870	6.95E-05	0.0003912	0.00017883	BAD/TNF/KLF7/DRD2/ALOX5/F2/PTPN11/CFTR	8
BP	GO:0048548	regulation of pinocytosis	3/132	12/18870	7.03E-05	0.00039425	0.00018022	CAV1/AXL/NR1H3	3
BP	GO:0071394	cellular response to testosterone stimulus	3/132	12/18870	7.03E-05	0.00039425	0.00018022	AR/SPP1/IGF1R	3
BP	GO:0001516	prostaglandin biosynthetic process	4/132	32/18870	7.07E-05	0.00039425	0.00018022	EDN1/AKR1C3/PTGS1/PTGS2	4
BP	GO:0002360	T cell lineage commitment	4/132	32/18870	7.07E-05	0.00039425	0.00018022	BCL2/TP53/IL6R/STAT3	4

BP	GO:0046457	prostanoid biosynthetic process	4/132	32/18870	7.07E-05	0.00039425	0.00018022	EDN1/AKR1C3/PTGS1/PTGS2	4
BP	GO:0060055	angiogenesis involved in wound healing	4/132	32/18870	7.07E-05	0.00039425	0.00018022	TNF/ITGB3/ALOX5/KDR	4
BP	GO:0034329	cell junction assembly	12/132	446/18870	7.07E-05	0.00039425	0.00018022	BCL2/TNF/DRD2/APP/ITGB3/CAV1/GJA1/CLDN4/SRC/	12
BP	GO:0050921	positive regulation of chemotaxis	7/132	145/18870	7.24E-05	0.00040301	0.00018423	IL6R/EDN1/CXCL10/PTK2/KDR/MET/F3	7
BP	GO:0018107	peptidyl-threonine phosphorylation	6/132	100/18870	7.28E-05	0.00040381	0.00018459	CDK1/APP/GSK3B/PLK1/CSNK2A1/AKT1	6
BP	GO:0034766	negative regulation of monoatomic ion transmembrane transport	6/132	100/18870	7.28E-05	0.00040381	0.00018459	KCNH2/BCL2/DRD2/CAV1/MMP9/GPR35	6
BP	GO:0046824	positive regulation of nucleocytoplasmic transport	5/132	62/18870	7.30E-05	0.00040444	0.00018488	HSP90AA1/CDK1/GSK3B/PIK3R1/PTGS2	5
BP	GO:0001822	kidney development	10/132	314/18870	7.35E-05	0.00040658	0.00018586	BCL2/BAX/ADIPOQ/MYC/IL6R/EDNRA/ITGB3/STAT1/A	10
BP	GO:0030177	positive regulation of Wnt signaling pathway	7/132	146/18870	7.56E-05	0.00041689	0.00019057	XIAP/CAV1/COL1A1/EGFR/SRC/CSNK2A1/TERT	7
BP	GO:0045598	regulation of fat cell differentiation	7/132	146/18870	7.56E-05	0.00041689	0.00019057	ADIPOQ/TNF/SULT1E1/ALOX5/PIM1/AKT1/PTGS2	7
BP	GO:0051783	regulation of nuclear division	7/132	146/18870	7.56E-05	0.00041689	0.00019057	CCNB1/TNF/RB1/BIRC5/EDN1/PLK1/INSR	7
BP	GO:0009746	response to hexose	8/132	198/18870	7.74E-05	0.00042595	0.00019471	BAD/ADIPOQ/TNF/KLF7/NOX4/IGF1R/PTGS2/CFTR	8
BP	GO:0002090	regulation of receptor internalization	5/132	63/18870	7.88E-05	0.00043217	0.00019756	DRD2/ITGB3/SELE/SYK/INSR	5
BP	GO:0008631	intrinsic apoptotic signaling pathway in response to oxidative stress	5/132	63/18870	7.88E-05	0.00043217	0.00019756	BCL2/MCL1/PARP1/CYP1B1/AKT1	5
BP	GO:0010573	vascular endothelial growth factor production	5/132	63/18870	7.88E-05	0.00043217	0.00019756	RELA/TNF/IL6R/CYP1B1/PTGS2	5
BP	GO:0010743	regulation of macrophage derived foam cell differentiation	4/132	33/18870	8.00E-05	0.00043767	0.00020007	ADIPOQ/NFKBIA/ITGB3/NR1H3	4
BP	GO:0030073	insulin secretion	8/132	199/18870	8.01E-05	0.00043767	0.00020007	BAD/TNF/KLF7/DRD2/ALOX5/F2/PTPN11/CFTR	8
BP	GO:1902075	cellular response to salt	8/132	199/18870	8.01E-05	0.00043767	0.00020007	SCN5A/FOS/EDN1/COL1A1/ALOX15/AKR1C3/ACHE/P	8
BP	GO:0150115	cell-substrate junction organization	6/132	102/18870	8.13E-05	0.00044315	0.00020258	BCL2/ITGB3/PIK3R1/SRC/PTK2/KDR	6
BP	GO:0008544	epidermis development	11/132	385/18870	8.43E-05	0.00045926	0.00020994	RELA/BCL2/CDKN1A/TNF/KLF7/CLDN4/EGFR/ALOX12	11
BP	GO:0002820	negative regulation of adaptive immune response	5/132	64/18870	8.50E-05	0.00046184	0.00021112	IL4R/ARG1/ALOX15/IL2/AHR	5
BP	GO:0030225	macrophage differentiation	5/132	64/18870	8.50E-05	0.00046184	0.00021112	ADIPOQ/RB1/APP/PARP1/MMP9	5
BP	GO:0006898	receptor-mediated endocytosis	9/132	258/18870	8.54E-05	0.00046251	0.00021143	ADIPOQ/DRD2/ITGB3/SELE/CAV1/SYK/CXCR1/ACHE/I	9
BP	GO:0006959	humoral immune response	9/132	258/18870	8.54E-05	0.00046251	0.00021143	BCL2/TNF/CCL2/CXCL11/CXCL2/CXCL10/ALOX5/F2/PI	9
BP	GO:0010906	regulation of glucose metabolic process	6/132	103/18870	8.58E-05	0.00046417	0.00021219	TP53/BAD/ADIPOQ/SRC/AKT1/INSR	6
BP	GO:0019058	viral life cycle	10/132	321/18870	8.82E-05	0.00047637	0.00021776	BCL2/CDK1/TNF/ITGB3/CAV1/CCL2/EGFR/AXL/TOP2A	10
BP	GO:0002832	negative regulation of response to biotic stimulus	7/132	150/18870	8.96E-05	0.00048246	0.00022055	DRD2/PARP1/MMP12/ARG1/AKT1/NR1H3/AHR	7
BP	GO:0008584	male gonad development	7/132	150/18870	8.96E-05	0.00048246	0.00022055	AR/BCL2/BAX/CYP1B1/AKR1C3/INSR/ESR1	7
BP	GO:0051092	positive regulation of NF-kappaB transcription factor activity	7/132	150/18870	8.96E-05	0.00048246	0.00022055	AR/RELA/TNF/STAT3/CAV1/ALK/PLA2G1B	7
BP	GO:0034405	response to fluid shear stress	4/132	34/18870	9.02E-05	0.00048396	0.00022123	MMP2/SRC/AKT1/PTGS2	4
BP	GO:0071398	cellular response to fatty acid	4/132	34/18870	9.02E-05	0.00048396	0.00022123	CDK4/EDN1/SRC/AKR1C3	4
BP	GO:0044403	biological process involved in symbiotic interaction	10/132	322/18870	9.05E-05	0.00048466	0.00022155	CDK1/ITGB3/CAV1/ARG1/EGFR/F2/SRC/AKT1/AXL/INS	10
BP	GO:0032642	regulation of chemokine production	6/132	104/18870	9.05E-05	0.00048466	0.00022155	ADIPOQ/TNF/IL6R/APP/IL4R/SYK	6
BP	GO:0010524	positive regulation of calcium ion transport into cytosol	3/132	13/18870	9.09E-05	0.00048545	0.00022191	BAX/CAV1/PLA2G1B	3
BP	GO:0035810	positive regulation of urine volume	3/132	13/18870	9.09E-05	0.00048545	0.00022191	DRD2/EDN1/ADORA2A	3
BP	GO:0045453	bone resorption	5/132	65/18870	9.16E-05	0.00048781	0.00022299	ITGB3/SPP1/NOX4/SYK/SRC	5
BP	GO:1905330	regulation of morphogenesis of an epithelium	5/132	65/18870	9.16E-05	0.00048781	0.00022299	AR/TNF/GJA1/CXCL10/ESR1	5
BP	GO:0005996	monosaccharide metabolic process	9/132	261/18870	9.32E-05	0.00049591	0.00022669	TP53/BAD/ADIPOQ/TNF/AKR1B1/SRC/AKT1/AKR1A1/I	9
BP	GO:0046546	development of primary male sexual characteristics	7/132	151/18870	9.35E-05	0.00049646	0.00022695	AR/BCL2/BAX/CYP1B1/AKR1C3/INSR/ESR1	7
BP	GO:0072001	renal system development	10/132	324/18870	9.52E-05	0.00050499	0.00023085	BCL2/BAX/ADIPOQ/MYC/IL6R/EDNRA/ITGB3/STAT1/A	10
BP	GO:0032602	chemokine production	6/132	105/18870	9.55E-05	0.00050581	0.00023122	ADIPOQ/TNF/IL6R/APP/IL4R/SYK	6
BP	GO:0050729	positive regulation of inflammatory response	7/132	152/18870	9.74E-05	0.00051542	0.00023561	TNF/NFKBIA/APP/ABCC1/IL2/PIK3CG/PTGS2	7
BP	GO:0002532	production of molecular mediator involved in inflammatory response	6/132	106/18870	0.00010062	0.00053022	0.00024238	TNF/IL4R/STAT3/ALOX5/SYK/F2	6
BP	GO:0034308	primary alcohol metabolic process	6/132	106/18870	0.00010062	0.00053022	0.00024238	SULT1E1/AKR1B1/CYP1B1/AKR1B10/AKR1C3/AKR1A1	6
BP	GO:0060291	long-term synaptic potentiation	6/132	106/18870	0.00010062	0.00053022	0.00024238	DRD2/APP/ADORA1/GSK3B/ADORA2A/HMGCR	6
BP	GO:0001893	maternal placenta development	4/132	35/18870	0.00010124	0.00053022	0.00024238	SPP1/PARP1/AKT1/PTGS2	4
BP	GO:0002446	neutrophil mediated immunity	4/132	35/18870	0.00010124	0.00053022	0.00024238	SYK/ARG1/F2/PLA2G1B	4
BP	GO:0032228	regulation of synaptic transmission, GABAergic	4/132	35/18870	0.00010124	0.00053022	0.00024238	DRD2/CA2/ADORA1/ADORA2A	4
BP	GO:0048147	negative regulation of fibroblast proliferation	4/132	35/18870	0.00010124	0.00053022	0.00024238	BAX/TP53/MYC/CAV1	4

BP	GO:0048873	homeostasis of number of cells within a tissue	4/132	35/18870	0.00010124	0.00053022	0.00024238	BCL2/BAX/XIAP/PTPN11	4
BP	GO:2000241	regulation of reproductive process	8/132	207/18870	0.00010544	0.00055149	0.0002521	AR/BAX/TIMP1/ITGB3/GJA1/SRC/INSR/ESR1	8
BP	GO:0007173	epidermal growth factor receptor signaling pathway	6/132	107/18870	0.00010599	0.00055226	0.00025245	MMP9/EGFR/SRC/PTK2/AKT1/PTPN11	6
BP	GO:0060079	excitatory postsynaptic potential	6/132	107/18870	0.00010599	0.00055226	0.00025245	DRD2/APP/ADORA1/GSK3B/ADORA2A/AKT1	6
BP	GO:0140888	interferon-mediated signaling pathway	6/132	107/18870	0.00010599	0.00055226	0.00025245	TP53/STAT1/MMP12/ARG1/PTPN11/NR1H3	6
BP	GO:0031330	negative regulation of cellular catabolic process	8/132	208/18870	0.00010902	0.00056731	0.00025933	BCL2/TP53/TIMP1/MCL1/STAT3/MET/AKT1/PIK3CG	8
BP	GO:0045807	positive regulation of endocytosis	7/132	155/18870	0.00011007	0.00057208	0.00026151	TNF/DRD2/SELE/CCL2/SYK/AXL/INSR	7
BP	GO:0002068	glandular epithelial cell development	4/132	36/18870	0.00011328	0.000588	0.00026879	BAD/GSK3B/AKT1/CDK6	4
BP	GO:0030193	regulation of blood coagulation	5/132	68/18870	0.00011367	0.00058927	0.00026937	EDN1/CAV1/F2/ALOX12/F3	5
BP	GO:0072006	nephron development	7/132	156/18870	0.00011458	0.00059324	0.00027119	BCL2/ADIPOQ/MYC/IL6R/EDNRA/ITGB3/STAT1	7
BP	GO:0019722	calcium-mediated signaling	8/132	210/18870	0.00011648	0.00060232	0.00027534	TNF/EDN1/SELE/SYK/GSK3B/KDR/CXCR1/MAPT	8
BP	GO:0046822	regulation of nucleocytoplasmic transport	6/132	109/18870	0.00011742	0.00060639	0.0002772	HSP90AA1/CDK1/GSK3B/PIK3R1/PTPN11/PTGS2	6
BP	GO:0051701	biological process involved in interaction with host	8/132	211/18870	0.00012037	0.00062085	0.00028381	CDK1/ITGB3/CAV1/EGFR/SRC/AKT1/AXL/INSR	8
BP	GO:0032835	glomerulus development	5/132	69/18870	0.00012187	0.00062542	0.0002859	BCL2/ADIPOQ/IL6R/EDNRA/ITGB3	5
BP	GO:1901224	positive regulation of non-canonical NF-kappaB signal transduction	5/132	69/18870	0.00012187	0.00062542	0.0002859	RELA/TNF/APP/EDN1/EGFR	5
BP	GO:2000401	regulation of lymphocyte migration	5/132	69/18870	0.00012187	0.00062542	0.0002859	APP/ITGB3/CCL2/CXCL10/AKT1	5
BP	GO:2000573	positive regulation of DNA biosynthetic process	5/132	69/18870	0.00012187	0.00062542	0.0002859	HSP90AA1/TNF/MYC/NOX4/CYP1B1	5
BP	GO:0002444	myeloid leukocyte mediated immunity	6/132	110/18870	0.00012349	0.00063056	0.00028825	IL4R/SYK/ARG1/F2/PLA2G1B/PIK3CG	6
BP	GO:0014812	muscle cell migration	6/132	110/18870	0.00012349	0.00063056	0.00028825	BCL2/ADIPOQ/ITGB3/CYP1B1/SRC/TERT	6
BP	GO:0018210	peptidyl-threonine modification	6/132	110/18870	0.00012349	0.00063056	0.00028825	CDK1/APP/GSK3B/PLK1/CSNK2A1/AKT1	6
BP	GO:0042116	macrophage activation	6/132	110/18870	0.00012349	0.00063056	0.00028825	TNF/APP/IL4R/SYK/NR1H3/MAPT	6
BP	GO:0010863	positive regulation of phospholipase C activity	4/132	37/18870	0.00012633	0.00064186	0.00029341	EDNRA/SELE/EGFR/ESR1	4
BP	GO:0035883	enteroendocrine cell differentiation	4/132	37/18870	0.00012633	0.00064186	0.00029341	BAD/GSK3B/AKT1/CDK6	4
BP	GO:0071276	cellular response to cadmium ion	4/132	37/18870	0.00012633	0.00064186	0.00029341	FOS/MMP9/EGFR/AKT1	4
BP	GO:2000279	negative regulation of DNA biosynthetic process	4/132	37/18870	0.00012633	0.00064186	0.00029341	TP53/ADIPOQ/CDKN1A/SRC	4
BP	GO:0035249	synaptic transmission, glutamatergic	6/132	111/18870	0.0001298	0.00065787	0.00030073	TNF/DRD2/CCL2/ADORA1/ADORA2A/PTGS2	6
BP	GO:1902106	negative regulation of leukocyte differentiation	6/132	111/18870	0.0001298	0.00065787	0.00030073	ADIPOQ/MYC/IL4R/PIK3R1/IL2/CDK6	6
BP	GO:0006081	cellular aldehyde metabolic process	5/132	70/18870	0.00013051	0.00065983	0.00030163	RELA/GLO1/CYP1B1/AKR1C3/AKR1A1	5
BP	GO:1900046	regulation of hemostasis	5/132	70/18870	0.00013051	0.00065983	0.00030163	EDN1/CAV1/F2/ALOX12/F3	5
BP	GO:0048167	regulation of synaptic plasticity	8/132	214/18870	0.00013268	0.00066998	0.00030627	DRD2/APP/ADORA1/GSK3B/ADORA2A/HMGCR/MAP[illegible]	8
BP	GO:0045833	negative regulation of lipid metabolic process	6/132	112/18870	0.00013637	0.00068775	0.00031439	TNF/ADORA1/ALK/AKT1/AKR1C3/PIK3CG	6
BP	GO:0060348	bone development	8/132	215/18870	0.00013701	0.00069013	0.00031548	TP53/GJA1/COL1A1/COL3A1/RUNX2/SRC/KDR/MMP1	8
BP	GO:0060416	response to growth hormone	4/132	38/18870	0.00014043	0.00070647	0.00032295	STAT3/PIK3R1/PTK2/AKT1	4
BP	GO:0021537	telencephalon development	9/132	276/18870	0.00014229	0.00071426	0.00032651	SCN5A/BAX/DRD2/COL3A1/GSK3B/IGF1R/EGFR/ALK/[illegible]	9
BP	GO:0010763	positive regulation of fibroblast migration	3/132	15/18870	0.00014315	0.00071426	0.00032651	ITGB3/PTK2/AKT1	3
BP	GO:0034310	primary alcohol catabolic process	3/132	15/18870	0.00014315	0.00071426	0.00032651	SULT1E1/AKR1B10/AKR1C3	3
BP	GO:0044849	estrous cycle	3/132	15/18870	0.00014315	0.00071426	0.00032651	CA12/CYP1B1/IGF1R	3
BP	GO:0045737	positive regulation of cyclin-dependent protein serine/threonine kinase ac	3/132	15/18870	0.00014315	0.00071426	0.00032651	CCND1/EGFR/AKT1	3
BP	GO:1901722	regulation of cell proliferation involved in kidney development	3/132	15/18870	0.00014315	0.00071426	0.00032651	MYC/IL6R/ITGB3	3
BP	GO:1901222	regulation of non-canonical NF-kappaB signal transduction	6/132	113/18870	0.0001432	0.00071426	0.00032651	RELA/TNF/NFKBIA/APP/EDN1/EGFR	6
BP	GO:0007265	Ras protein signal transduction	10/132	341/18870	0.00014466	0.00072063	0.00032942	TP53/CDK2/CDKN1A/RB1/COL3A1/RASSF1/SRC/MET/	10
BP	GO:0071260	cellular response to mechanical stimulus	5/132	72/18870	0.0001492	0.00074238	0.00033936	BAD/ITGB3/COL1A1/IGF1R/PTGS2	5
BP	GO:0044409	entry into host	7/132	163/18870	0.00015052	0.00074802	0.00034194	CDK1/ITGB3/CAV1/EGFR/SRC/AXL/INSR	7
BP	GO:0051346	negative regulation of hydrolase activity	8/132	218/18870	0.0001507	0.00074802	0.00034194	TIMP1/TNF/XIAP/GSK3B/MMP9/SRC/CSNK2A1/AKT1	8
BP	GO:0044060	regulation of endocrine process	4/132	39/18870	0.00015563	0.00076969	0.00035185	CYP19A1/GJA1/SPP1/PTPN11	4
BP	GO:1900274	regulation of phospholipase C activity	4/132	39/18870	0.00015563	0.00076969	0.00035185	EDNRA/SELE/EGFR/ESR1	4
BP	GO:1901661	quinone metabolic process	4/132	39/18870	0.00015563	0.00076969	0.00035185	AKR1B1/AKR1B10/AKR1C3/AKR1A1	4
BP	GO:0048259	regulation of receptor-mediated endocytosis	6/132	115/18870	0.00015767	0.00077695	0.00035517	ADIPOQ/DRD2/ITGB3/SELE/SYK/INSR	6
BP	GO:0062014	negative regulation of small molecule metabolic process	6/132	115/18870	0.00015767	0.00077695	0.00035517	TP53/ADIPOQ/STAT3/PARP1/AKT1/AKR1C3	6

BP	GO:0099565	chemical synaptic transmission, postsynaptic	6/132	115/18870	0.00015767	0.00077695	0.00035517	DRD2/APP/ADORA1/GSK3B/ADORA2A/AKT1	6
BP	GO:0002067	glandular epithelial cell differentiation	5/132	73/18870	0.00015928	0.00078206	0.0003575	BAD/CAV1/GSK3B/AKT1/CDK6	5
BP	GO:0033344	cholesterol efflux	5/132	73/18870	0.00015928	0.00078206	0.0003575	RXRA/ADIPOQ/NFKBIA/CAV1/NR1H3	5
BP	GO:0050818	regulation of coagulation	5/132	73/18870	0.00015928	0.00078206	0.0003575	EDN1/CAV1/F2/ALOX12/F3	5
BP	GO:0015908	fatty acid transport	6/132	116/18870	0.00016533	0.00081077	0.00037063	DRD2/EDN1/SYK/AKT1/PLA2G1B/PTGS2	6
BP	GO:0050795	regulation of behavior	5/132	74/18870	0.00016988	0.00083107	0.0003799	DRD2/STAT3/ADORA1/PARP1/ADORA2A	5
BP	GO:1905818	regulation of chromosome separation	5/132	74/18870	0.00016988	0.00083107	0.0003799	CCNB1/RB1/BIRC5/PLK1/CSNK2A1	5
BP	GO:0042307	positive regulation of protein import into nucleus	4/132	40/18870	0.00017199	0.00083942	0.00038372	HSP90AA1/CDK1/PIK3R1/PTGS2	4
BP	GO:0071354	cellular response to interleukin-6	4/132	40/18870	0.00017199	0.00083942	0.00038372	RELA/IL6R/STAT3/SRC	4
BP	GO:0042303	molting cycle	6/132	117/18870	0.00017328	0.00084367	0.00038567	RELA/BCL2/TNF/EGFR/TERT/PTGS2	6
BP	GO:0042633	hair cycle	6/132	117/18870	0.00017328	0.00084367	0.00038567	RELA/BCL2/TNF/EGFR/TERT/PTGS2	6
BP	GO:0032354	response to follicle-stimulating hormone	3/132	16/18870	0.00017529	0.00084841	0.00038783	EDN1/EDNRA/CYP1B1	3
BP	GO:0034349	glial cell apoptotic process	3/132	16/18870	0.00017529	0.00084841	0.00038783	TP53/RB1/CCL2	3
BP	GO:0045579	positive regulation of B cell differentiation	3/132	16/18870	0.00017529	0.00084841	0.00038783	BAD/SYK/IL2	3
BP	GO:0047484	regulation of response to osmotic stress	3/132	16/18870	0.00017529	0.00084841	0.00038783	BAD/ABCB1/PTGS2	3
BP	GO:0051770	positive regulation of nitric-oxide synthase biosynthetic process	3/132	16/18870	0.00017529	0.00084841	0.00038783	STAT1/CCL2/KDR	3
BP	GO:0035924	cellular response to vascular endothelial growth factor stimulus	5/132	75/18870	0.000181	0.00087293	0.00039904	RELA/XDH/FLT3/KDR/AKT1	5
BP	GO:0061045	negative regulation of wound healing	5/132	75/18870	0.000181	0.00087293	0.00039904	TNF/EDN1/ALOX5/F2/ALOX12	5
BP	GO:0090559	regulation of membrane permeability	5/132	75/18870	0.000181	0.00087293	0.00039904	BCL2/BAX/TP53/BAD/GSK3B	5
BP	GO:1903707	negative regulation of hemopoiesis	6/132	118/18870	0.00018152	0.00087444	0.00039973	ADIPOQ/MYC/IL4R/PIK3R1/IL2/CDK6	6
BP	GO:0048246	macrophage chemotaxis	4/132	41/18870	0.00018956	0.00090887	0.00041547	CYP19A1/CCL2/MMP2/PTK2	4
BP	GO:0051281	positive regulation of release of sequestered calcium ion into cytosol	4/132	41/18870	0.00018956	0.00090887	0.00041547	BAX/CXCL11/CXCL10/F2	4
BP	GO:0150077	regulation of neuroinflammatory response	4/132	41/18870	0.00018956	0.00090887	0.00041547	TNF/MMP9/MMP3/PTGS2	4
BP	GO:1904994	regulation of leukocyte adhesion to vascular endothelial cell	4/132	41/18870	0.00018956	0.00090887	0.00041547	RELA/TNF/SELE/ALOX5	4
BP	GO:0034502	protein localization to chromosome	6/132	119/18870	0.00019008	0.00091028	0.00041612	CDK1/RB1/PARP1/PLK1/TERT/ESR1	6
BP	GO:0086003	cardiac muscle cell contraction	5/132	76/18870	0.00019267	0.00092159	0.00042128	KCNH2/SCN5A/CAV1/GJA1/ADORA1	5
BP	GO:0006937	regulation of muscle contraction	7/132	170/18870	0.00019511	0.00093219	0.00042613	SCN5A/HSP90AA1/EDN1/CAV1/ADORA1/PIK3CG/PTG	7
BP	GO:0007006	mitochondrial membrane organization	6/132	120/18870	0.00019895	0.00094942	0.00043401	HSP90AA1/BCL2/BAX/TP53/BAD/GSK3B	6
BP	GO:0010506	regulation of autophagy	10/132	355/18870	0.00020028	0.00095467	0.00043641	BCL2/TP53/BAD/MCL1/STAT3/GSK3B/KDR/MET/AKT1/	10
BP	GO:0002260	lymphocyte homeostasis	5/132	77/18870	0.0002049	0.0009744	0.00044543	BCL2/BAX/IL2RA/AKT1/IL2	5
BP	GO:1903036	positive regulation of response to wounding	5/132	77/18870	0.0002049	0.0009744	0.00044543	CLDN4/IGF1R/F2/PTK2/F3	5
BP	GO:0045124	regulation of bone resorption	4/132	42/18870	0.00020839	0.00098983	0.00045248	ITGB3/SPP1/SYK/SRC	4
BP	GO:0090399	replicative senescence	3/132	17/18870	0.00021176	0.00100123	0.00045769	TP53/CDKN1A/TERT	3
BP	GO:0110096	cellular response to aldehyde	3/132	17/18870	0.00021176	0.00100123	0.00045769	AKR1B10/IGF1R/AKR1A1	3
BP	GO:1901163	regulation of trophoblast cell migration	3/132	17/18870	0.00021176	0.00100123	0.00045769	TIMP1/ITGB3/GJA1	3
BP	GO:1901522	positive regulation of transcription from RNA polymerase II promoter invo	3/132	17/18870	0.00021176	0.00100123	0.00045769	RELA/TP53/RUNX2	3
BP	GO:0021782	glial cell development	6/132	122/18870	0.00021767	0.0010258	0.00046892	TNF/APP/ADORA2A/AKT1/MAPT/CDK6	6
BP	GO:0042509	regulation of tyrosine phosphorylation of STAT protein	5/132	78/18870	0.00021772	0.0010258	0.00046892	TNF/IL6R/CAV1/FLT3/IL2	5
BP	GO:0055117	regulation of cardiac muscle contraction	5/132	78/18870	0.00021772	0.0010258	0.00046892	SCN5A/HSP90AA1/CAV1/ADORA1/PIK3CG	5
BP	GO:0046661	male sex differentiation	7/132	174/18870	0.00022503	0.00105903	0.00048411	AR/BCL2/BAX/CYP1B1/AKR1C3/INSR/ESR1	7
BP	GO:0021761	limbic system development	6/132	123/18870	0.00022753	0.0010659	0.00048725	BAX/DRD2/GSK3B/IGF1R/ALK/CDK6	6
BP	GO:0032479	regulation of type I interferon production	6/132	123/18870	0.00022753	0.0010659	0.00048725	HSP90AA1/XIAP/STAT1/SYK/MMP12/PTPN11	6
BP	GO:0032606	type I interferon production	6/132	123/18870	0.00022753	0.0010659	0.00048725	HSP90AA1/XIAP/STAT1/SYK/MMP12/PTPN11	6
BP	GO:2001257	regulation of cation channel activity	6/132	123/18870	0.00022753	0.0010659	0.00048725	DRD2/EDN1/EDNRA/CAV1/MMP9/GPR35	6
BP	GO:1904037	positive regulation of epithelial cell apoptotic process	4/132	43/18870	0.00022852	0.00106931	0.00048881	BAX/CCL2/PLA2G1B/AKR1C3	4
BP	GO:0072331	signal transduction by p53 class mediator	7/132	175/18870	0.00023306	0.00108929	0.00049795	BCL2/BAX/TP53/CDKN1A/MYC/AKT1/NUAK1	7
BP	GO:0019318	hexose metabolic process	8/132	233/18870	0.0002372	0.00110736	0.00050621	TP53/BAD/ADIPOQ/TNF/AKR1B1/SRC/AKT1/INSR	8
BP	GO:0038127	ERBB signaling pathway	6/132	124/18870	0.00023774	0.00110739	0.00050622	MMP9/EGFR/SRC/PTK2/AKT1/PTPN11	6
BP	GO:0046632	alpha-beta T cell differentiation	6/132	124/18870	0.00023774	0.00110739	0.00050622	BCL2/IL6R/IL4R/STAT3/SYK/IL2	6

BP	GO:0007193	adenylate cyclase-inhibiting G protein-coupled receptor signaling pathwa	5/132	80/18870	0.00024517	0.0011381	0.00052026	DRD2/EDN1/EDNRA/ITGB3/ADORA1	5
BP	GO:0032371	regulation of sterol transport	5/132	80/18870	0.00024517	0.0011381	0.00052026	RXRA/ADIPOQ/NFKBIA/CAV1/NR1H3	5
BP	GO:0032374	regulation of cholesterol transport	5/132	80/18870	0.00024517	0.0011381	0.00052026	RXRA/ADIPOQ/NFKBIA/CAV1/NR1H3	5
BP	GO:0002698	negative regulation of immune effector process	6/132	125/18870	0.00024832	0.00115138	0.00052633	TNF/IL4R/ARG1/AXL/IL2/AHR	6
BP	GO:0032635	interleukin-6 production	7/132	177/18870	0.00024981	0.00115274	0.00052695	RELA/TNF/IL6R/APP/STAT3/SYK/PTPN11	7
BP	GO:0032675	regulation of interleukin-6 production	7/132	177/18870	0.00024981	0.00115274	0.00052695	RELA/TNF/IL6R/APP/STAT3/SYK/PTPN11	7
BP	GO:0045840	positive regulation of mitotic nuclear division	4/132	44/18870	0.00025002	0.00115274	0.00052695	TNF/RB1/EDN1/INSR	4
BP	GO:0070741	response to interleukin-6	4/132	44/18870	0.00025002	0.00115274	0.00052695	RELA/IL6R/STAT3/SRC	4
BP	GO:0071526	semaphorin-plexin signaling pathway	4/132	44/18870	0.00025002	0.00115274	0.00052695	EDN1/EDNRA/KDR/MET	4
BP	GO:0044070	regulation of monoatomic anion transport	3/132	18/18870	0.00025282	0.00115652	0.00052868	CA2/ABCB1/CFTR	3
BP	GO:0051000	positive regulation of nitric-oxide synthase activity	3/132	18/18870	0.00025282	0.00115652	0.00052868	AKT1/TERT/DHFR	3
BP	GO:0060576	intestinal epithelial cell development	3/132	18/18870	0.00025282	0.00115652	0.00052868	CDKN1A/SRC/TYMS	3
BP	GO:0061450	trophoblast cell migration	3/132	18/18870	0.00025282	0.00115652	0.00052868	TIMP1/ITGB3/GJA1	3
BP	GO:0071371	cellular response to gonadotropin stimulus	3/132	18/18870	0.00025282	0.00115652	0.00052868	EDN1/EDNRA/CYP1B1	3
BP	GO:1904031	positive regulation of cyclin-dependent protein kinase activity	3/132	18/18870	0.00025282	0.00115652	0.00052868	CCND1/EGFR/AKT1	3
BP	GO:1904044	response to aldosterone	3/132	18/18870	0.00025282	0.00115652	0.00052868	CDKN1A/PARP1/IGF1R	3
BP	GO:2000058	regulation of ubiquitin-dependent protein catabolic process	7/132	178/18870	0.00025854	0.00118137	0.00054004	CDK2/CAV1/GSK3B/PTK2/PLK1/CSNK2A1/AKT1	7
BP	GO:0002688	regulation of leukocyte chemotaxis	6/132	126/18870	0.00025925	0.0011833	0.00054092	CYP19A1/IL6R/EDN1/CCL2/CXCL10/PTK2	6
BP	GO:0051304	chromosome separation	5/132	81/18870	0.00025985	0.0011847	0.00054156	CCNB1/RB1/BIRC5/PLK1/CSNK2A1	5
BP	GO:0051651	maintenance of location in cell	8/132	237/18870	0.0002661	0.00121185	0.00055397	BAX/DRD2/ITGB3/CAV1/CXCL11/CXCL10/F2/AKT1	8
BP	GO:0009267	cellular response to starvation	7/132	179/18870	0.00026752	0.00121695	0.0005563	BCL2/FOS/TP53/CDKN1A/IGF1R/NUAK1/AKR1C3	7
BP	GO:0007260	tyrosine phosphorylation of STAT protein	5/132	82/18870	0.00027519	0.00124627	0.0005697	TNF/IL6R/CAV1/FLT3/IL2	5
BP	GO:0140115	export across plasma membrane	5/132	82/18870	0.00027519	0.00124627	0.0005697	KCNH2/GJA1/ABCG2/ABCC1/ABCB1	5
BP	GO:1901657	glycosyl compound metabolic process	5/132	82/18870	0.00027519	0.00124627	0.0005697	AKR1B1/XDH/AKR1B10/AKR1C3/AKR1A1	5
BP	GO:1905897	regulation of response to endoplasmic reticulum stress	5/132	82/18870	0.00027519	0.00124627	0.0005697	BAX/CAV1/ALOX5/PIK3R1/NR1H3	5
BP	GO:0030301	cholesterol transport	6/132	128/18870	0.00028226	0.00127691	0.00058371	RXRA/ADIPOQ/NFKBIA/CAV1/NR1H3/CFTR	6
BP	GO:0007219	Notch signaling pathway	7/132	181/18870	0.00028623	0.0012934	0.00059125	NFKBIA/APP/IL2RA/STAT3/SRC/AKT1/CDK6	7
BP	GO:0042310	vasoconstriction	5/132	83/18870	0.0002912	0.00131152	0.00059953	EDN1/EDNRA/CAV1/MMP2/PTGS2	5
BP	GO:0051279	regulation of release of sequestered calcium ion into cytosol	5/132	83/18870	0.0002912	0.00131152	0.00059953	BAX/ITGB3/CXCL11/CXCL10/F2	5
BP	GO:0061844	antimicrobial humoral immune response mediated by antimicrobial peptic	5/132	83/18870	0.0002912	0.00131152	0.00059953	CXCL11/CXCL2/CXCL10/F2/PLA2G1B	5
BP	GO:0051972	regulation of telomerase activity	4/132	46/18870	0.00029732	0.00133433	0.00060996	HSP90AA1/TP53/MYC/SRC	4
BP	GO:0002523	leukocyte migration involved in inflammatory response	3/132	19/18870	0.00029869	0.00133433	0.00060996	TNF/SELE/ALOX5	3
BP	GO:0035821	modulation of process of another organism	3/132	19/18870	0.00029869	0.00133433	0.00060996	SYK/ARG1/AKT1	3
BP	GO:0038166	angiotensin-activated signaling pathway	3/132	19/18870	0.00029869	0.00133433	0.00060996	CAV1/CA2/SRC	3
BP	GO:0070431	nucleotide-binding oligomerization domain containing 2 signaling pathwa	3/132	19/18870	0.00029869	0.00133433	0.00060996	RELA/NFKBIA/XIAP	3
BP	GO:0071391	cellular response to estrogen stimulus	3/132	19/18870	0.00029869	0.00133433	0.00060996	AR/ESR1/ESR2	3
BP	GO:0097254	renal tubular secretion	3/132	19/18870	0.00029869	0.00133433	0.00060996	DRD2/EDN1/ABCG2	3
BP	GO:0002263	cell activation involved in immune response	9/132	305/18870	0.00029888	0.00133433	0.00060996	TP53/TNF/IL6R/APP/IL4R/STAT3/SYK/IL2/PIK3CG	9
BP	GO:0045621	positive regulation of lymphocyte differentiation	6/132	130/18870	0.00030685	0.00136694	0.00062487	BAD/IL2RA/IL4R/SYK/AXL/IL2	6
BP	GO:0050864	regulation of B cell activation	6/132	130/18870	0.00030685	0.00136694	0.00062487	BCL2/BAD/CDKN1A/SYK/IL2/AHR	6
BP	GO:0050871	positive regulation of B cell activation	5/132	84/18870	0.00030791	0.00136867	0.00062566	BCL2/BAD/CDKN1A/SYK/IL2	5
BP	GO:0072332	intrinsic apoptotic signaling pathway by p53 class mediator	5/132	84/18870	0.00030791	0.00136867	0.00062566	BCL2/BAX/TP53/CDKN1A/MYC	5
BP	GO:0044282	small molecule catabolic process	10/132	375/18870	0.00031019	0.00137731	0.00062961	TP53/BAD/ADIPOQ/SULT1E1/XDH/AKR1B10/ARG1/AK	10
BP	GO:0051983	regulation of chromosome segregation	6/132	131/18870	0.00031976	0.00141825	0.00064832	CDK1/CCNB1/RB1/BIRC5/PLK1/CSNK2A1	6
BP	GO:0035850	epithelial cell differentiation involved in kidney development	4/132	47/18870	0.00032322	0.00143207	0.00065464	ADIPOQ/EDNRA/STAT1/MMP9	4
BP	GO:0060560	developmental growth involved in morphogenesis	8/132	244/18870	0.00032361	0.00143221	0.00065471	HSP90AA1/APP/EDN1/EDNRA/SPP1/GSK3B/MAPT/ESI	8
BP	GO:0032414	positive regulation of ion transmembrane transporter activity	5/132	85/18870	0.00032534	0.00143676	0.00065678	EDN1/EDNRA/CCL2/ABCB1/CFTR	5
BP	GO:0071230	cellular response to amino acid stimulus	5/132	85/18870	0.00032534	0.00143676	0.00065678	TNF/COL1A1/COL3A1/MMP2/EGFR	5
BP	GO:0032640	tumor necrosis factor production	7/132	186/18870	0.00033762	0.0014862	0.00067939	ADIPOQ/APP/STAT3/SYK/PIK3R1/AXL/PTPN11	7

BP	GO:0032680	regulation of tumor necrosis factor production	7/132	186/18870	0.00033762	0.0014862	0.00067939	ADIPOQ/APP/STAT3/SYK/PIK3R1/AXL/PTPN11	7
BP	GO:0032872	regulation of stress-activated MAPK cascade	7/132	186/18870	0.00033762	0.0014862	0.00067939	TNF/MYC/APP/XIAP/XDH/IGF1R/EGFR	7
BP	GO:0002244	hematopoietic progenitor cell differentiation	6/132	133/18870	0.00034685	0.00152515	0.00069719	BCL2/TP53/FLT3/KDR/TOP2A/CDK6	6
BP	GO:0060749	mammary gland alveolus development	3/132	20/18870	0.00034961	0.0015306	0.00069968	AR/CCND1/ESR1	3
BP	GO:0061377	mammary gland lobule development	3/132	20/18870	0.00034961	0.0015306	0.00069968	AR/CCND1/ESR1	3
BP	GO:1900221	regulation of amyloid-beta clearance	3/132	20/18870	0.00034961	0.0015306	0.00069968	TNF/HMGCR/CYP51A1	3
BP	GO:0031295	T cell costimulation	4/132	48/18870	0.00035071	0.0015306	0.00069968	CAV1/SRC/AKT1/PTPN11	4
BP	GO:0032369	negative regulation of lipid transport	4/132	48/18870	0.00035071	0.0015306	0.00069968	ITGB3/AKT1/PTPN11/NR1H3	4
BP	GO:0042551	neuron maturation	4/132	48/18870	0.00035071	0.0015306	0.00069968	BCL2/RB1/APP/EDNRA	4
BP	GO:0045776	negative regulation of blood pressure	4/132	48/18870	0.00035071	0.0015306	0.00069968	ADIPOQ/TNF/DRD2/ADORA1	4
BP	GO:0090257	regulation of muscle system process	8/132	247/18870	0.00035117	0.001531	0.00069986	SCN5A/HSP90AA1/EDN1/CAV1/ADORA1/PARP1/PIK3	8
BP	GO:0044000	movement in host	7/132	188/18870	0.00036015	0.00156845	0.00071698	CDK1/ITGB3/CAV1/EGFR/SRC/AXL/INSR	7
BP	GO:0007188	adenylate cyclase-modulating G protein-coupled receptor signaling pathw	8/132	248/18870	0.00036078	0.00156952	0.00071747	DRD2/EDN1/EDNRA/ITGB3/CXCL11/CXCL10/ADORA1	8
BP	GO:0010232	vascular transport	5/132	87/18870	0.00036242	0.00157165	0.00071844	SLC2A4/ABCG2/ABCC1/ABCB1/INSR	5
BP	GO:0071277	cellular response to calcium ion	5/132	87/18870	0.00036242	0.00157165	0.00071844	SCN5A/FOS/EDN1/ALOX15/AKR1C3	5
BP	GO:0150104	transport across blood-brain barrier	5/132	87/18870	0.00036242	0.00157165	0.00071844	SLC2A4/ABCG2/ABCC1/ABCB1/INSR	5
BP	GO:0030308	negative regulation of cell growth	7/132	189/18870	0.00037185	0.00160743	0.0007348	BCL2/TP53/CDKN1A/RB1/GJA1/SPP1/ESR2	7
BP	GO:0042770	signal transduction in response to DNA damage	7/132	189/18870	0.00037185	0.00160743	0.0007348	TP53/CDK1/CDK2/CDKN1A/CCND1/PLK1/PTPN11	7
BP	GO:0070302	regulation of stress-activated protein kinase signaling cascade	7/132	189/18870	0.00037185	0.00160743	0.0007348	TNF/MYC/APP/XIAP/XDH/IGF1R/EGFR	7
BP	GO:0002673	regulation of acute inflammatory response	4/132	49/18870	0.00037983	0.00163326	0.00074661	TNF/ADORA1/PIK3CG/PTGS2	4
BP	GO:0046427	positive regulation of receptor signaling pathway via JAK-STAT	4/132	49/18870	0.00037983	0.00163326	0.00074661	TNF/IL6R/CYP1B1/F2	4
BP	GO:0046850	regulation of bone remodeling	4/132	49/18870	0.00037983	0.00163326	0.00074661	ITGB3/SPP1/SYK/SRC	4
BP	GO:0070266	necroptotic process	4/132	49/18870	0.00037983	0.00163326	0.00074661	TP53/TNF/CAV1/PARP1	4
BP	GO:2001258	negative regulation of cation channel activity	4/132	49/18870	0.00037983	0.00163326	0.00074661	DRD2/CAV1/MMP9/GPR35	4
BP	GO:0051209	release of sequestered calcium ion into cytosol	6/132	136/18870	0.00039081	0.00167874	0.0007674	BAX/DRD2/ITGB3/CXCL11/CXCL10/F2	6
BP	GO:0021543	pallium development	7/132	191/18870	0.00039618	0.00169643	0.00077549	BAX/COL3A1/GSK3B/IGF1R/EGFR/ALK/CDK6	7
BP	GO:0071706	tumor necrosis factor superfamily cytokine production	7/132	191/18870	0.00039618	0.00169643	0.00077549	ADIPOQ/APP/STAT3/SYK/PIK3R1/AXL/PTPN11	7
BP	GO:1903555	regulation of tumor necrosis factor superfamily cytokine production	7/132	191/18870	0.00039618	0.00169643	0.00077549	ADIPOQ/APP/STAT3/SYK/PIK3R1/AXL/PTPN11	7
BP	GO:0002429	immune response-activating cell surface receptor signaling pathway	9/132	318/18870	0.00040529	0.00171858	0.00078561	RELA/BCL2/BAX/NFKBIA/SYK/PIK3R1/SRC/PTK2/NR1H	9
BP	GO:0002902	regulation of B cell apoptotic process	3/132	21/18870	0.0004058	0.00171858	0.00078561	BCL2/BAX/IL2	3
BP	GO:0006907	pinocytosis	3/132	21/18870	0.0004058	0.00171858	0.00078561	CAV1/AXL/NR1H3	3
BP	GO:0010224	response to UV-B	3/132	21/18870	0.0004058	0.00171858	0.00078561	RELA/BCL2/CDKN1A	3
BP	GO:0019370	leukotriene biosynthetic process	3/132	21/18870	0.0004058	0.00171858	0.00078561	ALOX5/SYK/PLA2G1B	3
BP	GO:0043651	linoleic acid metabolic process	3/132	21/18870	0.0004058	0.00171858	0.00078561	ALOX5/ALOX15/ALOX12	3
BP	GO:0044321	response to leptin	3/132	21/18870	0.0004058	0.00171858	0.00078561	CCND1/STAT3/EDN1	3
BP	GO:0060444	branching involved in mammary gland duct morphogenesis	3/132	21/18870	0.0004058	0.00171858	0.00078561	AR/SRC/ESR1	3
BP	GO:0071498	cellular response to fluid shear stress	3/132	21/18870	0.0004058	0.00171858	0.00078561	MMP2/SRC/PTGS2	3
BP	GO:0097284	hepatocyte apoptotic process	3/132	21/18870	0.0004058	0.00171858	0.00078561	RB1/IGF1R/PIK3CG	3
BP	GO:0051283	negative regulation of sequestering of calcium ion	6/132	137/18870	0.0004064	0.00171858	0.00078561	BAX/DRD2/ITGB3/CXCL11/CXCL10/F2	6
BP	GO:1904375	regulation of protein localization to cell periphery	6/132	137/18870	0.0004064	0.00171858	0.00078561	AR/TNF/EGFR/PIK3R1/PLK1/AKT1	6
BP	GO:0009299	mRNA transcription	4/132	50/18870	0.00041063	0.00172754	0.00078971	RXRA/TP53/STAT3/NR1H3	4
BP	GO:0030850	prostate gland development	4/132	50/18870	0.00041063	0.00172754	0.00078971	AR/CYP19A1/MMP2/ESR1	4
BP	GO:0031294	lymphocyte costimulation	4/132	50/18870	0.00041063	0.00172754	0.00078971	CAV1/SRC/AKT1/PTPN11	4
BP	GO:0048066	developmental pigmentation	4/132	50/18870	0.00041063	0.00172754	0.00078971	BCL2/BAX/EDNRA/TYR	4
BP	GO:0071385	cellular response to glucocorticoid stimulus	4/132	50/18870	0.00041063	0.00172754	0.00078971	EDN1/FLT3/CYP1B1/IGF1R	4
BP	GO:0006006	glucose metabolic process	7/132	193/18870	0.00042176	0.00177071	0.00080944	TP53/BAD/ADIPOQ/TNF/SRC/AKT1/INSR	7
BP	GO:0033673	negative regulation of kinase activity	7/132	193/18870	0.00042176	0.00177071	0.00080944	ADIPOQ/CDKN1A/RB1/CAV1/PLK1/AKT1/MAPT	7
BP	GO:0043401	steroid hormone mediated signaling pathway	6/132	138/18870	0.00042247	0.00177185	0.00080996	RXRA/AR/PARP1/SRC/ESR1/ESR2	6
BP	GO:0010657	muscle cell apoptotic process	5/132	90/18870	0.00042394	0.00177436	0.00081111	HSP90AA1/TP53/EDN1/IGF1R/ALOX12	5

BP	GO:0046889	positive regulation of lipid biosynthetic process	5/132	90/18870	0.00042394	0.00177436	0.00081111	TNF/IGF1R/AKT1/NR1H3/PTGS2	5
BP	GO:0051282	regulation of sequestering of calcium ion	6/132	139/18870	0.00043903	0.00183564	0.00083912	BAX/DRD2/ITGB3/CXCL11/CXCL10/F2	6
BP	GO:0046622	positive regulation of organ growth	4/132	51/18870	0.00044318	0.00185113	0.00084621	CDK1/EDN1/PIM1/AKT1	4
BP	GO:1900273	positive regulation of long-term synaptic potentiation	3/132	22/18870	0.00046747	0.0019486	0.00089076	DRD2/APP/ADORA2A	3
BP	GO:2000831	regulation of steroid hormone secretion	3/132	22/18870	0.00046747	0.0019486	0.00089076	CYP19A1/SPP1/PTPN11	3
BP	GO:0050886	endocrine process	5/132	92/18870	0.00046911	0.00195344	0.00089297	CYP19A1/EDN1/GJA1/SPP1/PTPN11	5
BP	GO:0031647	regulation of protein stability	9/132	325/18870	0.00047444	0.00197361	0.00090219	HSP90AA1/BCL2/TP53/PIM1/PIK3R1/SRC/PLK1/CSNK2	9
BP	GO:0042572	retinol metabolic process	4/132	52/18870	0.00047753	0.00197642	0.00090348	AKR1B1/CYP1B1/AKR1B10/AKR1C3	4
BP	GO:0048009	insulin-like growth factor receptor signaling pathway	4/132	52/18870	0.00047753	0.00197642	0.00090348	AR/IGF1R/PIK3R1/AKT1	4
BP	GO:0060324	face development	4/132	52/18870	0.00047753	0.00197642	0.00090348	EDNRA/COL1A1/MMP2/PTPN11	4
BP	GO:0071470	cellular response to osmotic stress	4/132	52/18870	0.00047753	0.00197642	0.00090348	BAD/SLC2A4/AKR1B1/PTGS2	4
BP	GO:1904263	positive regulation of TORC1 signaling	4/132	52/18870	0.00047753	0.00197642	0.00090348	SYK/PIM1/SRC/AKT1	4
BP	GO:2000106	regulation of leukocyte apoptotic process	5/132	93/18870	0.00049302	0.00203843	0.00093182	BCL2/BAX/TP53/AXL/IL2	5
BP	GO:0003158	endothelium development	6/132	143/18870	0.00051039	0.00210601	0.00096272	TNF/EDNRA/CXCL10/XDH/KDR/MET	6
BP	GO:0051208	sequestering of calcium ion	6/132	143/18870	0.00051039	0.00210601	0.00096272	BAX/DRD2/ITGB3/CXCL11/CXCL10/F2	6
BP	GO:0002762	negative regulation of myeloid leukocyte differentiation	4/132	53/18870	0.00051374	0.00210917	0.00096416	ADIPOQ/MYC/PIK3R1/CDK6	4
BP	GO:0045058	T cell selection	4/132	53/18870	0.00051374	0.00210917	0.00096416	BCL2/IL6R/STAT3/SYK	4
BP	GO:0048260	positive regulation of receptor-mediated endocytosis	4/132	53/18870	0.00051374	0.00210917	0.00096416	DRD2/SELE/SYK/INSR	4
BP	GO:0086002	cardiac muscle cell action potential involved in contraction	4/132	53/18870	0.00051374	0.00210917	0.00096416	KCNH2/SCN5A/CAV1/GJA1	4
BP	GO:1904894	positive regulation of receptor signaling pathway via STAT	4/132	53/18870	0.00051374	0.00210917	0.00096416	TNF/IL6R/CYP1B1/F2	4
BP	GO:0001656	metanephros development	5/132	94/18870	0.00051782	0.00211955	0.00096891	BCL2/ADIPOQ/MYC/STAT1/AKR1B1	5
BP	GO:0019915	lipid storage	5/132	94/18870	0.00051782	0.00211955	0.00096891	TNF/NFKBIA/ITGB3/CAV1/NR1H3	5
BP	GO:0071229	cellular response to acid chemical	5/132	94/18870	0.00051782	0.00211955	0.00096891	TNF/COL1A1/COL3A1/MMP2/EGFR	5
BP	GO:0010565	regulation of cellular ketone metabolic process	6/132	144/18870	0.00052957	0.00216329	0.0009889	ADIPOQ/CAV1/AKT1/AKR1C3/NR1H3/PTGS2	6
BP	GO:0050714	positive regulation of protein secretion	6/132	144/18870	0.00052957	0.00216329	0.0009889	BAD/F2/ADORA2A/PLA2G1B/ACHE/CFTR	6
BP	GO:0060065	uterus development	3/132	23/18870	0.00053485	0.00218051	0.00099677	CYP19A1/SRC/ESR1	3
BP	GO:0061042	vascular wound healing	3/132	23/18870	0.00053485	0.00218051	0.00099677	TNF/ALOX5/KDR	3
BP	GO:0003073	regulation of systemic arterial blood pressure	5/132	95/18870	0.00054356	0.00220939	0.00100998	AR/TNF/DRD2/EDN1/ADORA1	5
BP	GO:0007044	cell-substrate junction assembly	5/132	95/18870	0.00054356	0.00220939	0.00100998	BCL2/ITGB3/SRC/PTK2/KDR	5
BP	GO:0045638	negative regulation of myeloid cell differentiation	5/132	95/18870	0.00054356	0.00220939	0.00100998	ADIPOQ/MYC/NFKBIA/PIK3R1/CDK6	5
BP	GO:0062207	regulation of pattern recognition receptor signaling pathway	6/132	145/18870	0.0005493	0.00223051	0.00101963	XIAP/CAV1/PIK3R1/SRC/NR1H3/ESR1	6
BP	GO:0010883	regulation of lipid storage	4/132	54/18870	0.00055185	0.002232	0.00102031	TNF/NFKBIA/ITGB3/NR1H3	4
BP	GO:0033047	regulation of mitotic sister chromatid segregation	4/132	54/18870	0.00055185	0.002232	0.00102031	CDK1/CCNB1/BIRC5/PLK1	4
BP	GO:0050873	brown fat cell differentiation	4/132	54/18870	0.00055185	0.002232	0.00102031	ADIPOQ/SLC2A4/PIM1/PTGS2	4
BP	GO:2000772	regulation of cellular senescence	4/132	54/18870	0.00055185	0.002232	0.00102031	TP53/NUAK1/TERT/CDK6	4
BP	GO:0002695	negative regulation of leukocyte activation	7/132	202/18870	0.00055373	0.00223737	0.00102276	IL2RA/IL4R/ARG1/ADORA2A/AXL/IL2/NR1H3	7
BP	GO:0043122	regulation of canonical NF-kappaB signal transduction	8/132	265/18870	0.00055967	0.00225915	0.00103272	RELA/ADIPOQ/TNF/XIAP/STAT1/GJA1/PARP1/ESR1	8
BP	GO:0001508	action potential	6/132	146/18870	0.0005696	0.0022947	0.00104897	KCNH2/SCN5A/TNF/CAV1/GJA1/GPR35	6
BP	GO:0002753	cytosolic pattern recognition receptor signaling pathway	6/132	146/18870	0.0005696	0.0022947	0.00104897	RELA/TNF/NFKBIA/XIAP/CAV1/SRC	6
BP	GO:0070663	regulation of leukocyte proliferation	8/132	267/18870	0.00058798	0.00236642	0.00108176	BCL2/CDKN1A/IL2RA/SYK/ARG1/PTK2/IL2/AHR	8
BP	GO:0002686	negative regulation of leukocyte migration	4/132	55/18870	0.00059194	0.00237531	0.00108582	CYP19A1/CCL2/ADORA1/AKT1	4
BP	GO:0034142	toll-like receptor 4 signaling pathway	4/132	55/18870	0.00059194	0.00237531	0.00108582	RELA/NFKBIA/PIK3R1/NR1H3	4
BP	GO:2000648	positive regulation of stem cell proliferation	4/132	55/18870	0.00059194	0.00237531	0.00108582	GJA1/RUNX2/KDR/TERT	4
BP	GO:0010640	regulation of platelet-derived growth factor receptor signaling pathway	3/132	24/18870	0.00060814	0.00242842	0.0011101	ADIPOQ/SRC/F3	3
BP	GO:0032891	negative regulation of organic acid transport	3/132	24/18870	0.00060814	0.00242842	0.0011101	TNF/ADORA1/AKT1	3
BP	GO:0034138	toll-like receptor 3 signaling pathway	3/132	24/18870	0.00060814	0.00242842	0.0011101	TNF/CAV1/SRC	3
BP	GO:0046628	positive regulation of insulin receptor signaling pathway	3/132	24/18870	0.00060814	0.00242842	0.0011101	SRC/PTPN11/CTSD	3
BP	GO:1905048	regulation of metallopeptidase activity	3/132	24/18870	0.00060814	0.00242842	0.0011101	TIMP1/STAT3/CLDN4	3
BP	GO:0060078	regulation of postsynaptic membrane potential	6/132	148/18870	0.00061195	0.00244123	0.00111595	DRD2/APP/ADORA1/GSK3B/ADORA2A/AKT1	6

BP	GO:0007178	transmembrane receptor protein serine/threonine kinase signaling pathwa	10/132	410/18870	0.00062291	0.00248254	0.00113484	FOS/TP53/XIAP/STAT3/COL3A1/RUNX2/PARP1/SRC/P	10
BP	GO:0030111	regulation of Wnt signaling pathway	9/132	338/18870	0.0006286	0.00250278	0.00114409	APP/XIAP/CAV1/COL1A1/GSK3B/EGFR/SRC/CSNK2A1/	9
BP	GO:0001954	positive regulation of cell-matrix adhesion	4/132	56/18870	0.00063404	0.00251219	0.0011484	ITGB3/GSK3B/KDR/CDK6	4
BP	GO:0043525	positive regulation of neuron apoptotic process	4/132	56/18870	0.00063404	0.00251219	0.0011484	BAX/TP53/TNF/MCL1	4
BP	GO:0045912	negative regulation of carbohydrate metabolic process	4/132	56/18870	0.00063404	0.00251219	0.0011484	TP53/ADIPOQ/STAT3/GSK3B	4
BP	GO:0060986	endocrine hormone secretion	4/132	56/18870	0.00063404	0.00251219	0.0011484	CYP19A1/GJA1/SPP1/PTPN11	4
BP	GO:0061097	regulation of protein tyrosine kinase activity	4/132	56/18870	0.00063404	0.00251219	0.0011484	APP/CAV1/NOX4/SRC	4
BP	GO:0048285	organelle fission	11/132	488/18870	0.00064297	0.00254507	0.00116342	CDK1/CCNB1/TNF/RB1/BIRC5/EDN1/KDR/PLK1/MAPT	11
BP	GO:0006942	regulation of striated muscle contraction	5/132	99/18870	0.00065622	0.00259442	0.00118598	SCN5A/HSP90AA1/CAV1/ADORA1/PIK3CG	5
BP	GO:0007612	learning	6/132	150/18870	0.0006567	0.00259442	0.00118598	FOS/DRD2/APP/HMGCR/INSR/PTGS2	6
BP	GO:0061005	cell differentiation involved in kidney development	4/132	57/18870	0.00067823	0.00267431	0.0012225	ADIPOQ/EDNRA/STAT1/MMP9	4
BP	GO:0061900	glial cell activation	4/132	57/18870	0.00067823	0.00267431	0.0012225	TNF/APP/ADORA2A/MAPT	4
BP	GO:0001890	placenta development	6/132	151/18870	0.00068001	0.00267872	0.00122452	SPP1/PARP1/EGFR/PTK2/AKT1/PTGS2	6
BP	GO:0019217	regulation of fatty acid metabolic process	5/132	100/18870	0.00068693	0.00268257	0.00122628	ADIPOQ/CAV1/AKT1/NR1H3/PTGS2	5
BP	GO:0034446	substrate adhesion-dependent cell spreading	5/132	100/18870	0.00068693	0.00268257	0.00122628	ITGB3/PIK3R1/SRC/PTK2/AXL	5
BP	GO:0050764	regulation of phagocytosis	5/132	100/18870	0.00068693	0.00268257	0.00122628	ADIPOQ/TNF/CCL2/SYK/ALOX15	5
BP	GO:0071901	negative regulation of protein serine/threonine kinase activity	5/132	100/18870	0.00068693	0.00268257	0.00122628	ADIPOQ/CDKN1A/RB1/PLK1/AKT1	5
BP	GO:0002053	positive regulation of mesenchymal cell proliferation	3/132	25/18870	0.00068755	0.00268257	0.00122628	MYC/STAT1/KDR	3
BP	GO:0002363	alpha-beta T cell lineage commitment	3/132	25/18870	0.00068755	0.00268257	0.00122628	BCL2/IL6R/STAT3	3
BP	GO:0006706	steroid catabolic process	3/132	25/18870	0.00068755	0.00268257	0.00122628	CYP19A1/SULT1E1/SPP1	3
BP	GO:0009110	vitamin biosynthetic process	3/132	25/18870	0.00068755	0.00268257	0.00122628	TNF/AKR1B1/AKR1A1	3
BP	GO:0046697	decidualization	3/132	25/18870	0.00068755	0.00268257	0.00122628	SPP1/PARP1/PTGS2	3
BP	GO:0060396	growth hormone receptor signaling pathway	3/132	25/18870	0.00068755	0.00268257	0.00122628	STAT3/PIK3R1/PTK2	3
BP	GO:0140014	mitotic nuclear division	8/132	274/18870	0.00069635	0.00271376	0.00124054	CDK1/CCNB1/TNF/RB1/BIRC5/EDN1/PLK1/INSR	8
BP	GO:0045732	positive regulation of protein catabolic process	7/132	210/18870	0.00069688	0.00271376	0.00124054	HSP90AA1/TNF/CAV1/GSK3B/PLK1/CSNK2A1/AKT1	7
BP	GO:1903038	negative regulation of leukocyte cell-cell adhesion	6/132	152/18870	0.00070395	0.00273871	0.00125194	IL2RA/IL4R/ARG1/ADORA2A/AKT1/IL2	6
BP	GO:0042306	regulation of protein import into nucleus	4/132	58/18870	0.00072456	0.00281085	0.00128492	HSP90AA1/CDK1/PIK3R1/PTGS2	4
BP	GO:0051932	synaptic transmission, GABAergic	4/132	58/18870	0.00072456	0.00281085	0.00128492	DRD2/CA2/ADORA1/ADORA2A	4
BP	GO:0061756	leukocyte adhesion to vascular endothelial cell	4/132	58/18870	0.00072456	0.00281085	0.00128492	RELA/TNF/SELE/ALOX5	4
BP	GO:0002768	immune response-regulating cell surface receptor signaling pathway	9/132	346/18870	0.00074235	0.00287713	0.00131522	RELA/BCL2/BAX/NFKBIA/SYK/PIK3R1/SRC/PTK2/NR1H	9
BP	GO:0016125	sterol metabolic process	6/132	154/18870	0.00075379	0.00291871	0.00133422	CYP19A1/APP/CYP1B1/HMGCR/CYP51A1/CFTR	6
BP	GO:0042098	T cell proliferation	7/132	213/18870	0.00075756	0.00293051	0.00133962	BAX/TP53/IL2RA/SYK/ARG1/IL2/PIK3CG	7
BP	GO:0002823	negative regulation of adaptive immune response based on somatic recor	4/132	59/18870	0.00077308	0.00294942	0.00134826	IL4R/ARG1/IL2/AHR	4
BP	GO:0010965	regulation of mitotic sister chromatid separation	4/132	59/18870	0.00077308	0.00294942	0.00134826	CCNB1/RB1/BIRC5/PLK1	4
BP	GO:0046579	positive regulation of Ras protein signal transduction	4/132	59/18870	0.00077308	0.00294942	0.00134826	COL3A1/SRC/GPR35/PIK3CG	4
BP	GO:0098900	regulation of action potential	4/132	59/18870	0.00077308	0.00294942	0.00134826	SCN5A/TNF/CAV1/GPR35	4
BP	GO:0002433	immune response-regulating cell surface receptor signaling pathway invol	3/132	26/18870	0.00077327	0.00294942	0.00134826	SYK/SRC/PTK2	3
BP	GO:0032469	endoplasmic reticulum calcium ion homeostasis	3/132	26/18870	0.00077327	0.00294942	0.00134826	BCL2/BAX/APP	3
BP	GO:0033598	mammary gland epithelial cell proliferation	3/132	26/18870	0.00077327	0.00294942	0.00134826	BAX/CCND1/ESR1	3
BP	GO:0038096	Fc-gamma receptor signaling pathway involved in phagocytosis	3/132	26/18870	0.00077327	0.00294942	0.00134826	SYK/SRC/PTK2	3
BP	GO:0043369	CD4-positive or CD8-positive, alpha-beta T cell lineage commitment	3/132	26/18870	0.00077327	0.00294942	0.00134826	BCL2/IL6R/STAT3	3
BP	GO:0044346	fibroblast apoptotic process	3/132	26/18870	0.00077327	0.00294942	0.00134826	TP53/MYC/PIK3CG	3
BP	GO:0060740	prostate gland epithelium morphogenesis	3/132	26/18870	0.00077327	0.00294942	0.00134826	AR/MMP2/ESR1	3
BP	GO:0070102	interleukin-6-mediated signaling pathway	3/132	26/18870	0.00077327	0.00294942	0.00134826	IL6R/STAT3/SRC	3
BP	GO:0071378	cellular response to growth hormone stimulus	3/132	26/18870	0.00077327	0.00294942	0.00134826	STAT3/PIK3R1/PTK2	3
BP	GO:0090218	positive regulation of lipid kinase activity	3/132	26/18870	0.00077327	0.00294942	0.00134826	FLT3/F2/MAPT	3
BP	GO:1900078	positive regulation of cellular response to insulin stimulus	3/132	26/18870	0.00077327	0.00294942	0.00134826	SRC/PTPN11/CTSD	3
BP	GO:0016051	carbohydrate biosynthetic process	7/132	214/18870	0.00077869	0.00296729	0.00135643	ADIPOQ/AKR1B1/GSK3B/PARP1/AKT1/AKR1A1/INSR	7
BP	GO:0045639	positive regulation of myeloid cell differentiation	5/132	103/18870	0.00078553	0.00299058	0.00136708	FOS/TNF/RB1/STAT3/STAT1	5

BP	GO:0000723	telomere maintenance	6/132	156/18870	0.00080632	0.00306116	0.00139934	HSP90AA1/MYC/PARP1/SRC/TERT/APEX1	6
BP	GO:0030010	establishment of cell polarity	6/132	156/18870	0.00080632	0.00306116	0.00139934	HSP90AA1/GJA1/GSK3B/IGF1R/PTK2/PLK1	6
BP	GO:0046718	viral entry into host cell	6/132	156/18870	0.00080632	0.00306116	0.00139934	CDK1/ITGB3/CAV1/EGFR/AXL/INSR	6
BP	GO:0033045	regulation of sister chromatid segregation	5/132	104/18870	0.00082063	0.00310972	0.00142154	CDK1/CCNB1/RB1/BIRC5/PLK1	5
BP	GO:0042100	B cell proliferation	5/132	104/18870	0.00082063	0.00310972	0.00142154	BCL2/BAX/CDKN1A/IL2/AHR	5
BP	GO:0042908	xenobiotic transport	4/132	60/18870	0.00082385	0.00311325	0.00142316	GJA1/ABCG2/ABCC1/ABCB1	4
BP	GO:0051785	positive regulation of nuclear division	4/132	60/18870	0.00082385	0.00311325	0.00142316	TNF/RB1/EDN1/INSR	4
BP	GO:0070228	regulation of lymphocyte apoptotic process	4/132	60/18870	0.00082385	0.00311325	0.00142316	BCL2/BAX/TP53/IL2	4
BP	GO:0001678	intracellular glucose homeostasis	6/132	157/18870	0.00083362	0.00314726	0.0014387	BAD/KLF7/NOX4/IGF1R/PIK3R1/CFTR	6
BP	GO:0070252	actin-mediated cell contraction	5/132	105/18870	0.00085689	0.00323213	0.0014775	KCNH2/SCN5A/CAV1/GJA1/ADORA1	5
BP	GO:0001783	B cell apoptotic process	3/132	27/18870	0.0008655	0.00324663	0.00148413	BCL2/BAX/IL2	3
BP	GO:0007205	protein kinase C-activating G protein-coupled receptor signaling pathway	3/132	27/18870	0.0008655	0.00324663	0.00148413	EDN1/ADORA2A/IL2	3
BP	GO:0010818	T cell chemotaxis	3/132	27/18870	0.0008655	0.00324663	0.00148413	CXCL11/CXCL10/PIK3CG	3
BP	GO:0035929	steroid hormone secretion	3/132	27/18870	0.0008655	0.00324663	0.00148413	CYP19A1/SPP1/PTPN11	3
BP	GO:0045745	positive regulation of G protein-coupled receptor signaling pathway	3/132	27/18870	0.0008655	0.00324663	0.00148413	DRD2/ITGB3/F2	3
BP	GO:0051984	positive regulation of chromosome segregation	3/132	27/18870	0.0008655	0.00324663	0.00148413	CDK1/CCNB1/BIRC5	3
BP	GO:0042594	response to starvation	7/132	218/18870	0.00086792	0.00325272	0.00148691	BCL2/FOS/TP53/CDKN1A/IGF1R/NUAK1/AKR1C3	7
BP	GO:0060350	endochondral bone morphogenesis	4/132	61/18870	0.00087693	0.00327746	0.00149822	COL1A1/COL3A1/RUNX2/MMP13	4
BP	GO:1903078	positive regulation of protein localization to plasma membrane	4/132	61/18870	0.00087693	0.00327746	0.00149822	TNF/EGFR/PIK3R1/AKT1	4
BP	GO:1905517	macrophage migration	4/132	61/18870	0.00087693	0.00327746	0.00149822	CYP19A1/CCL2/MMP2/PTK2	4
BP	GO:0006641	triglyceride metabolic process	5/132	106/18870	0.00089433	0.00332121	0.00151822	MTTP/CAV1/PTPN11/NR1H3/PIK3CG	5
BP	GO:0007631	feeding behavior	5/132	106/18870	0.00089433	0.00332121	0.00151822	FOS/DRD2/APP/STAT3/ADORA2A	5
BP	GO:0030316	osteoclast differentiation	5/132	106/18870	0.00089433	0.00332121	0.00151822	FOS/TNF/GLO1/PIK3R1/SRC	5
BP	GO:0032526	response to retinoic acid	5/132	106/18870	0.00089433	0.00332121	0.00151822	RXRA/TNF/COL1A1/GSK3B/MMP2	5
BP	GO:0032760	positive regulation of tumor necrosis factor production	5/132	106/18870	0.00089433	0.00332121	0.00151822	APP/STAT3/SYK/PIK3R1/PTPN11	5
BP	GO:0035303	regulation of dephosphorylation	5/132	106/18870	0.00089433	0.00332121	0.00151822	TNF/ADORA1/GSK3B/SRC/NUAK1	5
BP	GO:0046425	regulation of receptor signaling pathway via JAK-STAT	5/132	106/18870	0.00089433	0.00332121	0.00151822	TNF/IL6R/CAV1/CYP1B1/F2	5
BP	GO:0032731	positive regulation of interleukin-1 beta production	4/132	62/18870	0.00093238	0.00345217	0.00157808	RELA/TNF/APP/STAT3	4
BP	GO:0051306	mitotic sister chromatid separation	4/132	62/18870	0.00093238	0.00345217	0.00157808	CCNB1/RB1/BIRC5/PLK1	4
BP	GO:0090303	positive regulation of wound healing	4/132	62/18870	0.00093238	0.00345217	0.00157808	CLDN4/F2/PTK2/F3	4
BP	GO:1901796	regulation of signal transduction by p53 class mediator	5/132	107/18870	0.00093297	0.00345217	0.00157808	BCL2/TP53/MYC/AKT1/NUAK1	5
BP	GO:0001963	synaptic transmission, dopaminergic	3/132	28/18870	0.00096443	0.00353019	0.00161375	DRD2/ADORA2A/PTGS2	3
BP	GO:0002675	positive regulation of acute inflammatory response	3/132	28/18870	0.00096443	0.00353019	0.00161375	TNF/PIK3CG/PTGS2	3
BP	GO:0007202	activation of phospholipase C activity	3/132	28/18870	0.00096443	0.00353019	0.00161375	EDNRA/SELE/EGFR	3
BP	GO:0014047	glutamate secretion	3/132	28/18870	0.00096443	0.00353019	0.00161375	GJA1/ADORA1/ADORA2A	3
BP	GO:0032104	regulation of response to extracellular stimulus	3/132	28/18870	0.00096443	0.00353019	0.00161375	RXRA/BCL2/BAX	3
BP	GO:0032107	regulation of response to nutrient levels	3/132	28/18870	0.00096443	0.00353019	0.00161375	RXRA/BCL2/BAX	3
BP	GO:0034698	response to gonadotropin	3/132	28/18870	0.00096443	0.00353019	0.00161375	EDN1/EDNRA/CYP1B1	3
BP	GO:0042759	long-chain fatty acid biosynthetic process	3/132	28/18870	0.00096443	0.00353019	0.00161375	ALOX5/ALOX15/ALOX12	3
BP	GO:0051953	negative regulation of amine transport	3/132	28/18870	0.00096443	0.00353019	0.00161375	TNF/DRD2/ADORA1	3
BP	GO:0060512	prostate gland morphogenesis	3/132	28/18870	0.00096443	0.00353019	0.00161375	AR/MMP2/ESR1	3
BP	GO:0060575	intestinal epithelial cell differentiation	3/132	28/18870	0.00096443	0.00353019	0.00161375	CDKN1A/SRC/TYMS	3
BP	GO:0070423	nucleotide-binding oligomerization domain containing signaling pathway	3/132	28/18870	0.00096443	0.00353019	0.00161375	RELA/NFKBIA/XIAP	3
BP	GO:1903076	regulation of protein localization to plasma membrane	5/132	108/18870	0.00097285	0.00355781	0.00162638	AR/TNF/EGFR/PIK3R1/AKT1	5
BP	GO:0002931	response to ischemia	4/132	63/18870	0.00099024	0.00360852	0.00164955	RELA/BCL2/TP53/CAV1	4
BP	GO:0040014	regulation of multicellular organism growth	4/132	63/18870	0.00099024	0.00360852	0.00164955	BCL2/DRD2/APP/STAT3	4
BP	GO:0043470	regulation of carbohydrate catabolic process	4/132	63/18870	0.00099024	0.00360852	0.00164955	TP53/APP/STAT3/INSR	4
BP	GO:0140895	cell surface toll-like receptor signaling pathway	4/132	63/18870	0.00099024	0.00360852	0.00164955	RELA/NFKBIA/PIK3R1/NR1H3	4
BP	GO:0050905	neuromuscular process	6/132	163/18870	0.00101273	0.00368512	0.00168457	HSP90AA1/TNF/DRD2/APP/EDNRA/ADORA2A	6

BP	GO:0006766	vitamin metabolic process	5/132	109/18870	0.00101397	0.00368512	0.00168457	TNF/AKR1B1/AKR1C3/AKR1A1/DHFR	5
BP	GO:0006939	smooth muscle contraction	5/132	109/18870	0.00101397	0.00368512	0.00168457	DRD2/EDN1/EDNRA/CAV1/PTGS2	5
BP	GO:0019229	regulation of vasoconstriction	4/132	64/18870	0.00105059	0.00380805	0.00174077	EDN1/CAV1/MMP2/PTGS2	4
BP	GO:0021885	forebrain cell migration	4/132	64/18870	0.00105059	0.00380805	0.00174077	DRD2/COL3A1/EGFR/AXL	4
BP	GO:0048247	lymphocyte chemotaxis	4/132	64/18870	0.00105059	0.00380805	0.00174077	CCL2/CXCL11/CXCL10/PIK3CG	4
BP	GO:0014013	regulation of gliogenesis	5/132	110/18870	0.00105638	0.00381547	0.00174416	RELA/TP53/TNF/RB1/F2	5
BP	GO:0048477	oogenesis	5/132	110/18870	0.00105638	0.00381547	0.00174416	BCL2/EDN1/EDNRA/SRC/AKT1	5
BP	GO:0090263	positive regulation of canonical Wnt signaling pathway	5/132	110/18870	0.00105638	0.00381547	0.00174416	XIAP/CAV1/COL1A1/EGFR/SRC	5
BP	GO:1903557	positive regulation of tumor necrosis factor superfamily cytokine productic	5/132	110/18870	0.00105638	0.00381547	0.00174416	APP/STAT3/SYK/PIK3R1/PTPN11	5
BP	GO:0007588	excretion	3/132	29/18870	0.00107024	0.00384625	0.00175823	DRD2/EDN1/ABCG2	3
BP	GO:0035872	nucleotide-binding domain, leucine rich repeat containing receptor signal	3/132	29/18870	0.00107024	0.00384625	0.00175823	RELA/NFKBIA/XIAP	3
BP	GO:0060603	mammary gland duct morphogenesis	3/132	29/18870	0.00107024	0.00384625	0.00175823	AR/SRC/ESR1	3
BP	GO:0072012	glomerulus vasculature development	3/132	29/18870	0.00107024	0.00384625	0.00175823	IL6R/EDNRA/ITGB3	3
BP	GO:0097421	liver regeneration	3/132	29/18870	0.00107024	0.00384625	0.00175823	TNF/CCND1/TYMS	3
BP	GO:0033157	regulation of intracellular protein transport	7/132	226/18870	0.00107055	0.00384625	0.00175823	HSP90AA1/CDK1/ADIPOQ/GSK3B/PIK3R1/PTPN11/PT	7
BP	GO:0001938	positive regulation of endothelial cell proliferation	5/132	111/18870	0.00110008	0.00394196	0.00180198	STAT3/ITGB3/KDR/AKT1/F3	5
BP	GO:0002793	positive regulation of peptide secretion	5/132	111/18870	0.00110008	0.00394196	0.00180198	BAD/DRD2/ADORA1/F2/CFTR	5
BP	GO:0071347	cellular response to interleukin-1	5/132	111/18870	0.00110008	0.00394196	0.00180198	RELA/NFKBIA/EDN1/CCL2/MMP2	5
BP	GO:0070665	positive regulation of leukocyte proliferation	6/132	166/18870	0.0011127	0.00397601	0.00181755	BCL2/CDKN1A/IL2RA/SYK/PTK2/IL2	6
BP	GO:0010812	negative regulation of cell-substrate adhesion	4/132	65/18870	0.00111348	0.00397601	0.00181755	COL1A1/MMP12/PIK3R1/SRC	4
BP	GO:0030199	collagen fibril organization	4/132	65/18870	0.00111348	0.00397601	0.00181755	RB1/COL1A1/COL3A1/CYP1B1	4
BP	GO:0030888	regulation of B cell proliferation	4/132	65/18870	0.00111348	0.00397601	0.00181755	BCL2/CDKN1A/IL2/AHR	4
BP	GO:0007179	transforming growth factor beta receptor signaling pathway	7/132	229/18870	0.00115546	0.00412232	0.00188443	FOS/TP53/STAT3/COL3A1/PARP1/SRC/PTK2	7
BP	GO:0015800	acidic amino acid transport	4/132	66/18870	0.00117896	0.00419881	0.0019194	TNF/GJA1/ADORA1/ADORA2A	4
BP	GO:0042531	positive regulation of tyrosine phosphorylation of STAT protein	4/132	66/18870	0.00117896	0.00419881	0.0019194	TNF/IL6R/FLT3/IL2	4
BP	GO:0008209	androgen metabolic process	3/132	30/18870	0.0011831	0.00420259	0.00192112	CYP19A1/SPP1/ESR1	3
BP	GO:0010575	positive regulation of vascular endothelial growth factor production	3/132	30/18870	0.0011831	0.00420259	0.00192112	RELA/CYP1B1/PTGS2	3
BP	GO:0071295	cellular response to vitamin	3/132	30/18870	0.0011831	0.00420259	0.00192112	RXRA/COL1A1/PIM1	3
BP	GO:0051403	stress-activated MAPK cascade	7/132	230/18870	0.00118491	0.00420536	0.00192239	TNF/MYC/APP/XIAP/XDH/IGF1R/EGFR	7
BP	GO:0042177	negative regulation of protein catabolic process	5/132	113/18870	0.0011915	0.0042214	0.00192972	RELA/TIMP1/EGFR/HMGCR/CYP51A1	5
BP	GO:2000060	positive regulation of ubiquitin-dependent protein catabolic process	5/132	113/18870	0.0011915	0.0042214	0.00192972	CAV1/GSK3B/PTK2/PLK1/AKT1	5
BP	GO:0051250	negative regulation of lymphocyte activation	6/132	169/18870	0.00122008	0.0043189	0.00197429	IL2RA/IL4R/ARG1/ADORA2A/AXL/IL2	6
BP	GO:0022037	metencephalon development	5/132	114/18870	0.00123926	0.00437544	0.00200014	SCN5A/BCL2/TP53/IGF1R/PTPN11	5
BP	GO:0034440	lipid oxidation	5/132	114/18870	0.00123926	0.00437544	0.00200014	ADIPOQ/ALOX5/ALOX15/ALOX12/AKT1	5
BP	GO:2001243	negative regulation of intrinsic apoptotic signaling pathway	5/132	114/18870	0.00123926	0.00437544	0.00200014	BCL2/MMP9/SRC/AKT1/PTGS2	5
BP	GO:0001655	urogenital system development	4/132	67/18870	0.00124709	0.00439168	0.00200756	AR/CYP19A1/MMP2/ESR1	4
BP	GO:0015909	long-chain fatty acid transport	4/132	67/18870	0.00124709	0.00439168	0.00200756	DRD2/SYK/AKT1/PLA2G1B	4
BP	GO:0051057	positive regulation of small GTPase mediated signal transduction	4/132	67/18870	0.00124709	0.00439168	0.00200756	COL3A1/SRC/GPR35/PIK3CG	4
BP	GO:0002366	leukocyte activation involved in immune response	8/132	301/18870	0.00127477	0.00448529	0.00205035	TP53/TNF/IL6R/IL4R/STAT3/SYK/IL2/PIK3CG	8
BP	GO:0043473	pigmentation	5/132	115/18870	0.00128843	0.00452945	0.00207054	BCL2/BAX/DRD2/EDNRA/TYR	5
BP	GO:0005979	regulation of glycogen biosynthetic process	3/132	31/18870	0.0013032	0.0045423	0.00207642	GSK3B/AKT1/INSR	3
BP	GO:0010962	regulation of glucan biosynthetic process	3/132	31/18870	0.0013032	0.0045423	0.00207642	GSK3B/AKT1/INSR	3
BP	GO:0033688	regulation of osteoblast proliferation	3/132	31/18870	0.0013032	0.0045423	0.00207642	BCL2/ITGB3/IGF1R	3
BP	GO:0050482	arachidonic acid secretion	3/132	31/18870	0.0013032	0.0045423	0.00207642	DRD2/SYK/PLA2G1B	3
BP	GO:0060325	face morphogenesis	3/132	31/18870	0.0013032	0.0045423	0.00207642	COL1A1/MMP2/PTPN11	3
BP	GO:0061437	renal system vasculature development	3/132	31/18870	0.0013032	0.0045423	0.00207642	IL6R/EDNRA/ITGB3	3
BP	GO:0061440	kidney vasculature development	3/132	31/18870	0.0013032	0.0045423	0.00207642	IL6R/EDNRA/ITGB3	3
BP	GO:0090322	regulation of superoxide metabolic process	3/132	31/18870	0.0013032	0.0045423	0.00207642	SYK/MAPT/DHFR	3
BP	GO:1903963	arachidonate transport	3/132	31/18870	0.0013032	0.0045423	0.00207642	DRD2/SYK/PLA2G1B	3

BP	GO:2000191	regulation of fatty acid transport	3/132	31/18870	0.0013032	0.0045423	0.00207642	EDN1/SYK/AKT1	3
BP	GO:0051926	negative regulation of calcium ion transport	4/132	68/18870	0.00131793	0.0045858	0.0020963	BCL2/DRD2/GPR35/PTGS2	4
BP	GO:0150116	regulation of cell-substrate junction organization	4/132	68/18870	0.00131793	0.0045858	0.0020963	PIK3R1/SRC/PTK2/KDR	4
BP	GO:0045582	positive regulation of T cell differentiation	5/132	116/18870	0.00133902	0.00465126	0.00212622	BAD/IL2RA/IL4R/SYK/IL2	5
BP	GO:0046634	regulation of alpha-beta T cell activation	5/132	116/18870	0.00133902	0.00465126	0.00212622	IL2RA/IL4R/SYK/ADORA2A/IL2	5
BP	GO:1903050	regulation of proteolysis involved in protein catabolic process	7/132	235/18870	0.00134112	0.00465461	0.00212775	CDK2/CAV1/GSK3B/PTK2/PLK1/CSNK2A1/AKT1	7
BP	GO:0002262	myeloid cell homeostasis	6/132	173/18870	0.00137538	0.00476947	0.00218026	BAX/RB1/STAT3/STAT1/AXL/CDK6	6
BP	GO:0051146	striated muscle cell differentiation	8/132	305/18870	0.00138604	0.00480234	0.00219528	BCL2/CDK1/RB1/IL4R/EDN1/CXCL10/NOX4/AKT1	8
BP	GO:0032008	positive regulation of TOR signaling	4/132	69/18870	0.00139153	0.00480914	0.00219839	SYK/PIM1/SRC/AKT1	4
BP	GO:0046626	regulation of insulin receptor signaling pathway	4/132	69/18870	0.00139153	0.00480914	0.00219839	RELA/SRC/PTPN11/CTSD	4
BP	GO:0050918	positive chemotaxis	4/132	69/18870	0.00139153	0.00480914	0.00219839	CXCL10/KDR/MET/F3	4
BP	GO:0001958	endochondral ossification	3/132	32/18870	0.00143071	0.00491956	0.00224887	COL1A1/RUNX2/MMP13	3
BP	GO:0002431	Fc receptor mediated stimulatory signaling pathway	3/132	32/18870	0.00143071	0.00491956	0.00224887	SYK/SRC/PTK2	3
BP	GO:0010464	regulation of mesenchymal cell proliferation	3/132	32/18870	0.00143071	0.00491956	0.00224887	MYC/STAT1/KDR	3
BP	GO:0034694	response to prostaglandin	3/132	32/18870	0.00143071	0.00491956	0.00224887	EDN1/AKT1/AKR1C3	3
BP	GO:0036075	replacement ossification	3/132	32/18870	0.00143071	0.00491956	0.00224887	COL1A1/RUNX2/MMP13	3
BP	GO:0099171	presynaptic modulation of chemical synaptic transmission	3/132	32/18870	0.00143071	0.00491956	0.00224887	DRD2/GSK3B/ADORA2A	3
BP	GO:0031098	stress-activated protein kinase signaling cascade	7/132	238/18870	0.0014423	0.0049511	0.00226329	TNF/MYC/APP/XIAP/XDH/IGF1R/EGFR	7
BP	GO:0050670	regulation of lymphocyte proliferation	7/132	238/18870	0.0014423	0.0049511	0.00226329	BCL2/CDKN1A/IL2RA/SYK/ARG1/IL2/AHR	7
BP	GO:1902905	positive regulation of supramolecular fiber organization	6/132	175/18870	0.00145848	0.00500243	0.00228675	RB1/APP/EDN1/ALOX15/MET/MAPT	6
BP	GO:0002752	cell surface pattern recognition receptor signaling pathway	4/132	70/18870	0.00146796	0.0050307	0.00229968	RELA/NFKBIA/PIK3R1/NR1H3	4
BP	GO:0048588	developmental cell growth	7/132	239/18870	0.00147732	0.00505856	0.00231241	HSP90AA1/APP/EDN1/EDNRA/SPP1/GSK3B/MAPT	7
BP	GO:0035710	CD4-positive, alpha-beta T cell activation	5/132	119/18870	0.0014996	0.00512624	0.00234335	IL6R/IL2RA/IL4R/STAT3/IL2	5
BP	GO:1904892	regulation of receptor signaling pathway via STAT	5/132	119/18870	0.0014996	0.00512624	0.00234335	TNF/IL6R/CAV1/CYP1B1/F2	5
BP	GO:0022900	electron transport chain	6/132	176/18870	0.00150144	0.00512824	0.00234426	CDK1/CCNB1/CYCS/CYP19A1/NOX4/AKR1B1	6
BP	GO:0001764	neuron migration	6/132	177/18870	0.00154536	0.00527152	0.00240976	HSP90AA1/BAX/DRD2/STAT3/COL3A1/AXL	6
BP	GO:0045600	positive regulation of fat cell differentiation	4/132	71/18870	0.00154726	0.00527152	0.00240976	SULT1E1/PIM1/AKT1/PTGS2	4
BP	GO:1900076	regulation of cellular response to insulin stimulus	4/132	71/18870	0.00154726	0.00527152	0.00240976	RELA/SRC/PTPN11/CTSD	4
BP	GO:0002274	myeloid leukocyte activation	7/132	241/18870	0.00154936	0.00527427	0.00241102	TNF/APP/IL4R/SYK/NR1H3/MAPT/PIK3CG	7
BP	GO:0072009	nephron epithelium development	5/132	120/18870	0.00155615	0.00529298	0.00241957	BCL2/ADIPOQ/MYC/EDNRA/STAT1	5
BP	GO:0036336	dendritic cell migration	3/132	33/18870	0.00156578	0.00530365	0.00242445	ALOX5/CXCR1/PIK3CG	3
BP	GO:0038094	Fc-gamma receptor signaling pathway	3/132	33/18870	0.00156578	0.00530365	0.00242445	SYK/SRC/PTK2	3
BP	GO:0045577	regulation of B cell differentiation	3/132	33/18870	0.00156578	0.00530365	0.00242445	BAD/SYK/IL2	3
BP	GO:0061436	establishment of skin barrier	3/132	33/18870	0.00156578	0.00530365	0.00242445	CLDN4/ALOX12/MET	3
BP	GO:0072539	T-helper 17 cell differentiation	3/132	33/18870	0.00156578	0.00530365	0.00242445	IL6R/STAT3/IL2	3
BP	GO:0032944	regulation of mononuclear cell proliferation	7/132	242/18870	0.00158639	0.005369	0.00245432	BCL2/CDKN1A/IL2RA/SYK/ARG1/IL2/AHR	7
BP	GO:0030278	regulation of ossification	5/132	121/18870	0.00161425	0.00545427	0.0024933	RXRA/BCL2/RUNX2/ALOX5/PTPN11	5
BP	GO:0048675	axon extension	5/132	121/18870	0.00161425	0.00545427	0.0024933	HSP90AA1/EDN1/EDNRA/GSK3B/MAPT	5
BP	GO:1903320	regulation of protein modification by small protein conjugation or remova	7/132	243/18870	0.0016241	0.00548302	0.00250644	HSP90AA1/RELA/XIAP/CAV1/GSK3B/PLK1/AKT1	7
BP	GO:0070527	platelet aggregation	4/132	72/18870	0.0016295	0.00549669	0.00251269	ITGB3/SYK/ALOX12/PIK3CG	4
BP	GO:0006469	negative regulation of protein kinase activity	6/132	179/18870	0.00163612	0.00551448	0.00252082	ADIPOQ/CDKN1A/RB1/CAV1/PLK1/AKT1	6
BP	GO:0030518	intracellular steroid hormone receptor signaling pathway	5/132	122/18870	0.00167393	0.00563728	0.00257696	AR/PARP1/SRC/ESR1/ESR2	5
BP	GO:0090183	regulation of kidney development	3/132	34/18870	0.00170858	0.00574152	0.00262461	ADIPOQ/MYC/STAT1	3
BP	GO:0051656	establishment of organelle localization	10/132	469/18870	0.00171453	0.00574152	0.00262461	CCNB1/RB1/BIRC5/IL4R/SLC2A4/GJA1/SYK/PLK1/MAP	10
BP	GO:0032413	negative regulation of ion transmembrane transporter activity	4/132	73/18870	0.00171473	0.00574152	0.00262461	DRD2/CAV1/MMP9/GPR35	4
BP	GO:0032732	positive regulation of interleukin-1 production	4/132	73/18870	0.00171473	0.00574152	0.00262461	RELA/TNF/APP/STAT3	4
BP	GO:0042093	T-helper cell differentiation	4/132	73/18870	0.00171473	0.00574152	0.00262461	IL6R/IL4R/STAT3/IL2	4
BP	GO:0071677	positive regulation of mononuclear cell migration	4/132	73/18870	0.00171473	0.00574152	0.00262461	TNF/APP/ITGB3/CXCL10	4
BP	GO:1904377	positive regulation of protein localization to cell periphery	4/132	73/18870	0.00171473	0.00574152	0.00262461	TNF/EGFR/PIK3R1/AKT1	4

BP	GO:0060070	canonical Wnt signaling pathway	8/132	316/18870	0.00173231	0.00578644	0.00264514	XIAP/EDN1/EDNRA/CAV1/COL1A1/GSK3B/EGFR/SRC	8
BP	GO:0002286	T cell activation involved in immune response	5/132	123/18870	0.00173522	0.00578644	0.00264514	TP53/IL6R/IL4R/STAT3/IL2	5
BP	GO:0007613	memory	5/132	123/18870	0.00173522	0.00578644	0.00264514	DRD2/SLC2A4/MAPT/INSR/PTGS2	5
BP	GO:0010811	positive regulation of cell-substrate adhesion	5/132	123/18870	0.00173522	0.00578644	0.00264514	ITGB3/GSK3B/KDR/ALOX15/CDK6	5
BP	GO:0030282	bone mineralization	5/132	123/18870	0.00173522	0.00578644	0.00264514	RXRA/ALOX5/MMP13/ALOX15/PTGS2	5
BP	GO:0045926	negative regulation of growth	7/132	247/18870	0.00178201	0.00593276	0.00271203	BCL2/TP53/CDKN1A/RB1/GJA1/SPP1/ESR2	7
BP	GO:0048738	cardiac muscle tissue development	7/132	247/18870	0.00178201	0.00593276	0.00271203	SCN5A/CDK1/EDN1/EDNRA/GJA1/NOX4/PIM1	7
BP	GO:0045446	endothelial cell differentiation	5/132	124/18870	0.00179814	0.00598161	0.00273436	TNF/EDNRA/XDH/KDR/MET	5
BP	GO:0045123	cellular extravasation	4/132	74/18870	0.001803	0.0059929	0.00273952	TNF/SELE/CCL2/PIK3CG	4
BP	GO:0002719	negative regulation of cytokine production involved in immune response	3/132	35/18870	0.00185927	0.00613994	0.00280674	TNF/ARG1/AXL	3
BP	GO:0009112	nucleobase metabolic process	3/132	35/18870	0.00185927	0.00613994	0.00280674	XDH/TTR/TYMS	3
BP	GO:0033146	regulation of intracellular estrogen receptor signaling pathway	3/132	35/18870	0.00185927	0.00613994	0.00280674	AR/PARP1/SRC	3
BP	GO:0051968	positive regulation of synaptic transmission, glutamatergic	3/132	35/18870	0.00185927	0.00613994	0.00280674	CCL2/ADORA2A/PTGS2	3
BP	GO:0086004	regulation of cardiac muscle cell contraction	3/132	35/18870	0.00185927	0.00613994	0.00280674	SCN5A/CAV1/ADORA1	3
BP	GO:0086005	ventricular cardiac muscle cell action potential	3/132	35/18870	0.00185927	0.00613994	0.00280674	KCNH2/SCN5A/CAV1	3
BP	GO:1902253	regulation of intrinsic apoptotic signaling pathway by p53 class mediator	3/132	35/18870	0.00185927	0.00613994	0.00280674	BCL2/TP53/MYC	3
BP	GO:2000406	positive regulation of T cell migration	3/132	35/18870	0.00185927	0.00613994	0.00280674	APP/ITGB3/CXCL10	3
BP	GO:0002294	CD4-positive, alpha-beta T cell differentiation involved in immune respon	4/132	75/18870	0.00189439	0.00624581	0.00285514	IL6R/IL4R/STAT3/IL2	4
BP	GO:0045806	negative regulation of endocytosis	4/132	75/18870	0.00189439	0.00624581	0.00285514	ADIPOQ/ITGB3/CAV1/NR1H3	4
BP	GO:0045766	positive regulation of angiogenesis	6/132	185/18870	0.00193287	0.00636242	0.00290844	STAT3/ITGB3/CYP1B1/KDR/TERT/F3	6
BP	GO:1904018	positive regulation of vasculature development	6/132	185/18870	0.00193287	0.00636242	0.00290844	STAT3/ITGB3/CYP1B1/KDR/TERT/F3	6
BP	GO:0002287	alpha-beta T cell activation involved in immune response	4/132	76/18870	0.00198893	0.00653116	0.00298558	IL6R/IL4R/STAT3/IL2	4
BP	GO:0002293	alpha-beta T cell differentiation involved in immune response	4/132	76/18870	0.00198893	0.00653116	0.00298558	IL6R/IL4R/STAT3/IL2	4
BP	GO:0086001	cardiac muscle cell action potential	4/132	76/18870	0.00198893	0.00653116	0.00298558	KCNH2/SCN5A/CAV1/GJA1	4
BP	GO:0001569	branching involved in blood vessel morphogenesis	3/132	36/18870	0.00201799	0.00658427	0.00300985	EDN1/EDNRA/KDR	3
BP	GO:0033687	osteoblast proliferation	3/132	36/18870	0.00201799	0.00658427	0.00300985	BCL2/ITGB3/IGF1R	3
BP	GO:0046164	alcohol catabolic process	3/132	36/18870	0.00201799	0.00658427	0.00300985	SULT1E1/AKR1B10/AKR1C3	3
BP	GO:0055023	positive regulation of cardiac muscle tissue growth	3/132	36/18870	0.00201799	0.00658427	0.00300985	CDK1/EDN1/PIM1	3
BP	GO:0060218	hematopoietic stem cell differentiation	3/132	36/18870	0.00201799	0.00658427	0.00300985	BCL2/TP53/CDK6	3
BP	GO:1902745	positive regulation of lamellipodium organization	3/132	36/18870	0.00201799	0.00658427	0.00300985	HSP90AA1/PIK3R1/SRC	3
BP	GO:1905898	positive regulation of response to endoplasmic reticulum stress	3/132	36/18870	0.00201799	0.00658427	0.00300985	BAX/CAV1/PIK3R1	3
BP	GO:2000352	negative regulation of endothelial cell apoptotic process	3/132	36/18870	0.00201799	0.00658427	0.00300985	ITGB3/KDR/TERT	3
BP	GO:0032200	telomere organization	6/132	187/18870	0.00204032	0.00665179	0.00304072	HSP90AA1/MYC/PARP1/SRC/TERT/APEX1	6
BP	GO:0035304	regulation of protein dephosphorylation	4/132	77/18870	0.00208668	0.00673865	0.00308043	TNF/ADORA1/GSK3B/NUAK1	4
BP	GO:0050853	B cell receptor signaling pathway	4/132	77/18870	0.00208668	0.00673865	0.00308043	BCL2/BAX/NFKBIA/SYK	4
BP	GO:0002676	regulation of chronic inflammatory response	2/132	10/18870	0.00210655	0.00673865	0.00308043	CYP19A1/TNF	2
BP	GO:0003253	cardiac neural crest cell migration involved in outflow tract morphogenesis	2/132	10/18870	0.00210655	0.00673865	0.00308043	EDN1/EDNRA	2
BP	GO:0008300	isoprenoid catabolic process	2/132	10/18870	0.00210655	0.00673865	0.00308043	AKR1B10/AKR1C3	2
BP	GO:0008627	intrinsic apoptotic signaling pathway in response to osmotic stress	2/132	10/18870	0.00210655	0.00673865	0.00308043	BAD/PTGS2	2
BP	GO:0010918	positive regulation of mitochondrial membrane potential	2/132	10/18870	0.00210655	0.00673865	0.00308043	BAD/AKT1	2
BP	GO:0014820	tonic smooth muscle contraction	2/132	10/18870	0.00210655	0.00673865	0.00308043	EDN1/EDNRA	2
BP	GO:0019852	L-ascorbic acid metabolic process	2/132	10/18870	0.00210655	0.00673865	0.00308043	AKR1B1/AKR1A1	2
BP	GO:0035747	natural killer cell chemotaxis	2/132	10/18870	0.00210655	0.00673865	0.00308043	CCL2/PIK3CG	2
BP	GO:0038063	collagen-activated tyrosine kinase receptor signaling pathway	2/132	10/18870	0.00210655	0.00673865	0.00308043	COL1A1/SYK	2
BP	GO:0048865	stem cell fate commitment	2/132	10/18870	0.00210655	0.00673865	0.00308043	EDN1/EDNRA	2
BP	GO:0060346	bone trabecula formation	2/132	10/18870	0.00210655	0.00673865	0.00308043	COL1A1/MMP2	2
BP	GO:0071372	cellular response to follicle-stimulating hormone stimulus	2/132	10/18870	0.00210655	0.00673865	0.00308043	EDN1/EDNRA	2
BP	GO:0071803	positive regulation of podosome assembly	2/132	10/18870	0.00210655	0.00673865	0.00308043	TNF/SRC	2
BP	GO:0072203	cell proliferation involved in metanephros development	2/132	10/18870	0.00210655	0.00673865	0.00308043	MYC/STAT1	2

BP	GO:1900543	negative regulation of purine nucleotide metabolic process	2/132	10/18870	0.00210655	0.00673865	0.00308043	TP53/PARP1	2
BP	GO:1901334	lactone metabolic process	2/132	10/18870	0.00210655	0.00673865	0.00308043	AKR1B1/AKR1A1	2
BP	GO:1903799	negative regulation of miRNA processing	2/132	10/18870	0.00210655	0.00673865	0.00308043	TP53/STAT3	2
BP	GO:1903943	regulation of hepatocyte apoptotic process	2/132	10/18870	0.00210655	0.00673865	0.00308043	RB1/IGF1R	2
BP	GO:1904627	response to phorbol 13-acetate 12-myristate	2/132	10/18870	0.00210655	0.00673865	0.00308043	FOS/CDK4	2
BP	GO:1904628	cellular response to phorbol 13-acetate 12-myristate	2/132	10/18870	0.00210655	0.00673865	0.00308043	FOS/CDK4	2
BP	GO:1904760	regulation of myofibroblast differentiation	2/132	10/18870	0.00210655	0.00673865	0.00308043	RB1/PARP1	2
BP	GO:1905050	positive regulation of metallopeptidase activity	2/132	10/18870	0.00210655	0.00673865	0.00308043	STAT3/CLDN4	2
BP	GO:0060485	mesenchyme development	8/132	327/18870	0.0021438	0.00685245	0.00313245	BCL2/MYC/EDN1/EDNRA/STAT1/COL1A1/GSK3B/PTK2	8
BP	GO:0046890	regulation of lipid biosynthetic process	6/132	189/18870	0.00215223	0.006874	0.0031423	TNF/IGF1R/AKT1/AKR1C3/NR1H3/PTGS2	6
BP	GO:0007095	mitotic G2 DNA damage checkpoint signaling	3/132	37/18870	0.00218491	0.00696204	0.00318255	CDK1/CDKN1A/PLK1	3
BP	GO:0043243	positive regulation of protein-containing complex disassembly	3/132	37/18870	0.00218491	0.00696204	0.00318255	TNF/IGF1R/INSR	3
BP	GO:0060323	head morphogenesis	3/132	37/18870	0.00218491	0.00696204	0.00318255	COL1A1/MMP2/PTPN11	3
BP	GO:0006839	mitochondrial transport	6/132	190/18870	0.00220989	0.00703617	0.00321643	HSP90AA1/BCL2/BAX/TP53/BAD/GSK3B	6
BP	GO:0002700	regulation of production of molecular mediator of immune response	6/132	191/18870	0.00226871	0.00721783	0.00329948	TNF/IL4R/SYK/ARG1/AXL/IL2	6
BP	GO:0007212	dopamine receptor signaling pathway	3/132	38/18870	0.00236015	0.0074738	0.00341648	DRD2/GSK3B/ALK	3
BP	GO:0010737	protein kinase A signaling	3/132	38/18870	0.00236015	0.0074738	0.00341648	ADIPOQ/EDN1/EDNRA	3
BP	GO:0045740	positive regulation of DNA replication	3/132	38/18870	0.00236015	0.0074738	0.00341648	CDK1/CDK2/EGFR	3
BP	GO:0060306	regulation of membrane repolarization	3/132	38/18870	0.00236015	0.0074738	0.00341648	KCNH2/SCN5A/CAV1	3
BP	GO:0070873	regulation of glycogen metabolic process	3/132	38/18870	0.00236015	0.0074738	0.00341648	GSK3B/AKT1/INSR	3
BP	GO:0086091	regulation of heart rate by cardiac conduction	3/132	38/18870	0.00236015	0.0074738	0.00341648	KCNH2/SCN5A/CAV1	3
BP	GO:0046328	regulation of JNK cascade	5/132	132/18870	0.00236333	0.00747807	0.00341844	TNF/APP/XIAP/IGF1R/EGFR	5
BP	GO:0009749	response to glucose	6/132	193/18870	0.0023899	0.00755629	0.00345419	BAD/ADIPOQ/KLF7/NOX4/IGF1R/CFTR	6
BP	GO:0007264	small GTPase mediated signal transduction	10/132	491/18870	0.00239423	0.00756413	0.00345778	TP53/CDK2/CDKN1A/RB1/COL3A1/RASSF1/SRC/MET/	10
BP	GO:0002437	inflammatory response to antigenic stimulus	4/132	80/18870	0.00239979	0.00757583	0.00346313	TNF/IL2RA/SYK/SRC	4
BP	GO:0006720	isoprenoid metabolic process	5/132	133/18870	0.0024421	0.00768564	0.00351332	AKR1B1/CYP1B1/AKR1B10/AKR1C3/HMGCR	5
BP	GO:0019730	antimicrobial humoral response	5/132	133/18870	0.0024421	0.00768564	0.00351332	CXCL11/CXCL2/CXCL10/F2/PLA2G1B	5
BP	GO:0030048	actin filament-based movement	5/132	133/18870	0.0024421	0.00768564	0.00351332	KCNH2/SCN5A/CAV1/GJA1/ADORA1	5
BP	GO:0050868	negative regulation of T cell activation	5/132	133/18870	0.0024421	0.00768564	0.00351332	IL2RA/IL4R/ARG1/ADORA2A/IL2	5
BP	GO:0048762	mesenchymal cell differentiation	7/132	262/18870	0.00248301	0.00780837	0.00356943	BCL2/EDN1/EDNRA/STAT1/COL1A1/GSK3B/PTK2	7
BP	GO:0002534	cytokine production involved in inflammatory response	4/132	81/18870	0.00251095	0.00786584	0.0035957	TNF/STAT3/ALOX5/F2	4
BP	GO:0010833	telomere maintenance via telomere lengthening	4/132	81/18870	0.00251095	0.00786584	0.0035957	HSP90AA1/PARP1/SRC/TERT	4
BP	GO:0045913	positive regulation of carbohydrate metabolic process	4/132	81/18870	0.00251095	0.00786584	0.0035957	APP/SRC/AKT1/INSR	4
BP	GO:1900015	regulation of cytokine production involved in inflammatory response	4/132	81/18870	0.00251095	0.00786584	0.0035957	TNF/STAT3/ALOX5/F2	4
BP	GO:0006639	acylglycerol metabolic process	5/132	134/18870	0.00252276	0.00786584	0.0035957	MTTP/CAV1/PTPN11/NR1H3/PIK3CG	5
BP	GO:0032386	regulation of intracellular transport	8/132	336/18870	0.0025347	0.00786584	0.0035957	HSP90AA1/CDK1/ADIPOQ/GSK3B/PIK3R1/SRC/PTPN1	8
BP	GO:0010762	regulation of fibroblast migration	3/132	39/18870	0.00254386	0.00786584	0.0035957	ITGB3/PTK2/AKT1	3
BP	GO:0014037	Schwann cell differentiation	3/132	39/18870	0.00254386	0.00786584	0.0035957	RELA/CDK1/AKT1	3
BP	GO:0032885	regulation of polysaccharide biosynthetic process	3/132	39/18870	0.00254386	0.00786584	0.0035957	GSK3B/AKT1/INSR	3
BP	GO:0071548	response to dexamethasone	3/132	39/18870	0.00254386	0.00786584	0.0035957	EDN1/CYP1B1/IGF1R	3
BP	GO:0001660	fever generation	2/132	11/18870	0.0025629	0.00786584	0.0035957	TNF/PTGS2	2
BP	GO:0003357	noradrenergic neuron differentiation	2/132	11/18870	0.0025629	0.00786584	0.0035957	EDN1/EDNRA	2
BP	GO:0006983	ER overload response	2/132	11/18870	0.0025629	0.00786584	0.0035957	TP53/GSK3B	2
BP	GO:0031953	negative regulation of protein autophosphorylation	2/132	11/18870	0.0025629	0.00786584	0.0035957	ADIPOQ/CAV1	2
BP	GO:0033148	positive regulation of intracellular estrogen receptor signaling pathway	2/132	11/18870	0.0025629	0.00786584	0.0035957	AR/PARP1	2
BP	GO:0034139	regulation of toll-like receptor 3 signaling pathway	2/132	11/18870	0.0025629	0.00786584	0.0035957	CAV1/SRC	2
BP	GO:0035812	renal sodium excretion	2/132	11/18870	0.0025629	0.00786584	0.0035957	DRD2/EDN1	2
BP	GO:0042045	epithelial fluid transport	2/132	11/18870	0.0025629	0.00786584	0.0035957	EDN1/CFTR	2
BP	GO:0044062	regulation of excretion	2/132	11/18870	0.0025629	0.00786584	0.0035957	DRD2/EDN1	2

BP	GO:0045793	positive regulation of cell size	2/132	11/18870	0.0025629	0.00786584	0.0035957	HSP90AA1/EDN1	2
BP	GO:0045980	negative regulation of nucleotide metabolic process	2/132	11/18870	0.0025629	0.00786584	0.0035957	TP53/PARP1	2
BP	GO:0048563	post-embryonic animal organ morphogenesis	2/132	11/18870	0.0025629	0.00786584	0.0035957	BAX/KDR	2
BP	GO:0051709	regulation of killing of cells of another organism	2/132	11/18870	0.0025629	0.00786584	0.0035957	SYK/ARG1	2
BP	GO:0051987	positive regulation of attachment of spindle microtubules to kinetochore	2/132	11/18870	0.0025629	0.00786584	0.0035957	CCNB1/BIRC5	2
BP	GO:0060159	regulation of dopamine receptor signaling pathway	2/132	11/18870	0.0025629	0.00786584	0.0035957	DRD2/ALK	2
BP	GO:0061370	testosterone biosynthetic process	2/132	11/18870	0.0025629	0.00786584	0.0035957	CYP19A1/AKR1C3	2
BP	GO:0062100	positive regulation of programmed necrotic cell death	2/132	11/18870	0.0025629	0.00786584	0.0035957	TP53/PARP1	2
BP	GO:0072124	regulation of glomerular mesangial cell proliferation	2/132	11/18870	0.0025629	0.00786584	0.0035957	IL6R/ITGB3	2
BP	GO:0072584	caveolin-mediated endocytosis	2/132	11/18870	0.0025629	0.00786584	0.0035957	CAV1/SRC	2
BP	GO:0086100	endothelin receptor signaling pathway	2/132	11/18870	0.0025629	0.00786584	0.0035957	EDN1/EDNRA	2
BP	GO:1904181	positive regulation of membrane depolarization	2/132	11/18870	0.0025629	0.00786584	0.0035957	PARP1/KDR	2
BP	GO:2000833	positive regulation of steroid hormone secretion	2/132	11/18870	0.0025629	0.00786584	0.0035957	CYP19A1/SPP1	2
BP	GO:0043123	positive regulation of canonical NF-kappaB signal transduction	6/132	196/18870	0.00258073	0.00791065	0.00361618	RELA/ADIPOQ/TNF/XIAP/GJA1/PARP1	6
BP	GO:0015980	energy derivation by oxidation of organic compounds	8/132	337/18870	0.00258137	0.00791065	0.00361618	TP53/CDK1/CCNB1/CYCS/TNF/GSK3B/AKT1/INSR	8
BP	GO:0014706	striated muscle tissue development	7/132	264/18870	0.00259055	0.00793284	0.00362633	SCN5A/CDK1/EDN1/EDNRA/GJA1/NOX4/PIM1	7
BP	GO:0006638	neutral lipid metabolic process	5/132	135/18870	0.00260534	0.00797214	0.00364429	MTTP/CAV1/PTPN11/NR1H3/PIK3CG	5
BP	GO:0007369	gastrulation	6/132	197/18870	0.00264682	0.00809302	0.00369955	TP53/ADIPOQ/ITGB3/GJA1/MMP9/MMP2	6
BP	GO:0071333	cellular response to glucose stimulus	5/132	136/18870	0.00268986	0.00821846	0.00375689	BAD/KLF7/NOX4/IGF1R/CFTR	5
BP	GO:0001953	negative regulation of cell-matrix adhesion	3/132	40/18870	0.00273617	0.0083164	0.00380166	MMP12/PIK3R1/SRC	3
BP	GO:0008210	estrogen metabolic process	3/132	40/18870	0.00273617	0.0083164	0.00380166	CYP19A1/SULT1E1/CYP1B1	3
BP	GO:0043368	positive T cell selection	3/132	40/18870	0.00273617	0.0083164	0.00380166	BCL2/IL6R/STAT3	3
BP	GO:0045730	respiratory burst	3/132	40/18870	0.00273617	0.0083164	0.00380166	MPO/INSR/PIK3CG	3
BP	GO:0046676	negative regulation of insulin secretion	3/132	40/18870	0.00273617	0.0083164	0.00380166	KLF7/DRD2/PTPN11	3
BP	GO:0060421	positive regulation of heart growth	3/132	40/18870	0.00273617	0.0083164	0.00380166	CDK1/EDN1/PIM1	3
BP	GO:0062098	regulation of programmed necrotic cell death	3/132	40/18870	0.00273617	0.0083164	0.00380166	TP53/CAV1/PARP1	3
BP	GO:0014855	striated muscle cell proliferation	4/132	83/18870	0.0027438	0.00832722	0.00380661	FOS/CDK1/PIM1/SRC	4
BP	GO:0050848	regulation of calcium-mediated signaling	4/132	83/18870	0.0027438	0.00832722	0.00380661	TNF/SYK/GSK3B/MAPT	4
BP	GO:0042692	muscle cell differentiation	9/132	419/18870	0.00276689	0.00839105	0.00383579	BCL2/CDK1/RB1/IL4R/EDN1/EDNRA/CXCL10/NOX4/A	9
BP	GO:0031396	regulation of protein ubiquitination	6/132	200/18870	0.00285273	0.00864497	0.00395186	HSP90AA1/XIAP/CAV1/GSK3B/PLK1/AKT1	6
BP	GO:0071331	cellular response to hexose stimulus	5/132	138/18870	0.00286485	0.00865826	0.00395794	BAD/KLF7/NOX4/IGF1R/CFTR	5
BP	GO:0001523	retinoid metabolic process	4/132	84/18870	0.0028656	0.00865826	0.00395794	AKR1B1/CYP1B1/AKR1B10/AKR1C3	4
BP	GO:0002292	T cell differentiation involved in immune response	4/132	84/18870	0.0028656	0.00865826	0.00395794	IL6R/IL4R/STAT3/IL2	4
BP	GO:0010660	regulation of muscle cell apoptotic process	4/132	84/18870	0.0028656	0.00865826	0.00395794	TP53/EDN1/IGF1R/ALOX12	4
BP	GO:0045923	positive regulation of fatty acid metabolic process	3/132	41/18870	0.00293722	0.008855	0.00404787	ADIPOQ/NR1H3/PTGS2	3
BP	GO:0060251	regulation of glial cell proliferation	3/132	41/18870	0.00293722	0.008855	0.00404787	TP53/TNF/RB1	3
BP	GO:1903115	regulation of actin filament-based movement	3/132	41/18870	0.00293722	0.008855	0.00404787	SCN5A/CAV1/ADORA1	3
BP	GO:0008277	regulation of G protein-coupled receptor signaling pathway	5/132	139/18870	0.00295536	0.00889658	0.00406688	DRD2/EDN1/ITGB3/F2/MET	5
BP	GO:1903052	positive regulation of proteolysis involved in protein catabolic process	5/132	139/18870	0.00295536	0.00889658	0.00406688	CAV1/GSK3B/PTK2/PLK1/AKT1	5
BP	GO:0008306	associative learning	4/132	85/18870	0.00299104	0.00899074	0.00410992	FOS/DRD2/APP/HMGCR	4
BP	GO:0048041	focal adhesion assembly	4/132	85/18870	0.00299104	0.00899074	0.00410992	BCL2/SRC/PTK2/KDR	4
BP	GO:0034101	erythrocyte homeostasis	5/132	140/18870	0.00304793	0.00904258	0.00413362	RB1/STAT3/STAT1/AXL/CDK6	5
BP	GO:0071326	cellular response to monosaccharide stimulus	5/132	140/18870	0.00304793	0.00904258	0.00413362	BAD/KLF7/NOX4/IGF1R/CFTR	5
BP	GO:0001973	G protein-coupled adenosine receptor signaling pathway	2/132	12/18870	0.00306142	0.00904258	0.00413362	ADORA1/ADORA2A	2
BP	GO:0010917	negative regulation of mitochondrial membrane potential	2/132	12/18870	0.00306142	0.00904258	0.00413362	BAX/MAPT	2
BP	GO:0014854	response to inactivity	2/132	12/18870	0.00306142	0.00904258	0.00413362	SCN5A/DRD2	2
BP	GO:0030656	regulation of vitamin metabolic process	2/132	12/18870	0.00306142	0.00904258	0.00413362	TNF/AKR1C3	2
BP	GO:0031650	regulation of heat generation	2/132	12/18870	0.00306142	0.00904258	0.00413362	TNF/PTGS2	2
BP	GO:0033145	positive regulation of intracellular steroid hormone receptor signaling path	2/132	12/18870	0.00306142	0.00904258	0.00413362	AR/PARP1	2

BP	GO:0033629	negative regulation of cell adhesion mediated by integrin	2/132	12/18870	0.00306142	0.00904258	0.00413362	CYP1B1/PTPN11	2
BP	GO:0035588	G protein-coupled purinergic receptor signaling pathway	2/132	12/18870	0.00306142	0.00904258	0.00413362	ADORA1/ADORA2A	2
BP	GO:0042364	water-soluble vitamin biosynthetic process	2/132	12/18870	0.00306142	0.00904258	0.00413362	AKR1B1/AKR1A1	2
BP	GO:0042754	negative regulation of circadian rhythm	2/132	12/18870	0.00306142	0.00904258	0.00413362	DRD2/ADORA1	2
BP	GO:0045837	negative regulation of membrane potential	2/132	12/18870	0.00306142	0.00904258	0.00413362	BAX/MAPT	2
BP	GO:0060123	regulation of growth hormone secretion	2/132	12/18870	0.00306142	0.00904258	0.00413362	DRD2/PTPN11	2
BP	GO:0060965	negative regulation of miRNA-mediated gene silencing	2/132	12/18870	0.00306142	0.00904258	0.00413362	TP53/STAT3	2
BP	GO:0061309	cardiac neural crest cell development involved in outflow tract morphoger	2/132	12/18870	0.00306142	0.00904258	0.00413362	EDN1/EDNRA	2
BP	GO:0070561	vitamin D receptor signaling pathway	2/132	12/18870	0.00306142	0.00904258	0.00413362	RXRA/PIM1	2
BP	GO:0070943	neutrophil-mediated killing of symbiont cell	2/132	12/18870	0.00306142	0.00904258	0.00413362	ARG1/F2	2
BP	GO:0071389	cellular response to mineralocorticoid stimulus	2/132	12/18870	0.00306142	0.00904258	0.00413362	EDN1/IGF1R	2
BP	GO:0072110	glomerular mesangial cell proliferation	2/132	12/18870	0.00306142	0.00904258	0.00413362	IL6R/ITGB3	2
BP	GO:0097048	dendritic cell apoptotic process	2/132	12/18870	0.00306142	0.00904258	0.00413362	BCL2/AXL	2
BP	GO:0106014	regulation of inflammatory response to wounding	2/132	12/18870	0.00306142	0.00904258	0.00413362	STAT3/ALOX5	2
BP	GO:1990535	neuron projection maintenance	2/132	12/18870	0.00306142	0.00904258	0.00413362	APP/INSR	2
BP	GO:2000668	regulation of dendritic cell apoptotic process	2/132	12/18870	0.00306142	0.00904258	0.00413362	BCL2/AXL	2
BP	GO:2000243	positive regulation of reproductive process	4/132	86/18870	0.00312019	0.00920953	0.00420993	AR/BAX/SRC/INSR	4
BP	GO:0007595	lactation	3/132	42/18870	0.00314713	0.00927562	0.00424015	CCND1/CAV1/XDH	3
BP	GO:2000403	positive regulation of lymphocyte migration	3/132	42/18870	0.00314713	0.00927562	0.00424015	APP/ITGB3/CXCL10	3
BP	GO:0060491	regulation of cell projection assembly	6/132	205/18870	0.0032223	0.00949033	0.0043383	HSP90AA1/CAV1/GSK3B/PIK3R1/SRC/AKT1	6
BP	GO:0001889	liver development	5/132	142/18870	0.00323932	0.00953358	0.00435807	RELA/TNF/CCND1/MET/TYMS	5
BP	GO:0016101	diterpenoid metabolic process	4/132	87/18870	0.0032531	0.00955349	0.00436717	AKR1B1/CYP1B1/AKR1B10/AKR1C3	4
BP	GO:0071868	cellular response to monoamine stimulus	4/132	87/18870	0.0032531	0.00955349	0.00436717	ADIPOQ/DRD2/GSK3B/ALK	4
BP	GO:0071870	cellular response to catecholamine stimulus	4/132	87/18870	0.0032531	0.00955349	0.00436717	ADIPOQ/DRD2/GSK3B/ALK	4
BP	GO:0032434	regulation of proteasomal ubiquitin-dependent protein catabolic process	5/132	143/18870	0.00333819	0.00979634	0.00447818	CDK2/CAV1/GSK3B/PLK1/AKT1	5
BP	GO:0051955	regulation of amino acid transport	3/132	43/18870	0.00336602	0.00987092	0.00451228	TNF/ADORA1/ADORA2A	3
BP	GO:0098586	cellular response to virus	4/132	88/18870	0.00338981	0.00993355	0.00454091	HSP90AA1/BAX/CXCL10/MMP12	4
BP	GO:1990778	protein localization to cell periphery	8/132	353/18870	0.0034239	0.01002625	0.00458328	AR/ADIPOQ/TNF/CAV1/EGFR/PIK3R1/PLK1/AKT1	8
BP	GO:0002819	regulation of adaptive immune response	6/132	208/18870	0.00346054	0.0101263	0.00462901	TNF/IL4R/ARG1/ALOX15/IL2/AHR	6
BP	GO:0015844	monoamine transport	4/132	89/18870	0.00353038	0.01031589	0.00471568	DRD2/ITGB3/SYK/ADORA2A	4
BP	GO:0097194	execution phase of apoptosis	4/132	89/18870	0.00353038	0.01031589	0.00471568	BAX/TP53/AKT1/TOP2A	4
BP	GO:0050671	positive regulation of lymphocyte proliferation	5/132	145/18870	0.00354243	0.01033631	0.00472502	BCL2/CDKN1A/IL2RA/SYK/IL2	5
BP	GO:0061008	hepaticobiliary system development	5/132	145/18870	0.00354243	0.01033631	0.00472502	RELA/TNF/CCND1/MET/TYMS	5
BP	GO:0010463	mesenchymal cell proliferation	3/132	44/18870	0.00359402	0.01035339	0.00473283	MYC/STAT1/KDR	3
BP	GO:0042771	intrinsic apoptotic signaling pathway in response to DNA damage by p53	3/132	44/18870	0.00359402	0.01035339	0.00473283	BCL2/TP53/CDKN1A	3
BP	GO:0046006	regulation of activated T cell proliferation	3/132	44/18870	0.00359402	0.01035339	0.00473283	IL2RA/ARG1/IL2	3
BP	GO:0046640	regulation of alpha-beta T cell proliferation	3/132	44/18870	0.00359402	0.01035339	0.00473283	IL2RA/SYK/IL2	3
BP	GO:0050691	regulation of defense response to virus by host	3/132	44/18870	0.00359402	0.01035339	0.00473283	HSP90AA1/STAT1/MMP12	3
BP	GO:0009615	response to virus	9/132	436/18870	0.00359713	0.01035339	0.00473283	HSP90AA1/RELA/BCL2/BAX/TNF/STAT1/CXCL10/MMF	9
BP	GO:0002069	columnar/cuboidal epithelial cell maturation	2/132	13/18870	0.00360151	0.01035339	0.00473283	CDKN1A/TYMS	2
BP	GO:0033690	positive regulation of osteoblast proliferation	2/132	13/18870	0.00360151	0.01035339	0.00473283	ITGB3/IGF1R	2
BP	GO:0048521	negative regulation of behavior	2/132	13/18870	0.00360151	0.01035339	0.00473283	DRD2/ADORA1	2
BP	GO:0051481	negative regulation of cytosolic calcium ion concentration	2/132	13/18870	0.00360151	0.01035339	0.00473283	BCL2/DRD2	2
BP	GO:0060134	prepulse inhibition	2/132	13/18870	0.00360151	0.01035339	0.00473283	DRD2/ADORA2A	2
BP	GO:0060149	negative regulation of post-transcriptional gene silencing	2/132	13/18870	0.00360151	0.01035339	0.00473283	TP53/STAT3	2
BP	GO:0060967	negative regulation of gene silencing by regulatory ncRNA	2/132	13/18870	0.00360151	0.01035339	0.00473283	TP53/STAT3	2
BP	GO:0061430	bone trabecula morphogenesis	2/132	13/18870	0.00360151	0.01035339	0.00473283	COL1A1/MMP2	2
BP	GO:0070294	renal sodium ion absorption	2/132	13/18870	0.00360151	0.01035339	0.00473283	EDN1/EDNRA	2
BP	GO:0071801	regulation of podosome assembly	2/132	13/18870	0.00360151	0.01035339	0.00473283	TNF/SRC	2

BP	GO:0106049	regulation of cellular response to osmotic stress	2/132	13/18870	0.00360151	0.01035339	0.00473283	BAD/PTGS2	2
BP	GO:1900103	positive regulation of endoplasmic reticulum unfolded protein response	2/132	13/18870	0.00360151	0.01035339	0.00473283	BAX/PIK3R1	2
BP	GO:1900272	negative regulation of long-term synaptic potentiation	2/132	13/18870	0.00360151	0.01035339	0.00473283	APP/ADORA1	2
BP	GO:1900369	negative regulation of post-transcriptional gene silencing by regulatory no	2/132	13/18870	0.00360151	0.01035339	0.00473283	TP53/STAT3	2
BP	GO:1902947	regulation of tau-protein kinase activity	2/132	13/18870	0.00360151	0.01035339	0.00473283	HSP90AA1/RB1	2
BP	GO:0051053	negative regulation of DNA metabolic process	5/132	146/18870	0.00364785	0.01047923	0.00479035	TP53/ADIPOQ/CDKN1A/PARP1/SRC	5
BP	GO:0030071	regulation of mitotic metaphase/anaphase transition	4/132	90/18870	0.00367487	0.01053463	0.00481567	CCNB1/RB1/BIRC5/PLK1	4
BP	GO:0071867	response to monoamine	4/132	90/18870	0.00367487	0.01053463	0.00481567	ADIPOQ/DRD2/GSK3B/ALK	4
BP	GO:0071869	response to catecholamine	4/132	90/18870	0.00367487	0.01053463	0.00481567	ADIPOQ/DRD2/GSK3B/ALK	4
BP	GO:0032006	regulation of TOR signaling	5/132	147/18870	0.0037555	0.01075823	0.00491789	SYK/PIM1/SRC/AKT1/NUAK1	5
BP	GO:0045619	regulation of lymphocyte differentiation	6/132	212/18870	0.00379831	0.01087323	0.00497046	BAD/IL2RA/IL4R/SYK/AXL/IL2	6
BP	GO:0032273	positive regulation of protein polymerization	4/132	91/18870	0.00382332	0.01091397	0.00498908	HSP90AA1/ALOX15/MET/MAPT	4
BP	GO:0106027	neuron projection organization	4/132	91/18870	0.00382332	0.01091397	0.00498908	APP/GSK3B/IGF1R/INSR	4
BP	GO:0010907	positive regulation of glucose metabolic process	3/132	45/18870	0.00383124	0.01091397	0.00498908	SRC/AKT1/INSR	3
BP	GO:0014014	negative regulation of gliogenesis	3/132	45/18870	0.00383124	0.01091397	0.00498908	TP53/RB1/F2	3
BP	GO:0030890	positive regulation of B cell proliferation	3/132	45/18870	0.00383124	0.01091397	0.00498908	BCL2/CDKN1A/IL2	3
BP	GO:0090278	negative regulation of peptide hormone secretion	3/132	45/18870	0.00383124	0.01091397	0.00498908	KLF7/DRD2/PTPN11	3
BP	GO:0140894	endolysosomal toll-like receptor signaling pathway	3/132	45/18870	0.00383124	0.01091397	0.00498908	TNF/CAV1/SRC	3
BP	GO:0032946	positive regulation of mononuclear cell proliferation	5/132	148/18870	0.00386542	0.01100365	0.00503008	BCL2/CDKN1A/IL2RA/SYK/IL2	5
BP	GO:1901873	regulation of post-translational protein modification	7/132	284/18870	0.0038743	0.01101649	0.00503595	HSP90AA1/RELA/XIAP/CAV1/GSK3B/PLK1/AKT1	7
BP	GO:0000280	nuclear division	9/132	441/18870	0.00387532	0.01101649	0.00503595	CDK1/CCNB1/TNF/RB1/BIRC5/EDN1/PLK1/TOP2A/INS	9
BP	GO:1901888	regulation of cell junction assembly	6/132	213/18870	0.00388645	0.01104043	0.00504689	TNF/APP/CAV1/SRC/PTK2/KDR	6
BP	GO:0050768	negative regulation of neurogenesis	5/132	149/18870	0.00397762	0.01129158	0.0051617	TP53/TNF/RB1/SPP1/F2	5
BP	GO:0002792	negative regulation of peptide secretion	3/132	46/18870	0.0040778	0.01147231	0.00524432	KLF7/DRD2/PTPN11	3
BP	GO:0005978	glycogen biosynthetic process	3/132	46/18870	0.0040778	0.01147231	0.00524432	GSK3B/AKT1/INSR	3
BP	GO:0007094	mitotic spindle assembly checkpoint signaling	3/132	46/18870	0.0040778	0.01147231	0.00524432	CCNB1/BIRC5/PLK1	3
BP	GO:0009250	glucan biosynthetic process	3/132	46/18870	0.0040778	0.01147231	0.00524432	GSK3B/AKT1/INSR	3
BP	GO:0032881	regulation of polysaccharide metabolic process	3/132	46/18870	0.0040778	0.01147231	0.00524432	GSK3B/AKT1/INSR	3
BP	GO:0035272	exocrine system development	3/132	46/18870	0.0040778	0.01147231	0.00524432	TNF/EGFR/INSR	3
BP	GO:0042789	mRNA transcription by RNA polymerase II	3/132	46/18870	0.0040778	0.01147231	0.00524432	RXRA/STAT3/NR1H3	3
BP	GO:0045646	regulation of erythrocyte differentiation	3/132	46/18870	0.0040778	0.01147231	0.00524432	STAT3/STAT1/CDK6	3
BP	GO:0055078	sodium ion homeostasis	3/132	46/18870	0.0040778	0.01147231	0.00524432	DRD2/EDN1/EDNRA	3
BP	GO:0071173	spindle assembly checkpoint signaling	3/132	46/18870	0.0040778	0.01147231	0.00524432	CCNB1/BIRC5/PLK1	3
BP	GO:0071174	mitotic spindle checkpoint signaling	3/132	46/18870	0.0040778	0.01147231	0.00524432	CCNB1/BIRC5/PLK1	3
BP	GO:1902175	regulation of oxidative stress-induced intrinsic apoptotic signaling pathwa	3/132	46/18870	0.0040778	0.01147231	0.00524432	MCL1/PARP1/AKT1	3
BP	GO:1990090	cellular response to nerve growth factor stimulus	3/132	46/18870	0.0040778	0.01147231	0.00524432	PARP1/AKT1/MAPT	3
BP	GO:0045667	regulation of osteoblast differentiation	5/132	150/18870	0.00409213	0.01149681	0.00525552	TNF/IL6R/RUNX2/PTK2/CDK6	5
BP	GO:0072073	kidney epithelium development	5/132	150/18870	0.00409213	0.01149681	0.00525552	BCL2/ADIPOQ/MYC/EDNRA/STAT1	5
BP	GO:0001942	hair follicle development	4/132	93/18870	0.00413231	0.01154617	0.00527808	RELA/BCL2/TNF/EGFR	4
BP	GO:0032436	positive regulation of proteasomal ubiquitin-dependent protein catabolic	4/132	93/18870	0.00413231	0.01154617	0.00527808	CAV1/GSK3B/PLK1/AKT1	4
BP	GO:0032465	regulation of cytokinesis	4/132	93/18870	0.00413231	0.01154617	0.00527808	DRD2/BIRC5/IGF1R/PLK1	4
BP	GO:0042475	odontogenesis of dentin-containing tooth	4/132	93/18870	0.00413231	0.01154617	0.00527808	SCN5A/BAX/RUNX2/CFTR	4
BP	GO:0043279	response to alkaloid	4/132	93/18870	0.00413231	0.01154617	0.00527808	HSP90AA1/DRD2/IGF1R/ADORA2A	4
BP	GO:0043367	CD4-positive, alpha-beta T cell differentiation	4/132	93/18870	0.00413231	0.01154617	0.00527808	IL6R/IL4R/STAT3/IL2	4
BP	GO:1902099	regulation of metaphase/anaphase transition of cell cycle	4/132	93/18870	0.00413231	0.01154617	0.00527808	CCNB1/RB1/BIRC5/PLK1	4
BP	GO:1903432	regulation of TORC1 signaling	4/132	93/18870	0.00413231	0.01154617	0.00527808	SYK/PIM1/SRC/AKT1	4
BP	GO:0030238	male sex determination	2/132	14/18870	0.00418258	0.01159147	0.00529879	AR/INSR	2
BP	GO:0030252	growth hormone secretion	2/132	14/18870	0.00418258	0.01159147	0.00529879	DRD2/PTPN11	2
BP	GO:0051412	response to corticosterone	2/132	14/18870	0.00418258	0.01159147	0.00529879	FOS/CDKN1A	2

BP	GO:0060330	regulation of response to type II interferon	2/132	14/18870	0.00418258	0.01159147	0.00529879	ARG1/NR1H3	2
BP	GO:0060334	regulation of type II interferon-mediated signaling pathway	2/132	14/18870	0.00418258	0.01159147	0.00529879	ARG1/NR1H3	2
BP	GO:0061043	regulation of vascular wound healing	2/132	14/18870	0.00418258	0.01159147	0.00529879	TNF/ALOX5	2
BP	GO:0070942	neutrophil mediated cytotoxicity	2/132	14/18870	0.00418258	0.01159147	0.00529879	ARG1/F2	2
BP	GO:0072075	metanephric mesenchyme development	2/132	14/18870	0.00418258	0.01159147	0.00529879	MYC/STAT1	2
BP	GO:1900452	regulation of long-term synaptic depression	2/132	14/18870	0.00418258	0.01159147	0.00529879	ADORA1/MAPT	2
BP	GO:1902287	semaphorin-plexin signaling pathway involved in axon guidance	2/132	14/18870	0.00418258	0.01159147	0.00529879	EDN1/EDNRA	2
BP	GO:1904321	response to forskolin	2/132	14/18870	0.00418258	0.01159147	0.00529879	AHR/CFTR	2
BP	GO:1904322	cellular response to forskolin	2/132	14/18870	0.00418258	0.01159147	0.00529879	AHR/CFTR	2
BP	GO:0071322	cellular response to carbohydrate stimulus	5/132	151/18870	0.00420899	0.01165676	0.00532863	BAD/KLF7/NOX4/IGF1R/CFTR	5
BP	GO:0007091	metaphase/anaphase transition of mitotic cell cycle	4/132	94/18870	0.00429296	0.01187321	0.00542758	CCNB1/RB1/BIRC5/PLK1	4
BP	GO:0021766	hippocampus development	4/132	94/18870	0.00429296	0.01187321	0.00542758	GSK3B/IGF1R/ALK/CDK6	4
BP	GO:0002701	negative regulation of production of molecular mediator of immune respc	3/132	47/18870	0.00433379	0.01188156	0.0054314	TNF/ARG1/AXL	3
BP	GO:0030261	chromosome condensation	3/132	47/18870	0.00433379	0.01188156	0.0054314	CDK1/PLK1/TOP2A	3
BP	GO:0031577	spindle checkpoint signaling	3/132	47/18870	0.00433379	0.01188156	0.0054314	CCNB1/BIRC5/PLK1	3
BP	GO:0032467	positive regulation of cytokinesis	3/132	47/18870	0.00433379	0.01188156	0.0054314	DRD2/BIRC5/IGF1R	3
BP	GO:0035794	positive regulation of mitochondrial membrane permeability	3/132	47/18870	0.00433379	0.01188156	0.0054314	BAX/TP53/GSK3B	3
BP	GO:0042311	vasodilation	3/132	47/18870	0.00433379	0.01188156	0.0054314	TNF/ADORA1/ADORA2A	3
BP	GO:0043666	regulation of phosphoprotein phosphatase activity	3/132	47/18870	0.00433379	0.01188156	0.0054314	TNF/GSK3B/NUAK1	3
BP	GO:0045933	positive regulation of muscle contraction	3/132	47/18870	0.00433379	0.01188156	0.0054314	HSP90AA1/EDN1/PTGS2	3
BP	GO:0046633	alpha-beta T cell proliferation	3/132	47/18870	0.00433379	0.01188156	0.0054314	IL2RA/SYK/IL2	3
BP	GO:0046636	negative regulation of alpha-beta T cell activation	3/132	47/18870	0.00433379	0.01188156	0.0054314	IL4R/ADORA2A/IL2	3
BP	GO:0050798	activated T cell proliferation	3/132	47/18870	0.00433379	0.01188156	0.0054314	IL2RA/ARG1/IL2	3
BP	GO:0072538	T-helper 17 type immune response	3/132	47/18870	0.00433379	0.01188156	0.0054314	IL6R/STAT3/IL2	3
BP	GO:0120163	negative regulation of cold-induced thermogenesis	3/132	47/18870	0.00433379	0.01188156	0.0054314	ADIPOQ/RB1/NR1H3	3
BP	GO:0002861	regulation of inflammatory response to antigenic stimulus	3/132	48/18870	0.00459933	0.0125255	0.00572576	TNF/SYK/SRC	3
BP	GO:0008542	visual learning	3/132	48/18870	0.00459933	0.0125255	0.00572576	DRD2/APP/HMGCR	3
BP	GO:0030195	negative regulation of blood coagulation	3/132	48/18870	0.00459933	0.0125255	0.00572576	EDN1/F2/ALOX12	3
BP	GO:0031670	cellular response to nutrient	3/132	48/18870	0.00459933	0.0125255	0.00572576	RXRA/COL1A1/PIM1	3
BP	GO:0033046	negative regulation of sister chromatid segregation	3/132	48/18870	0.00459933	0.0125255	0.00572576	CCNB1/BIRC5/PLK1	3
BP	GO:0033048	negative regulation of mitotic sister chromatid segregation	3/132	48/18870	0.00459933	0.0125255	0.00572576	CCNB1/BIRC5/PLK1	3
BP	GO:0045841	negative regulation of mitotic metaphase/anaphase transition	3/132	48/18870	0.00459933	0.0125255	0.00572576	CCNB1/BIRC5/PLK1	3
BP	GO:1990089	response to nerve growth factor	3/132	48/18870	0.00459933	0.0125255	0.00572576	PARP1/AKT1/MAPT	3
BP	GO:2000816	negative regulation of mitotic sister chromatid separation	3/132	48/18870	0.00459933	0.0125255	0.00572576	CCNB1/BIRC5/PLK1	3
BP	GO:2001222	regulation of neuron migration	3/132	48/18870	0.00459933	0.0125255	0.00572576	DRD2/STAT3/COL3A1	3
BP	GO:0002690	positive regulation of leukocyte chemotaxis	4/132	96/18870	0.00462679	0.01256677	0.00574463	IL6R/EDN1/CXCL10/PTK2	4
BP	GO:0022404	molting cycle process	4/132	96/18870	0.00462679	0.01256677	0.00574463	RELA/BCL2/TNF/EGFR	4
BP	GO:0022405	hair cycle process	4/132	96/18870	0.00462679	0.01256677	0.00574463	RELA/BCL2/TNF/EGFR	4
BP	GO:0034109	homotypic cell-cell adhesion	4/132	96/18870	0.00462679	0.01256677	0.00574463	ITGB3/SYK/ALOX12/PIK3CG	4
BP	GO:0051961	negative regulation of nervous system development	5/132	155/18870	0.00470032	0.012758	0.00583204	TP53/TNF/RB1/SPP1/F2	5
BP	GO:0044784	metaphase/anaphase transition of cell cycle	4/132	97/18870	0.00480007	0.01280133	0.00585185	CCNB1/RB1/BIRC5/PLK1	4
BP	GO:0051952	regulation of amine transport	4/132	97/18870	0.00480007	0.01280133	0.00585185	TNF/DRD2/ADORA1/ADORA2A	4
BP	GO:0061337	cardiac conduction	4/132	97/18870	0.00480007	0.01280133	0.00585185	KCNH2/SCN5A/CAV1/GJA1	4
BP	GO:0003376	sphingosine-1-phosphate receptor signaling pathway	2/132	15/18870	0.00480403	0.01280133	0.00585185	AKT1/PIK3CG	2
BP	GO:0007567	parturition	2/132	15/18870	0.00480403	0.01280133	0.00585185	EDN1/MMP2	2
BP	GO:0014029	neural crest formation	2/132	15/18870	0.00480403	0.01280133	0.00585185	EDN1/EDNRA	2
BP	GO:0016322	neuron remodeling	2/132	15/18870	0.00480403	0.01280133	0.00585185	APP/EDNRA	2
BP	GO:0032230	positive regulation of synaptic transmission, GABAergic	2/132	15/18870	0.00480403	0.01280133	0.00585185	CA2/ADORA2A	2
BP	GO:0033008	positive regulation of mast cell activation involved in immune response	2/132	15/18870	0.00480403	0.01280133	0.00585185	IL4R/SYK	2

BP	GO:0033599	regulation of mammary gland epithelial cell proliferation	2/132	15/18870	0.00480403	0.01280133	0.00585185	BAX/CCND1	2
BP	GO:0036446	myofibroblast differentiation	2/132	15/18870	0.00480403	0.01280133	0.00585185	RB1/PARP1	2
BP	GO:0038065	collagen-activated signaling pathway	2/132	15/18870	0.00480403	0.01280133	0.00585185	COL1A1/SYK	2
BP	GO:0043306	positive regulation of mast cell degranulation	2/132	15/18870	0.00480403	0.01280133	0.00585185	IL4R/SYK	2
BP	GO:0045725	positive regulation of glycogen biosynthetic process	2/132	15/18870	0.00480403	0.01280133	0.00585185	AKT1/INSR	2
BP	GO:0045838	positive regulation of membrane potential	2/132	15/18870	0.00480403	0.01280133	0.00585185	BAD/AKT1	2
BP	GO:0048070	regulation of developmental pigmentation	2/132	15/18870	0.00480403	0.01280133	0.00585185	BCL2/BAX	2
BP	GO:0048308	organelle inheritance	2/132	15/18870	0.00480403	0.01280133	0.00585185	CDK1/PLK1	2
BP	GO:0048313	Golgi inheritance	2/132	15/18870	0.00480403	0.01280133	0.00585185	CDK1/PLK1	2
BP	GO:0061307	cardiac neural crest cell differentiation involved in heart development	2/132	15/18870	0.00480403	0.01280133	0.00585185	EDN1/EDNRA	2
BP	GO:0061308	cardiac neural crest cell development involved in heart development	2/132	15/18870	0.00480403	0.01280133	0.00585185	EDN1/EDNRA	2
BP	GO:0071639	positive regulation of monocyte chemotactic protein-1 production	2/132	15/18870	0.00480403	0.01280133	0.00585185	ADIPOQ/SYK	2
BP	GO:0071732	cellular response to nitric oxide	2/132	15/18870	0.00480403	0.01280133	0.00585185	CDK2/MMP3	2
BP	GO:1901386	negative regulation of voltage-gated calcium channel activity	2/132	15/18870	0.00480403	0.01280133	0.00585185	DRD2/GPR35	2
BP	GO:1902065	response to L-glutamate	2/132	15/18870	0.00480403	0.01280133	0.00585185	TNF/IGF1R	2
BP	GO:1902563	regulation of neutrophil activation	2/132	15/18870	0.00480403	0.01280133	0.00585185	TNF/SYK	2
BP	GO:2000402	negative regulation of lymphocyte migration	2/132	15/18870	0.00480403	0.01280133	0.00585185	CCL2/AKT1	2
BP	GO:2000671	regulation of motor neuron apoptotic process	2/132	15/18870	0.00480403	0.01280133	0.00585185	BCL2/BAX	2
BP	GO:2001028	positive regulation of endothelial cell chemotaxis	2/132	15/18870	0.00480403	0.01280133	0.00585185	KDR/MET	2
BP	GO:0044242	cellular lipid catabolic process	6/132	223/18870	0.0048534	0.01290498	0.00589923	ADIPOQ/CYP1B1/AKR1B10/AKT1/AKR1C3/PIK3CG	6
BP	GO:0001774	microglial cell activation	3/132	49/18870	0.00487451	0.01290498	0.00589923	TNF/APP/MAPT	3
BP	GO:0008608	attachment of spindle microtubules to kinetochore	3/132	49/18870	0.00487451	0.01290498	0.00589923	CCNB1/RB1/BIRC5	3
BP	GO:0010171	body morphogenesis	3/132	49/18870	0.00487451	0.01290498	0.00589923	COL1A1/MMP2/PTPN11	3
BP	GO:0010543	regulation of platelet activation	3/132	49/18870	0.00487451	0.01290498	0.00589923	SYK/F2/ALOX12	3
BP	GO:1900047	negative regulation of hemostasis	3/132	49/18870	0.00487451	0.01290498	0.00589923	EDN1/F2/ALOX12	3
BP	GO:1902108	regulation of mitochondrial membrane permeability involved in apoptotic	3/132	49/18870	0.00487451	0.01290498	0.00589923	BAX/TP53/GSK3B	3
BP	GO:1903170	negative regulation of calcium ion transmembrane transport	3/132	49/18870	0.00487451	0.01290498	0.00589923	BCL2/DRD2/GPR35	3
BP	GO:2000378	negative regulation of reactive oxygen species metabolic process	3/132	49/18870	0.00487451	0.01290498	0.00589923	BCL2/TP53/MMP3	3
BP	GO:2000404	regulation of T cell migration	3/132	49/18870	0.00487451	0.01290498	0.00589923	APP/ITGB3/CXCL10	3
BP	GO:0030902	hindbrain development	5/132	157/18870	0.00496069	0.01312463	0.00599964	SCN5A/BCL2/TP53/IGF1R/PTPN11	5
BP	GO:0002275	myeloid cell activation involved in immune response	4/132	98/18870	0.00497767	0.01312704	0.00600074	TNF/IL4R/SYK/PIK3CG	4
BP	GO:0006721	terpenoid metabolic process	4/132	98/18870	0.00497767	0.01312704	0.00600074	AKR1B1/CYP1B1/AKR1B10/AKR1C3	4
BP	GO:0006835	dicarboxylic acid transport	4/132	98/18870	0.00497767	0.01312704	0.00600074	TNF/GJA1/ADORA1/ADORA2A	4
BP	GO:0008593	regulation of Notch signaling pathway	4/132	98/18870	0.00497767	0.01312704	0.00600074	NFKBIA/STAT3/SRC/AKT1	4
BP	GO:0032410	negative regulation of transporter activity	4/132	98/18870	0.00497767	0.01312704	0.00600074	DRD2/CAV1/MMP9/GPR35	4
BP	GO:0000819	sister chromatid segregation	6/132	225/18870	0.00506628	0.01335209	0.00610362	CDK1/CCNB1/RB1/BIRC5/PLK1/TOP2A	6
BP	GO:0090316	positive regulation of intracellular protein transport	5/132	158/18870	0.00509465	0.01341821	0.00613384	HSP90AA1/CDK1/GSK3B/PIK3R1/PTGS2	5
BP	GO:0010656	negative regulation of muscle cell apoptotic process	3/132	50/18870	0.00515944	0.01350229	0.00617227	EDN1/IGF1R/ALOX12	3
BP	GO:0022602	ovulation cycle process	3/132	50/18870	0.00515944	0.01350229	0.00617227	MMP2/SRC/ESR1	3
BP	GO:0042149	cellular response to glucose starvation	3/132	50/18870	0.00515944	0.01350229	0.00617227	BCL2/TP53/NUAK1	3
BP	GO:0051985	negative regulation of chromosome segregation	3/132	50/18870	0.00515944	0.01350229	0.00617227	CCNB1/BIRC5/PLK1	3
BP	GO:1902100	negative regulation of metaphase/anaphase transition of cell cycle	3/132	50/18870	0.00515944	0.01350229	0.00617227	CCNB1/BIRC5/PLK1	3
BP	GO:1905819	negative regulation of chromosome separation	3/132	50/18870	0.00515944	0.01350229	0.00617227	CCNB1/BIRC5/PLK1	3
BP	GO:0038202	TORC1 signaling	4/132	99/18870	0.00515963	0.01350229	0.00617227	SYK/PIM1/SRC/AKT1	4
BP	GO:0046620	regulation of organ growth	4/132	99/18870	0.00515963	0.01350229	0.00617227	CDK1/EDN1/PIM1/AKT1	4
BP	GO:0098869	cellular oxidant detoxification	4/132	99/18870	0.00515963	0.01350229	0.00617227	MPO/PTGS1/PTGS2/DHFR	4
BP	GO:0141060	disruption of anatomical structure in another organism	4/132	99/18870	0.00515963	0.01350229	0.00617227	SYK/ARG1/F2/CSNK2A1	4
BP	GO:0032970	regulation of actin filament-based process	8/132	379/18870	0.00523214	0.01368328	0.00625501	SCN5A/EDN1/ITGB3/CAV1/ADORA1/PIK3R1/ALOX15/	8
BP	GO:0060349	bone morphogenesis	4/132	100/18870	0.00534599	0.01397208	0.00638703	COL1A1/COL3A1/RUNX2/MMP13	4

BP	GO:0007163	establishment or maintenance of cell polarity	6/132	228/18870	0.00539835	0.01408558	0.00643891	HSP90AA1/GJA1/GSK3B/IGF1R/PTK2/PLK1	6
BP	GO:0001974	blood vessel remodeling	3/132	51/18870	0.00545421	0.01408558	0.00643891	BAX/EDNRA/AXL	3
BP	GO:0038084	vascular endothelial growth factor signaling pathway	3/132	51/18870	0.00545421	0.01408558	0.00643891	XDH/FLT3/KDR	3
BP	GO:1905710	positive regulation of membrane permeability	3/132	51/18870	0.00545421	0.01408558	0.00643891	BAX/TP53/GSK3B	3
BP	GO:0002679	respiratory burst involved in defense response	2/132	16/18870	0.00546527	0.01408558	0.00643891	MPO/PIK3CG	2
BP	GO:0002903	negative regulation of B cell apoptotic process	2/132	16/18870	0.00546527	0.01408558	0.00643891	BCL2/IL2	2
BP	GO:0003096	renal sodium ion transport	2/132	16/18870	0.00546527	0.01408558	0.00643891	EDN1/EDNRA	2
BP	GO:0031645	negative regulation of nervous system process	2/132	16/18870	0.00546527	0.01408558	0.00643891	TNF/GPR35	2
BP	GO:0032930	positive regulation of superoxide anion generation	2/132	16/18870	0.00546527	0.01408558	0.00643891	SYK/MAPT	2
BP	GO:0034374	low-density lipoprotein particle remodeling	2/132	16/18870	0.00546527	0.01408558	0.00643891	MTTP/MPO	2
BP	GO:0035627	ceramide transport	2/132	16/18870	0.00546527	0.01408558	0.00643891	MTTP/ABCB1	2
BP	GO:0046653	tetrahydrofolate metabolic process	2/132	16/18870	0.00546527	0.01408558	0.00643891	TYMS/DHFR	2
BP	GO:0051956	negative regulation of amino acid transport	2/132	16/18870	0.00546527	0.01408558	0.00643891	TNF/ADORA1	2
BP	GO:0070230	positive regulation of lymphocyte apoptotic process	2/132	16/18870	0.00546527	0.01408558	0.00643891	BAX/TP53	2
BP	GO:0072540	T-helper 17 cell lineage commitment	2/132	16/18870	0.00546527	0.01408558	0.00643891	IL6R/STAT3	2
BP	GO:0090336	positive regulation of brown fat cell differentiation	2/132	16/18870	0.00546527	0.01408558	0.00643891	PIM1/PTGS2	2
BP	GO:1900034	regulation of cellular response to heat	2/132	16/18870	0.00546527	0.01408558	0.00643891	GSK3B/MAPT	2
BP	GO:1902285	semaphorin-plexin signaling pathway involved in neuron projection guida	2/132	16/18870	0.00546527	0.01408558	0.00643891	EDN1/EDNRA	2
BP	GO:1903798	regulation of miRNA processing	2/132	16/18870	0.00546527	0.01408558	0.00643891	TP53/STAT3	2
BP	GO:1904177	regulation of adipose tissue development	2/132	16/18870	0.00546527	0.01408558	0.00643891	KLF7/PARP1	2
BP	GO:2000696	regulation of epithelial cell differentiation involved in kidney development	2/132	16/18870	0.00546527	0.01408558	0.00643891	STAT1/MMP9	2
BP	GO:2001212	regulation of vasculogenesis	2/132	16/18870	0.00546527	0.01408558	0.00643891	XDH/KDR	2
BP	GO:0002443	leukocyte mediated immunity	9/132	466/18870	0.00552917	0.0142323	0.00650599	TNF/IL4R/SYK/ARG1/F2/PLA2G1B/IL2/PIK3CG/AHR	9
BP	GO:0051960	regulation of nervous system development	9/132	466/18870	0.00552917	0.0142323	0.00650599	RELA/TP53/TNF/DRD2/RB1/SPP1/F2/AKT1/MAPT	9
BP	GO:0043266	regulation of potassium ion transport	4/132	102/18870	0.00573212	0.01474005	0.00673809	KCNH2/DRD2/CAV1/ADORA1	4
BP	GO:0002269	leukocyte activation involved in inflammatory response	3/132	52/18870	0.0057589	0.01474005	0.00673809	TNF/APP/MAPT	3
BP	GO:0006110	regulation of glycolytic process	3/132	52/18870	0.0057589	0.01474005	0.00673809	APP/STAT3/INSR	3
BP	GO:0010823	negative regulation of mitochondrion organization	3/132	52/18870	0.0057589	0.01474005	0.00673809	TP53/AKT1/MAPT	3
BP	GO:0050819	negative regulation of coagulation	3/132	52/18870	0.0057589	0.01474005	0.00673809	EDN1/F2/ALOX12	3
BP	GO:0051180	vitamin transport	3/132	52/18870	0.0057589	0.01474005	0.00673809	SLC2A4/ABCG2/ABCC1	3
BP	GO:0060688	regulation of morphogenesis of a branching structure	3/132	52/18870	0.0057589	0.01474005	0.00673809	AR/TNF/ESR1	3
BP	GO:1900087	positive regulation of G1/S transition of mitotic cell cycle	3/132	52/18870	0.0057589	0.01474005	0.00673809	EGFR/AKT1/TERT	3
BP	GO:1901570	fatty acid derivative biosynthetic process	3/132	52/18870	0.0057589	0.01474005	0.00673809	ALOX5/ALOX15/ALOX12	3
BP	GO:0007254	JNK cascade	5/132	163/18870	0.00580314	0.01482541	0.00677711	TNF/APP/XIAP/IGF1R/EGFR	5
BP	GO:0016052	carbohydrate catabolic process	5/132	163/18870	0.00580314	0.01482541	0.00677711	TP53/BAD/APP/STAT3/INSR	5
BP	GO:1902850	microtubule cytoskeleton organization involved in mitosis	5/132	163/18870	0.00580314	0.01482541	0.00677711	CDK1/CCNB1/BIRC5/GJA1/PLK1	5
BP	GO:0022412	cellular process involved in reproduction in multicellular organism	9/132	471/18870	0.00591732	0.01510766	0.00690613	BCL2/BAX/EDN1/EDNRA/SRC/PLK1/AKT1/TOP2A/CFT	9
BP	GO:0006304	DNA modification	4/132	103/18870	0.00593198	0.01512618	0.0069146	FOS/PARP1/CYP1B1/APEX1	4
BP	GO:0006885	regulation of pH	4/132	103/18870	0.00593198	0.01512618	0.0069146	BCL2/EDN1/CA2/CFTR	4
BP	GO:0043124	negative regulation of canonical NF-kappaB signal transduction	3/132	53/18870	0.00607361	0.01544876	0.00706206	ADIPOQ/STAT1/ESR1	3
BP	GO:0044818	mitotic G2/M transition checkpoint	3/132	53/18870	0.00607361	0.01544876	0.00706206	CDK1/CDKN1A/PLK1	3
BP	GO:0045778	positive regulation of ossification	3/132	53/18870	0.00607361	0.01544876	0.00706206	RXRA/ALOX5/PTPN11	3
BP	GO:1902743	regulation of lamellipodium organization	3/132	53/18870	0.00607361	0.01544876	0.00706206	HSP90AA1/PIK3R1/SRC	3
BP	GO:0035051	cardiocyte differentiation	5/132	165/18870	0.00610508	0.01548067	0.00707665	CDK1/EDN1/EDNRA/NOX4/EGFR	5
BP	GO:0021549	cerebellum development	4/132	104/18870	0.00613644	0.01548067	0.00707665	SCN5A/TP53/IGF1R/PTPN11	4
BP	GO:1905477	positive regulation of protein localization to membrane	4/132	104/18870	0.00613644	0.01548067	0.00707665	TNF/EGFR/PIK3R1/AKT1	4
BP	GO:0002076	osteoblast development	2/132	17/18870	0.00616574	0.01548067	0.00707665	RUNX2/ACHE	2
BP	GO:0007252	I-kappaB phosphorylation	2/132	17/18870	0.00616574	0.01548067	0.00707665	TNF/AKT1	2
BP	GO:0009886	post-embryonic animal morphogenesis	2/132	17/18870	0.00616574	0.01548067	0.00707665	BAX/KDR	2

BP	GO:0010544	negative regulation of platelet activation	2/132	17/18870	0.00616574	0.01548067	0.00707665	F2/ALOX12	2
BP	GO:0031649	heat generation	2/132	17/18870	0.00616574	0.01548067	0.00707665	TNF/PTGS2	2
BP	GO:0032310	prostaglandin secretion	2/132	17/18870	0.00616574	0.01548067	0.00707665	EDN1/PTGS2	2
BP	GO:0036005	response to macrophage colony-stimulating factor	2/132	17/18870	0.00616574	0.01548067	0.00707665	TNF/SPP1	2
BP	GO:0060253	negative regulation of glial cell proliferation	2/132	17/18870	0.00616574	0.01548067	0.00707665	TP53/RB1	2
BP	GO:0070875	positive regulation of glycogen metabolic process	2/132	17/18870	0.00616574	0.01548067	0.00707665	AKT1/INSR	2
BP	GO:0070920	regulation of regulatory ncRNA processing	2/132	17/18870	0.00616574	0.01548067	0.00707665	TP53/STAT3	2
BP	GO:0072074	kidney mesenchyme development	2/132	17/18870	0.00616574	0.01548067	0.00707665	MYC/STAT1	2
BP	GO:0072109	glomerular mesangium development	2/132	17/18870	0.00616574	0.01548067	0.00707665	IL6R/ITGB3	2
BP	GO:0097396	response to interleukin-17	2/132	17/18870	0.00616574	0.01548067	0.00707665	STAT3/CXCL10	2
BP	GO:0097398	cellular response to interleukin-17	2/132	17/18870	0.00616574	0.01548067	0.00707665	STAT3/CXCL10	2
BP	GO:0120305	regulation of pigmentation	2/132	17/18870	0.00616574	0.01548067	0.00707665	BCL2/BAX	2
BP	GO:1900120	regulation of receptor binding	2/132	17/18870	0.00616574	0.01548067	0.00707665	ADIPOQ/MMP9	2
BP	GO:1902170	cellular response to reactive nitrogen species	2/132	17/18870	0.00616574	0.01548067	0.00707665	CDK2/MMP3	2
BP	GO:2000310	regulation of NMDA receptor activity	2/132	17/18870	0.00616574	0.01548067	0.00707665	APP/CCL2	2
BP	GO:0046486	glycerolipid metabolic process	8/132	390/18870	0.00618683	0.01551454	0.00709213	MTTP/CAV1/PIK3R1/ALOX15/PLA2G1B/PTPN11/NR1H	8
BP	GO:0050767	regulation of neurogenesis	8/132	390/18870	0.00618683	0.01551454	0.00709213	RELA/TP53/TNF/DRD2/RB1/SPP1/F2/MAPT	8
BP	GO:0001654	eye development	8/132	391/18870	0.00627978	0.01573796	0.00719427	BCL2/BAX/DRD2/STAT3/CYP1B1/EGFR/KDR/ACHE	8
BP	GO:0043255	regulation of carbohydrate biosynthetic process	4/132	105/18870	0.00634553	0.01589299	0.00726514	ADIPOQ/GSK3B/AKT1/INSR	4
BP	GO:0001701	in utero embryonic development	8/132	392/18870	0.00637378	0.01595398	0.00729302	AR/TP53/CCNB1/EDN1/EDNRA/COL3A1/EGFR/AKT1	8
BP	GO:0010761	fibroblast migration	3/132	54/18870	0.00639842	0.01596674	0.00729884	ITGB3/PTK2/AKT1	3
BP	GO:0043303	mast cell degranulation	3/132	54/18870	0.00639842	0.01596674	0.00729884	IL4R/SYK/PIK3CG	3
BP	GO:0061178	regulation of insulin secretion involved in cellular response to glucose stim	3/132	54/18870	0.00639842	0.01596674	0.00729884	BAD/KLF7/CFTR	3
BP	GO:0070169	positive regulation of biomineral tissue development	3/132	54/18870	0.00639842	0.01596674	0.00729884	RXRA/ALOX5/CFTR	3
BP	GO:0086009	membrane repolarization	3/132	54/18870	0.00639842	0.01596674	0.00729884	KCNH2/SCN5A/CAV1	3
BP	GO:0031929	TOR signaling	5/132	167/18870	0.00641791	0.01599583	0.00731215	SYK/PIM1/SRC/AKT1/NUAK1	5
BP	GO:0043433	negative regulation of DNA-binding transcription factor activity	5/132	167/18870	0.00641791	0.01599583	0.00731215	RB1/NFKBIA/CYP1B1/PIM1/ESR1	5
BP	GO:0050792	regulation of viral process	5/132	168/18870	0.00657848	0.01638603	0.00749051	BCL2/TNF/STAT1/AXL/TOP2A	5
BP	GO:0150063	visual system development	8/132	395/18870	0.00666224	0.01658455	0.00758126	BCL2/BAX/DRD2/STAT3/CYP1B1/EGFR/KDR/ACHE	8
BP	GO:0019395	fatty acid oxidation	4/132	107/18870	0.0067778	0.01686194	0.00770807	ADIPOQ/ALOX15/ALOX12/AKT1	4
BP	GO:0006977	DNA damage response, signal transduction by p53 class mediator resultin	2/132	18/18870	0.00690486	0.01708439	0.00780976	TP53/CDKN1A	2
BP	GO:0006978	DNA damage response, signal transduction by p53 class mediator resultin	2/132	18/18870	0.00690486	0.01708439	0.00780976	TP53/CDKN1A	2
BP	GO:0016264	gap junction assembly	2/132	18/18870	0.00690486	0.01708439	0.00780976	CAV1/GJA1	2
BP	GO:0038128	ERBB2 signaling pathway	2/132	18/18870	0.00690486	0.01708439	0.00780976	EGFR/SRC	2
BP	GO:0042481	regulation of odontogenesis	2/132	18/18870	0.00690486	0.01708439	0.00780976	EDN1/RUNX2	2
BP	GO:0042574	retinal metabolic process	2/132	18/18870	0.00690486	0.01708439	0.00780976	CYP1B1/AKR1C3	2
BP	GO:0048569	post-embryonic animal organ development	2/132	18/18870	0.00690486	0.01708439	0.00780976	BAX/KDR	2
BP	GO:0090520	sphingolipid mediated signaling pathway	2/132	18/18870	0.00690486	0.01708439	0.00780976	AKT1/PIK3CG	2
BP	GO:1901970	positive regulation of mitotic sister chromatid separation	2/132	18/18870	0.00690486	0.01708439	0.00780976	RB1/BIRC5	2
BP	GO:0050808	synapse organization	9/132	483/18870	0.00693496	0.0171485	0.00783906	TNF/DRD2/APP/ITGB3/IGF1R/AKT1/ACHE/MAPT/INSR	9
BP	GO:0015837	amine transport	4/132	108/18870	0.00700106	0.01730148	0.00790899	TNF/DRD2/ADORA1/ADORA2A	4
BP	GO:0002279	mast cell activation involved in immune response	3/132	56/18870	0.00707866	0.017451	0.00797734	IL4R/SYK/PIK3CG	3
BP	GO:0007632	visual behavior	3/132	56/18870	0.00707866	0.017451	0.00797734	DRD2/APP/HMGCR	3
BP	GO:0038093	Fc receptor signaling pathway	3/132	56/18870	0.00707866	0.017451	0.00797734	SYK/SRC/PTK2	3
BP	GO:0045839	negative regulation of mitotic nuclear division	3/132	56/18870	0.00707866	0.017451	0.00797734	CCNB1/BIRC5/PLK1	3
BP	GO:0090277	positive regulation of peptide hormone secretion	4/132	109/18870	0.00722914	0.01781125	0.00814202	BAD/DRD2/F2/CFTR	4
BP	GO:0006820	monoatomic anion transport	5/132	172/18870	0.00724899	0.01784937	0.00815945	CLDN4/CA2/ABCC1/ABCB1/CFTR	5
BP	GO:0048880	sensory system development	8/132	401/18870	0.00726878	0.01788733	0.0081768	BCL2/BAX/DRD2/STAT3/CYP1B1/EGFR/KDR/ACHE	8
BP	GO:0002448	mast cell mediated immunity	3/132	57/18870	0.00743424	0.01827248	0.00835286	IL4R/SYK/PIK3CG	3

BP	GO:1901616	organic hydroxy compound catabolic process	3/132	57/18870	0.00743424	0.01827248	0.00835286	SULT1E1/AKR1B10/AKR1C3	3
BP	GO:0010469	regulation of signaling receptor activity	4/132	110/18870	0.00746208	0.01831886	0.00837407	TNF/APP/EDN1/CCL2	4
BP	GO:0031398	positive regulation of protein ubiquitination	4/132	110/18870	0.00746208	0.01831886	0.00837407	XIAP/CAV1/GSK3B/PLK1	4
BP	GO:0002281	macrophage activation involved in immune response	2/132	19/18870	0.00768205	0.01872385	0.0085592	TNF/SYK	2
BP	GO:0002544	chronic inflammatory response	2/132	19/18870	0.00768205	0.01872385	0.0085592	CYP19A1/TNF	2
BP	GO:0032928	regulation of superoxide anion generation	2/132	19/18870	0.00768205	0.01872385	0.0085592	SYK/MAPT	2
BP	GO:0042772	DNA damage response, signal transduction resulting in transcription	2/132	19/18870	0.00768205	0.01872385	0.0085592	TP53/CDKN1A	2
BP	GO:0045623	negative regulation of T-helper cell differentiation	2/132	19/18870	0.00768205	0.01872385	0.0085592	IL4R/IL2	2
BP	GO:0051580	regulation of neurotransmitter uptake	2/132	19/18870	0.00768205	0.01872385	0.0085592	DRD2/ITGB3	2
BP	GO:0055070	copper ion homeostasis	2/132	19/18870	0.00768205	0.01872385	0.0085592	APP/XIAP	2
BP	GO:0071800	podosome assembly	2/132	19/18870	0.00768205	0.01872385	0.0085592	TNF/SRC	2
BP	GO:0086014	atrial cardiac muscle cell action potential	2/132	19/18870	0.00768205	0.01872385	0.0085592	SCN5A/GJA1	2
BP	GO:0086026	atrial cardiac muscle cell to AV node cell signaling	2/132	19/18870	0.00768205	0.01872385	0.0085592	SCN5A/GJA1	2
BP	GO:0086066	atrial cardiac muscle cell to AV node cell communication	2/132	19/18870	0.00768205	0.01872385	0.0085592	SCN5A/GJA1	2
BP	GO:1903376	regulation of oxidative stress-induced neuron intrinsic apoptotic signaling	2/132	19/18870	0.00768205	0.01872385	0.0085592	MCL1/PARP1	2
BP	GO:0032611	interleukin-1 beta production	4/132	111/18870	0.00769991	0.01874501	0.00856887	RELA/TNF/APP/STAT3	4
BP	GO:0032651	regulation of interleukin-1 beta production	4/132	111/18870	0.00769991	0.01874501	0.00856887	RELA/TNF/APP/STAT3	4
BP	GO:0086065	cell communication involved in cardiac conduction	3/132	58/18870	0.00780022	0.0189666	0.00867017	SCN5A/CAV1/GJA1	3
BP	GO:1903409	reactive oxygen species biosynthetic process	3/132	58/18870	0.00780022	0.0189666	0.00867017	NOX4/ALOX5/MPO	3
BP	GO:0022604	regulation of cell morphogenesis	6/132	247/18870	0.00788333	0.01915729	0.00875734	CCL2/CLDN4/F2/SRC/PTK2/KDR	6
BP	GO:0006367	transcription initiation at RNA polymerase II promoter	4/132	112/18870	0.00794268	0.01929004	0.00881802	TP53/CDK4/NFKBIA/ESR1	4
BP	GO:0002886	regulation of myeloid leukocyte mediated immunity	3/132	59/18870	0.00817667	0.01978774	0.00904553	IL4R/SYK/ARG1	3
BP	GO:0010574	regulation of vascular endothelial growth factor production	3/132	59/18870	0.00817667	0.01978774	0.00904553	RELA/CYP1B1/PTGS2	3
BP	GO:0014009	glial cell proliferation	3/132	59/18870	0.00817667	0.01978774	0.00904553	TP53/TNF/RB1	3
BP	GO:0051480	regulation of cytosolic calcium ion concentration	3/132	59/18870	0.00817667	0.01978774	0.00904553	CAV1/ADORA1/F2	3
BP	GO:0071168	protein localization to chromatin	3/132	59/18870	0.00817667	0.01978774	0.00904553	PARP1/PLK1/ESR1	3
BP	GO:2000107	negative regulation of leukocyte apoptotic process	3/132	59/18870	0.00817667	0.01978774	0.00904553	BCL2/AXL/IL2	3
BP	GO:0002703	regulation of leukocyte mediated immunity	6/132	249/18870	0.00818588	0.01979758	0.00905003	TNF/IL4R/SYK/ARG1/IL2/AHR	6
BP	GO:0007200	phospholipase C-activating G protein-coupled receptor signaling pathway	4/132	113/18870	0.00819043	0.01979758	0.00905003	DRD2/F2/GPR35/ESR1	4
BP	GO:0001837	epithelial to mesenchymal transition	5/132	178/18870	0.00834216	0.0201524	0.00921223	EDN1/EDNRA/COL1A1/GSK3B/PTK2	5
BP	GO:0018958	phenol-containing compound metabolic process	4/132	114/18870	8.44E-03	2.04E-02	9.31E-03	BCL2/DRD2/EDNRA/TYR	4
BP	GO:0002295	T-helper cell lineage commitment	2/132	20/18870	8.50E-03	2.04E-02	9.31E-03	IL6R/STAT3	2
BP	GO:0006144	purine nucleobase metabolic process	2/132	20/18870	8.50E-03	2.04E-02	9.31E-03	XDH/TTR	2
BP	GO:0014048	regulation of glutamate secretion	2/132	20/18870	8.50E-03	0.0203693	9.31E-03	ADORA1/ADORA2A	2
BP	GO:0032303	regulation of icosanoid secretion	2/132	20/18870	8.50E-03	0.0203693	0.00931138	EDN1/SYK	2
BP	GO:0032515	negative regulation of phosphoprotein phosphatase activity	2/132	20/18870	8.50E-03	0.0203693	0.00931138	TNF/GSK3B	2
BP	GO:0042474	middle ear morphogenesis	2/132	20/18870	8.50E-03	0.0203693	0.00931138	EDN1/EDNRA	2
BP	GO:0045056	transcytosis	2/132	20/18870	8.50E-03	0.0203693	0.00931138	IGF1R/SRC	2
BP	GO:0045655	regulation of monocyte differentiation	2/132	20/18870	8.50E-03	0.0203693	0.00931138	MYC/CDK6	2
BP	GO:0070242	thymocyte apoptotic process	2/132	20/18870	0.00849677	0.0203693	0.00931138	BAX/TP53	2
BP	GO:0071731	response to nitric oxide	2/132	20/18870	0.00849677	0.0203693	0.00931138	CDK2/MMP3	2
BP	GO:1902004	positive regulation of amyloid-beta formation	2/132	20/18870	0.00849677	0.0203693	0.00931138	RELA/TNF	2
BP	GO:2000269	regulation of fibroblast apoptotic process	2/132	20/18870	0.00849677	0.0203693	0.00931138	TP53/PIK3CG	2
BP	GO:1990138	neuron projection extension	5/132	179/18870	0.00853486	0.02044568	0.0093463	HSP90AA1/EDN1/EDNRA/GSK3B/MAPT	5
BP	GO:0001961	positive regulation of cytokine-mediated signaling pathway	3/132	60/18870	0.00856366	0.02044568	0.0093463	EDN1/MMP12/AXL	3
BP	GO:0002763	positive regulation of myeloid leukocyte differentiation	3/132	60/18870	0.00856366	0.02044568	0.0093463	FOS/TNF/RB1	3
BP	GO:0006695	cholesterol biosynthetic process	3/132	60/18870	0.00856366	0.02044568	0.0093463	HMGCR/CYP51A1/CFTR	3
BP	GO:0006767	water-soluble vitamin metabolic process	3/132	60/18870	0.00856366	0.02044568	0.0093463	AKR1B1/AKR1A1/DHFR	3
BP	GO:0010803	regulation of tumor necrosis factor-mediated signaling pathway	3/132	60/18870	0.00856366	0.02044568	0.0093463	ADIPOQ/XIAP/SYK	3

BP	GO:1902653	secondary alcohol biosynthetic process	3/132	60/18870	0.00856366	0.02044568	0.0093463	HMGCR/CYP51A1/CFTR	3
BP	GO:0042742	defense response to bacterium	7/132	330/18870	0.0086135	0.02055266	0.0093952	TNF/IL6R/XIAP/SYK/F2/MPO/PLA2G1B	7
BP	GO:0044344	cellular response to fibroblast growth factor stimulus	4/132	115/18870	0.00870103	0.02074939	0.00948513	CCL2/COL1A1/RUNX2/PTPN11	4
BP	GO:0043534	blood vessel endothelial cell migration	5/132	181/18870	0.00892948	0.02125695	0.00971715	TNF/CYP1B1/KDR/AKT1/PTGS2	5
BP	GO:0045580	regulation of T cell differentiation	5/132	181/18870	0.00892948	0.02125695	0.00971715	BAD/IL2RA/IL4R/SYK/IL2	5
BP	GO:1905475	regulation of protein localization to membrane	5/132	181/18870	0.00892948	0.02125695	0.00971715	AR/TNF/EGFR/PIK3R1/AKT1	5
BP	GO:0006940	regulation of smooth muscle contraction	3/132	61/18870	0.00896125	0.02126467	0.00972068	EDN1/CAV1/PTGS2	3
BP	GO:0010921	regulation of phosphatase activity	3/132	61/18870	0.00896125	0.02126467	0.00972068	TNF/GSK3B/NUAK1	3
BP	GO:0022029	telencephalon cell migration	3/132	61/18870	0.00896125	0.02126467	0.00972068	DRD2/COL3A1/EGFR	3
BP	GO:0034113	heterotypic cell-cell adhesion	3/132	61/18870	0.00896125	0.02126467	0.00972068	ADIPOQ/TNF/ITGB3	3
BP	GO:0048599	oocyte development	3/132	61/18870	0.00896125	0.02126467	0.00972068	BCL2/EDN1/EDNRA	3
BP	GO:0002062	chondrocyte differentiation	4/132	116/18870	0.00896396	0.02126467	0.00972068	RB1/COL3A1/RUNX2/PTPN11	4
BP	GO:0051607	defense response to virus	7/132	334/18870	0.00916766	0.02173528	0.00993581	HSP90AA1/RELA/BCL2/TNF/STAT1/CXCL10/MMP12	7
BP	GO:1901800	positive regulation of proteasomal protein catabolic process	4/132	117/18870	0.00923203	0.02187519	0.00999976	CAV1/GSK3B/PLK1/AKT1	4
BP	GO:0140546	defense response to symbiont	7/132	335/18870	0.0093102	0.02190959	0.01001549	HSP90AA1/RELA/BCL2/TNF/STAT1/CXCL10/MMP12	7
BP	GO:0046578	regulation of Ras protein signal transduction	5/132	183/18870	0.00933655	0.02190959	0.01001549	COL3A1/SRC/MET/GPR35/PIK3CG	5
BP	GO:0010288	response to lead ion	2/132	21/18870	0.00934845	0.02190959	0.01001549	MAPT/PTGS2	2
BP	GO:0032495	response to muramyl dipeptide	2/132	21/18870	0.00934845	0.02190959	0.01001549	RELA/NFKBIA	2
BP	GO:0033005	positive regulation of mast cell activation	2/132	21/18870	0.00934845	0.02190959	0.01001549	IL4R/SYK	2
BP	GO:0036480	neuron intrinsic apoptotic signaling pathway in response to oxidative stres	2/132	21/18870	0.00934845	0.02190959	0.01001549	MCL1/PARP1	2
BP	GO:0051988	regulation of attachment of spindle microtubules to kinetochore	2/132	21/18870	0.00934845	0.02190959	0.01001549	CCNB1/BIRC5	2
BP	GO:0060973	cell migration involved in heart development	2/132	21/18870	0.00934845	0.02190959	0.01001549	EDN1/EDNRA	2
BP	GO:0061318	renal filtration cell differentiation	2/132	21/18870	9.35E-03	2.19E-02	1.00E-02	ADIPOQ/EDNRA	2
BP	GO:0071379	cellular response to prostaglandin stimulus	2/132	21/18870	9.35E-03	2.19E-02	1.00E-02	AKT1/AKR1C3	2
BP	GO:0071605	monocyte chemotactic protein-1 production	2/132	21/18870	9.35E-03	2.19E-02	1.00E-02	ADIPOQ/SYK	2
BP	GO:0071637	regulation of monocyte chemotactic protein-1 production	2/132	21/18870	9.35E-03	2.19E-02	1.00E-02	ADIPOQ/SYK	2
BP	GO:0072112	podocyte differentiation	2/132	21/18870	9.35E-03	2.19E-02	1.00E-02	ADIPOQ/EDNRA	2
BP	GO:0090231	regulation of spindle checkpoint	2/132	21/18870	9.35E-03	2.19E-02	1.00E-02	CCNB1/BIRC5	2
BP	GO:0090266	regulation of mitotic cell cycle spindle assembly checkpoint	2/132	21/18870	9.35E-03	2.19E-02	1.00E-02	CCNB1/BIRC5	2
BP	GO:0097049	motor neuron apoptotic process	2/132	21/18870	9.35E-03	2.19E-02	1.00E-02	BCL2/BAX	2
BP	GO:1901739	regulation of myoblast fusion	2/132	21/18870	9.35E-03	2.19E-02	1.00E-02	IL4R/CXCL10	2
BP	GO:1903504	regulation of mitotic spindle checkpoint	2/132	21/18870	9.35E-03	2.19E-02	1.00E-02	CCNB1/BIRC5	2
BP	GO:2000010	positive regulation of protein localization to cell surface	2/132	21/18870	9.35E-03	2.19E-02	1.00E-02	TNF/AKT1	2
BP	GO:0000070	mitotic sister chromatid segregation	5/132	184/18870	9.54E-03	2.24E-02	1.02E-02	CDK1/CCNB1/RB1/BIRC5/PLK1	5
BP	GO:0031644	regulation of nervous system process	4/132	119/18870	9.78E-03	2.29E-02	1.05E-02	TNF/APP/ADORA1/GPR35	4
BP	GO:0051784	negative regulation of nuclear division	3/132	63/18870	9.79E-03	2.29E-02	1.05E-02	CCNB1/BIRC5/PLK1	3
BP	GO:0051893	regulation of focal adhesion assembly	3/132	63/18870	9.79E-03	2.29E-02	1.05E-02	SRC/PTK2/KDR	3
BP	GO:0070050	neuron cellular homeostasis	3/132	63/18870	9.79E-03	0.02286213	0.01045092	APP/CA2/ADORA1	3
BP	GO:0090109	regulation of cell-substrate junction assembly	3/132	63/18870	9.79E-03	0.02286213	0.01045092	SRC/PTK2/KDR	3
BP	GO:0051302	regulation of cell division	5/132	186/18870	9.97E-03	0.02327494	0.01063963	MYC/DRD2/BIRC5/IGF1R/PLK1	5
BP	GO:0007059	chromosome segregation	8/132	424/18870	9.99E-03	0.02329969	0.01065094	CDK1/CCNB1/RB1/BIRC5/TOP1/PLK1/CSNK2A1/TOP2.	8
BP	GO:0002718	regulation of cytokine production involved in immune response	4/132	120/18870	1.01E-02	0.02347345	0.01073037	TNF/SYK/ARG1/AXL	4
BP	GO:0008361	regulation of cell size	5/132	187/18870	1.02E-02	0.02366513	0.010818	HSP90AA1/EDN1/SPP1/GSK3B/MAPT	5
BP	GO:0001885	endothelial cell development	3/132	64/18870	1.02E-02	0.02366513	0.010818	TNF/EDNRA/MET	3
BP	GO:0035773	insulin secretion involved in cellular response to glucose stimulus	3/132	64/18870	1.02E-02	0.02366513	0.010818	BAD/KLF7/CFTR	3
BP	GO:0003085	negative regulation of systemic arterial blood pressure	2/132	22/18870	1.02E-02	0.02366513	0.010818	TNF/ADORA1	2
BP	GO:0006837	serotonin transport	2/132	22/18870	1.02E-02	0.02366513	0.010818	ITGB3/SYK	2
BP	GO:0007530	sex determination	2/132	22/18870	0.01023655	0.02366513	0.010818	AR/INSR	2
BP	GO:0032042	mitochondrial DNA metabolic process	2/132	22/18870	0.01023655	0.02366513	0.010818	TP53/PARP1	2

BP	GO:0033630	positive regulation of cell adhesion mediated by integrin	2/132	22/18870	0.01023655	0.02366513	0.010818	ITGB3/SYK	2
BP	GO:0035739	CD4-positive, alpha-beta T cell proliferation	2/132	22/18870	0.01023655	0.02366513	0.010818	IL2RA/IL2	2
BP	GO:0045821	positive regulation of glycolytic process	2/132	22/18870	0.01023655	0.02366513	0.010818	APP/INSR	2
BP	GO:0048169	regulation of long-term neuronal synaptic plasticity	2/132	22/18870	0.01023655	0.02366513	0.010818	DRD2/APP	2
BP	GO:0071305	cellular response to vitamin D	2/132	22/18870	0.01023655	0.02366513	0.010818	RXRA/PIM1	2
BP	GO:0072311	glomerular epithelial cell differentiation	2/132	22/18870	0.01023655	0.02366513	0.010818	ADIPOQ/EDNRA	2
BP	GO:0097062	dendritic spine maintenance	2/132	22/18870	0.01023655	0.02366513	0.010818	IGF1R/INSR	2
BP	GO:2000561	regulation of CD4-positive, alpha-beta T cell proliferation	2/132	22/18870	0.01023655	0.02366513	0.010818	IL2RA/IL2	2
BP	GO:0060828	regulation of canonical Wnt signaling pathway	6/132	262/18870	0.01035963	0.02393614	0.01094188	XIAP/CAV1/COL1A1/GSK3B/EGFR/SRC	6
BP	GO:0002367	cytokine production involved in immune response	4/132	122/18870	0.01065076	0.02454395	0.01121973	TNF/SYK/ARG1/AXL	4
BP	GO:0032874	positive regulation of stress-activated MAPK cascade	4/132	122/18870	0.01065076	0.02454395	0.01121973	TNF/APP/XIAP/XDH	4
BP	GO:0009994	oocyte differentiation	3/132	65/18870	0.01065875	0.02454395	0.01121973	BCL2/EDN1/EDNRA	3
BP	GO:0055021	regulation of cardiac muscle tissue growth	3/132	65/18870	0.01065875	0.02454395	0.01121973	CDK1/EDN1/PIM1	3
BP	GO:0070059	intrinsic apoptotic signaling pathway in response to endoplasmic reticulum	3/132	65/18870	0.01065875	0.02454395	0.01121973	BCL2/BAX/TP53	3
BP	GO:1902808	positive regulation of cell cycle G1/S phase transition	3/132	65/18870	0.01065875	0.02454395	0.01121973	EGFR/AKT1/TERT	3
BP	GO:0043010	camera-type eye development	7/132	344/18870	0.01066723	0.02454965	0.01122234	BAX/DRD2/STAT3/CYP1B1/EGFR/KDR/ACHE	7
BP	GO:0003007	heart morphogenesis	6/132	264/18870	0.0107272	0.02467377	0.01127907	TP53/EDN1/EDNRA/PIM1/PTK2/INSR	6
BP	GO:0071774	response to fibroblast growth factor	4/132	123/18870	0.01095044	0.02517307	0.01150732	CCL2/COL1A1/RUNX2/PTPN11	4
BP	GO:0000271	polysaccharide biosynthetic process	3/132	66/18870	0.01111016	0.02544125	0.01162991	GSK3B/AKT1/INSR	3
BP	GO:0042446	hormone biosynthetic process	3/132	66/18870	0.01111016	0.02544125	0.01162991	CYP19A1/AKR1B1/IGF1R	3
BP	GO:0002223	stimulatory C-type lectin receptor signaling pathway	2/132	23/18870	0.01116052	0.02544125	0.01162991	SYK/SRC	2
BP	GO:0002689	negative regulation of leukocyte chemotaxis	2/132	23/18870	0.01116052	0.02544125	0.01162991	CYP19A1/CCL2	2
BP	GO:0010893	positive regulation of steroid biosynthetic process	2/132	23/18870	0.01116052	0.02544125	0.01162991	TNF/IGF1R	2
BP	GO:0019430	removal of superoxide radicals	2/132	23/18870	0.01116052	0.02544125	0.01162991	MPO/DHFR	2
BP	GO:0036120	cellular response to platelet-derived growth factor stimulus	2/132	23/18870	0.01116052	0.02544125	0.01162991	ITGB3/SRC	2
BP	GO:0043371	negative regulation of CD4-positive, alpha-beta T cell differentiation	2/132	23/18870	0.01116052	0.02544125	0.01162991	IL4R/IL2	2
BP	GO:0043373	CD4-positive, alpha-beta T cell lineage commitment	2/132	23/18870	0.01116052	0.02544125	0.01162991	IL6R/STAT3	2
BP	GO:0045932	negative regulation of muscle contraction	2/132	23/18870	0.01116052	0.02544125	0.01162991	PIK3CG/PTGS2	2
BP	GO:0051590	positive regulation of neurotransmitter transport	2/132	23/18870	0.01116052	0.02544125	0.01162991	DRD2/ADORA2A	2
BP	GO:0060307	regulation of ventricular cardiac muscle cell membrane repolarization	2/132	23/18870	0.01116052	0.02544125	0.01162991	KCNH2/SCN5A	2
BP	GO:0072202	cell differentiation involved in metanephros development	2/132	23/18870	0.01116052	0.02544125	0.01162991	ADIPOQ/STAT1	2
BP	GO:1990840	response to lectin	2/132	23/18870	0.01116052	0.02544125	0.01162991	SYK/SRC	2
BP	GO:1990858	cellular response to lectin	2/132	23/18870	0.01116052	0.02544125	0.01162991	SYK/SRC	2
BP	GO:0006275	regulation of DNA replication	4/132	124/18870	0.01125552	0.02561492	0.0117093	TP53/CDK1/CDK2/EGFR	4
BP	GO:0070304	positive regulation of stress-activated protein kinase signaling cascade	4/132	124/18870	0.01125552	0.02561492	0.0117093	TNF/APP/XIAP/XDH	4
BP	GO:0072655	establishment of protein localization to mitochondrion	4/132	124/18870	0.01125552	0.02561492	0.0117093	HSP90AA1/BAX/AKT1/MAPT	4
BP	GO:0030258	lipid modification	5/132	192/18870	0.01132732	0.02576398	0.01177744	ADIPOQ/ALOX5/ALOX15/ALOX12/AKT1	5
BP	GO:0002822	regulation of adaptive immune response based on somatic recombination	5/132	193/18870	0.01156502	0.02619039	0.01197236	TNF/IL4R/ARG1/IL2/AHR	5
BP	GO:0006805	xenobiotic metabolic process	4/132	125/18870	0.01156602	0.02619039	0.01197236	CYP1B1/ABCC1/ABCB1/AHR	4
BP	GO:0006821	chloride transport	4/132	125/18870	0.01156602	0.02619039	0.01197236	CLDN4/CA2/ABCB1/CFTR	4
BP	GO:0006949	syncytium formation	3/132	67/18870	0.0115725	0.02619039	0.01197236	CYP19A1/IL4R/CXCL10	3
BP	GO:0009620	response to fungus	3/132	67/18870	0.0115725	0.02619039	0.01197236	SYK/ARG1/MPO	3
BP	GO:0010972	negative regulation of G2/M transition of mitotic cell cycle	3/132	67/18870	0.0115725	0.02619039	0.01197236	CDK1/CDKN1A/PLK1	3
BP	GO:0016126	sterol biosynthetic process	3/132	67/18870	0.0115725	0.02619039	0.01197236	HMGCR/CYP51A1/CFTR	3
BP	GO:0060760	positive regulation of response to cytokine stimulus	3/132	67/18870	0.0115725	0.02619039	0.01197236	EDN1/MMP12/AXL	3
BP	GO:0071300	cellular response to retinoic acid	3/132	67/18870	0.0115725	0.02619039	0.01197236	TNF/COL1A1/GSK3B	3
BP	GO:2000027	regulation of animal organ morphogenesis	4/132	126/18870	0.01188199	0.02687593	0.01228574	AR/TNF/EDN1/RUNX2	4
BP	GO:0002320	lymphoid progenitor cell differentiation	2/132	24/18870	0.01211982	0.02715824	0.0124148	BCL2/FLT3	2
BP	GO:0009162	deoxyribonucleoside monophosphate metabolic process	2/132	24/18870	0.01211982	0.02715824	0.0124148	XDH/TYMS	2

BP	GO:0010923	negative regulation of phosphatase activity	2/132	24/18870	0.01211982	0.02715824	0.0124148	TNF/GSK3B	2
BP	GO:0014821	phasic smooth muscle contraction	2/132	24/18870	0.01211982	0.02715824	0.0124148	DRD2/EDN1	2
BP	GO:0016093	polyprenol metabolic process	2/132	24/18870	0.01211982	0.02715824	0.0124148	AKR1B10/AKR1C3	2
BP	GO:0031145	anaphase-promoting complex-dependent catabolic process	2/132	24/18870	0.01211982	0.02715824	0.0124148	CDK2/PLK1	2
BP	GO:0032727	positive regulation of interferon-alpha production	2/132	24/18870	0.01211982	0.02715824	0.0124148	STAT1/MMP12	2
BP	GO:0034114	regulation of heterotypic cell-cell adhesion	2/132	24/18870	0.01211982	0.02715824	0.0124148	ADIPOQ/TNF	2
BP	GO:0035357	peroxisome proliferator activated receptor signaling pathway	2/132	24/18870	0.01211982	0.02715824	0.0124148	RXRA/ALOX15	2
BP	GO:0045723	positive regulation of fatty acid biosynthetic process	2/132	24/18870	0.01211982	0.02715824	0.0124148	NR1H3/PTGS2	2
BP	GO:0045736	negative regulation of cyclin-dependent protein serine/threonine kinase a	2/132	24/18870	0.01211982	0.02715824	0.0124148	CDKN1A/PLK1	2
BP	GO:0045822	negative regulation of heart contraction	2/132	24/18870	0.01211982	0.02715824	0.0124148	TNF/PIK3CG	2
BP	GO:0051043	regulation of membrane protein ectodomain proteolysis	2/132	24/18870	0.01211982	0.02715824	0.0124148	TIMP1/TNF	2
BP	GO:0060571	morphogenesis of an epithelial fold	2/132	24/18870	0.01211982	0.02715824	0.0124148	AR/EGFR	2
BP	GO:1904030	negative regulation of cyclin-dependent protein kinase activity	2/132	24/18870	0.01211982	0.02715824	0.0124148	CDKN1A/PLK1	2
BP	GO:2000353	positive regulation of endothelial cell apoptotic process	2/132	24/18870	0.01211982	0.02715824	0.0124148	CCL2/AKR1C3	2
BP	GO:2001026	regulation of endothelial cell chemotaxis	2/132	24/18870	0.01211982	0.02715824	0.0124148	KDR/MET	2
BP	GO:0021987	cerebral cortex development	4/132	127/18870	0.01220345	0.02733065	0.01249361	BAX/COL3A1/GSK3B/EGFR	4
BP	GO:0061136	regulation of proteasomal protein catabolic process	5/132	196/18870	0.01229848	0.02752837	0.01258399	CDK2/CAV1/GSK3B/PLK1/AKT1	5
BP	GO:0007517	muscle organ development	7/132	354/18870	0.01233822	0.0276022	0.01261774	BCL2/FOS/RB1/EDNRA/CAV1/COL3A1/CXCL10	7
BP	GO:0031346	positive regulation of cell projection organization	7/132	355/18870	0.01251514	0.0279557	0.01277934	HSP90AA1/GSK3B/IGF1R/PIK3R1/SRC/ALK/MAPT	7
BP	GO:0007004	telomere maintenance via telomerase	3/132	69/18870	0.01253007	0.0279557	0.01277934	HSP90AA1/SRC/TERT	3
BP	GO:0045576	mast cell activation	3/132	69/18870	0.01253007	0.0279557	0.01277934	IL4R/SYK/PIK3CG	3
BP	GO:0045670	regulation of osteoclast differentiation	3/132	69/18870	0.01253007	0.0279557	0.01277934	FOS/TNF/PIK3R1	3
BP	GO:0001704	formation of primary germ layer	4/132	128/18870	0.01253045	0.0279557	0.01277934	ITGB3/GJA1/MMP9/MMP2	4
BP	GO:0032922	circadian regulation of gene expression	3/132	70/18870	0.01302539	0.0289101	0.01321562	DRD2/TOP1/AHR	3
BP	GO:1902750	negative regulation of cell cycle G2/M phase transition	3/132	70/18870	0.01302539	0.0289101	0.01321562	CDK1/CDKN1A/PLK1	3
BP	GO:0002520	immune system development	5/132	199/18870	0.01306293	0.0289101	0.01321562	BCL2/TP53/IL2RA/IL2/TYR	5
BP	GO:0010623	programmed cell death involved in cell development	2/132	25/18870	0.01311393	0.0289101	0.01321562	BCL2/BAX	2
BP	GO:0014856	skeletal muscle cell proliferation	2/132	25/18870	0.01311393	0.0289101	0.01321562	FOS/SRC	2
BP	GO:0036119	response to platelet-derived growth factor	2/132	25/18870	0.01311393	0.0289101	0.01321562	ITGB3/SRC	2
BP	GO:0043302	positive regulation of leukocyte degranulation	2/132	25/18870	0.01311393	0.0289101	0.01321562	IL4R/SYK	2
BP	GO:0045649	regulation of macrophage differentiation	2/132	25/18870	0.01311393	0.0289101	0.01321562	ADIPOQ/RB1	2
BP	GO:0045662	negative regulation of myoblast differentiation	2/132	25/18870	0.01311393	0.0289101	0.01321562	TNF/CXCL10	2
BP	GO:0048011	neurotrophin TRK receptor signaling pathway	2/132	25/18870	0.01311393	0.0289101	0.01321562	SRC/PTPN11	2
BP	GO:0050927	positive regulation of positive chemotaxis	2/132	25/18870	0.01311393	0.0289101	0.01321562	KDR/F3	2
BP	GO:0071450	cellular response to oxygen radical	2/132	25/18870	0.01311393	0.0289101	0.01321562	MPO/DHFR	2
BP	GO:0071451	cellular response to superoxide	2/132	25/18870	0.01311393	0.0289101	0.01321562	MPO/DHFR	2
BP	GO:0071676	negative regulation of mononuclear cell migration	2/132	25/18870	0.01311393	0.0289101	0.01321562	CCL2/AKT1	2
BP	GO:0072010	glomerular epithelium development	2/132	25/18870	0.01311393	0.0289101	0.01321562	ADIPOQ/EDNRA	2
BP	GO:0072243	metanephric nephron epithelium development	2/132	25/18870	0.01311393	0.0289101	0.01321562	ADIPOQ/STAT1	2
BP	GO:0086064	cell communication by electrical coupling involved in cardiac conduction	2/132	25/18870	0.01311393	0.0289101	0.01321562	CAV1/GJA1	2
BP	GO:0090335	regulation of brown fat cell differentiation	2/132	25/18870	0.01311393	0.0289101	0.01321562	PIM1/PTGS2	2
BP	GO:1902894	negative regulation of miRNA transcription	2/132	25/18870	0.01311393	0.0289101	0.01321562	RELA/ESR1	2
BP	GO:1902993	positive regulation of amyloid precursor protein catabolic process	2/132	25/18870	0.01311393	0.0289101	0.01321562	RELA/TNF	2
BP	GO:1903523	negative regulation of blood circulation	2/132	25/18870	0.01311393	0.0289101	0.01321562	TNF/PIK3CG	2
BP	GO:2000773	negative regulation of cellular senescence	2/132	25/18870	0.01311393	0.0289101	0.01321562	TERT/CDK6	2
BP	GO:0032612	interleukin-1 production	4/132	130/18870	0.01320115	0.029071	0.01328917	RELA/TNF/APP/STAT3	4
BP	GO:0032652	regulation of interleukin-1 production	4/132	130/18870	0.01320115	0.029071	0.01328917	RELA/TNF/APP/STAT3	4
BP	GO:0007281	germ cell development	7/132	359/18870	0.01324119	0.02914347	0.0133223	BCL2/BAX/EDN1/EDNRA/SRC/AKT1/CFTR	7
BP	GO:0090596	sensory organ morphogenesis	6/132	277/18870	0.01334516	0.02935648	0.01341967	BCL2/BAX/STAT3/EDN1/EDNRA/KDR	6

BP	GO:0002548	monocyte chemotaxis	3/132	71/18870	0.01353177	0.0297159	0.01358397	IL6R/CCL2/CXCL10	3
BP	GO:0060420	regulation of heart growth	3/132	71/18870	0.01353177	0.0297159	0.01358397	CDK1/EDN1/PIM1	3
BP	GO:0019079	viral genome replication	4/132	131/18870	0.01354492	0.0297159	0.01358397	BCL2/TNF/CCL2/TOP2A	4
BP	GO:0030218	erythrocyte differentiation	4/132	131/18870	0.01354492	0.0297159	0.01358397	RB1/STAT3/STAT1/CDK6	4
BP	GO:0070585	protein localization to mitochondrion	4/132	131/18870	0.01354492	0.0297159	0.01358397	HSP90AA1/BAX/AKT1/MAPT	4
BP	GO:0006260	DNA replication	6/132	278/18870	0.0135635	0.02974068	0.0135953	TP53/CDK1/CDK2/PARP1/TOP1/EGFR	6
BP	GO:0032388	positive regulation of intracellular transport	5/132	201/18870	0.01359005	0.02978292	0.01361461	HSP90AA1/CDK1/GSK3B/PIK3R1/PTGS2	5
BP	GO:1903322	positive regulation of protein modification by small protein conjugation or	4/132	132/18870	0.01389435	0.03043346	0.01391199	XIAP/CAV1/GSK3B/PLK1	4
BP	GO:0043954	cellular component maintenance	3/132	72/18870	0.01404926	0.03071313	0.01403983	ITGB3/IGF1R/INSR	3
BP	GO:0060260	regulation of transcription initiation by RNA polymerase II	3/132	72/18870	0.01404926	0.03071313	0.01403983	TP53/CDK4/NFKBIA	3
BP	GO:0006470	protein dephosphorylation	5/132	203/18870	0.01413136	0.03071313	0.01403983	TNF/ADORA1/GSK3B/NUAK1/PTPN11	5
BP	GO:0120032	regulation of plasma membrane bounded cell projection assembly	5/132	203/18870	0.01413136	0.03071313	0.01403983	HSP90AA1/CAV1/GSK3B/PIK3R1/AKT1	5
BP	GO:0002407	dendritic cell chemotaxis	2/132	26/18870	0.01414232	0.03071313	0.01403983	CXCR1/PIK3CG	2
BP	GO:0006582	melanin metabolic process	2/132	26/18870	0.01414232	0.03071313	0.01403983	BCL2/TYR	2
BP	GO:0006760	folic acid-containing compound metabolic process	2/132	26/18870	0.01414232	0.03071313	0.01403983	TYMS/DHFR	2
BP	GO:0014829	vascular associated smooth muscle contraction	2/132	26/18870	0.01414232	0.03071313	0.01403983	EDN1/EDNRA	2
BP	GO:0018904	ether metabolic process	2/132	26/18870	0.01414232	0.03071313	0.01403983	ALOX5/ALOX12	2
BP	GO:0050926	regulation of positive chemotaxis	2/132	26/18870	0.01414232	0.03071313	0.01403983	KDR/F3	2
BP	GO:0060343	trabecula formation	2/132	26/18870	0.01414232	0.03071313	0.01403983	COL1A1/MMP2	2
BP	GO:0062149	detection of stimulus involved in sensory perception of pain	2/132	26/18870	0.01414232	0.03071313	0.01403983	TNF/ADORA1	2
BP	GO:1900407	regulation of cellular response to oxidative stress	2/132	26/18870	0.01414232	0.03071313	0.01403983	ALOX5/DHFR	2
BP	GO:1901623	regulation of lymphocyte chemotaxis	2/132	26/18870	0.01414232	0.03071313	0.01403983	CCL2/CXCL10	2
BP	GO:2000629	negative regulation of miRNA metabolic process	2/132	26/18870	0.01414232	0.03071313	0.01403983	RELA/ESR1	2
BP	GO:2000737	negative regulation of stem cell differentiation	2/132	26/18870	0.01414232	0.03071313	0.01403983	STAT3/GSK3B	2
BP	GO:0048568	embryonic organ development	8/132	453/18870	0.01441635	0.03129159	0.01430427	TP53/TNF/EDN1/EDNRA/RUNX2/EGFR/KDR/AKT1	8
BP	GO:0033143	regulation of intracellular steroid hormone receptor signaling pathway	3/132	73/18870	0.01457789	0.03159187	0.01444153	AR/PARP1/SRC	3
BP	GO:0140962	multicellular organismal-level chemical homeostasis	3/132	73/18870	0.01457789	0.03159187	0.01444153	AKR1B1/KDR/CFTR	3
BP	GO:1903351	cellular response to dopamine	3/132	73/18870	0.01457789	0.03159187	0.01444153	DRD2/GSK3B/ALK	3
BP	GO:0050851	antigen receptor-mediated signaling pathway	5/132	205/18870	0.01468701	0.03181147	0.01454192	RELA/BCL2/BAX/NFKBIA/SYK	5
BP	GO:0007585	respiratory gaseous exchange by respiratory system	3/132	74/18870	0.01511767	0.03269226	0.01494455	EDN1/EDNRA/ADORA1	3
BP	GO:0046637	regulation of alpha-beta T cell differentiation	3/132	74/18870	0.01511767	0.03269226	0.01494455	IL4R/SYK/IL2	3
BP	GO:1903350	response to dopamine	3/132	74/18870	0.01511767	0.03269226	0.01494455	DRD2/GSK3B/ALK	3
BP	GO:0001964	startle response	2/132	27/18870	0.01520447	0.03270682	0.01495121	DRD2/ADORA2A	2
BP	GO:0006007	glucose catabolic process	2/132	27/18870	0.01520447	0.03270682	0.01495121	TP53/BAD	2
BP	GO:0010804	negative regulation of tumor necrosis factor-mediated signaling pathway	2/132	27/18870	0.01520447	0.03270682	0.01495121	ADIPOQ/XIAP	2
BP	GO:0042104	positive regulation of activated T cell proliferation	2/132	27/18870	0.01520447	0.03270682	0.01495121	IL2RA/IL2	2
BP	GO:0045672	positive regulation of osteoclast differentiation	2/132	27/18870	0.01520447	0.03270682	0.01495121	FOS/TNF	2
BP	GO:0045940	positive regulation of steroid metabolic process	2/132	27/18870	0.01520447	0.03270682	0.01495121	TNF/IGF1R	2
BP	GO:0060384	innervation	2/132	27/18870	0.01520447	0.03270682	0.01495121	EDN1/EDNRA	2
BP	GO:0071480	cellular response to gamma radiation	2/132	27/18870	0.01520447	0.03270682	0.01495121	TP53/CDKN1A	2
BP	GO:0090330	regulation of platelet aggregation	2/132	27/18870	0.01520447	0.03270682	0.01495121	SYK/ALOX12	2
BP	GO:1904357	negative regulation of telomere maintenance via telomere lengthening	2/132	27/18870	0.01520447	0.03270682	0.01495121	PARP1/SRC	2
BP	GO:0051321	meiotic cell cycle	6/132	287/18870	0.01564231	0.03363096	0.01537366	CDK2/EDN1/EDNRA/PLK1/TOP2A/INSR	6
BP	GO:0006278	RNA-templated DNA biosynthetic process	3/132	75/18870	0.01566864	0.03365216	0.01538335	HSP90AA1/SRC/TERT	3
BP	GO:0030968	endoplasmic reticulum unfolded protein response	3/132	75/18870	0.01566864	0.03365216	0.01538335	BAX/CCND1/PIK3R1	3
BP	GO:0006986	response to unfolded protein	4/132	137/18870	0.0157273	0.03376039	0.01543283	HSP90AA1/BAX/CCND1/PIK3R1	4
BP	GO:0005977	glycogen metabolic process	3/132	76/18870	0.01623082	0.0345716	0.01580365	GSK3B/AKT1/INSR	3
BP	GO:0043536	positive regulation of blood vessel endothelial cell migration	3/132	76/18870	0.01623082	0.0345716	0.01580365	KDR/AKT1/PTGS2	3
BP	GO:0000303	response to superoxide	2/132	28/18870	0.01629986	0.0345716	0.01580365	MPO/DHFR	2

BP	GO:0003094	glomerular filtration	2/132	28/18870	0.01629986	0.0345716	0.01580365	EDNRA/ADORA1	2
BP	GO:0010592	positive regulation of lamellipodium assembly	2/132	28/18870	0.01629986	0.0345716	0.01580365	HSP90AA1/PIK3R1	2
BP	GO:0010758	regulation of macrophage chemotaxis	2/132	28/18870	0.01629986	0.0345716	0.01580365	CYP19A1/PTK2	2
BP	GO:0015732	prostaglandin transport	2/132	28/18870	0.01629986	0.0345716	0.01580365	EDN1/PTGS2	2
BP	GO:0030194	positive regulation of blood coagulation	2/132	28/18870	0.01629986	0.0345716	0.01580365	F2/F3	2
BP	GO:0030325	adrenal gland development	2/132	28/18870	0.01629986	0.0345716	0.01580365	CYP1B1/INSR	2
BP	GO:0035308	negative regulation of protein dephosphorylation	2/132	28/18870	0.01629986	0.0345716	0.01580365	TNF/GSK3B	2
BP	GO:0042634	regulation of hair cycle	2/132	28/18870	0.01629986	0.0345716	0.01580365	TNF/TERT	2
BP	GO:0042832	defense response to protozoan	2/132	28/18870	0.01629986	0.0345716	0.01580365	IL4R/ARG1	2
BP	GO:0044342	type B pancreatic cell proliferation	2/132	28/18870	0.01629986	0.0345716	0.01580365	BAD/CDK4	2
BP	GO:0046639	negative regulation of alpha-beta T cell differentiation	2/132	28/18870	0.01629986	0.0345716	0.01580365	IL4R/IL2	2
BP	GO:0048668	collateral sprouting	2/132	28/18870	0.01629986	0.0345716	0.01580365	APP/SPP1	2
BP	GO:0051873	killing by host of symbiont cells	2/132	28/18870	0.01629986	0.0345716	0.01580365	ARG1/F2	2
BP	GO:0060964	regulation of miRNA-mediated gene silencing	2/132	28/18870	0.01629986	0.0345716	0.01580365	TP53/STAT3	2
BP	GO:0086010	membrane depolarization during action potential	2/132	28/18870	0.01629986	0.0345716	0.01580365	KCNH2/SCN5A	2
BP	GO:0099623	regulation of cardiac muscle cell membrane repolarization	2/132	28/18870	0.01629986	0.0345716	0.01580365	KCNH2/SCN5A	2
BP	GO:0099625	ventricular cardiac muscle cell membrane repolarization	2/132	28/18870	0.01629986	0.0345716	0.01580365	KCNH2/SCN5A	2
BP	GO:0120255	olefinic compound biosynthetic process	2/132	28/18870	0.01629986	0.0345716	0.01580365	CYP19A1/AKR1C3	2
BP	GO:1900048	positive regulation of hemostasis	2/132	28/18870	0.01629986	0.0345716	0.01580365	F2/F3	2
BP	GO:2000108	positive regulation of leukocyte apoptotic process	2/132	28/18870	0.01629986	0.0345716	0.01580365	BAX/TP53	2
BP	GO:0072659	protein localization to plasma membrane	6/132	290/18870	0.01638179	0.03472736	0.01587485	AR/ADIPOQ/TNF/EGFR/PIK3R1/AKT1	6
BP	GO:0002637	regulation of immunoglobulin production	3/132	77/18870	0.01680423	0.03558595	0.01626734	TNF/IL4R/IL2	3
BP	GO:0051205	protein insertion into membrane	3/132	77/18870	0.01680423	0.03558595	0.01626734	HSP90AA1/BAX/EGFR	3
BP	GO:0008203	cholesterol metabolic process	4/132	140/18870	0.01689674	0.03574479	0.01633995	APP/HMGCR/CYP51A1/CFTR	4
BP	GO:0008360	regulation of cell shape	4/132	140/18870	0.01689674	0.03574479	0.01633995	CCL2/F2/PTK2/KDR	4
BP	GO:0002285	lymphocyte activation involved in immune response	5/132	213/18870	0.01705608	0.0360632	0.0164855	TP53/IL6R/IL4R/STAT3/IL2	5
BP	GO:0045216	cell-cell junction organization	5/132	214/18870	0.017369	0.03654686	0.0167066	TNF/CAV1/GJA1/CLDN4/SRC	5
BP	GO:0019233	sensory perception of pain	3/132	78/18870	0.01738889	0.03654686	0.0167066	TNF/CCL2/ADORA1	3
BP	GO:0044042	glucan metabolic process	3/132	78/18870	0.01738889	0.03654686	0.0167066	GSK3B/AKT1/INSR	3
BP	GO:0045921	positive regulation of exocytosis	3/132	78/18870	0.01738889	0.03654686	0.0167066	IL4R/SYK/CFTR	3
BP	GO:1901568	fatty acid derivative metabolic process	3/132	78/18870	0.01738889	0.03654686	0.0167066	ALOX5/ALOX15/ALOX12	3
BP	GO:2000514	regulation of CD4-positive, alpha-beta T cell activation	3/132	78/18870	0.01738889	0.03654686	0.0167066	IL2RA/IL4R/IL2	3
BP	GO:2000736	regulation of stem cell differentiation	3/132	78/18870	0.01738889	0.03654686	0.0167066	STAT3/GSK3B/CDK6	3
BP	GO:0000305	response to oxygen radical	2/132	29/18870	0.01742798	0.03654686	0.0167066	MPO/DHFR	2
BP	GO:0002710	negative regulation of T cell mediated immunity	2/132	29/18870	0.01742798	0.03654686	0.0167066	ARG1/AHR	2
BP	GO:0008299	isoprenoid biosynthetic process	2/132	29/18870	0.01742798	0.03654686	0.0167066	AKR1C3/HMGCR	2
BP	GO:0010165	response to X-ray	2/132	29/18870	0.01742798	0.03654686	0.0167066	TP53/CDKN1A	2
BP	GO:0010800	positive regulation of peptidyl-threonine phosphorylation	2/132	29/18870	0.01742798	0.03654686	0.0167066	APP/PLK1	2
BP	GO:0060037	pharyngeal system development	2/132	29/18870	0.01742798	0.03654686	0.0167066	EDN1/EDNRA	2
BP	GO:0072207	metanephric epithelium development	2/132	29/18870	0.01742798	0.03654686	0.0167066	ADIPOQ/STAT1	2
BP	GO:1900368	regulation of post-transcriptional gene silencing by regulatory ncRNA	2/132	29/18870	0.01742798	0.03654686	0.0167066	TP53/STAT3	2
BP	GO:1905820	positive regulation of chromosome separation	2/132	29/18870	0.01742798	0.03654686	0.0167066	RB1/BIRC5	2
BP	GO:0002460	adaptive immune response based on somatic recombination of immune re	7/132	380/18870	0.0175576	0.03679979	0.01682222	TNF/IL6R/IL4R/STAT3/ARG1/IL2/AHR	7
BP	GO:0045685	regulation of glial cell differentiation	3/132	79/18870	0.01798482	0.03765659	0.01721388	RELA/CDK1/F2	3
BP	GO:0072088	nephron epithelium morphogenesis	3/132	79/18870	0.01798482	0.03765659	0.01721388	BCL2/MYC/STAT1	3
BP	GO:1903900	regulation of viral life cycle	4/132	143/18870	0.01811929	0.03791869	0.0173337	BCL2/TNF/AXL/TOP2A	4
BP	GO:0050821	protein stabilization	5/132	217/18870	0.01833056	0.03834119	0.01752684	HSP90AA1/TP53/PIM1/PIK3R1/CSNK2A1	5
BP	GO:0000002	mitochondrial genome maintenance	2/132	30/18870	0.01858832	0.03847439	0.01758773	TP53/PARP1	2
BP	GO:0010039	response to iron ion	2/132	30/18870	0.01858832	0.03847439	0.01758773	BCL2/DRD2	2

BP	GO:0030262	apoptotic nuclear changes	2/132	30/18870	0.01858832	0.03847439	0.01758773	BAX/TOP2A	2
BP	GO:0032607	interferon-alpha production	2/132	30/18870	0.01858832	0.03847439	0.01758773	STAT1/MMP12	2
BP	GO:0032647	regulation of interferon-alpha production	2/132	30/18870	0.01858832	0.03847439	0.01758773	STAT1/MMP12	2
BP	GO:0033137	negative regulation of peptidyl-serine phosphorylation	2/132	30/18870	0.01858832	0.03847439	0.01758773	BAX/CAV1	2
BP	GO:0035774	positive regulation of insulin secretion involved in cellular response to gluc	2/132	30/18870	0.01858832	0.03847439	0.01758773	BAD/CFTR	2
BP	GO:0043304	regulation of mast cell degranulation	2/132	30/18870	0.01858832	0.03847439	0.01758773	IL4R/SYK	2
BP	GO:0045987	positive regulation of smooth muscle contraction	2/132	30/18870	0.01858832	0.03847439	0.01758773	EDN1/PTGS2	2
BP	GO:0048679	regulation of axon regeneration	2/132	30/18870	0.01858832	0.03847439	0.01758773	SPP1/IGF1R	2
BP	GO:0050820	positive regulation of coagulation	2/132	30/18870	0.01858832	0.03847439	0.01758773	F2/F3	2
BP	GO:0060045	positive regulation of cardiac muscle cell proliferation	2/132	30/18870	0.01858832	0.03847439	0.01758773	CDK1/PIM1	2
BP	GO:0060142	regulation of syncytium formation by plasma membrane fusion	2/132	30/18870	0.01858832	0.03847439	0.01758773	IL4R/CXCL10	2
BP	GO:0060147	regulation of post-transcriptional gene silencing	2/132	30/18870	0.01858832	0.03847439	0.01758773	TP53/STAT3	2
BP	GO:0061098	positive regulation of protein tyrosine kinase activity	2/132	30/18870	0.01858832	0.03847439	0.01758773	NOX4/SRC	2
BP	GO:0090140	regulation of mitochondrial fission	2/132	30/18870	0.01858832	0.03847439	0.01758773	KDR/MAPT	2
BP	GO:0097205	renal filtration	2/132	30/18870	0.01858832	0.03847439	0.01758773	EDNRA/ADORA1	2
BP	GO:1900101	regulation of endoplasmic reticulum unfolded protein response	2/132	30/18870	0.01858832	0.03847439	0.01758773	BAX/PIK3R1	2
BP	GO:1901380	negative regulation of potassium ion transmembrane transport	2/132	30/18870	0.01858832	0.03847439	0.01758773	KCNH2/CAV1	2
BP	GO:0048844	artery morphogenesis	3/132	80/18870	0.01859203	0.03847439	0.01758773	EDN1/EDNRA/COL3A1	3
BP	GO:2000142	regulation of DNA-templated transcription initiation	3/132	80/18870	0.01859203	0.03847439	0.01758773	TP53/CDK4/NFKBIA	3
BP	GO:1902115	regulation of organelle assembly	5/132	218/18870	0.01865874	0.03859289	0.0176419	TNF/GSK3B/SRC/PLK1/AKT1	5
BP	GO:0046165	alcohol biosynthetic process	4/132	145/18870	0.01896414	0.03920472	0.01792158	HMGCR/CYP51A1/CFTR/DHFR	4
BP	GO:0007422	peripheral nervous system development	3/132	81/18870	0.01921053	0.03961385	0.0181086	RELA/CDK1/AKT1	3
BP	GO:0042440	pigment metabolic process	3/132	81/18870	0.01921053	0.03961385	0.0181086	BCL2/ABCC1/TYR	3
BP	GO:0043299	leukocyte degranulation	3/132	81/18870	0.01921053	0.03961385	0.0181086	IL4R/SYK/PIK3CG	3
BP	GO:0050810	regulation of steroid biosynthetic process	3/132	81/18870	0.01921053	0.03961385	0.0181086	TNF/IGF1R/AKR1C3	3
BP	GO:0072028	nephron morphogenesis	3/132	81/18870	0.01921053	0.03961385	0.0181086	BCL2/MYC/STAT1	3
BP	GO:0016311	dephosphorylation	6/132	302/18870	0.01958178	0.04035901	0.01844924	TNF/ADORA1/GSK3B/SRC/NUAK1/PTPN11	6
BP	GO:0002697	regulation of immune effector process	7/132	389/18870	0.01968168	0.04040147	0.01846865	TNF/IL4R/SYK/ARG1/AXL/IL2/AHR	7
BP	GO:1902903	regulation of supramolecular fiber organization	7/132	389/18870	0.01968168	0.04040147	0.01846865	RB1/APP/EDN1/PIK3R1/ALOX15/MET/MAPT	7
BP	GO:0001562	response to protozoan	2/132	31/18870	0.0197804	0.04040147	0.01846865	IL4R/ARG1	2
BP	GO:0001945	lymph vessel development	2/132	31/18870	0.0197804	0.04040147	0.01846865	SYK/KDR	2
BP	GO:0007271	synaptic transmission, cholinergic	2/132	31/18870	0.0197804	0.04040147	0.01846865	ADORA2A/ACHE	2
BP	GO:0008053	mitochondrial fusion	2/132	31/18870	0.0197804	0.04040147	0.01846865	BAX/MCL1	2
BP	GO:0010996	response to auditory stimulus	2/132	31/18870	0.0197804	0.04040147	0.01846865	DRD2/CXCL10	2
BP	GO:0030878	thyroid gland development	2/132	31/18870	0.0197804	0.04040147	0.01846865	EDN1/EDNRA	2
BP	GO:0034143	regulation of toll-like receptor 4 signaling pathway	2/132	31/18870	0.0197804	0.04040147	0.01846865	PIK3R1/NR1H3	2
BP	GO:0034505	tooth mineralization	2/132	31/18870	0.0197804	0.04040147	0.01846865	COL1A1/CFTR	2
BP	GO:0035767	endothelial cell chemotaxis	2/132	31/18870	0.0197804	0.04040147	0.01846865	KDR/MET	2
BP	GO:0040018	positive regulation of multicellular organism growth	2/132	31/18870	0.0197804	0.04040147	0.01846865	BCL2/DRD2	2
BP	GO:0043507	positive regulation of JUN kinase activity	2/132	31/18870	0.0197804	0.04040147	0.01846865	TNF/EDN1	2
BP	GO:0060966	regulation of gene silencing by regulatory ncRNA	2/132	31/18870	0.0197804	0.04040147	0.01846865	TP53/STAT3	2
BP	GO:0086011	membrane repolarization during action potential	2/132	31/18870	0.0197804	0.04040147	0.01846865	KCNH2/CAV1	2
BP	GO:0150117	positive regulation of cell-substrate junction organization	2/132	31/18870	0.0197804	0.04040147	0.01846865	PIK3R1/KDR	2
BP	GO:1900017	positive regulation of cytokine production involved in inflammatory respor	2/132	31/18870	0.0197804	0.04040147	0.01846865	TNF/STAT3	2
BP	GO:2000273	positive regulation of signaling receptor activity	2/132	31/18870	0.0197804	0.04040147	0.01846865	EDN1/CCL2	2
BP	GO:0098656	monoatomic anion transmembrane transport	4/132	147/18870	0.01983307	0.04048341	0.01850611	CLDN4/ABCC1/ABCB1/CFTR	4
BP	GO:0060337	type I interferon-mediated signaling pathway	3/132	82/18870	0.01984034	0.04048341	0.01850611	STAT1/MMP12/PTPN11	3
BP	GO:0006119	oxidative phosphorylation	4/132	148/18870	0.02027662	0.04135297	0.0189036	CDK1/CCNB1/CYCS/TNF	4
BP	GO:0001570	vasculogenesis	3/132	83/18870	0.02048146	0.04168748	0.01905652	CAV1/XDH/KDR	3

BP	GO:0035023	regulation of Rho protein signal transduction	3/132	83/18870	0.02048146	0.04168748	0.01905652	COL3A1/MET/GPR35	3
BP	GO:0071357	cellular response to type I interferon	3/132	83/18870	0.02048146	0.04168748	0.01905652	STAT1/MMP12/PTPN11	3
BP	GO:1902117	positive regulation of organelle assembly	3/132	83/18870	0.02048146	0.04168748	0.01905652	TNF/GSK3B/SRC	3
BP	GO:0002230	positive regulation of defense response to virus by host	2/132	32/18870	0.02100371	0.04234954	0.01935917	HSP90AA1/STAT1	2
BP	GO:0006308	DNA catabolic process	2/132	32/18870	0.02100371	0.04234954	0.01935917	BAX/APEX1	2
BP	GO:0006921	cellular component disassembly involved in execution phase of apoptosis	2/132	32/18870	0.02100371	0.04234954	0.01935917	BAX/TOP2A	2
BP	GO:0007435	salivary gland morphogenesis	2/132	32/18870	0.02100371	0.04234954	0.01935917	TNF/EGFR	2
BP	GO:0010644	cell communication by electrical coupling	2/132	32/18870	0.02100371	0.04234954	0.01935917	CAV1/GJA1	2
BP	GO:0010765	positive regulation of sodium ion transport	2/132	32/18870	0.02100371	0.04234954	0.01935917	SCN5A/AKT1	2
BP	GO:0016242	negative regulation of macroautophagy	2/132	32/18870	0.02100371	0.04234954	0.01935917	TP53/AKT1	2
BP	GO:0031116	positive regulation of microtubule polymerization	2/132	32/18870	0.02100371	0.04234954	0.01935917	MET/MAPT	2
BP	GO:0033006	regulation of mast cell activation involved in immune response	2/132	32/18870	0.02100371	0.04234954	0.01935917	IL4R/SYK	2
BP	GO:0035590	purinergic nucleotide receptor signaling pathway	2/132	32/18870	0.02100371	0.04234954	0.01935917	ADORA1/ADORA2A	2
BP	GO:0042558	pteridine-containing compound metabolic process	2/132	32/18870	0.02100371	0.04234954	0.01935917	TYMS/DHFR	2
BP	GO:0045907	positive regulation of vasoconstriction	2/132	32/18870	0.02100371	0.04234954	0.01935917	CAV1/PTGS2	2
BP	GO:0046685	response to arsenic-containing substance	2/132	32/18870	0.02100371	0.04234954	0.01935917	CDKN1A/CYP1B1	2
BP	GO:0048384	retinoic acid receptor signaling pathway	2/132	32/18870	0.02100371	0.04234954	0.01935917	RXRA/AKR1C3	2
BP	GO:0060292	long-term synaptic depression	2/132	32/18870	0.02100371	0.04234954	0.01935917	ADORA1/MAPT	2
BP	GO:0070498	interleukin-1-mediated signaling pathway	2/132	32/18870	0.02100371	0.04234954	0.01935917	RELA/NFKBIA	2
BP	GO:0070570	regulation of neuron projection regeneration	2/132	32/18870	0.02100371	0.04234954	0.01935917	SPP1/IGF1R	2
BP	GO:1901020	negative regulation of calcium ion transmembrane transporter activity	2/132	32/18870	0.02100371	0.04234954	0.01935917	DRD2/GPR35	2
BP	GO:1901889	negative regulation of cell junction assembly	2/132	32/18870	0.02100371	0.04234954	0.01935917	TNF/SRC	2
BP	GO:0032024	positive regulation of insulin secretion	3/132	84/18870	0.0211339	0.04257001	0.01945995	BAD/F2/CFTR	3
BP	GO:0060395	SMAD protein signal transduction	3/132	84/18870	0.0211339	0.04257001	0.01945995	FOS/RUNX2/PARP1	3
BP	GO:0006865	amino acid transport	4/132	150/18870	0.02118198	0.04260383	0.01947541	TNF/GJA1/ADORA1/ADORA2A	4
BP	GO:0016525	negative regulation of angiogenesis	4/132	150/18870	0.02118198	0.04260383	0.01947541	TNF/STAT1/CXCL10/ALOX5	4
BP	GO:1902652	secondary alcohol metabolic process	4/132	150/18870	0.02118198	0.04260383	0.01947541	APP/HMGCR/CYP51A1/CFTR	4
BP	GO:0031331	positive regulation of cellular catabolic process	7/132	396/18870	0.02145383	0.04312937	0.01971565	BAX/BAD/TNF/APP/GSK3B/KDR/INSR	7
BP	GO:0002028	regulation of sodium ion transport	3/132	85/18870	0.02179766	0.04373451	0.01999227	SCN5A/DRD2/AKT1	3
BP	GO:0045069	regulation of viral genome replication	3/132	85/18870	0.02179766	0.04373451	0.01999227	BCL2/TNF/TOP2A	3
BP	GO:0051101	regulation of DNA binding	3/132	85/18870	0.02179766	0.04373451	0.01999227	RB1/NFKBIA/MMP9	3
BP	GO:0061515	myeloid cell development	3/132	85/18870	0.02179766	0.04373451	0.01999227	APP/SRC/PTPN11	3
BP	GO:2000181	negative regulation of blood vessel morphogenesis	4/132	152/18870	0.02211182	0.04434304	0.02027045	TNF/STAT1/CXCL10/ALOX5	4
BP	GO:0001556	oocyte maturation	2/132	33/18870	0.02225777	0.04441769	0.02030458	EDN1/EDNRA	2
BP	GO:0002862	negative regulation of inflammatory response to antigenic stimulus	2/132	33/18870	0.02225777	0.04441769	0.02030458	SYK/SRC	2
BP	GO:0003382	epithelial cell morphogenesis	2/132	33/18870	0.02225777	0.04441769	0.02030458	AR/MET	2
BP	GO:0033032	regulation of myeloid cell apoptotic process	2/132	33/18870	0.02225777	0.04441769	0.02030458	BCL2/ADIPOQ	2
BP	GO:0034368	protein-lipid complex remodeling	2/132	33/18870	0.02225777	0.04441769	0.02030458	MTTP/MPO	2
BP	GO:0034369	plasma lipoprotein particle remodeling	2/132	33/18870	0.02225777	0.04441769	0.02030458	MTTP/MPO	2
BP	GO:0045648	positive regulation of erythrocyte differentiation	2/132	33/18870	0.02225777	0.04441769	0.02030458	STAT3/STAT1	2
BP	GO:0050891	multicellular organismal-level water homeostasis	2/132	33/18870	0.02225777	0.04441769	0.02030458	AKR1B1/CFTR	2
BP	GO:0051497	negative regulation of stress fiber assembly	2/132	33/18870	0.02225777	0.04441769	0.02030458	PIK3R1/MET	2
BP	GO:1990806	ligand-gated ion channel signaling pathway	2/132	33/18870	0.02225777	0.04441769	0.02030458	APP/F2	2
BP	GO:0032418	lysosome localization	3/132	86/18870	0.02247275	0.04478107	0.02047069	IL4R/SYK/PIK3CG	3
BP	GO:0070918	regulatory ncRNA processing	3/132	86/18870	0.02247275	0.04478107	0.02047069	TP53/STAT3/TERT	3
BP	GO:1990849	vacuolar localization	3/132	86/18870	0.02247275	0.04478107	0.02047069	IL4R/SYK/PIK3CG	3
BP	GO:0098813	nuclear chromosome segregation	6/132	312/18870	0.02255539	0.04492384	0.02053595	CDK1/CCNB1/RB1/BIRC5/PLK1/TOP2A	6
BP	GO:0043535	regulation of blood vessel endothelial cell migration	4/132	153/18870	0.02258596	0.0449409	0.02054375	TNF/KDR/AKT1/PTGS2	4
BP	GO:1901343	negative regulation of vasculature development	4/132	153/18870	0.02258596	0.0449409	0.02054375	TNF/STAT1/CXCL10/ALOX5	4

BP	GO:0072521	purine-containing compound metabolic process	8/132	493/18870	0.02267681	0.04509969	0.02061634	TP53/BAD/SULT1E1/ABCG2/XDH/PARP1/TTR/HMGCR	8
BP	GO:0048705	skeletal system morphogenesis	5/132	230/18870	0.0229024	0.04552619	0.0208113	COL1A1/COL3A1/RUNX2/MMP2/MMP13	5
BP	GO:0006352	DNA-templated transcription initiation	4/132	154/18870	0.02306629	0.04582966	0.02095003	TP53/CDK4/NFKBIA/ESR1	4
BP	GO:0033077	T cell differentiation in thymus	3/132	87/18870	0.02315917	0.04599184	0.02102416	BCL2/TP53/CDK6	3
BP	GO:0002440	production of molecular mediator of immune response	6/132	314/18870	0.02318471	0.04602019	0.02103712	TNF/IL4R/SYK/ARG1/AXL/IL2	6
BP	GO:0001782	B cell homeostasis	2/132	34/18870	0.0235421	0.04641384	0.02121707	BCL2/BAX	2
BP	GO:0002828	regulation of type 2 immune response	2/132	34/18870	0.0235421	0.04641384	0.02121707	IL4R/ARG1	2
BP	GO:0007431	salivary gland development	2/132	34/18870	0.0235421	0.04641384	0.02121707	TNF/EGFR	2
BP	GO:0034110	regulation of homotypic cell-cell adhesion	2/132	34/18870	0.0235421	0.04641384	0.02121707	SYK/ALOX12	2
BP	GO:0035025	positive regulation of Rho protein signal transduction	2/132	34/18870	0.0235421	0.04641384	0.02121707	COL3A1/GPR35	2
BP	GO:0045879	negative regulation of smoothened signaling pathway	2/132	34/18870	0.0235421	0.04641384	0.02121707	RB1/RUNX2	2
BP	GO:0046825	regulation of protein export from nucleus	2/132	34/18870	0.0235421	0.04641384	0.02121707	GSK3B/PTPN11	2
BP	GO:0048333	mesodermal cell differentiation	2/132	34/18870	0.0235421	0.04641384	0.02121707	ITGB3/GJA1	2
BP	GO:0050901	leukocyte tethering or rolling	2/132	34/18870	0.0235421	0.04641384	0.02121707	TNF/SELE	2
BP	GO:0051450	myoblast proliferation	2/132	34/18870	0.0235421	0.04641384	0.02121707	FOS/SRC	2
BP	GO:0051973	positive regulation of telomerase activity	2/132	34/18870	0.0235421	0.04641384	0.02121707	HSP90AA1/MYC	2
BP	GO:0060351	cartilage development involved in endochondral bone morphogenesis	2/132	34/18870	0.0235421	0.04641384	0.02121707	COL1A1/MMP13	2
BP	GO:0071353	cellular response to interleukin-4	2/132	34/18870	0.0235421	0.04641384	0.02121707	CDK4/IL4R	2
BP	GO:0086019	cell-cell signaling involved in cardiac conduction	2/132	34/18870	0.0235421	0.04641384	0.02121707	SCN5A/GJA1	2
BP	GO:0032147	activation of protein kinase activity	3/132	88/18870	0.02385691	0.04694388	0.02145937	IL6R/ITGB3/INSR	3
BP	GO:0046330	positive regulation of JNK cascade	3/132	88/18870	0.02385691	0.04694388	0.02145937	TNF/APP/XIAP	3
BP	GO:0048864	stem cell development	3/132	88/18870	0.02385691	0.04694388	0.02145937	BCL2/EDN1/EDNRA	3
BP	GO:0050672	negative regulation of lymphocyte proliferation	3/132	88/18870	0.02385691	0.04694388	0.02145937	IL2RA/ARG1/IL2	3
BP	GO:1905039	carboxylic acid transmembrane transport	4/132	156/18870	0.02404553	0.04723925	0.02159439	TNF/ABCC1/AKT1/ABCB1	4
BP	GO:0007411	axon guidance	5/132	233/18870	0.02405328	0.04723925	0.02159439	KLF7/APP/EDN1/EDNRA/PTK2	5
BP	GO:0016358	dendrite development	5/132	233/18870	0.02405328	0.04723925	0.02159439	KLF7/APP/GSK3B/ALK/IL2	5
BP	GO:0097485	neuron projection guidance	5/132	233/18870	0.02405328	0.04723925	0.02159439	KLF7/APP/EDN1/EDNRA/PTK2	5
BP	GO:0007043	cell-cell junction assembly	4/132	157/18870	0.02454448	0.04801532	0.02194915	TNF/CAV1/GJA1/CLDN4	4
BP	GO:1903825	organic acid transmembrane transport	4/132	157/18870	0.02454448	0.04801532	0.02194915	TNF/ABCC1/AKT1/ABCB1	4
BP	GO:0006096	glycolytic process	3/132	89/18870	0.02456598	0.04801532	0.02194915	APP/STAT3/INSR	3
BP	GO:0006112	energy reserve metabolic process	3/132	89/18870	0.02456598	0.04801532	0.02194915	GSK3B/AKT1/INSR	3
BP	GO:0021954	central nervous system neuron development	3/132	89/18870	0.02456598	0.04801532	0.02194915	HSP90AA1/DRD2/MAPT	3
BP	GO:0032945	negative regulation of mononuclear cell proliferation	3/132	89/18870	0.02456598	0.04801532	0.02194915	IL2RA/ARG1/IL2	3
BP	GO:0034340	response to type I interferon	3/132	89/18870	0.02456598	0.04801532	0.02194915	STAT1/MMP12/PTPN11	3
BP	GO:0034620	cellular response to unfolded protein	3/132	89/18870	0.02456598	0.04801532	0.02194915	BAX/CCND1/PIK3R1	3
BP	GO:0051453	regulation of intracellular pH	3/132	89/18870	0.02456598	0.04801532	0.02194915	BCL2/CA2/CFTR	3
BP	GO:0055017	cardiac muscle tissue growth	3/132	89/18870	0.02456598	0.04801532	0.02194915	CDK1/EDN1/PIM1	3
BP	GO:0140694	non-membrane-bounded organelle assembly	7/132	408/18870	0.02474546	0.0482364	0.02205021	CDK2/TNF/BIRC5/EDN1/SRC/PLK1/MAPT	7
BP	GO:0000132	establishment of mitotic spindle orientation	2/132	35/18870	0.02485621	0.0482364	0.02205021	GJA1/PLK1	2
BP	GO:0010613	positive regulation of cardiac muscle hypertrophy	2/132	35/18870	0.02485621	0.0482364	0.02205021	EDN1/PARP1	2
BP	GO:0030224	monocyte differentiation	2/132	35/18870	0.02485621	0.0482364	0.02205021	MYC/CDK6	2
BP	GO:0032205	negative regulation of telomere maintenance	2/132	35/18870	0.02485621	0.0482364	0.02205021	PARP1/SRC	2
BP	GO:0034367	protein-containing complex remodeling	2/132	35/18870	0.02485621	0.0482364	0.02205021	MTTP/MPO	2
BP	GO:0035305	negative regulation of dephosphorylation	2/132	35/18870	0.02485621	0.0482364	0.02205021	TNF/GSK3B	2
BP	GO:0051204	protein insertion into mitochondrial membrane	2/132	35/18870	0.02485621	0.0482364	0.02205021	HSP90AA1/BAX	2
BP	GO:0060544	regulation of necroptotic process	2/132	35/18870	0.02485621	0.0482364	0.02205021	CAV1/PARP1	2
BP	GO:0090050	positive regulation of cell migration involved in sprouting angiogenesis	2/132	35/18870	0.02485621	0.0482364	0.02205021	KDR/PTGS2	2
BP	GO:0098810	neurotransmitter reuptake	2/132	35/18870	0.02485621	0.0482364	0.02205021	DRD2/ITGB3	2
BP	GO:0110110	positive regulation of animal organ morphogenesis	2/132	35/18870	0.02485621	0.0482364	0.02205021	AR/EDN1	2

BP	GO:1901385	regulation of voltage-gated calcium channel activity	2/132	35/18870	0.02485621	0.0482364	0.02205021	DRD2/GPR35	2
BP	GO:1902882	regulation of response to oxidative stress	2/132	35/18870	0.02485621	0.0482364	0.02205021	ALOX5/DHFR	2
BP	GO:2000515	negative regulation of CD4-positive, alpha-beta T cell activation	2/132	35/18870	0.02485621	0.0482364	0.02205021	IL4R/IL2	2
BP	GO:0007189	adenylate cyclase-activating G protein-coupled receptor signaling pathwa	4/132	158/18870	0.02504967	0.04858874	0.02221128	DRD2/CXCL11/CXCL10/ADORA2A	4
BP	GO:1903321	negative regulation of protein modification by small protein conjugation o	3/132	90/18870	0.02528636	0.04902458	0.02241051	RELA/CAV1/AKT1	3
BP	GO:0035966	response to topologically incorrect protein	4/132	159/18870	0.02556111	0.0494868	0.02262181	HSP90AA1/BAX/CCND1/PIK3R1	4
BP	GO:0060041	retina development in camera-type eye	4/132	159/18870	0.02556111	0.0494868	0.02262181	BAX/STAT3/CYP1B1/ACHE	4
BP	GO:1901875	positive regulation of post-translational protein modification	4/132	159/18870	0.02556111	0.0494868	0.02262181	XIAP/CAV1/GSK3B/PLK1	4
BP	GO:0031507	heterochromatin formation	3/132	91/18870	0.02601805	0.05029993	0.02299351	CDK2/MYC/RB1	3
BP	GO:0097006	regulation of plasma lipoprotein particle levels	3/132	91/18870	0.02601805	0.05029993	0.02299351	MTTP/ADIPOQ/MPO	3
BP	GO:0097581	lamellipodium organization	3/132	91/18870	0.02601805	0.05029993	0.02299351	HSP90AA1/PIK3R1/SRC	3
BP	GO:0014742	positive regulation of muscle hypertrophy	2/132	36/18870	0.02619963	0.05048373	0.02307753	EDN1/PARP1	2
BP	GO:0031112	positive regulation of microtubule polymerization or depolymerization	2/132	36/18870	0.02619963	0.05048373	0.02307753	MET/MAPT	2
BP	GO:0032094	response to food	2/132	36/18870	0.02619963	0.05048373	0.02307753	MPO/AKT1	2
BP	GO:0032232	negative regulation of actin filament bundle assembly	2/132	36/18870	0.02619963	0.05048373	0.02307753	PIK3R1/MET	2
BP	GO:0070670	response to interleukin-4	2/132	36/18870	0.02619963	0.05048373	0.02307753	CDK4/IL4R	2
BP	GO:0071711	basement membrane organization	2/132	36/18870	0.02619963	0.05048373	0.02307753	CAV1/COL3A1	2
BP	GO:1905332	positive regulation of morphogenesis of an epithelium	2/132	36/18870	0.02619963	0.05048373	0.02307753	AR/GJA1	2
BP	GO:0070507	regulation of microtubule cytoskeleton organization	4/132	161/18870	0.02660283	0.05123647	0.02342163	GSK3B/PLK1/MET/MAPT	4
BP	GO:0031640	killing of cells of another organism	3/132	92/18870	0.02676105	0.05149265	0.02353874	SYK/ARG1/F2	3
BP	GO:0141061	disruption of cell in another organism	3/132	92/18870	0.02676105	0.05149265	0.02353874	SYK/ARG1/F2	3
BP	GO:0048592	eye morphogenesis	4/132	162/18870	0.02713312	0.05218399	0.02385477	BCL2/BAX/STAT3/KDR	4
BP	GO:0006813	potassium ion transport	5/132	241/18870	0.02730291	0.05248584	0.02399275	KCNH2/CDK2/DRD2/CAV1/ADORA1	5
BP	GO:0030641	regulation of cellular pH	3/132	93/18870	0.02751534	0.0527053	0.02409308	BCL2/CA2/CFTR	3
BP	GO:0039531	regulation of viral-induced cytoplasmic pattern recognition receptor signa	3/132	93/18870	0.02751534	0.0527053	0.02409308	XIAP/CAV1/SRC	3
BP	GO:0043502	regulation of muscle adaptation	3/132	93/18870	0.02751534	0.0527053	0.02409308	SCN5A/EDN1/PARP1	3
BP	GO:0045185	maintenance of protein location	3/132	93/18870	0.02751534	0.0527053	0.02409308	NFKBIA/CAV1/AKT1	3
BP	GO:0003161	cardiac conduction system development	2/132	37/18870	0.02757191	0.0527053	0.02409308	SCN5A/GJA1	2
BP	GO:0008207	C21-steroid hormone metabolic process	2/132	37/18870	0.02757191	0.0527053	0.02409308	AKR1B1/AKR1C3	2
BP	GO:0042755	eating behavior	2/132	37/18870	0.02757191	0.0527053	0.02409308	STAT3/ADORA2A	2
BP	GO:0043267	negative regulation of potassium ion transport	2/132	37/18870	0.02757191	0.0527053	0.02409308	KCNH2/CAV1	2
BP	GO:0046677	response to antibiotic	2/132	37/18870	0.02757191	0.0527053	0.02409308	HSP90AA1/TP53	2
BP	GO:0048261	negative regulation of receptor-mediated endocytosis	2/132	37/18870	0.02757191	0.0527053	0.02409308	ADIPOQ/ITGB3	2
BP	GO:0097345	mitochondrial outer membrane permeabilization	2/132	37/18870	0.02757191	0.0527053	0.02409308	BAX/GSK3B	2
BP	GO:1905144	response to acetylcholine	2/132	37/18870	0.02757191	0.0527053	0.02409308	EDNRA/ACHE	2
BP	GO:0009791	post-embryonic development	3/132	94/18870	0.02828092	0.05401007	0.02468952	BCL2/BAX/KDR	3
BP	GO:0051781	positive regulation of cell division	3/132	94/18870	0.02828092	0.05401007	0.02468952	DRD2/BIRC5/IGF1R	3
BP	GO:0007143	female meiotic nuclear division	2/132	38/18870	0.02897256	0.05517618	0.02522258	PLK1/TOP2A	2
BP	GO:0007616	long-term memory	2/132	38/18870	0.02897256	0.05517618	0.02522258	DRD2/SLC2A4	2
BP	GO:0035633	maintenance of blood-brain barrier	2/132	38/18870	0.02897256	0.05517618	0.02522258	GJA1/PTGS2	2
BP	GO:0042044	fluid transport	2/132	38/18870	0.02897256	0.05517618	0.02522258	EDN1/CFTR	2
BP	GO:0071404	cellular response to low-density lipoprotein particle stimulus	2/132	38/18870	0.02897256	0.05517618	0.02522258	SYK/AKT1	2
BP	GO:0090151	establishment of protein localization to mitochondrial membrane	2/132	38/18870	0.02897256	0.05517618	0.02522258	HSP90AA1/BAX	2
BP	GO:0016485	protein processing	5/132	245/18870	0.02902776	0.0552298	0.0252471	BAD/XIAP/PARP1/SRC/F3	5
BP	GO:0032984	protein-containing complex disassembly	5/132	245/18870	0.02902776	0.0552298	0.0252471	TNF/MYC/GSK3B/IGF1R/INSR	5
BP	GO:0007519	skeletal muscle tissue development	4/132	166/18870	0.0293175	0.05575512	0.02548724	BCL2/FOS/RB1/CAV1	4
BP	GO:0002042	cell migration involved in sprouting angiogenesis	3/132	96/18870	0.02984585	0.05665442	0.02589833	KDR/AKT1/PTGS2	3
BP	GO:0042982	amyloid precursor protein metabolic process	3/132	96/18870	0.02984585	0.05665442	0.02589833	RELA/TNF/ACHE	3
BP	GO:0051310	metaphase chromosome alignment	3/132	96/18870	0.02984585	0.05665442	0.02589833	CCNB1/RB1/BIRC5	3

BP	GO:0070664	negative regulation of leukocyte proliferation	3/132	96/18870	0.02984585	0.05665442	0.02589833	IL2RA/ARG1/IL2	3
BP	GO:0006814	sodium ion transport	5/132	248/18870	0.03036575	0.05744156	0.02625815	SCN5A/DRD2/EDN1/EDNRA/AKT1	5
BP	GO:0032350	regulation of hormone metabolic process	2/132	39/18870	0.03040114	0.05744156	0.02625815	IGF1R/AKR1C3	2
BP	GO:0038179	neurotrophin signaling pathway	2/132	39/18870	0.03040114	0.05744156	0.02625815	SRC/PTPN11	2
BP	GO:0043620	regulation of DNA-templated transcription in response to stress	2/132	39/18870	0.03040114	0.05744156	0.02625815	RELA/TP53	2
BP	GO:0045746	negative regulation of Notch signaling pathway	2/132	39/18870	0.03040114	0.05744156	0.02625815	NFKBIA/AKT1	2
BP	GO:0045823	positive regulation of heart contraction	2/132	39/18870	0.03040114	0.05744156	0.02625815	HSP90AA1/EDN1	2
BP	GO:0055094	response to lipoprotein particle	2/132	39/18870	0.03040114	0.05744156	0.02625815	SYK/AKT1	2
BP	GO:0070229	negative regulation of lymphocyte apoptotic process	2/132	39/18870	0.03040114	0.05744156	0.02625815	BCL2/IL2	2
BP	GO:0098926	postsynaptic signal transduction	2/132	39/18870	0.03040114	0.05744156	0.02625815	RELA/ACHE	2
BP	GO:0099622	cardiac muscle cell membrane repolarization	2/132	39/18870	0.03040114	0.05744156	0.02625815	KCNH2/SCN5A	2
BP	GO:0003205	cardiac chamber development	4/132	168/18870	0.03044777	0.05750308	0.02628628	SCN5A/TP53/EDNRA/IGF1R	4
BP	GO:0050688	regulation of defense response to virus	3/132	97/18870	0.03064519	0.05779575	0.02642006	HSP90AA1/STAT1/MMP12	3
BP	GO:0060419	heart growth	3/132	97/18870	0.03064519	0.05779575	0.02642006	CDK1/EDN1/PIM1	3
BP	GO:1901874	negative regulation of post-translational protein modification	3/132	97/18870	0.03064519	0.05779575	0.02642006	RELA/CAV1/AKT1	3
BP	GO:0015698	inorganic anion transport	4/132	169/18870	0.03102246	0.05848028	0.02673298	CLDN4/CA2/ABCB1/CFTR	4
BP	GO:0048534	hematopoietic or lymphoid organ development	3/132	98/18870	0.03145574	0.05924237	0.02708135	BCL2/TP53/TYR	3
BP	GO:0060993	kidney morphogenesis	3/132	98/18870	0.03145574	0.05924237	0.02708135	BCL2/MYC/STAT1	3
BP	GO:0000266	mitochondrial fission	2/132	40/18870	0.03185719	0.05975051	0.02731364	KDR/MAPT	2
BP	GO:0032728	positive regulation of interferon-beta production	2/132	40/18870	0.03185719	0.05975051	0.02731364	HSP90AA1/PTPN11	2
BP	GO:0033028	myeloid cell apoptotic process	2/132	40/18870	0.03185719	0.05975051	0.02731364	BCL2/ADIPOQ	2
BP	GO:0040001	establishment of mitotic spindle localization	2/132	40/18870	0.03185719	0.05975051	0.02731364	GJA1/PLK1	2
BP	GO:0070232	regulation of T cell apoptotic process	2/132	40/18870	0.03185719	0.05975051	0.02731364	BCL2/TP53	2
BP	GO:0070884	regulation of calcineurin-NFAT signaling cascade	2/132	40/18870	0.03185719	0.05975051	0.02731364	TNF/GSK3B	2
BP	GO:0071459	protein localization to chromosome, centromeric region	2/132	40/18870	0.03185719	0.05975051	0.02731364	CDK1/RB1	2
BP	GO:1901532	regulation of hematopoietic progenitor cell differentiation	2/132	40/18870	0.03185719	0.05975051	0.02731364	KDR/CDK6	2
BP	GO:1903524	positive regulation of blood circulation	2/132	40/18870	0.03185719	0.05975051	0.02731364	HSP90AA1/EDN1	2
BP	GO:0051588	regulation of neurotransmitter transport	3/132	99/18870	0.0322775	0.06051105	0.0276613	DRD2/ITGB3/ADORA2A	3
BP	GO:0042632	cholesterol homeostasis	3/132	100/18870	0.03311043	0.06198721	0.0283361	MTTP/CAV1/NR1H3	3
BP	GO:0070828	heterochromatin organization	3/132	100/18870	0.03311043	0.06198721	0.0283361	CDK2/MYC/RB1	3
BP	GO:0072080	nephron tubule development	3/132	100/18870	0.03311043	0.06198721	0.0283361	BCL2/MYC/STAT1	3
BP	GO:0010591	regulation of lamellipodium assembly	2/132	41/18870	0.03334026	0.06224633	0.02845455	HSP90AA1/PIK3R1	2
BP	GO:0031952	regulation of protein autophosphorylation	2/132	41/18870	0.03334026	0.06224633	0.02845455	ADIPOQ/CAV1	2
BP	GO:0043388	positive regulation of DNA binding	2/132	41/18870	0.03334026	0.06224633	0.02845455	RB1/MMP9	2
BP	GO:0051954	positive regulation of amine transport	2/132	41/18870	0.03334026	0.06224633	0.02845455	DRD2/ADORA2A	2
BP	GO:0072210	metanephric nephron development	2/132	41/18870	0.03334026	0.06224633	0.02845455	ADIPOQ/STAT1	2
BP	GO:0106056	regulation of calcineurin-mediated signaling	2/132	41/18870	0.03334026	0.06224633	0.02845455	TNF/GSK3B	2
BP	GO:0055092	sterol homeostasis	3/132	101/18870	0.03395452	0.06336419	0.02896555	MTTP/CAV1/NR1H3	3
BP	GO:0055088	lipid homeostasis	4/132	175/18870	0.03460488	0.06453396	0.02950029	MTTP/CAV1/ADORA1/NR1H3	4
BP	GO:0005976	polysaccharide metabolic process	3/132	102/18870	0.03480974	0.06453396	0.02950029	GSK3B/AKT1/INSR	3
BP	GO:0042773	ATP synthesis coupled electron transport	3/132	102/18870	0.03480974	0.06453396	0.02950029	CDK1/CCNB1/CYCS	3
BP	GO:0042775	mitochondrial ATP synthesis coupled electron transport	3/132	102/18870	0.03480974	0.06453396	0.02950029	CDK1/CCNB1/CYCS	3
BP	GO:0046364	monosaccharide biosynthetic process	3/132	102/18870	0.03480974	0.06453396	0.02950029	ADIPOQ/AKR1B1/AKR1A1	3
BP	GO:0070167	regulation of biomineral tissue development	3/132	102/18870	0.03480974	0.06453396	0.02950029	RXRA/ALOX5/CFTR	3
BP	GO:0009394	2'-deoxyribonucleotide metabolic process	2/132	42/18870	0.03484992	0.06453396	0.02950029	XDH/TYMS	2
BP	GO:0032733	positive regulation of interleukin-10 production	2/132	42/18870	0.03484992	0.06453396	0.02950029	STAT3/SYK	2
BP	GO:0032941	secretion by tissue	2/132	42/18870	0.03484992	0.06453396	0.02950029	ADORA1/NR1H3	2
BP	GO:0042092	type 2 immune response	2/132	42/18870	0.03484992	0.06453396	0.02950029	IL4R/ARG1	2
BP	GO:0042119	neutrophil activation	2/132	42/18870	0.03484992	0.06453396	0.02950029	TNF/SYK	2

BP	GO:0050850	positive regulation of calcium-mediated signaling	2/132	42/18870	0.03484992	0.06453396	0.02950029	TNF/SYK	2
BP	GO:0051294	establishment of spindle orientation	2/132	42/18870	0.03484992	0.06453396	0.02950029	GJA1/PLK1	2
BP	GO:0071402	cellular response to lipoprotein particle stimulus	2/132	42/18870	0.03484992	0.06453396	0.02950029	SYK/AKT1	2
BP	GO:1904706	negative regulation of vascular associated smooth muscle cell proliferatior	2/132	42/18870	0.03484992	0.06453396	0.02950029	ADIPOQ/CDKN1A	2
BP	GO:1990000	amyloid fibril formation	2/132	42/18870	0.03484992	0.06453396	0.02950029	APP/MAPT	2
BP	GO:2000008	regulation of protein localization to cell surface	2/132	42/18870	0.03484992	0.06453396	0.02950029	TNF/AKT1	2
BP	GO:0043409	negative regulation of MAPK cascade	4/132	176/18870	0.03522439	0.06519784	0.02980377	ADIPOQ/MYC/CAV1/IGF1R	4
BP	GO:0015914	phospholipid transport	3/132	103/18870	0.03567608	0.06600397	0.03017227	MTTP/ABCC1/ABCB1	3
BP	GO:0002251	organ or tissue specific immune response	2/132	43/18870	0.03638572	0.066953	0.0306061	IL6R/PLA2G1B	2
BP	GO:0002714	positive regulation of B cell mediated immunity	2/132	43/18870	0.03638572	0.066953	0.0306061	TNF/IL2	2
BP	GO:0002891	positive regulation of immunoglobulin mediated immune response	2/132	43/18870	0.03638572	0.066953	0.0306061	TNF/IL2	2
BP	GO:0009262	deoxyribonucleotide metabolic process	2/132	43/18870	0.03638572	0.066953	0.0306061	XDH/TYMS	2
BP	GO:0019692	deoxyribose phosphate metabolic process	2/132	43/18870	0.03638572	0.066953	0.0306061	XDH/TYMS	2
BP	GO:0032735	positive regulation of interleukin-12 production	2/132	43/18870	0.03638572	0.066953	0.0306061	RELA/SYK	2
BP	GO:0033003	regulation of mast cell activation	2/132	43/18870	0.03638572	0.066953	0.0306061	IL4R/SYK	2
BP	GO:0045622	regulation of T-helper cell differentiation	2/132	43/18870	0.03638572	0.066953	0.0306061	IL4R/IL2	2
BP	GO:0046717	acid secretion	2/132	43/18870	0.03638572	0.066953	0.0306061	TNF/DRD2	2
BP	GO:0055090	acylglycerol homeostasis	2/132	43/18870	0.03638572	0.066953	0.0306061	ADORA1/NR1H3	2
BP	GO:0070328	triglyceride homeostasis	2/132	43/18870	0.03638572	0.066953	0.0306061	ADORA1/NR1H3	2
BP	GO:1902110	positive regulation of mitochondrial membrane permeability involved in ap	2/132	43/18870	0.03638572	0.066953	0.0306061	BAX/GSK3B	2
BP	GO:0060538	skeletal muscle organ development	4/132	178/18870	0.0364827	0.06710123	0.03067386	BCL2/FOS/RB1/CAV1	4
BP	GO:0019218	regulation of steroid metabolic process	3/132	104/18870	0.03655349	0.06714074	0.03069192	TNF/IGF1R/AKR1C3	3
BP	GO:0032204	regulation of telomere maintenance	3/132	104/18870	0.03655349	0.06714074	0.03069192	MYC/PARP1/SRC	3
BP	GO:0061326	renal tubule development	3/132	104/18870	0.03655349	0.06714074	0.03069192	BCL2/MYC/STAT1	3
BP	GO:0042102	positive regulation of T cell proliferation	3/132	105/18870	0.03744196	0.06871088	0.03140967	IL2RA/SYK/IL2	3
BP	GO:0110020	regulation of actomyosin structure organization	3/132	105/18870	0.03744196	0.06871088	0.03140967	EDN1/PIK3R1/MET	3
BP	GO:0006284	base-excision repair	2/132	44/18870	0.03794723	0.06923378	0.03164871	PARP1/APEX1	2
BP	GO:0006509	membrane protein ectodomain proteolysis	2/132	44/18870	0.03794723	0.06923378	0.03164871	TIMP1/TNF	2
BP	GO:0009409	response to cold	2/132	44/18870	0.03794723	0.06923378	0.03164871	HSP90AA1/NFKBIA	2
BP	GO:0019320	hexose catabolic process	2/132	44/18870	0.03794723	0.06923378	0.03164871	TP53/BAD	2
BP	GO:0030501	positive regulation of bone mineralization	2/132	44/18870	0.03794723	0.06923378	0.03164871	RXRA/ALOX5	2
BP	GO:0043268	positive regulation of potassium ion transport	2/132	44/18870	0.03794723	0.06923378	0.03164871	KCNH2/ADORA1	2
BP	GO:0043506	regulation of JUN kinase activity	2/132	44/18870	0.03794723	0.06923378	0.03164871	TNF/EDN1	2
BP	GO:0045761	regulation of adenylate cyclase activity	2/132	44/18870	0.03794723	0.06923378	0.03164871	DRD2/EDNRA	2
BP	GO:0060338	regulation of type I interferon-mediated signaling pathway	2/132	44/18870	0.03794723	0.06923378	0.03164871	MMP12/PTPN11	2
BP	GO:0090207	regulation of triglyceride metabolic process	2/132	44/18870	0.03794723	0.06923378	0.03164871	NR1H3/PIK3CG	2
BP	GO:0120178	steroid hormone biosynthetic process	2/132	44/18870	0.03794723	0.06923378	0.03164871	CYP19A1/AKR1B1	2
BP	GO:1903307	positive regulation of regulated secretory pathway	2/132	44/18870	0.03794723	0.06923378	0.03164871	IL4R/SYK	2
BP	GO:1903573	negative regulation of response to endoplasmic reticulum stress	2/132	44/18870	0.03794723	0.06923378	0.03164871	ALOX5/NR1H3	2
BP	GO:0042129	regulation of T cell proliferation	4/132	182/18870	0.03907658	0.07126242	0.03257606	IL2RA/SYK/ARG1/IL2	4
BP	GO:0001504	neurotransmitter uptake	2/132	45/18870	0.03953402	0.07180812	0.03282551	DRD2/ITGB3	2
BP	GO:0002347	response to tumor cell	2/132	45/18870	0.03953402	0.07180812	0.03282551	RELA/AHR	2
BP	GO:0010799	regulation of peptidyl-threonine phosphorylation	2/132	45/18870	0.03953402	0.07180812	0.03282551	APP/PLK1	2
BP	GO:0031641	regulation of myelination	2/132	45/18870	0.03953402	0.07180812	0.03282551	TNF/AKT1	2
BP	GO:0033173	calcineurin-NFAT signaling cascade	2/132	45/18870	0.03953402	0.07180812	0.03282551	TNF/GSK3B	2
BP	GO:0051602	response to electrical stimulus	2/132	45/18870	0.03953402	0.07180812	0.03282551	MMP2/SRC	2
BP	GO:0071604	transforming growth factor beta production	2/132	45/18870	0.03953402	0.07180812	0.03282551	COL3A1/PTGS2	2
BP	GO:1902686	mitochondrial outer membrane permeabilization involved in programmed	2/132	45/18870	0.03953402	0.07180812	0.03282551	BAX/GSK3B	2
BP	GO:1905521	regulation of macrophage migration	2/132	45/18870	0.03953402	0.07180812	0.03282551	CYP19A1/PTK2	2

BP	GO:0090287	regulation of cellular response to growth factor stimulus	6/132	357/18870	0.03967895	0.07203934	0.03293121	TP53/XIAP/ITGB3/RUNX2/XDH/KDR	6
BP	GO:0051303	establishment of chromosome localization	3/132	108/18870	0.0401734	0.07290464	0.03332676	CCNB1/RB1/BIRC5	3
BP	GO:0051495	positive regulation of cytoskeleton organization	4/132	184/18870	0.04041219	0.07330542	0.03350997	EDN1/ALOX15/MET/MAPT	4
BP	GO:0035967	cellular response to topologically incorrect protein	3/132	109/18870	0.04110578	0.07440462	0.03401245	BAX/CCND1/PIK3R1	3
BP	GO:0061387	regulation of extent of cell growth	3/132	109/18870	0.04110578	0.07440462	0.03401245	SPP1/GSK3B/MAPT	3
BP	GO:0003009	skeletal muscle contraction	2/132	46/18870	0.04114567	0.07440462	0.03401245	HSP90AA1/TNF	2
BP	GO:0006984	ER-nucleus signaling pathway	2/132	46/18870	0.04114567	0.07440462	0.03401245	TP53/GSK3B	2
BP	GO:0032892	positive regulation of organic acid transport	2/132	46/18870	0.04114567	0.07440462	0.03401245	EDN1/ADORA2A	2
BP	GO:0097028	dendritic cell differentiation	2/132	46/18870	0.04114567	0.07440462	0.03401245	FLT3/AXL	2
BP	GO:1901976	regulation of cell cycle checkpoint	2/132	46/18870	0.04114567	0.07440462	0.03401245	CCNB1/BIRC5	2
BP	GO:0002699	positive regulation of immune effector process	5/132	270/18870	0.04136453	0.0747673	0.03417823	TNF/IL4R/SYK/ARG1/IL2	5
BP	GO:0030038	contractile actin filament bundle assembly	3/132	110/18870	0.04204907	0.07587033	0.03468246	PIK3R1/SRC/MET	3
BP	GO:0043149	stress fiber assembly	3/132	110/18870	0.04204907	0.07587033	0.03468246	PIK3R1/SRC/MET	3
BP	GO:0045445	myoblast differentiation	3/132	110/18870	0.04204907	0.07587033	0.03468246	TNF/RB1/CXCL10	3
BP	GO:0120034	positive regulation of plasma membrane bounded cell projection assembly	3/132	110/18870	0.04204907	0.07587033	0.03468246	HSP90AA1/GSK3B/PIK3R1	3
BP	GO:0001881	receptor recycling	2/132	47/18870	0.04278175	0.0769883	0.03519352	ACHE/CTSD	2
BP	GO:0035196	miRNA processing	2/132	47/18870	0.04278175	0.0769883	0.03519352	TP53/STAT3	2
BP	GO:0043300	regulation of leukocyte degranulation	2/132	47/18870	0.04278175	0.0769883	0.03519352	IL4R/SYK	2
BP	GO:0045601	regulation of endothelial cell differentiation	2/132	47/18870	0.04278175	0.0769883	0.03519352	TNF/XDH	2
BP	GO:0060043	regulation of cardiac muscle cell proliferation	2/132	47/18870	0.04278175	0.0769883	0.03519352	CDK1/PIM1	2
BP	GO:0061383	trabecula morphogenesis	2/132	47/18870	0.04278175	0.0769883	0.03519352	COL1A1/MMP2	2
BP	GO:0060840	artery development	3/132	111/18870	0.04300321	0.07735276	0.03536012	EDN1/EDNRA/COL3A1	3
BP	GO:0000910	cytokinesis	4/132	188/18870	0.0431608	0.07753381	0.03544289	DRD2/BIRC5/IGF1R/PLK1	4
BP	GO:0002706	regulation of lymphocyte mediated immunity	4/132	188/18870	0.0431608	0.07753381	0.03544289	TNF/ARG1/IL2/AHR	4
BP	GO:0030324	lung development	4/132	188/18870	0.0431608	0.07753381	0.03544289	TNF/COL3A1/MMP12/KDR	4
BP	GO:0021795	cerebral cortex cell migration	2/132	48/18870	0.04444186	0.07952036	0.03635099	COL3A1/EGFR	2
BP	GO:0032964	collagen biosynthetic process	2/132	48/18870	0.04444186	0.07952036	0.03635099	COL1A1/F2	2
BP	GO:0042181	ketone biosynthetic process	2/132	48/18870	0.04444186	0.07952036	0.03635099	CYP19A1/AKR1C3	2
BP	GO:0045747	positive regulation of Notch signaling pathway	2/132	48/18870	0.04444186	0.07952036	0.03635099	STAT3/SRC	2
BP	GO:0097720	calcineurin-mediated signaling	2/132	48/18870	0.04444186	0.07952036	0.03635099	TNF/GSK3B	2
BP	GO:0099601	regulation of neurotransmitter receptor activity	2/132	48/18870	0.04444186	0.07952036	0.03635099	APP/CCL2	2
BP	GO:0140374	antiviral innate immune response	2/132	48/18870	0.04444186	0.07952036	0.03635099	TNF/CXCL10	2
BP	GO:1900542	regulation of purine nucleotide metabolic process	2/132	48/18870	0.04444186	0.07952036	0.03635099	TP53/PARP1	2
BP	GO:1902003	regulation of amyloid-beta formation	2/132	48/18870	0.04444186	0.07952036	0.03635099	RELA/TNF	2
BP	GO:0002040	sprouting angiogenesis	4/132	190/18870	0.0445738	0.07972154	0.03644296	ALOX5/KDR/AKT1/PTGS2	4
BP	GO:0032535	regulation of cellular component size	6/132	370/18870	0.04583669	0.08194436	0.03745907	HSP90AA1/EDN1/SPP1/GSK3B/ALOX15/MAPT	6
BP	GO:0030323	respiratory tube development	4/132	192/18870	0.04601258	0.08199433	0.03748191	TNF/COL3A1/MMP12/KDR	4
BP	GO:0006140	regulation of nucleotide metabolic process	2/132	49/18870	0.04612557	0.08199433	0.03748191	TP53/PARP1	2
BP	GO:0007520	myoblast fusion	2/132	49/18870	0.04612557	0.08199433	0.03748191	IL4R/CXCL10	2
BP	GO:0031648	protein destabilization	2/132	49/18870	0.04612557	0.08199433	0.03748191	SRC/PLK1	2
BP	GO:0035987	endodermal cell differentiation	2/132	49/18870	0.04612557	0.08199433	0.03748191	MMP9/MMP2	2
BP	GO:0036230	granulocyte activation	2/132	49/18870	0.04612557	0.08199433	0.03748191	TNF/SYK	2
BP	GO:0042220	response to cocaine	2/132	49/18870	0.04612557	0.08199433	0.03748191	HSP90AA1/DRD2	2
BP	GO:0043330	response to exogenous dsRNA	2/132	49/18870	0.04612557	0.08199433	0.03748191	NFKBIA/CAV1	2
BP	GO:0048286	lung alveolus development	2/132	49/18870	0.04612557	0.08199433	0.03748191	MMP12/KDR	2
BP	GO:0048538	thymus development	2/132	49/18870	0.04612557	0.08199433	0.03748191	BCL2/TYR	2
BP	GO:0048546	digestive tract morphogenesis	2/132	49/18870	0.04612557	0.08199433	0.03748191	BCL2/EGFR	2
BP	GO:0061028	establishment of endothelial barrier	2/132	49/18870	0.04612557	0.08199433	0.03748191	TNF/EDNRA	2
BP	GO:2001259	positive regulation of cation channel activity	2/132	49/18870	0.04612557	0.08199433	0.03748191	EDN1/EDNRA	2

BP	GO:0006305	DNA alkylation	2/132	50/18870	0.04783249	0.08480718	0.03876775	FOS/PARP1	2
BP	GO:0006306	DNA methylation	2/132	50/18870	0.04783249	0.08480718	0.03876775	FOS/PARP1	2
BP	GO:0043114	regulation of vascular permeability	2/132	50/18870	0.04783249	0.08480718	0.03876775	ADORA2A/SRC	2
BP	GO:0061082	myeloid leukocyte cytokine production	2/132	50/18870	0.04783249	0.08480718	0.03876775	SYK/AXL	2
BP	GO:0071827	plasma lipoprotein particle organization	2/132	50/18870	0.04783249	0.08480718	0.03876775	MTTP/MPO	2
BP	GO:1904752	regulation of vascular associated smooth muscle cell migration	2/132	50/18870	0.04783249	0.08480718	0.03876775	ADIPOQ/TERT	2
BP	GO:0050000	chromosome localization	3/132	116/18870	0.04793554	0.08495301	0.03883441	CCNB1/RB1/BIRC5	3
BP	GO:0055001	muscle cell development	4/132	195/18870	0.04821903	0.08541836	0.03904714	BCL2/CDK1/EDN1/EDNRA	4
BP	GO:0099173	postsynapse organization	4/132	196/18870	0.04896738	0.08670643	0.03963595	ITGB3/IGF1R/AKT1/INSR	4
BP	GO:0045581	negative regulation of T cell differentiation	2/132	51/18870	0.04956222	0.08772169	0.04010005	IL4R/IL2	2
BP	GO:0009060	aerobic respiration	4/132	197/18870	0.04972214	0.08796663	0.04021202	CDK1/CCNB1/CYCS/TNF	4
BP	GO:0006090	pyruvate metabolic process	3/132	118/18870	0.04998316	0.08835188	0.04038813	APP/STAT3/INSR	3
BP	GO:0043500	muscle adaptation	3/132	118/18870	0.04998316	0.08835188	0.04038813	SCN5A/EDN1/PARP1	3
CC	GO:0045121	membrane raft	16/133	286/19886	8.73E-11	1.35E-08	9.97E-09	SCN5A/TNF/APP/SLC2A4/SELE/CAV1/GJA1/ABCG2/IG	16
CC	GO:0098857	membrane microdomain	16/133	287/19886	9.20E-11	1.35E-08	9.97E-09	SCN5A/TNF/APP/SLC2A4/SELE/CAV1/GJA1/ABCG2/IG	16
CC	GO:1902911	protein kinase complex	11/133	148/19886	4.63E-09	4.54E-07	3.35E-07	CDK1/CCNB1/CDK2/CDKN1A/CCND1/CDK4/IGF1R/CS	11
CC	GO:0009897	external side of plasma membrane	16/133	387/19886	7.01E-09	5.15E-07	3.80E-07	CD163/TNF/IL6R/IL2RA/IL4R/SLC2A4/ITGB3/SELE/ABC	16
CC	GO:0061695	transferase complex, transferring phosphorus-containing groups	14/133	306/19886	1.84E-08	1.08E-06	7.97E-07	CDK1/CCNB1/CDK2/CDKN1A/CCND1/CDK4/IGF1R/PII	14
CC	GO:0000307	cyclin-dependent protein kinase holoenzyme complex	7/133	54/19886	6.96E-08	3.41E-06	2.52E-06	CDK1/CCNB1/CDK2/CDKN1A/CCND1/CDK4/CDK6	7
CC	GO:0097136	Bcl-2 family protein complex	4/133	10/19886	3.89E-07	1.63E-05	1.21E-05	BCL2/BAX/BAD/MCL1	4
CC	GO:0005901	caveola	7/133	81/19886	1.18E-06	4.32E-05	3.19E-05	SCN5A/SELE/CAV1/IGF1R/SRC/INSR/PTGS2	7
CC	GO:1902554	serine/threonine protein kinase complex	8/133	128/19886	2.36E-06	7.72E-05	5.69E-05	CDK1/CCNB1/CDK2/CDKN1A/CCND1/CDK4/CSNK2A1	8
CC	GO:0031253	cell projection membrane	12/133	352/19886	4.42E-06	0.00012993	9.58E-05	HSP90AA1/MTTP/DRD2/ITGB3/ABCG2/ADORA1/EGFR	12
CC	GO:0044853	plasma membrane raft	7/133	112/19886	1.03E-05	0.00027599	0.00020356	SCN5A/SELE/CAV1/IGF1R/SRC/INSR/PTGS2	7
CC	GO:0045177	apical part of cell	13/133	469/19886	1.62E-05	0.000396	0.00029208	HSP90AA1/IL6R/APP/ITGB3/GJA1/ABCG2/CLDN4/CA2	13
CC	GO:0062023	collagen-containing extracellular matrix	12/133	429/19886	3.18E-05	0.00071896	0.00053027	HSP90AA1/PRSS1/ADIPOQ/TIMP1/COL1A1/COL3A1/M	12
CC	GO:0045178	basal part of cell	10/133	301/19886	3.56E-05	0.00074741	0.00055126	HSP90AA1/MTTP/EDN1/CLDN4/ADORA1/CA12/EGFR/	10
CC	GO:0005788	endoplasmic reticulum lumen	10/133	313/19886	4.95E-05	0.00096959	0.00071513	MTTP/TIMP1/APP/EDN1/COL1A1/COL3A1/SPP1/FLT3/	10
CC	GO:0016324	apical plasma membrane	11/133	403/19886	8.54E-05	0.0015693	0.00115745	HSP90AA1/IL6R/ITGB3/GJA1/ABCG2/CLDN4/CA12/AB	11
CC	GO:0009925	basal plasma membrane	9/133	282/19886	0.00011991	0.00194155	0.001432	HSP90AA1/MTTP/CLDN4/ADORA1/CA12/EGFR/ABCC1	9
CC	GO:0005635	nuclear envelope	12/133	494/19886	0.00012217	0.00194155	0.001432	BCL2/BAX/CDK2/CCND1/CDK4/APP/SULT1E1/ALOX5/	12
CC	GO:0030673	axolemma	3/133	15/19886	0.00012547	0.00194155	0.001432	ADORA1/ADORA2A/MAPT	3
CC	GO:0031256	leading edge membrane	7/133	179/19886	0.00020426	0.00300256	0.00221457	ITGB3/ADORA1/EGFR/ADORA2A/SRC/MAPT/INSR	7
CC	GO:0031983	vesicle lumen	9/133	326/19886	0.00035178	0.00492496	0.00363244	HSP90AA1/TIMP1/APP/ALOX5/TTR/ARG1/EGFR/MPO/	9
CC	GO:0005798	Golgi-associated vesicle	5/133	94/19886	0.00042301	0.00565291	0.00416935	APP/SLC2A4/GJA1/TYR/CFTR	5
CC	GO:0043025	neuronal cell body	11/133	489/19886	0.00045245	0.00578348	0.00426566	HSP90AA1/TNF/DRD2/APP/ADORA1/TOP1/IGF1R/AD	11
CC	GO:0032589	neuron projection membrane	4/133	60/19886	0.00069781	0.00854822	0.00630481	ADORA1/ADORA2A/MAPT/INSR	4
CC	GO:0016328	lateral plasma membrane	4/133	67/19886	0.00105803	0.01244241	0.009177	SCN5A/DRD2/CLDN4/ABCC1	4
CC	GO:0043073	germ cell nucleus	4/133	68/19886	0.00111839	0.01264638	0.00932744	TP53/CDK2/TOP1/TOP2A	4
CC	GO:0031528	microvillus membrane	3/133	33/19886	0.00137791	0.01445423	0.01066084	MTTP/ITGB3/CA9	3
CC	GO:0016323	basolateral plasma membrane	7/133	249/19886	0.00144908	0.01445423	0.01066084	HSP90AA1/MTTP/ADORA1/CA12/EGFR/ABCC1/CA9	7
CC	GO:0098687	chromosomal region	9/133	399/19886	0.00147215	0.01445423	0.01066084	CDK1/CCNB1/CDK2/BIRC5/PARP1/PLK1/TERT/TOP2A/	9
CC	GO:0034774	secretory granule lumen	8/133	322/19886	0.00147492	0.01445423	0.01066084	HSP90AA1/TIMP1/APP/ALOX5/TTR/ARG1/MPO/CTSD	8
CC	GO:0060205	cytoplasmic vesicle lumen	8/133	325/19886	0.00156361	0.01482909	0.01093732	HSP90AA1/TIMP1/APP/ALOX5/TTR/ARG1/MPO/CTSD	8
CC	GO:0090575	RNA polymerase II transcription regulator complex	7/133	254/19886	0.00162316	0.01491279	0.01099905	RXRA/FOS/MYC/RB1/STAT3/STAT1/NR1H3	7
CC	GO:0005641	nuclear envelope lumen	2/133	10/19886	0.00192885	0.01718433	0.01267444	APP/ALOX5	2
CC	GO:0031252	cell leading edge	9/133	423/19886	0.00218987	0.01893597	0.01396638	ITGB3/ADORA1/EGFR/ADORA2A/SRC/AKT1/MAPT/IN	9
CC	GO:0030139	endocytic vesicle	8/133	348/19886	0.00239298	0.02010106	0.0148257	HSP90AA1/CD163/DRD2/CAV1/SYK/EGFR/MPO/CFTR	8
CC	GO:0042383	sarcolemma	5/133	141/19886	0.00259615	0.02120187	0.01563761	SCN5A/SLC2A4/CAV1/IGF1R/ALOX12	5

CC	GO:0005583	fibrillar collagen trimer	2/133	12/19886	0.0028043	0.02169641	0.01600236	COL1A1/COL3A1	2
CC	GO:0098643	banded collagen fibril	2/133	12/19886	0.0028043	0.02169641	0.01600236	COL1A1/COL3A1	2
CC	GO:0044292	dendrite terminus	2/133	13/19886	0.00329969	0.02487457	0.01834645	HSP90AA1/SRC	2
CC	GO:0043209	myelin sheath	3/133	46/19886	0.00359882	0.02645134	0.0195094	HSP90AA1/BCL2/CA2	3
CC	GO:0000228	nuclear chromosome	6/133	228/19886	0.00435877	0.03125559	0.02305281	BIRC5/PARP1/TOP1/PLK1/CSNK2A1/TOP2A	6
CC	GO:0030315	T-tubule	3/133	51/19886	0.00481884	0.03373188	0.02487922	SCN5A/SLC2A4/IGF1R	3
CC	GO:0031965	nuclear membrane	7/133	311/19886	0.00497875	0.03400479	0.02508051	BCL2/CCND1/CDK4/SULT1E1/ALOX5/EGFR/PTGS2	7
CC	GO:0001673	male germ cell nucleus	3/133	52/19886	0.00508915	0.03400479	0.02508051	CDK2/TOP1/TOP2A	3
CC	GO:0000781	chromosome, telomeric region	5/133	171/19886	0.00588808	0.03768394	0.0277941	CDK1/CDK2/PARP1/TERT/APEX1	5
CC	GO:0098802	plasma membrane signaling receptor complex	7/133	321/19886	0.00589613	0.03768394	0.0277941	DRD2/IL6R/IL2RA/ITGB3/SYK/IGF1R/INSR	7
CC	GO:0016605	PML body	4/133	109/19886	0.00619288	0.03849587	0.02839294	TP53/RB1/CSNK2A1/TERT	4
CC	GO:0043197	dendritic spine	5/133	174/19886	0.00632888	0.03849587	0.02839294	DRD2/APP/ADORA1/PTK2/MAPT	5
CC	GO:0005775	vacuolar lumen	5/133	176/19886	0.00663532	0.03849587	0.02839294	HSP90AA1/TTR/ARG1/MPO/CTSD	5
CC	GO:0044309	neuron spine	5/133	176/19886	0.00663532	0.03849587	0.02839294	DRD2/APP/ADORA1/PTK2/MAPT	5
CC	GO:0042470	melanosome	4/133	112/19886	0.00680879	0.03849587	0.02839294	HSP90AA1/ITGB3/CTSD/TYR	4
CC	GO:0048770	pigment granule	4/133	112/19886	0.00680879	0.03849587	0.02839294	HSP90AA1/ITGB3/CTSD/TYR	4
CC	GO:0031526	brush border membrane	3/133	60/19886	0.00758094	0.04205277	0.03101637	HSP90AA1/MTTP/ABCG2	3
CC	GO:0000940	outer kinetochore	2/133	20/19886	0.00779556	0.0424425	0.03130381	CCNB1/PLK1	2
CC	GO:0005819	spindle	8/133	431/19886	0.00851406	0.0442478	0.03263533	CDK1/CCNB1/RB1/APP/BIRC5/RASSF1/PLK1/AKT1	8
CC	GO:0097038	perinuclear endoplasmic reticulum	2/133	21/19886	0.00857866	0.0442478	0.03263533	NOX4/PIK3R1	2
CC	GO:0098644	complex of collagen trimers	2/133	21/19886	0.00857866	0.0442478	0.03263533	COL1A1/COL3A1	2
CC	GO:1904813	ficolin-1-rich granule lumen	4/133	124/19886	0.00967505	0.04904252	0.03617171	HSP90AA1/ALOX5/MMP9/CTSD	4
CC	GO:0071682	endocytic vesicle lumen	2/133	23/19886	0.01024556	0.05027434	0.03708025	HSP90AA1/MPO	2
CC	GO:0044304	main axon	3/133	67/19886	0.01026007	0.05027434	0.03708025	ADORA1/ADORA2A/MAPT	3
CC	GO:0150034	distal axon	6/133	276/19886	0.01072304	0.05168153	0.03811814	HSP90AA1/DRD2/APP/ADORA1/SRC/MAPT	6
CC	GO:0070160	tight junction	4/133	131/19886	0.01166145	0.05529785	0.04078538	CCND1/CDK4/GJA1/CLDN4	4
CC	GO:0046930	pore complex	2/133	26/19886	0.01299061	0.06062283	0.04471286	BCL2/BAX	2
CC	GO:0005905	clathrin-coated pit	3/133	75/19886	0.01391568	0.06392517	0.04714853	APP/SLC2A4/SELE	3
CC	GO:0017053	transcription repressor complex	3/133	77/19886	0.01493065	0.06650926	0.04905445	TP53/MYC/CCND1	3
CC	GO:0048786	presynaptic active zone	3/133	77/19886	0.01493065	0.06650926	0.04905445	APP/ADORA1/ADORA2A	3
MF	GO:0019838	growth factor binding	11/133	135/18496	3.67E-09	9.85E-07	6.29E-07	SCN5A/IL6R/IL2RA/ITGB3/COL1A1/COL3A1/FLT3/IGF1	11
MF	GO:0004713	protein tyrosine kinase activity	11/133	138/18496	4.64E-09	9.85E-07	6.29E-07	FLT3/SYK/IGF1R/EGFR/SRC/PTK2/KDR/MET/ALK/AXL/I	11
MF	GO:0019903	protein phosphatase binding	11/133	142/18496	6.27E-09	9.85E-07	6.29E-07	HSP90AA1/BCL2/TP53/BAD/STAT3/STAT1/EGFR/PIK3F	11
MF	GO:0140297	DNA-binding transcription factor binding	18/133	477/18496	9.56E-09	9.85E-07	6.29E-07	RXRA/AR/RELA/BCL2/FOS/TP53/MYC/RB1/NFKBIA/ST/	18
MF	GO:0004714	transmembrane receptor protein tyrosine kinase activity	8/133	60/18496	1.08E-08	9.85E-07	6.29E-07	FLT3/IGF1R/EGFR/KDR/MET/ALK/AXL/INSR	8
MF	GO:0019902	phosphatase binding	12/133	189/18496	1.19E-08	9.85E-07	6.29E-07	HSP90AA1/BCL2/TP53/BAD/STAT3/STAT1/SYK/EGFR/I	12
MF	GO:0044389	ubiquitin-like protein ligase binding	15/133	327/18496	1.38E-08	9.85E-07	6.29E-07	KCNH2/SCN5A/HSP90AA1/RELA/BCL2/TP53/CCNB1/(	15
MF	GO:0061629	RNA polymerase II-specific DNA-binding transcription factor binding	15/133	344/18496	2.72E-08	1.69E-06	1.08E-06	RXRA/AR/RELA/FOS/TP53/RB1/NFKBIA/STAT3/STAT1/	15
MF	GO:0004879	nuclear receptor activity	7/133	52/18496	8.67E-08	4.32E-06	2.76E-06	RXRA/AR/STAT3/NR1H3/ESR1/ESR2/AHR	7
MF	GO:0098531	ligand-activated transcription factor activity	7/133	52/18496	8.67E-08	4.32E-06	2.76E-06	RXRA/AR/STAT3/NR1H3/ESR1/ESR2/AHR	7
MF	GO:0019199	transmembrane receptor protein kinase activity	8/133	79/18496	9.83E-08	4.45E-06	2.84E-06	FLT3/IGF1R/EGFR/KDR/MET/ALK/AXL/INSR	8
MF	GO:0001221	transcription coregulator binding	9/133	112/18496	1.15E-07	4.78E-06	3.05E-06	RXRA/AR/RELA/FOS/MYC/STAT1/TERT/ESR1/AHR	9
MF	GO:0031625	ubiquitin protein ligase binding	13/133	308/18496	3.51E-07	1.35E-05	8.59E-06	KCNH2/SCN5A/HSP90AA1/RELA/BCL2/TP53/CDKN1A	13
MF	GO:0001046	core promoter sequence-specific DNA binding	6/133	41/18496	4.51E-07	1.61E-05	1.02E-05	RELA/FOS/TP53/MYC/STAT1/MMP12	6
MF	GO:0016702	oxidoreductase activity, acting on single donors with incorporation of mole	5/133	24/18496	6.79E-07	2.17E-05	1.38E-05	ALOX5/ALOX15/ALOX12/PTGS1/PTGS2	5
MF	GO:0001223	transcription coactivator binding	6/133	44/18496	6.96E-07	2.17E-05	1.38E-05	AR/RELA/STAT1/TERT/ESR1/AHR	6
MF	GO:0020037	heme binding	9/133	141/18496	8.22E-07	2.29E-05	1.46E-05	CYCS/CYP19A1/NOX4/CYP1B1/MPO/SRC/CYP51A1/P	9
MF	GO:0016701	oxidoreductase activity, acting on single donors with incorporation of mole	5/133	25/18496	8.44E-07	2.29E-05	1.46E-05	ALOX5/ALOX15/ALOX12/PTGS1/PTGS2	5
MF	GO:0002020	protease binding	9/133	142/18496	8.72E-07	2.29E-05	1.46E-05	BCL2/TP53/TIMP1/TNF/ITGB3/COL1A1/COL3A1/GSK3	9

MF	GO:0004032	alditol:NADP+ 1-oxidoreductase activity	4/133	12/18496	1.21E-06	3.01E-05	1.92E-05	AKR1B1/AKR1B10/AKR1C3/AKR1A1	4
MF	GO:0046906	tetrapyrrole binding	9/133	151/18496	1.46E-06	3.46E-05	2.21E-05	CYCS/CYP19A1/NOX4/CYP1B1/MPO/SRC/CYP51A1/PT	9
MF	GO:0017171	serine hydrolase activity	10/133	196/18496	1.55E-06	3.50E-05	2.24E-05	PRSS1/MMP9/MMP2/MMP12/F2/MMP13/MMP3/ACH	10
MF	GO:0019207	kinase regulator activity	11/133	256/18496	2.46E-06	5.16E-05	3.29E-05	CCNB1/ADIPOQ/CDKN1A/CCND1/CDK4/CXCL10/EGF	11
MF	GO:0004674	protein serine/threonine kinase activity	14/133	427/18496	2.49E-06	5.16E-05	3.29E-05	CDK1/CDK2/CDK4/SYK/GSK3B/TOP1/EGFR/PIM1/PLK1	14
MF	GO:0042562	hormone binding	7/133	85/18496	2.63E-06	5.23E-05	3.34E-05	AR/TTR/IGF1R/EGFR/PIK3R1/SHBG/INSR	7
MF	GO:0015562	efflux transmembrane transporter activity	4/133	15/18496	3.28E-06	6.28E-05	4.01E-05	GJA1/ABCG2/ABCC1/ABCB1	4
MF	GO:0030332	cyclin binding	5/133	34/18496	4.20E-06	7.74E-05	4.94E-05	CDK1/CDK2/CDKN1A/CDK4/CDK6	5
MF	GO:0005126	cytokine receptor binding	11/133	273/18496	4.55E-06	8.07E-05	5.15E-05	TNF/IL6R/ITGB3/STAT1/CCL2/CXCL11/CXCL2/CXCL10/	11
MF	GO:0004252	serine-type endopeptidase activity	9/133	174/18496	4.70E-06	8.07E-05	5.15E-05	PRSS1/MMP9/MMP2/MMP12/F2/MMP13/MMP3/F3/N	9
MF	GO:0019209	kinase activator activity	8/133	133/18496	5.33E-06	8.86E-05	5.65E-05	CCNB1/ADIPOQ/CDKN1A/CCND1/EGFR/PIK3R1/ALK/I	8
MF	GO:0004896	cytokine receptor activity	7/133	96/18496	5.94E-06	9.54E-05	6.09E-05	IL6R/IL2RA/IL4R/FLT3/CXCR1/GPR35/F3	7
MF	GO:0005125	cytokine activity	10/133	237/18496	8.41E-06	0.00013082	8.35E-05	ADIPOQ/TIMP1/TNF/EDN1/CCL2/CXCL11/CXCL2/CXC	10
MF	GO:0005496	steroid binding	7/133	102/18496	8.88E-06	0.00013401	8.55E-05	AR/CAV1/SULT1E1/SHBG/NR1H3/ESR1/ESR2	7
MF	GO:0097110	scaffold protein binding	6/133	68/18496	9.37E-06	0.00013731	8.77E-05	KCNH2/SCN5A/HSP90AA1/CD163/SYK/SRC	6
MF	GO:0008236	serine-type peptidase activity	9/133	192/18496	1.04E-05	0.00014846	9.48E-05	PRSS1/MMP9/MMP2/MMP12/F2/MMP13/MMP3/F3/N	9
MF	GO:0005158	insulin receptor binding	4/133	22/18496	1.69E-05	0.00023385	0.00014928	IGF1R/PIK3R1/SRC/PTPN11	4
MF	GO:0008106	alcohol dehydrogenase (NADP+) activity	4/133	23/18496	2.03E-05	0.0002739	0.00017484	AKR1B1/AKR1B10/AKR1C3/AKR1A1	4
MF	GO:0001091	RNA polymerase II general transcription initiation factor binding	4/133	25/18496	2.87E-05	0.0003671	0.00023433	AR/TP53/ESR1/AHR	4
MF	GO:0043548	phosphatidylinositol 3-kinase binding	4/133	25/18496	2.87E-05	0.0003671	0.00023433	IGF1R/PIK3R1/AXL/INSR	4
MF	GO:0019887	protein kinase regulator activity	9/133	226/18496	3.80E-05	0.00047253	0.00030164	CCNB1/ADIPOQ/CDKN1A/CCND1/CDK4/CXCL10/EGF	9
MF	GO:0031072	heat shock protein binding	7/133	128/18496	3.89E-05	0.00047302	0.00030195	BAX/CDK1/ADORA1/KDR/CSNK2A1/MAPT/AHR	7
MF	GO:0140296	general transcription initiation factor binding	5/133	54/18496	4.25E-05	0.00050389	0.00032166	AR/RELA/TP53/ESR1/AHR	5
MF	GO:0051087	protein-folding chaperone binding	7/133	133/18496	4.98E-05	0.00057148	0.0003648	BAX/TP53/CDK1/BIRC5/TERT/MAPT/CFTR	7
MF	GO:0004693	cyclin-dependent protein serine/threonine kinase activity	4/133	29/18496	5.28E-05	0.00057148	0.0003648	CDK1/CDK2/CDK4/CDK6	4
MF	GO:0051721	protein phosphatase 2A binding	4/133	29/18496	5.28E-05	0.00057148	0.0003648	BCL2/TP53/STAT1/MAPT	4
MF	GO:0097472	cyclin-dependent protein kinase activity	4/133	29/18496	5.28E-05	0.00057148	0.0003648	CDK1/CDK2/CDK4/CDK6	4
MF	GO:0034056	estrogen response element binding	3/133	11/18496	5.75E-05	0.00059657	0.00038081	AR/ESR1/ESR2	3
MF	GO:0070513	death domain binding	3/133	11/18496	5.75E-05	0.00059657	0.00038081	BCL2/BAX/MCL1	3
MF	GO:0004033	aldo-keto reductase (NADP) activity	4/133	30/18496	6.06E-05	0.00060452	0.00038589	AKR1B1/AKR1B10/AKR1C3/AKR1A1	4
MF	GO:0106310	protein serine kinase activity	11/133	361/18496	6.07E-05	0.00060452	0.00038589	CDK1/CDK2/CDK4/GSK3B/PIM1/PLK1/CSNK2A1/AKT1	11
MF	GO:0140375	immune receptor activity	7/133	147/18496	9.37E-05	0.00090952	0.00058058	IL6R/IL2RA/IL4R/FLT3/CXCR1/GPR35/F3	7
MF	GO:0031994	insulin-like growth factor I binding	3/133	13/18496	9.86E-05	0.00090952	0.00058058	ITGB3/IGF1R/INSR	3
MF	GO:0043560	insulin receptor substrate binding	3/133	13/18496	9.86E-05	0.00090952	0.00058058	IGF1R/PIK3R1/INSR	3
MF	GO:0052650	NADP-retinol dehydrogenase activity	3/133	13/18496	9.86E-05	0.00090952	0.00058058	AKR1B1/AKR1B10/AKR1C3	3
MF	GO:0005506	iron ion binding	7/133	153/18496	0.00012033	0.00108952	0.00069548	CYP19A1/XDH/ALOX5/CYP1B1/ALOX15/ALOX12/CYP	7
MF	GO:0008559	ABC-type xenobiotic transporter activity	3/133	14/18496	0.00012486	0.00109484	0.00069888	ABCG2/ABCC1/ABCB1	3
MF	GO:0005178	integrin binding	7/133	154/18496	0.00012531	0.00109484	0.00069888	ITGB3/COL3A1/SPP1/SYK/SRC/PTK2/KDR	7
MF	GO:0004222	metalloendopeptidase activity	6/133	110/18496	0.0001434	0.00123125	0.00078596	MMP9/MMP2/MMP12/MMP13/MMP3/MMP1	6
MF	GO:0004089	carbonate dehydratase activity	3/133	15/18496	0.00015525	0.00131045	0.00083652	CA2/CA12/CA9	3
MF	GO:0140313	molecular sequestering activity	4/133	40/18496	0.00019106	0.00155805	0.00099457	CDKN1A/NFKBIA/STAT3/CAV1	4
MF	GO:0042379	chemokine receptor binding	5/133	74/18496	0.00019298	0.00155805	0.00099457	STAT1/CCL2/CXCL11/CXCL2/CXCL10	5
MF	GO:0046982	protein heterodimerization activity	10/133	344/18496	0.00019397	0.00155805	0.00099457	BCL2/BAX/TP53/MTTP/MCL1/CAV1/ADORA1/PIK3R1/	10
MF	GO:0051879	Hsp90 protein binding	4/133	42/18496	0.00023141	0.00182927	0.0011677	KDR/CSNK2A1/MAPT/AHR	4
MF	GO:0001664	G protein-coupled receptor binding	9/133	291/18496	0.00025889	0.00201451	0.00128595	EDN1/STAT1/CCL2/CXCL11/CXCL2/CXCL10/ADORA1/	9
MF	GO:0045236	CXCR chemokine receptor binding	3/133	18/18496	0.00027407	0.00207848	0.00132678	CXCL11/CXCL2/CXCL10	3
MF	GO:0030295	protein kinase activator activity	6/133	124/18496	0.00027546	0.00207848	0.00132678	CCNB1/ADIPOQ/CDKN1A/CCND1/EGFR/ALK	6
MF	GO:0016705	oxidoreductase activity, acting on paired donors, with incorporation or red	7/133	179/18496	0.00031584	0.00233664	0.00149158	CYP19A1/CYP1B1/AKR1C3/CYP51A1/PTGS1/TYR/PTGS	7
MF	GO:0000979	RNA polymerase II core promoter sequence-specific DNA binding	3/133	19/18496	0.00032375	0.00233664	0.00149158	RELA/FOS/STAT1	3

MF	GO:0005520	insulin-like growth factor binding	3/133	19/18496	0.00032375	0.00233664	0.00149158	ITGB3/IGF1R/INSR	3
MF	GO:0051117	ATPase binding	5/133	84/18496	0.00034924	0.00248461	0.00158603	AR/CAV1/EGFR/SRC/ESR1	5
MF	GO:0140359	ABC-type transporter activity	4/133	48/18496	0.00038911	0.00272925	0.0017422	ABCG2/ABCC1/ABCB1/CFTR	4
MF	GO:0008009	chemokine activity	4/133	49/18496	0.00042135	0.00291436	0.00186036	CCL2/CXCL11/CXCL2/CXCL10	4
MF	GO:0016538	cyclin-dependent protein serine/threonine kinase regulator activity	4/133	50/18496	0.00045546	0.00310711	0.0019834	CCNB1/CDKN1A/CCND1/CDK4	4
MF	GO:0016922	nuclear receptor binding	6/133	137/18496	0.0004699	0.0031623	0.00201863	RXRA/STAT1/FLT3/PARP1/SRC/ESR1	6
MF	GO:0004175	endopeptidase activity	10/133	389/18496	0.00051319	0.00340758	0.00217521	PRSS1/MMP9/MMP2/MMP12/F2/MMP13/MMP3/CTSI	10
MF	GO:0031406	carboxylic acid binding	7/133	195/18496	0.00052833	0.00346192	0.00220989	RXRA/ADIPOQ/SELE/PLA2G1B/AKR1C3/TYMS/DHFR	7
MF	GO:0051213	dioxygenase activity	5/133	93/18496	0.00055841	0.00356525	0.00227585	ALOX5/ALOX15/ALOX12/PTGS1/PTGS2	5
MF	GO:0051219	phosphoprotein binding	5/133	93/18496	0.00055841	0.00356525	0.00227585	RB1/SYK/PIK3R1/SRC/PTPN11	5
MF	GO:0043274	phospholipase binding	3/133	23/18496	0.00057939	0.00360673	0.00230233	SELE/SYK/SRC	3
MF	GO:0140311	protein sequestering activity	3/133	23/18496	0.00057939	0.00360673	0.00230233	CDKN1A/NFKBIA/CAV1	3
MF	GO:0003707	nuclear steroid receptor activity	3/133	25/18496	0.0007446	0.00456908	0.00291664	RXRA/ESR1/ESR2	3
MF	GO:0043177	organic acid binding	7/133	207/18496	0.00075234	0.00456908	0.00291664	RXRA/ADIPOQ/SELE/PLA2G1B/AKR1C3/TYMS/DHFR	7
MF	GO:0001098	basal transcription machinery binding	4/133	60/18496	0.00091241	0.00540927	0.00345297	AR/TP53/ESR1/AHR	4
MF	GO:0001099	basal RNA polymerase II transcription machinery binding	4/133	60/18496	0.00091241	0.00540927	0.00345297	AR/TP53/ESR1/AHR	4
MF	GO:0004497	monooxygenase activity	5/133	104/18496	0.0009279	0.0054364	0.00347029	CYP19A1/CYP1B1/AKR1C3/CYP51A1/TYR	5
MF	GO:0016836	hydro-lyase activity	4/133	61/18496	0.00097105	0.00562304	0.00358942	CA2/CA12/CYP1B1/CA9	4
MF	GO:0051059	NF-kappaB binding	3/133	29/18496	0.00115837	0.00663067	0.00423264	RELA/NFKBIA/GSK3B	3
MF	GO:0005518	collagen binding	4/133	68/18496	0.00145784	0.00825005	0.00526636	MMP9/MMP12/MMP13/ACHE	4
MF	GO:0071889	14-3-3 protein binding	3/133	32/18496	0.00154786	0.00856483	0.0054673	TP53/AKT1/ESR1	3
MF	GO:0097718	disordered domain specific binding	3/133	32/18496	0.00154786	0.00856483	0.0054673	HSP90AA1/TP53/RB1	3
MF	GO:0008201	heparin binding	6/133	174/18496	0.00162823	0.00891053	0.00568797	APP/CXCL11/CXCL10/F2/MPO/ALK	6
MF	GO:0031490	chromatin DNA binding	5/133	120/18496	0.00175521	0.00950104	0.00606492	RELA/STAT3/RUNX2/NR1H3/APEX1	5
MF	GO:1990782	protein tyrosine kinase binding	5/133	123/18496	0.00195629	0.01047562	0.00668704	HSP90AA1/TP53/NOX4/PIK3R1/PTPN11	5
MF	GO:0016248	channel inhibitor activity	3/133	35/18496	0.00201066	0.01065222	0.00679977	BCL2/CAV1/CFTR	3
MF	GO:0016616	oxidoreductase activity, acting on the CH-OH group of donors, NAD or N/	5/133	125/18496	0.00209938	0.01100518	0.00702508	AKR1B1/AKR1B10/AKR1C3/AKR1A1/HMGCR	5
MF	GO:0001228	DNA-binding transcription activator activity, RNA polymerase II-specific	10/133	471/18496	0.00216324	0.01107444	0.00706929	AR/RELA/FOS/TP53/MYC/KLF7/STAT3/RUNX2/NR1H3/	10
MF	GO:0008237	metallopeptidase activity	6/133	185/18496	0.002218	0.01107444	0.00706929	MMP9/MMP2/MMP12/MMP13/MMP3/MMP1	6
MF	GO:0001094	TFIID-class transcription factor complex binding	2/133	10/18496	0.00222378	0.01107444	0.00706929	TP53/AHR	2
MF	GO:0032052	bile acid binding	2/133	10/18496	0.00222378	0.01107444	0.00706929	PLA2G1B/AKR1C3	2
MF	GO:0043225	ATPase-coupled inorganic anion transmembrane transporter activity	2/133	10/18496	0.00222378	0.01107444	0.00706929	ABCC1/CFTR	2
MF	GO:0042826	histone deacetylase binding	5/133	127/18496	0.00224997	0.01109391	0.00708172	HSP90AA1/RELA/TP53/CCND1/PARP1	5
MF	GO:0001216	DNA-binding transcription activator activity	10/133	475/18496	0.00229998	0.01114506	0.00711437	AR/RELA/FOS/TP53/MYC/KLF7/STAT3/RUNX2/NR1H3/	10
MF	GO:0016835	carbon-oxygen lyase activity	4/133	77/18496	0.0023051	0.01114506	0.00711437	CA2/CA12/CYP1B1/CA9	4
MF	GO:0042910	xenobiotic transmembrane transporter activity	3/133	37/18496	0.00236214	0.01131102	0.00722031	ABCG2/ABCC1/ABCB1	3
MF	GO:0001618	virus receptor activity	4/133	79/18496	0.00253122	0.01200522	0.00766345	CDK1/ITGB3/EGFR/AXL	4
MF	GO:0140272	exogenous protein binding	4/133	80/18496	0.0026498	0.01233528	0.00787414	CDK1/ITGB3/EGFR/AXL	4
MF	GO:0033218	amide binding	9/133	405/18496	0.00265081	0.01233528	0.00787414	RXRA/RELA/NFKBIA/IGF1R/PIK3R1/ACHE/TYMS/INSR/	9
MF	GO:0048407	platelet-derived growth factor binding	2/133	11/18496	0.00270518	0.01233528	0.00787414	COL1A1/COL3A1	2
MF	GO:0050998	nitric-oxide synthase binding	2/133	11/18496	0.00270518	0.01233528	0.00787414	SCN5A/CAV1	2
MF	GO:0016620	oxidoreductase activity, acting on the aldehyde or oxo group of donors, N	3/133	39/18496	0.00274943	0.01233528	0.00787414	AKR1B1/AKR1B10/AKR1C3	3
MF	GO:0030331	nuclear estrogen receptor binding	3/133	39/18496	0.00274943	0.01233528	0.00787414	PARP1/SRC/ESR1	3
MF	GO:0016614	oxidoreductase activity, acting on CH-OH group of donors	5/133	135/18496	0.00293184	0.01303624	0.00832159	AKR1B1/AKR1B10/AKR1C3/AKR1A1/HMGCR	5
MF	GO:0072341	modified amino acid binding	4/133	84/18496	0.00316226	0.01386397	0.00884997	NOX4/AXL/TYMS/DHFR	4
MF	GO:0042169	SH2 domain binding	3/133	41/18496	0.00317368	0.01386397	0.00884997	SYK/SRC/PTK2	3
MF	GO:0034452	dynactin binding	2/133	12/18496	0.00323096	0.01399147	0.00893135	GSK3B/MAPT	2
MF	GO:0008013	beta-catenin binding	4/133	85/18496	0.0033002	0.01416809	0.0090441	AR/GJA1/GSK3B/ESR1	4
MF	GO:0005542	folic acid binding	2/133	13/18496	0.00380048	0.01617639	0.01032608	TYMS/DHFR	2

MF	GO:0004715	non-membrane spanning protein tyrosine kinase activity	3/133	45/18496	0.00413734	0.01746099	0.0111461	SYK/SRC/PTK2	3
MF	GO:1990825	sequence-specific mRNA binding	2/133	14/18496	0.00441308	0.01846818	0.01178903	TYMS/DHFR	2
MF	GO:0001784	phosphotyrosine residue binding	3/133	47/18496	0.00467872	0.01941669	0.01239451	SYK/PIK3R1/PTPN11	3
MF	GO:0016903	oxidoreductase activity, acting on the aldehyde or oxo group of donors	3/133	48/18496	0.00496469	0.02043321	0.01304339	AKR1B1/AKR1B10/AKR1C3	3
MF	GO:0042626	ATPase-coupled transmembrane transporter activity	4/133	96/18496	0.00509669	0.02080454	0.01328043	ABCG2/ABCC1/ABCB1/CFTR	4
MF	GO:0017046	peptide hormone binding	3/133	49/18496	0.005261	0.02130063	0.01359711	IGF1R/PIK3R1/INSR	3
MF	GO:0000900	mRNA regulatory element binding translation repressor activity	2/133	16/18496	0.00576499	0.02260604	0.01443041	TYMS/DHFR	2
MF	GO:0005159	insulin-like growth factor receptor binding	2/133	16/18496	0.00576499	0.02260604	0.01443041	PIK3R1/INSR	2
MF	GO:0061575	cyclin-dependent protein serine/threonine kinase activator activity	2/133	16/18496	0.00576499	0.02260604	0.01443041	CCNB1/CCND1	2
MF	GO:0070402	NADPH binding	2/133	16/18496	0.00576499	0.02260604	0.01443041	HMGCR/DHFR	2
MF	GO:0005080	protein kinase C binding	3/133	51/18496	0.005885	0.02289633	0.01461571	ITGB3/SRC/TOP2A	3
MF	GO:0061134	peptidase regulator activity	6/133	228/18496	0.00615327	0.02375447	0.0151635	BAD/TIMP1/APP/BIRC5/XIAP/CAV1	6
MF	GO:0008525	phosphatidylcholine transporter activity	2/133	17/18496	0.00650305	0.02453423	0.01566125	MTTP/ABCB1	2
MF	GO:0034185	apolipoprotein binding	2/133	17/18496	0.00650305	0.02453423	0.01566125	MTTP/MAPT	2
MF	GO:0046624	sphingolipid transporter activity	2/133	17/18496	0.00650305	0.02453423	0.01566125	MTTP/ABCB1	2
MF	GO:0004601	peroxidase activity	3/133	55/18496	0.00726117	0.02686127	0.0171467	MPO/PTGS1/PTGS2	3
MF	GO:0050840	extracellular matrix binding	3/133	55/18496	0.00726117	0.02686127	0.0171467	ITGB3/SPP1/ACHE	3
MF	GO:0008301	DNA binding, bending	2/133	18/18496	0.00728167	0.02686127	0.0171467	TOP1/TOP2A	2
MF	GO:0005539	glycosaminoglycan binding	6/133	240/18496	0.00783644	0.0286952	0.01831738	APP/CXCL11/CXCL10/F2/MPO/ALK	6
MF	GO:0016684	oxidoreductase activity, acting on peroxide as acceptor	3/133	57/18496	0.00801468	0.02913364	0.01859725	MPO/PTGS1/PTGS2	3
MF	GO:0045309	protein phosphorylated amino acid binding	3/133	59/18496	0.00881262	0.03180205	0.02030061	SYK/PIK3R1/PTPN11	3
MF	GO:0035173	histone kinase activity	2/133	20/18496	0.00895818	0.03209477	0.02048746	CDK1/CDK2	2
MF	GO:0042277	peptide binding	7/133	325/18496	0.00917727	0.03259399	0.02080614	RXRA/RELA/NFKBIA/IGF1R/PIK3R1/ACHE/INSR	7
MF	GO:0043539	protein serine/threonine kinase activator activity	3/133	60/18496	0.00922842	0.03259399	0.02080614	CCNB1/ADIPOQ/CCND1	3
MF	GO:0016500	protein-hormone receptor activity	2/133	21/18496	0.00985486	0.0345614	0.02206202	IGF1R/INSR	2
MF	GO:0009055	electron transfer activity	4/133	117/18496	0.0101383	0.03530681	0.02253785	CYCS/CYP19A1/NOX4/AKR1B1	4
MF	GO:0033691	sialic acid binding	2/133	22/18496	0.0107897	0.03731437	0.02381936	ADIPOQ/SELE	2
MF	GO:0017134	fibroblast growth factor binding	2/133	23/18496	0.01176211	0.04039675	0.02578698	SCN5A/ITGB3	2
MF	GO:0002039	p53 binding	3/133	66/18496	0.01196259	0.04080391	0.02604688	TP53/GSK3B/NUAK1	3
MF	GO:0016829	lyase activity	5/133	196/18496	0.01371236	0.04618139	0.02947956	CA2/GLO1/CA12/CYP1B1/CA9	5
MF	GO:0017025	TBP-class protein binding	2/133	25/18496	0.01381732	0.04618139	0.02947956	ESR1/AHR	2
MF	GO:0070412	R-SMAD binding	2/133	25/18496	0.01381732	0.04618139	0.02947956	FOS/PARP1	2
MF	GO:0043621	protein self-association	3/133	70/18496	0.01401698	0.04653637	0.02970616	TP53/FLT3/ACHE	3
MF	GO:1901681	sulfur compound binding	6/133	275/18496	0.01461596	0.04756217	0.03036097	APP/CXCL11/CXCL10/F2/MPO/ALK	6
MF	GO:0001637	G protein-coupled chemoattractant receptor activity	2/133	26/18496	0.01489899	0.04756217	0.03036097	CXCR1/GPR35	2
MF	GO:0004950	chemokine receptor activity	2/133	26/18496	0.01489899	0.04756217	0.03036097	CXCR1/GPR35	2
MF	GO:0008395	steroid hydroxylase activity	2/133	26/18496	0.01489899	0.04756217	0.03036097	CYP19A1/CYP1B1	2
MF	GO:0052742	phosphatidylinositol kinase activity	2/133	26/18496	0.01489899	0.04756217	0.03036097	PIK3R1/PIK3CG	2
MF	GO:0070330	aromatase activity	2/133	26/18496	0.01489899	0.04756217	0.03036097	CYP19A1/CYP1B1	2
MF	GO:0044325	transmembrane transporter binding	4/133	133/18496	0.01561186	0.04952044	0.03161102	SCN5A/HSP90AA1/CAV1/SRC	4
MF	GO:0046875	ephrin receptor binding	2/133	27/18496	0.01601595	0.05048065	0.03222396	SRC/PIK3CG	2
MF	GO:0016247	channel regulator activity	4/133	139/18496	0.01806306	0.05657486	0.03611415	BCL2/CAV1/AKT1/CFTR	4
MF	GO:0001965	G-protein alpha-subunit binding	2/133	29/18496	0.01835351	0.05677048	0.03623903	DRD2/IGF1R	2
MF	GO:0001968	fibronectin binding	2/133	29/18496	0.01835351	0.05677048	0.03623903	ITGB3/MMP2	2
MF	GO:0033293	monocarboxylic acid binding	3/133	79/18496	0.01932993	0.0590571	0.03769868	RXRA/PLA2G1B/AKR1C3	3
MF	GO:0046332	SMAD binding	3/133	79/18496	0.01932993	0.0590571	0.03769868	FOS/COL3A1/PARP1	3
MF	GO:0030971	receptor tyrosine kinase binding	3/133	81/18496	0.02064164	0.0624769	0.03988168	TP53/PIK3R1/PTPN11	3
MF	GO:0019955	cytokine binding	4/133	145/18496	0.02074134	0.0624769	0.03988168	IL6R/IL2RA/ITGB3/CXCR1	4
MF	GO:0005164	tumor necrosis factor receptor binding	2/133	31/18496	0.02082563	0.0624769	0.03988168	TNF/STAT1	2

MF	GO:0016209	antioxidant activity	3/133	83/18496	0.02200121	0.0656084	0.04188065	MPO/PTGS1/PTGS2	3
MF	GO:0019956	chemokine binding	2/133	33/18496	0.02342805	0.0690365	0.04406895	ITGB3/CXCR1	2
MF	GO:0030296	protein tyrosine kinase activator activity	2/133	33/18496	0.02342805	0.0690365	0.04406895	EGFR/ALK	2
MF	GO:0008200	ion channel inhibitor activity	2/133	34/18496	0.0247768	0.0724109	0.04622298	CAV1/CFTR	2
MF	GO:0016651	oxidoreductase activity, acting on NAD(P)H	3/133	87/18496	0.02486398	0.0724109	0.04622298	NOX4/AKR1C3/AKR1A1	3
MF	GO:0090482	vitamin transmembrane transporter activity	2/133	35/18496	0.02615657	0.0757324	0.04834323	ABCG2/ABCC1	2
