# Supplementary material for: An integrated network pharmacology and proteomics approach reveals the anti-fibrotic effect of Fushen Granule on peritoneal fibrosis
Source: BMC Complement Med Ther. 2026 Mar 9;26:143. doi: 10.1186/s12906-026-05333-2 (PMC13085474; doi:10.1186/s12906-026-05333-2)
Supplement: Supplementary file 7 — Supplementary Material 7. [file 12906_2026_5333_MOESM7_ESM.pdf]

Article title: An Integrated Network Pharmacology and Proteomics Approach Reveals the Anti-fibrotic Effect of Fushen Granule on Peritoneal Fibrosis

Author names: Kang Yang, Jie Li, Lin Wang, Hangxing Yu, Xinyue Liu, Zhiqing Gao, Zheng Wang, Linqi Zhang, Hongtao Yang
Affiliation and e-mail address of the corresponding author: First Teaching Hospital of Tianjin University of Traditional Chinese Medicine, tjtcmht@126.com

KEGG enrichment analysis of the 133 overlapped genes

ID	Descriptio	GeneRatio	BgRatio	pvalue	p.adjust	qvalue	geneID	Count
hsa04151	PI3K-Akt s	32/126	359/8673	3.43E-17	2.78E-15	1.18E-15	RXRA/HSP	32
hsa05160	Hepatitis C	24/126	158/8673	2.51E-18	6.09E-16	2.59E-16	RXRA/REL	24
hsa05417	Lipid and a	23/126	215/8673	3.57E-14	9.64E-13	4.09E-13	RXRA/HSP	23
hsa05163	Human cy	23/126	225/8673	9.56E-14	2.11E-12	8.96E-13	RELA/BAX	23
hsa05167	Kaposi sar	22/126	194/8673	4.05E-14	9.84E-13	4.18E-13	RELA/FOS	22
hsa05165	Human pa	22/126	331/8673	1.72E-09	1.50E-08	6.35E-09	RELA/BAX	22
hsa05205	Proteoglyc	21/126	203/8673	1.01E-12	1.67E-11	7.11E-12	TP53/CDK	21
hsa05207	Chemical c	21/126	212/8673	2.38E-12	3.61E-11	1.53E-11	RXRA/HSP	21
hsa05162	Measles	20/126	138/8673	5.51E-15	1.67E-13	7.10E-14	RELA/BCL2	20
hsa05161	Hepatitis B	20/126	162/8673	1.28E-13	2.59E-12	1.10E-12	RELA/BCL2	20
hsa05169	Epstein-Ba	20/126	202/8673	8.43E-12	1.02E-10	4.35E-11	RELA/BCL2	20
hsa05222	Small cell l	19/126	92/8673	2.84E-17	2.78E-15	1.18E-15	RXRA/REL	19
hsa01522	Endocrine	19/126	98/8673	1.00E-16	6.09E-15	2.58E-15	BCL2/FOS	19
hsa04933	AGE-RAGE	19/126	100/8673	1.49E-16	7.26E-15	3.08E-15	RELA/BCL2	19
hsa05206	MicroRNA	19/126	310/8673	9.25E-08	4.89E-07	2.07E-07	BCL2/TP53	19
hsa05215	Prostate ca	18/126	97/8673	1.55E-15	6.27E-14	2.66E-14	HSP90AA1	18
hsa05418	Fluid shear	18/126	139/8673	1.03E-12	1.67E-11	7.11E-12	HSP90AA1	18
hsa05166	Human T-	18/126	222/8673	2.71E-09	2.12E-08	9.01E-09	RELA/FOS	18
hsa04510	Focal adhe	17/126	202/8673	4.42E-09	3.16E-08	1.34E-08	BCL2/BAD	17
hsa05223	Non-small	16/126	72/8673	3.10E-15	1.08E-13	4.56E-14	RXRA/BAX	16
hsa04210	Apoptosis	16/126	136/8673	9.07E-11	8.81E-10	3.74E-10	RELA/BCL2	16
hsa05224	Breast can	16/126	147/8673	2.96E-10	2.76E-09	1.17E-09	FOS/BAX/T	16
hsa05226	Gastric car	16/126	149/8673	3.63E-10	3.26E-09	1.39E-09	RXRA/BCL	16
hsa04630	JAK-STAT	16/126	166/8673	1.82E-09	1.52E-08	6.46E-09	BCL2/CDK	16
hsa05225	Hepatocel	16/126	168/8673	2.17E-09	1.75E-08	7.45E-09	BAX/TP53	16
hsa04062	Chemokin	16/126	192/8673	1.51E-08	9.39E-08	3.98E-08	RELA/BAD	16
hsa05203	Viral carcir	16/126	204/8673	3.57E-08	2.13E-07	9.06E-08	RELA/BAX	16
hsa05208	Chemical c	16/126	223/8673	1.24E-07	6.42E-07	2.72E-07	RELA/FOS	16
hsa05171	Coronaviru	16/126	233/8673	2.27E-07	1.10E-06	4.69E-07	RELA/FOS	16
hsa04668	TNF signal	15/126	114/8673	7.23E-11	7.32E-10	3.11E-10	RELA/FOS	15
hsa05212	Pancreatic	14/126	76/8673	2.88E-12	3.89E-11	1.65E-11	RELA/BAX	14
hsa05220	Chronic m	14/126	76/8673	2.88E-12	3.89E-11	1.65E-11	RELA/BAX	14
hsa01521	EGFR tyros	14/126	79/8673	5.03E-12	6.43E-11	2.73E-11	BCL2/BAX	14
hsa05210	Colorectal	14/126	86/8673	1.68E-11	1.95E-10	8.27E-11	BCL2/FOS	14
hsa04657	IL-17 signa	14/126	94/8673	5.87E-11	6.20E-10	2.63E-10	HSP90AA1	14
hsa04926	Relaxin sig	14/126	129/8673	4.28E-09	3.15E-08	1.34E-08	RELA/FOS	14
hsa05202	Transcripti	14/126	193/8673	7.07E-07	2.96E-06	1.26E-06	RXRA/REL	14
hsa05218	Melanoma	13/126	72/8673	2.36E-11	2.60E-10	1.10E-10	BAX/TP53	13
hsa04932	Non-alcoh	13/126	155/8673	3.43E-07	1.60E-06	6.81E-07	RXRA/REL	13
hsa04218	Cellular se	13/126	156/8673	3.70E-07	1.70E-06	7.20E-07	RELA/TP53	13
hsa05164	Influenza A	13/126	171/8673	1.06E-06	4.38E-06	1.86E-06	PRSS1/REL	13
hsa05170	Human im	13/126	212/8673	1.15E-05	4.00E-05	1.70E-05	RELA/BCL2	13
hsa04014	Ras signali	13/126	236/8673	3.60E-05	0.000115	4.89E-05	RELA/BAD	13

hsa05131	Shigellosis	13/126	247/8673	5.78E-05	0.000176	7.49E-05	RELA/BCL2	13
hsa04010	MAPK sigr	13/126	299/8673	0.000387	0.000969	0.000411	RELA/FOS/	13
hsa05010	Alzheimer	13/126	384/8673	0.003738	0.007831	0.003325	RELA/CYC	13
hsa05219	Bladder ca	12/126	41/8673	2.87E-13	5.37E-12	2.28E-12	TP53/CDK	12
hsa04066	HIF-1 sign	12/126	108/8673	4.47E-08	2.52E-07	1.07E-07	RELA/BCL2	12
hsa04659	Th17 cell c	12/126	108/8673	4.47E-08	2.52E-07	1.07E-07	RXRA/HSP	12
hsa05145	Toxoplasm	12/126	111/8673	6.09E-08	3.29E-07	1.40E-07	RELA/BCL2	12
hsa04919	Thyroid hc	12/126	121/8673	1.60E-07	8.09E-07	3.44E-07	RXRA/TP5	12
hsa04068	FoxO signa	12/126	131/8673	3.83E-07	1.72E-06	7.32E-07	CCNB1/CD	12
hsa04915	Estrogen s	12/126	137/8673	6.24E-07	2.71E-06	1.15E-06	HSP90AA1	12
hsa04110	Cell cycle	12/126	157/8673	2.67E-06	1.06E-05	4.52E-06	TP53/CDK	12
hsa05415	Diabetic ca	12/126	203/8673	3.67E-05	0.000116	4.91E-05	RELA/SLC2	12
hsa04024	cAMP sign	12/126	225/8673	9.94E-05	0.000284	0.000121	RELA/FOS/	12
hsa05022	Pathways (	12/126	476/8673	0.043506	0.074979	0.03183	RELA/BCL2	12
hsa04917	Prolactin s	11/126	70/8673	4.10E-09	3.12E-08	1.32E-08	RELA/FOS/	11
hsa01524	Platinum c	11/126	73/8673	6.51E-09	4.52E-08	1.92E-08	BCL2/BAX/	11
hsa04115	p53 signal	11/126	75/8673	8.74E-09	5.59E-08	2.37E-08	BCL2/BAX/	11
hsa05214	Glioma	11/126	75/8673	8.74E-09	5.59E-08	2.37E-08	BAX/TP53/	11
hsa05235	PD-L1 exp	11/126	89/8673	5.49E-08	3.03E-07	1.29E-07	RELA/FOS/	11
hsa04625	C-type lec	11/126	104/8673	2.79E-07	1.33E-06	5.64E-07	RELA/TNF/	11
hsa04931	Insulin resi	11/126	108/8673	4.11E-07	1.81E-06	7.70E-07	RELA/TNF/	11
hsa05135	Yersinia in	11/126	137/8673	4.42E-06	1.68E-05	7.12E-06	RELA/FOS/	11
hsa05152	Tuberculos	11/126	180/8673	5.81E-05	0.000176	7.49E-05	RELA/BCL2	11
hsa04015	Rap1 signa	11/126	210/8673	0.000229	0.000599	0.000254	DRD2/ITGI	11
hsa05132	Salmonella	11/126	247/8673	0.000905	0.002074	0.000881	HSP90AA1	11
hsa05213	Endometri	10/126	58/8673	8.46E-09	5.59E-08	2.37E-08	BAX/TP53/	10
hsa05221	Acute mye	10/126	67/8673	3.60E-08	2.13E-07	9.06E-08	RELA/BAD	10
hsa04064	NF-kappa	10/126	105/8673	2.63E-06	1.06E-05	4.52E-06	RELA/BCL2	10
hsa04620	Toll-like re	10/126	108/8673	3.40E-06	1.33E-05	5.66E-06	RELA/FOS/	10
hsa04722	Neurotrop	10/126	119/8673	8.17E-06	2.96E-05	1.26E-05	RELA/BCL2	10
hsa04071	Sphingolip	10/126	121/8673	9.49E-06	3.34E-05	1.42E-05	RELA/BCL2	10
hsa04660	T cell rece	10/126	121/8673	9.49E-06	3.34E-05	1.42E-05	RELA/FOS/	10
hsa04611	Platelet ac	10/126	124/8673	1.18E-05	4.04E-05	1.71E-05	ITGB3/COI	10
hsa04148	Efferocyto:	10/126	156/8673	8.56E-05	0.000251	0.000106	RXRA/ITGE	10
hsa05130	Pathogeni	10/126	198/8673	0.000593	0.001398	0.000594	RELA/FOS/	10
hsa04060	Cytokine-(	10/126	297/8673	0.011081	0.021542	0.009145	TNF/IL6R/I	10
hsa04920	Adipocyto	9/126	70/8673	6.67E-07	2.84E-06	1.21E-06	RXRA/REL/	9
hsa04012	ErbB signa	9/126	85/8673	3.52E-06	1.36E-05	5.76E-06	BAD/CDKN	9
hsa04061	Viral prote	9/126	100/8673	1.35E-05	4.50E-05	1.91E-05	TNF/IL6R/I	9
hsa04152	AMPK sigr	9/126	121/8673	6.22E-05	0.000187	7.92E-05	ADIPOQ/C	9
hsa04380	Osteoclast	9/126	135/8673	0.000145	0.000396	0.000168	RELA/FOS/	9
hsa04936	Alcoholic l	9/126	142/8673	0.000213	0.000563	0.000239	RELA/ADIF	9
hsa04072	Phospholi	9/126	148/8673	0.00029	0.000751	0.000319	SYK/EGFR/	9
hsa04934	Cushing sy	9/126	155/8673	0.000409	0.001014	0.00043	CDK2/CDK	9
hsa04217	Necroptos	9/126	159/8673	0.000493	0.001198	0.000509	HSP90AA1	9
hsa04621	NOD-like	9/126	186/8673	0.001508	0.003392	0.00144	HSP90AA1	9
hsa05120	Epithelial (	8/126	70/8673	7.05E-06	2.64E-05	1.12E-05	RELA/NFKI	8
hsa04211	Longevity	8/126	89/8673	4.19E-05	0.00013	5.54E-05	RELA/BAX/	8
hsa04914	Progester(	8/126	102/8673	0.000111	0.000307	0.00013	HSP90AA1	8

hsa05142	Chagas dis	8/126	102/8673	0.000111	0.000307	0.00013	RELA/FOS/	8
hsa05146	Amoebiasi	8/126	102/8673	0.000111	0.000307	0.00013	RELA/TNF/	8
hsa05020	Prion disea	8/126	272/8673	0.044287	0.075787	0.032173	BAX/CYCS	8
hsa01523	Antifolate	7/126	30/8673	1.78E-07	8.84E-07	3.75E-07	RELA/TNF/	7
hsa04913	Ovarian st	7/126	51/8673	7.87E-06	2.90E-05	1.23E-05	CYP19A1/	7
hsa04370	VEGF signa	7/126	59/8673	2.11E-05	6.92E-05	2.94E-05	BAD/PIK3F	7
hsa00590	Arachidon	7/126	61/8673	2.63E-05	8.52E-05	3.62E-05	ALOX5/AL	7
hsa05230	Central ca	7/126	70/8673	6.48E-05	0.000192	8.16E-05	TP53/MYC	7
hsa04662	B cell rece	7/126	84/8673	0.000207	0.000553	0.000235	RELA/FOS/	7
hsa04658	Th1 and Tl	7/126	92/8673	0.000364	0.000921	0.000391	RELA/FOS/	7
hsa04928	Parathyroi	7/126	106/8673	0.000856	0.001982	0.000841	RXRA/BCL	7
hsa04935	Growth hc	7/126	120/8673	0.001769	0.003907	0.001659	FOS/STAT	7
hsa04921	Oxytocin s	7/126	154/8673	0.007044	0.013917	0.005908	FOS/CDKN	7
hsa04613	Neutrophi	7/126	191/8673	0.021105	0.037989	0.016127	RELA/ITGB	7
hsa04810	Regulatior	7/126	229/8673	0.049055	0.082342	0.034956	ITGB3/EGF	7
hsa05216	Thyroid ca	6/126	37/8673	1.34E-05	4.50E-05	1.91E-05	RXRA/BAX	6
hsa04923	Regulatior	6/126	58/8673	0.000182	0.000492	0.000209	ADORA1/	6
hsa05321	Inflammat	6/126	65/8673	0.000342	0.000875	0.000371	RELA/TNF/	6
hsa05211	Renal cell	6/126	69/8673	0.000473	0.001162	0.000493	BAD/CDKN	6
hsa05140	Leishmani	6/126	77/8673	0.000852	0.001982	0.000841	RELA/FOS/	6
hsa04976	Bile secreti	6/126	89/8673	0.001813	0.003969	0.001685	RXRA/ABC	6
hsa04520	Adherens	6/126	93/8673	0.002269	0.004879	0.002071	IGF1R/EGF	6
hsa05323	Rheumato	6/126	93/8673	0.002269	0.004879	0.002071	FOS/TNF/	6
hsa04640	Hematopc	6/126	99/8673	0.003108	0.006568	0.002788	TNF/IL6R/	6
hsa04725	Cholinergi	6/126	113/8673	0.005948	0.012045	0.005114	BCL2/FOS/	6
hsa04670	Leukocyte	6/126	115/8673	0.006471	0.01289	0.005472	CLDN4/MI	6
hsa04726	Serotoner	6/126	115/8673	0.006471	0.01289	0.005472	APP/ALOX	6
hsa04114	Oocyte me	6/126	131/8673	0.011926	0.022818	0.009687	AR/CDK1/	6
hsa04910	Insulin sigr	6/126	137/8673	0.014625	0.027128	0.011516	BAD/SLC2	6
hsa04550	Signaling	6/126	143/8673	0.017724	0.032382	0.013747	MYC/STAT	6
hsa04150	mTOR sigr	6/126	156/8673	0.025926	0.045986	0.019522	TNF/GSK3	6
hsa04140	Autophag	6/126	165/8673	0.03289	0.057916	0.024586	BCL2/BAD	6
hsa04022	cGMP-PK	6/126	166/8673	0.033732	0.05855	0.024855	BAD/EDNF	6
hsa04360	Axon guid	6/126	182/8673	0.049134	0.082342	0.034956	GSK3B/PIK	6
hsa04215	Apoptosis	5/126	32/8673	8.79E-05	0.000254	0.000108	BCL2/BAX/	5
hsa04930	Type II dia	5/126	46/8673	0.000508	0.001223	0.000519	ADIPOQ/T	5
hsa05134	Legionello	5/126	56/8673	0.001262	0.002867	0.001217	RELA/CYC	5
hsa04664	Fc epsilon	5/126	68/8673	0.003	0.006394	0.002714	TNF/ALOX	5
hsa05100	Bacterial ir	5/126	77/8673	0.005127	0.010559	0.004482	CAV1/PIK3	5
hsa04540	Gap juncti	5/126	88/8673	0.008963	0.017565	0.007457	CDK1/DRD	5
hsa00790	Folate bios	4/126	27/8673	0.000575	0.001371	0.000582	AKR1B1/A	4
hsa02010	ABC transp	4/126	45/8673	0.003994	0.008295	0.003521	ABCG2/AE	4
hsa05144	Malaria	4/126	50/8673	0.005839	0.011924	0.005062	TNF/SELE/	4
hsa04213	Longevity	4/126	61/8673	0.011714	0.02259	0.00959	IGF1R/PIK3	4
hsa00140	Steroid ho	4/126	62/8673	0.012384	0.02351	0.00998	CYP19A1/	4
hsa05217	Basal cell	4/126	63/8673	0.013078	0.024635	0.010458	BAX/TP53/	4
hsa04929	GnRH secr	4/126	64/8673	0.013797	0.025789	0.010948	SPP1/PIK3	4
hsa04622	RIG-I-like	4/126	72/8673	0.020455	0.037094	0.015747	RELA/TNF/	4
hsa03320	PPAR sign	4/126	75/8673	0.023383	0.041779	0.017736	RXRA/ADI	4

hsa00910	Nitrogen r	3/126	17/8673	0.001754	0.003907	0.001659	CA2/CA12	3
hsa00040	Pentose ar	3/126	36/8673	0.015077	0.027756	0.011783	AKR1B1/A	3
hsa04340	Hedgehoç	3/126	56/8673	0.047551	0.080803	0.034303	BCL2/CCN	3
hsa00670	One carbc	2/126	20/8673	0.033542	0.05855	0.024855	TYMS/DHI	2
