# Supplementary material for: An integrated network pharmacology and proteomics approach reveals the anti-fibrotic effect of Fushen Granule on peritoneal fibrosis
Source: BMC Complement Med Ther. 2026 Mar 9;26:143. doi: 10.1186/s12906-026-05333-2 (PMC13085474; doi:10.1186/s12906-026-05333-2)
Supplement: Supplementary file 8 — Supplementary Material 8. [file 12906_2026_5333_MOESM8_ESM.pdf]

Article title: An Integrated Network Pharmacology and Proteomics Approach Reveals the Anti-fibrotic Effect of Fushen Granule on Peritoneal Fibrosis

Author names: Kang Yang, Jie Li, Lin Wang, Hangxing Yu, Xinyue Liu, Zhiqing Gao, Zheng Wang, Linqi Zhang, Hongtao Yang
Affiliation and e-mail address of the corresponding author: First Teaching Hospital of Tianjin University of Traditional Chinese Medicine, tjtcmht@126.com

303 DEPs was identified between control and model groups										
Protein accession	Protein description	Gene name	Sequence coverage [%]	Mol. weight [kDa]	Score	MS/MS Counts	Peptides	Unique peptides	Model_pro/Control_pro Ratio	Model_pro/Control_pro P value
P78504	Protein jagg	JAG1	30	133.8	143.42	76	22	20	48.199	4.00E-03
Q15582	Transformi	TGFBI	38.5	74.68	71.174	79	18	18	14.322	1.22E-06
Q8NEY1	Neuron nav	NAV1	10.1	202.47	26.482	26	10	10	4.829	4.36E-07
Q99541	Perilipin-2 (	PLIN2	59.3	48.075	142.19	56	17	17	4.724	1.06E-02
P29279	Connective	CTGF	27.5	38.091	31.212	37	6	6	3.894	1.79E-04
Q01196	Runt-relate	RUNX1	17.9	48.736	15.501	29	5	5	3.452	1.57E-05
P20908	Collagen al	COL5A1	12.7	183.56	162.99	69	14	14	3.307	1.79E-06
P05106	Integrin bet	ITGB3	30.8	87.057	58.8	78	18	18	3.024	1.29E-09
P11169	Solute carri	SLC2A3	11.9	53.924	18.124	22	5	5	2.999	1.66E-05
Q14534	Squalene m	SQLE	24.4	63.922	42.4	45	8	8	2.879	3.02E-04
P02751	Fibronectin	FN1	64.7	262.62	323.31	926	122	119	2.715	4.34E-06
Q9NZU5	LIM and cy	LMCD1	71.5	40.832	258.51	166	25	25	2.667	3.08E-08
Q13643	Four and a l	FHL3	46.8	31.192	89.637	48	8	8	2.644	2.45E-07
Q9HBL0	Tensin-1 OS	TNS1	35	185.7	60.59	131	34	32	2.636	1.63E-05
Q15800	Methylstero	MSMO1	14.3	35.215	10.241	9	3	3	2.561	3.41E-04
P98095	Fibulin-2 O	FBLN2	36.1	126.57	234.71	170	26	26	2.478	2.75E-06
P53814	Smoothelin	SMTN	30.3	99.058	70.136	66	15	15	2.465	3.80E-04
Q9Y625	Glypican-6	GPC6	24.3	62.735	35.058	66	8	8	2.444	4.28E-06
P02462	Collagen al	COL4A1	5.7	160.61	85.813	56	7	7	2.374	3.94E-05
P09486	SPARC OS	SPARC	56.4	34.632	82.723	59	11	11	2.359	1.08E-07
P01137	Transformi	TGFB1	23.1	44.341	10.18	22	4	4	2.314	2.80E-03
P26232	Catenin alp	CTNNA2	14.7	105.31	69.319	15	10	2	2.308	1.72E-03
P01130	Low-density	LDLR	29	95.375	50.315	66	16	15	2.246	2.03E-05
Q9GZU1	Mucolipin-1	MCOLN1	7.6	65.022	11.026	11	3	3	2.24	1.32E-02
P06756	Integrin alp	ITGAV	36.7	116.04	105.72	141	28	28	2.181	7.23E-07
P55287	Cadherin-11	CDH11	16.3	87.964	19.773	32	8	8	2.173	3.59E-05
P41221	Protein Wnt	WNT5A	19.5	42.339	23.662	33	6	5	2.162	4.96E-03
Q96K49	Transmemb	TMEM87B	10.1	63.535	10.293	27	4	4	2.145	7.77E-04

P08648	Integrin alp	ITGA5	25.8	114.54	311.58	127	17	17	2.131	1.98E-06
O95070	Protein YIF	YIF1A	20.1	32.011	11.363	23	5	5	2.082	1.85E-04
Q07954	Prolow-den	LRP1	17.9	504.6	143.67	195	53	50	2.063	1.64E-06
P84243	Histone H3.	H3F3A	60.3	15.328	230.14	57	10	1	2.042	7.39E-04
Q6PCE3	Glucose 1,6	PGM2L1	10.1	70.441	19.067	24	4	4	2.027	2.16E-04
P07204	Thrombomo	THBD	51	60.329	229.34	88	14	14	2.025	3.90E-05
Q8N8Z6	Discoidin, (	DCBLD1	13.6	77.92	11.465	18	5	5	2.016	1.98E-02
Q03405	Urokinase p	PLAUR	30.1	36.978	17.812	33	6	6	2.003	4.81E-04
O15254	Peroxisoma	ACOX3	31.9	77.628	47.656	74	14	14	1.963	3.73E-06
Q9UBG0	C-type man	MRC2	14.8	166.67	39.988	65	13	13	1.938	2.77E-04
O15460	Prolyl 4-hyd	P4HA2	48.2	60.901	323.31	173	21	21	1.924	9.90E-08
Q15118	[Pyruvate d	PDK1	26.6	49.244	7.2587	18	8	7	1.905	1.12E-03
Q99715	Collagen alp	COL12A1	21.7	333.14	112.34	147	43	43	1.903	1.86E-05
Q99878	Histone H2.	HIST1H2A	35.9	13.936	195.07	27	5	2	1.902	1.59E-04
Q9BUN8	Derlin-1 OS	DERL1	18.7	28.8	9.6751	27	4	4	1.898	5.00E-04
Q96EM0	Trans-3-hyd	L3HYPDH	18.9	38.137	4.4796	7	4	4	1.89	1.26E-02
P08572	Collagen alp	COL4A2	13.8	167.55	120.43	96	15	15	1.865	1.85E-06
Q6ZSZ5	Rho guanine	ARHGEF18	18.3	151.64	45.614	71	14	14	1.865	7.67E-05
Q969M3	Protein YIP	YIPF5	8.2	27.989	4.8933	17	2	2	1.865	2.00E-04
P61916	NPC intrace	NPC2	45	16.57	119.56	51	5	5	1.84	3.53E-05
O00622	Protein CYI	CYR61	46.5	42.026	96.159	64	13	13	1.822	6.03E-04
P37268	Squalene sy	FDFT1	40.3	48.115	65.569	75	12	12	1.82	1.80E-05
Q9C0E8	Endoplasmi	LNPK	16.8	47.739	12.225	12	4	4	1.813	4.70E-02
Q8N5W9	Refilin-B O	RFLNB	40.2	22.882	8.3883	15	5	5	1.812	2.97E-02
P23469	Receptor-ty	PTPRE	31	80.641	33.318	73	14	13	1.81	8.05E-07
P15151	Poliovirus r	PVR	13.2	45.302	34.778	30	4	4	1.808	8.03E-04
O94855	Protein tran	SEC24D	23.7	113.01	49.262	85	16	15	1.774	1.03E-04
P35555	Fibrillin-1 (	FBN1	3.3	312.3	6.8925	13	6	6	1.755	1.14E-02
P11166	Solute carri	SLC2A1	14	54.083	99.629	58	8	8	1.741	1.05E-06
O94925	Glutaminase	GLS	38.4	73.46	215.67	121	18	18	1.736	8.27E-07
O14531	Dihydropyr	DPYSL4	32.2	61.877	26.84	16	8	8	1.716	1.71E-02
P61254	60S ribosom	RPL26	46.9	17.258	50.382	34	8	2	1.693	1.58E-03
Q9Y6R4	Mitogen-act	MAP3K4	3.2	181.68	1.4096	5	4	4	1.688	3.40E-02
Q99538	Legumain (	LGMN	16.6	49.411	57.354	27	4	4	1.687	2.10E-03
Q16270	Insulin-like	IGFBP7	47.2	29.13	35.17	44	11	11	1.681	2.56E-03

P30825	High affinit	SLC7A1	8.4	67.638	15.623	35	5	4	1.68	2.17E-04
Q9Y2H1	Serine/threo	STK38L	12.5	54.002	8.8012	20	4	4	1.676	8.16E-05
Q01650	Large neutra	SLC7A5	9.3	55.01	124.9	22	3	3	1.674	9.96E-05
P09972	Fructose-bis	ALDOC	70.1	39.455	278.16	128	18	14	1.663	7.59E-05
Q9UDY4	DnaJ homol	DNAJB4	33.2	37.806	17.419	29	8	7	1.661	1.52E-03
Q86UU1	Pleckstrin h	PHLDB1	26.3	151.16	140.53	138	21	21	1.649	1.92E-03
P61966	AP-1 compl	AP1S1	27.2	18.733	38.619	15	3	3	1.634	1.55E-04
Q8WWX9	Selenoprote	SELENOM	30.3	16.231	7.3474	27	3	3	1.627	3.17E-04
Q9UBM7	7-dehydrocl	DHCR7	9.7	54.489	21.741	26	3	3	1.618	8.64E-03
O00469	Procollagen	PLOD2	43.4	84.685	233.89	170	26	26	1.61	5.78E-05
Q6VY07	Phosphofur	PACS1	20.4	104.9	16.891	42	11	10	1.608	2.44E-04
O76054	SEC14-like	SEC14L2	29.5	46.145	37.437	56	7	7	1.6	3.58E-04
Q8IZQ5	Selenoprote	SELENOH	39.3	13.453	5.6869	17	3	3	1.599	5.60E-04
P13807	Glycogen [s	GYS1	45.7	83.785	155.22	122	23	23	1.591	1.65E-05
Q9Y6M7	Sodium bica	SLC4A7	7.2	136.04	13.636	28	5	5	1.589	2.09E-05
P21980	Protein-glut	TGM2	64.5	77.328	323.31	250	34	34	1.587	3.63E-04
Q9NRY6	Phospholipi	PLSCR3	16.9	31.648	22.885	19	3	3	1.58	4.21E-05
P13674	Prolyl 4-hyd	P4HA1	55.4	61.049	232.24	251	28	28	1.578	6.71E-07
P62805	Histone H4	HIST1H4A	65	11.367	180.06	141	17	17	1.569	1.77E-05
Q04726	Transducin-	TLE3	37.6	83.416	53.991	80	17	13	1.555	7.74E-05
P46778	60S ribosom	RPL21	33.1	18.565	71.295	19	6	6	1.538	3.42E-04
Q5VTL8	Pre-mRNA-	PRPF38B	19.6	64.467	7.6539	19	6	6	1.535	1.11E-02
Q7Z3C6	Autophagy-	ATG9A	31.1	94.446	52.336	118	18	18	1.534	1.08E-03
O14613	Cdc42 effec	CDC42EP2	31.4	22.483	17.829	23	3	3	1.533	7.14E-03
Q5T0N5	Formin-binc	FNBP1L	19.2	70.065	11.948	23	7	7	1.528	3.86E-02
Q02809	Procollagen	PLOD1	46.5	83.549	213.16	197	27	27	1.527	3.36E-06
O60784	Target of M	TOM1	30.1	53.818	28.893	43	10	10	1.526	5.58E-05
Q8N128	Protein FAI	FAM177A1	34.7	23.757	56.019	35	5	5	1.52	4.21E-04
Q8WUH6	Transmemb	TMEM263	56.9	11.748	40.722	24	4	4	1.516	1.65E-05
P13987	CD59 glyco	CD59	18.8	14.177	13.887	18	3	3	1.513	1.52E-02
Q8WUP2	Filamin-bin	FBLIM1	45.8	40.669	107.32	133	18	18	1.512	1.96E-03
P51911	Calponin-1	CNN1	55.2	33.17	125.81	70	11	10	1.511	1.63E-05
P05997	Collagen al	COL5A2	16.3	144.91	183.12	84	14	14	1.506	2.63E-06
Q6NUQ4	Transmemb	TMEM214	28.2	77.15	58.12	96	13	13	1.502	1.13E-07
P62851	40S ribosom	RPS25	24	13.742	6.2516	14	4	4	1.501	2.68E-02

Q9H9Y6	DNA-direct	POLR1B	8.5	128.23	11.561	23	7	7	0.666	1.08E-02
Q5U4P2	Aspartate b	ASPHD1	25.6	41.127	17.062	31	6	6	0.666	2.07E-02
Q9NYL2	Mitogen-ac	MAP3K20	15.2	91.154	25.913	38	8	8	0.665	3.17E-04
Q8TDD1	ATP-depen	DDX54	30.5	98.594	59.827	76	17	17	0.665	2.50E-03
Q6P4E1	Protein CA	CASC4	13.9	48.864	5.3	25	4	4	0.665	8.88E-03
Q9H4H8	Protein FA	FAM83D	20.5	64.424	62.004	36	7	7	0.664	8.39E-04
Q96SB3	Neurabin-2	PPP1R9B	12.7	89.333	8.555	18	7	7	0.664	1.98E-02
P18074	General tra	ERCC2	30	86.908	51.795	73	14	14	0.663	1.23E-04
Q9NRZ9	Lymphoid-s	HELLS	19.7	97.073	34.676	68	13	13	0.662	3.16E-04
A0A0U1RF	Protein MM	MMP24OS	74.6	7.6794	26.297	25	2	2	0.661	6.16E-03
Q9BSV6	tRNA-splic	TSEN34	50	33.652	15.235	28	11	11	0.661	1.02E-02
Q96I51	RCC1-like	RCC1L	16.2	49.996	12.997	27	4	4	0.66	1.34E-02
Q9H3Q1	Cdc42 effec	CDC42EP4	21.6	37.979	20.784	21	4	4	0.659	1.04E-03
Q96RQ3	Methylcrot	MCCC1	22.1	80.472	31.045	38	8	8	0.658	8.41E-05
Q8N0X7	Spartin OS=	SPART	18.3	72.832	29.442	25	7	7	0.657	6.19E-04
Q9UL33	Trafficking	TRAPPC2L	20.7	16.145	6.0271	14	2	2	0.657	1.84E-03
Q9Y2K2	Serine/thre	SIK3	5.8	144.85	73.992	16	4	4	0.657	4.29E-02
Q13325	Interferon-i	IFIT5	25.1	55.846	29.032	35	8	8	0.656	8.80E-04
Q05932	Folylpolygl	FPGS	18.2	64.608	12.416	24	6	6	0.656	6.12E-03
Q8TCZ2	CD99 antig	CD99L2	13	27.986	4.9063	10	2	2	0.656	9.90E-03
Q9NS91	E3 ubiquiti	RAD18	28.9	56.222	32.853	81	11	11	0.655	1.86E-05
Q9UPT9	Ubiquitin c	USP22	23.8	59.96	50.963	38	8	7	0.655	5.89E-05
P40424	Pre-B-cell l	PBX1	24.2	46.625	59.374	28	5	3	0.655	4.00E-04
Q9BV57	1,2-dihydro	ADI1	46.4	21.498	11.028	30	6	6	0.655	4.55E-04
Q9UNS1	Protein tim	TIMELESS	6.2	138.66	7.4502	19	5	5	0.655	3.22E-03
Q7L5Y9	E3 ubiquiti	MAEA	7.1	45.287	1.7413	6	2	2	0.655	2.62E-02
Q9GZY8	Mitochondr	MFF	44.4	38.464	32.484	62	12	12	0.654	1.52E-05
P23025	DNA repair	XPA	17.2	31.368	5.7489	10	3	3	0.653	1.73E-02
Q9UQB8	Brain-speci	BAIAP2	40.4	60.867	45.436	85	14	14	0.652	7.76E-05
Q16512	Serine/thre	PKN1	14.4	103.93	17.08	33	8	8	0.652	2.57E-04
Q12774	Rho guanin	ARHGEF5	3.8	176.8	17.037	15	4	3	0.652	2.58E-03
Q9Y5B8	Nucleoside	NME7	19.1	42.491	11.759	15	4	4	0.652	3.20E-03
P55957	BH3-intera	BID	34.4	21.994	79.322	34	4	4	0.65	3.56E-04
Q9P270	SLAIN mot	SLAIN2	19.6	62.543	21.378	34	7	7	0.65	4.17E-04
A6NKD9	Coiled-coil	CCDC85C	25.3	45.209	17.782	14	7	7	0.65	2.45E-02

Q16763	Ubiquitin-c	UBE2S	53.6	23.845	45.647	31	7	7	0.647	1.59E-04
Q9C0D5	Protein TAI	TANC1	22.6	202.22	55.176	97	25	24	0.646	1.40E-03
Q5VV41	Rho guanin	ARHGEF16	13.8	80.104	12.325	30	6	6	0.645	1.32E-03
Q8TDB4	Protein MG	MGARP	35	25.389	149.9	43	6	6	0.644	9.83E-05
Q6NSJ5	Volume-reg	LRRC8E	11.8	90.246	13.004	21	6	6	0.644	1.15E-02
Q14181	DNA polym	POLA2	29.1	65.947	36.53	64	9	9	0.642	2.62E-04
P35568	Insulin rece	IRS1	15.1	131.59	40.674	36	11	10	0.641	3.06E-03
Q86SF2	N-acetylgal	GALNT7	7.9	75.388	11.309	12	3	3	0.64	9.90E-03
Q9NYP9	Protein Mis	MIS18A	25.8	25.863	30.029	22	3	3	0.639	5.54E-03
Q9HAW4	Claspin OS	CLSPN	8.9	151.09	19.816	34	8	8	0.638	2.30E-03
Q96CS2	HAUS augr	HAUS1	11.5	31.863	5.6137	11	2	2	0.637	1.99E-02
Q9NW81	Distal meml	DMAC2	11.7	29.267	4.9316	18	2	2	0.636	4.39E-02
P50895	Basal cell a	BCAM	54.6	67.404	257.54	191	25	25	0.635	3.64E-05
Q9NUQ3	Gamma-tax	TXLNG	27.3	60.585	16.146	40	9	8	0.635	3.40E-04
Q9BT17	Mitochondr	MTG1	22.2	37.236	15.721	33	5	5	0.635	7.37E-04
Q15785	Mitochondr	TOMM34	51.5	34.559	123.34	65	11	11	0.634	1.83E-04
Q86VP1	Tax1-bindir	TAX1BP1	19.6	90.876	15.321	29	8	8	0.634	2.44E-04
O14777	Kinetochor	NDC80	20.9	73.912	20.399	30	8	8	0.634	5.19E-04
Q9NQE9	Histidine tri	HINT3	37.4	20.361	7.8991	28	4	4	0.632	2.78E-03
Q9BX63	Fanconi ane	BRIP1	14.1	140.87	21.534	44	11	11	0.631	1.83E-04
Q9UL42	Paraneoplas	PNMA2	24.5	41.509	45.036	34	5	5	0.631	3.82E-04
Q5JSZ5	Protein PRI	PRRC2B	7	242.96	14.102	28	8	8	0.631	8.21E-04
Q96RL7	Vacuolar pr	VPS13A	2.4	360.27	4.724	14	5	5	0.631	2.08E-02
Q9BZI7	Regulator o	UPF3B	9.9	57.761	6.078	8	3	3	0.63	2.64E-03
P35080	Profilin-2 C	PFN2	29.3	15.046	10.241	16	3	3	0.628	3.75E-02
Q9ULV3	Cip1-intera	CIZ1	10.4	100.04	20.629	16	6	6	0.626	5.56E-03
Q9NXA8	NAD-deper	SIRT5	20.6	33.881	6.5268	20	4	4	0.624	7.96E-03
P80217	Interferon-i	IFI35	21.7	31.546	13.091	12	3	2	0.624	8.86E-03
P21926	CD9 antige	CD9	20.6	25.416	63.109	18	3	3	0.624	1.25E-02
Q15555	Microtubul	MAPRE2	43.1	37.031	30.061	39	8	6	0.623	1.84E-04
Q5U5X0	Complex III	LYRM7	40.4	11.955	2.9264	16	3	3	0.623	5.96E-04
Q86Y37	CDK2-asso	CACUL1	20.1	41.063	8.9263	20	5	5	0.623	2.98E-03
Q14118	Dystroglyca	DAG1	10.1	97.44	15.592	30	8	8	0.622	1.04E-04
Q5VZ89	DENN dom	DENND4C	3.2	212.71	4.9704	8	4	4	0.622	7.74E-03
P04114	Apolipopro	APOB	1.4	515.6	1.7919	11	5	4	0.621	1.36E-03

O75144	ICOS ligan	ICOSLG	12.9	33.348	5.8593	11	3	3	0.621	2.50E-02
P23497	Nuclear aut	SP100	6.3	100.42	4.9667	18	5	3	0.62	2.63E-04
Q8NG31	Kinetochore	KNL1	3.5	265.39	7.2863	9	5	5	0.619	5.16E-03
Q9NRW3	DNA dC->c	APOBEC3C	23.2	22.826	4.4654	12	3	2	0.617	9.21E-04
P56962	Syntaxin-17	STX17	27.5	33.403	14.536	18	4	4	0.616	3.66E-03
Q9H977	WD repeat-	WDR54	21.6	35.891	10.832	11	3	3	0.615	1.05E-04
Q9Y3Z3	Deoxynucle	SAMHD1	39.1	72.2	48.753	101	17	16	0.614	1.90E-05
Q9H788	SH2 domain	SH2D4A	32.2	52.726	35.357	83	13	13	0.613	1.84E-05
Q96SZ6	CDK5 regu	CDK5RAP1	7.8	67.688	8.4744	16	3	3	0.612	1.84E-03
Q9P2K8	eIF-2-alpha	EIF2AK4	6	186.91	9.768	15	6	6	0.609	1.34E-03
Q8TEU7	Rap guanin	RAPGEF6	2.2	179.42	5.5587	8	2	2	0.609	2.47E-02
Q6PJG2	ELM2 and S	ELMSAN1	10.4	114.99	7.1465	14	5	5	0.609	3.66E-02
O00625	Pirin OS=H	PIR	24.1	32.113	6.8302	12	4	4	0.608	1.02E-03
P30504	HLA class I	HLA-C	41.3	40.994	6.6714	11	11	1	0.608	6.72E-03
Q9NXH8	Torsin-4A (	TOR4A	17	46.914	22.917	29	5	5	0.606	1.97E-04
Q8NI35	InaD-like pr	PATJ	6.2	196.37	11.848	10	6	6	0.605	2.90E-03
Q07617	Sperm-asso	SPAG1	8.6	103.64	8.8391	20	6	6	0.605	7.90E-03
Q6VN20	Ran-binding	RANBP10	13.1	67.256	8.1423	14	5	4	0.605	2.16E-02
P56556	NADH dehy	NDUFA6	41.4	15.136	5.1421	20	4	4	0.604	5.56E-03
A0AV96	RNA-bindin	RBM47	19.2	64.098	14.417	28	7	7	0.601	3.83E-04
Q9Y6J9	TAF6-like I	TAF6L	6.3	67.814	5.3033	11	2	2	0.599	9.54E-03
Q8N8R3	Mitochondr	SLC25A29	23.1	32.062	8.1246	16	4	4	0.599	1.11E-02
Q08431	Lactadherin	MFGE8	28.9	43.104	14.469	32	7	6	0.598	1.48E-03
Q9NUL3	Double-stra	STAU2	19.6	62.608	7.9636	15	7	7	0.594	1.64E-03
Q6PJG6	BRCA1-ass	BRAT1	26.6	88.118	28.956	44	12	12	0.591	7.57E-04
Q8TCY9	Up-regulato	URGCP	11.6	104.99	16.58	35	7	7	0.59	1.10E-03
Q15746	Myosin ligh	MYLK	14.6	210.71	39.955	58	17	17	0.585	1.63E-05
Q92796	Disks large	DLG3	8.4	90.313	7.3799	18	4	4	0.584	2.22E-03
Q9NVH0	Exonucleas	EXD2	15.9	70.352	10.948	22	6	6	0.582	3.40E-03
O75976	Carboxypep	CPD	13.8	152.93	25.651	37	11	11	0.581	6.23E-05
O94788	Retinal deh	ALDH1A2	52.1	56.723	39.286	97	18	16	0.579	2.16E-03
Q96HR8	H/ACA ribo	NAF1	5.3	53.716	4.3831	11	2	2	0.579	8.24E-03
Q08380	Galectin-3-l	LGALS3BP	27.4	65.33	50.054	51	10	10	0.578	1.14E-06
P61457	Pterin-4-alp	PCBD1	51	11.999	7.5114	23	4	4	0.577	8.84E-03
P04818	Thymidylat	TYMS	47.9	35.716	111.52	79	10	10	0.576	1.82E-05

P47895	Aldehyde d	ALDH1A3	72.7	56.108	323.31	233	32	30	0.575	1.80E-06
Q9H467	CUE domai	CUEDC2	9.4	32.009	8.1195	5	2	2	0.575	2.31E-02
P23229	Integrin alp	ITGA6	8	126.6	15.164	18	5	5	0.574	7.62E-03
Q8IZ07	Ankyrin rep	ANKRD13A	26.8	67.618	47.892	42	11	10	0.571	1.02E-04
Q9NVP2	Histone cha	ASF1B	38.6	22.433	12.139	26	4	4	0.57	8.13E-05
Q13625	Apoptosis-s	TP53BP2	10.3	125.61	25.955	24	6	6	0.568	1.11E-02
O60353	Frizzled-6 (	FZD6	4.4	79.291	3.5213	7	2	2	0.567	1.93E-02
P27707	Deoxycytid	DCK	21.5	30.518	5.5929	13	4	4	0.564	6.58E-03
Q13572	Inositol-tetr	ITPK1	24.9	45.621	16.665	31	6	5	0.559	6.59E-04
Q01484	Ankyrin-2 (	ANK2	1.8	433.71	2.2063	6	5	5	0.554	2.13E-02
Q9BQS8	FYVE and (	FYCO1	17.3	166.98	23.163	43	15	15	0.553	9.77E-04
Q9UBB4	Ataxin-10 (	ATXN10	43.8	53.488	53.25	71	14	14	0.55	1.82E-06
P61077	Ubiquitin-c	UBE2D3	42.9	16.687	60.846	24	4	4	0.549	3.08E-03
Q9C010	cAMP-depe	PKIB	53.8	8.4682	6.908	11	3	3	0.547	1.40E-03
Q00978	Interferon r	IRF9	7.6	43.696	6.1917	12	2	2	0.546	2.52E-02
O75843	AP-1 compl	AP1G2	9.6	87.116	7.4127	14	6	6	0.542	2.12E-05
Q8N490	Probable hy	PNKD	17.9	42.875	11.507	16	4	4	0.541	3.22E-03
P43121	Cell surface	MCAM	29.9	71.607	27.925	45	12	11	0.539	1.22E-04
Q99797	Mitochondr	MIPEP	10.8	80.64	3.5398	12	5	5	0.539	2.75E-02
Q96PZ2	Protein FAI	FAM111A	19	70.195	22.121	26	7	7	0.536	3.68E-05
Q14680	Maternal en	MELK	10.3	74.641	9.5369	21	4	4	0.536	7.84E-04
O75911	Short-chain	DHRS3	14.9	33.548	7.793	14	3	3	0.536	1.88E-03
Q9BRQ8	Apoptosis-i	AIFM2	49.1	40.526	34.327	47	10	10	0.535	2.55E-04
Q14764	Major vault	MVP	42.2	99.326	94.59	164	26	26	0.534	1.75E-05
O75330	Hyaluronan	HMMR	8.6	84.099	8.0015	14	5	5	0.533	1.82E-02
Q99959	Plakophilin	PKP2	36.7	97.414	67.685	84	18	18	0.522	4.34E-05
Q5GLZ8	Probable E3	HERC4	24.3	118.56	53.562	54	13	13	0.52	4.13E-05
Q9H4B6	Protein salv	SAV1	19.3	44.633	12.978	21	4	4	0.52	8.37E-04
Q8IYS1	Peptidase M	PM20D2	26.1	47.776	27.185	48	8	8	0.519	5.68E-05
O60566	Mitotic che	BUB1B	17.3	119.54	23.316	44	10	10	0.519	1.28E-02
Q56NI9	N-acetyltra	ESCO2	10	68.306	4.9614	11	4	4	0.518	5.88E-03
Q460N5	Protein mor	PARP14	4.4	202.8	7.4073	6	4	4	0.515	2.08E-02
Q4KMQ1	Taperin OS	TPRN	6	75.555	17.793	10	2	2	0.514	2.66E-03
P00374	Dihydrofola	DHFR	53.5	21.452	12.486	30	7	7	0.513	1.56E-04
Q8IZV5	Retinol deh	RDH10	18.2	38.087	7.535	16	4	4	0.513	2.35E-02

Q03169	Tumor necr	TNFAIP2	24.9	72.66	23.768	42	11	11	0.512	2.01E-04
O95810	Caveolae-as	CAVIN2	18.4	47.173	17.31	26	5	5	0.51	8.20E-04
Q8N392	Rho GTPas	ARHGAP18	9.7	74.976	7.6626	11	4	4	0.51	3.43E-02
Q9H4L7	SWI/SNF-r	SMARCAD	7.3	117.4	7.0756	12	5	5	0.505	2.16E-02
Q96QD8	Sodium-cou	SLC38A2	12.3	56.025	56.772	23	3	3	0.503	1.30E-03
P14923	Junction pla	JUP	40.9	81.744	73.753	80	18	16	0.501	4.92E-07
O00401	Neural Wisl	WASL	19.2	54.826	8.741	16	6	6	0.5	3.16E-03
Q8IV63	Inactive ser	VRK3	10.8	52.881	8.9578	11	3	3	0.5	2.32E-02
Q9C035	Tripartite m	TRIM5	8.7	56.338	15.276	13	3	3	0.496	1.18E-03
P05549	Transcriptic	TFAP2A	11.4	48.062	9.3135	12	3	3	0.494	5.68E-03
P23458	Tyrosine-pr	JAK1	8.8	133.28	6.0214	19	7	7	0.493	1.56E-03
Q7Z3E5	LisH domai	ARMC9	3.5	91.818	10.436	11	2	2	0.493	4.85E-02
Q9H0Z9	RNA-bindir	RBM38	15.1	25.498	6.3	16	2	2	0.492	2.91E-02
Q99650	Oncostatin-	OSMR	15.5	110.51	17.196	23	7	7	0.491	1.31E-02
Q9UKV5	E3 ubiquitir	AMFR	19.9	72.995	34.354	34	5	5	0.49	7.84E-04
O14545	TRAF-type	TRAFD1	13.1	64.84	7.7165	18	5	5	0.489	1.58E-03
Q96AE7	Tetratricope	TTC17	9.9	129.56	9.8271	20	7	7	0.488	9.58E-04
P02686	Myelin basi	MBP	10.2	33.117	2.5103	8	2	2	0.482	3.58E-03
Q13501	Sequestosor	SQSTM1	68.2	47.687	305.58	122	18	18	0.481	9.52E-07
P30530	Tyrosine-pr	AXL	24.8	98.336	40.967	66	15	15	0.478	1.83E-06
P40692	DNA misma	MLH1	15.7	84.6	5.1882	10	7	6	0.478	3.84E-02
O75616	GTPase Era	ERAL1	7.8	48.349	16.247	10	2	2	0.475	1.34E-02
Q96DR7	Rho guanin	ARHGEF26	6.1	97.344	2.7292	7	3	3	0.475	3.12E-02
Q9BXW9	Fanconi ane	FANCD2	8.9	164.13	17.225	30	9	9	0.472	7.81E-04
P17535	Transcriptic	JUND	30.8	35.173	19.827	19	5	5	0.466	2.92E-02
Q6P0N0	Mis18-bind	MIS18BP1	6.7	129.08	7.0672	10	4	4	0.462	2.74E-03
Q8IUC4	Rhophilin-2	RHPN2	10.8	76.992	11.108	10	4	4	0.458	1.44E-02
Q14651	Plastin-1 O	PLS1	28.5	70.253	12.718	20	13	10	0.457	2.21E-04
P08236	Beta-glucur	GUSB	5.1	74.731	5.4682	11	2	2	0.456	4.78E-03
Q8WY36	HMG box t	BBX	6.6	105.13	14.705	20	3	3	0.453	2.08E-02
P33552	Cyclin-depe	CKS2	64.6	9.8602	9.3222	15	4	4	0.451	4.04E-03
O43181	NADH deh	NDUFS4	14.3	20.108	10.349	12	2	2	0.449	7.26E-03
Q9H496	Torsin-1A-i	TOR1AIP2	22.1	15.347	5.1839	6	2	2	0.447	2.30E-03
Q92626	Peroxidasin	PXDN	12	165.27	26.575	35	10	10	0.443	5.84E-03
P31431	Syndecan-4	SDC4	19.2	21.641	5.9325	18	4	4	0.442	1.35E-02

P24593	Insulin-like	IGFBP5	30.1	30.57	9.6908	18	5	5	0.437	1.17E-04
O00213	Amyloid-be	APBB1	11.4	77.243	11.502	19	5	5	0.437	5.82E-04
Q13671	Ras and Ral	RIN1	6.4	84.098	4.0454	15	3	3	0.43	8.06E-05
Q8N573	Oxidation r	OXR1	9	97.969	4.7352	9	5	5	0.429	1.68E-03
Q8NBJ4	Golgi meml	GOLM1	19.5	45.333	12.293	26	6	6	0.427	2.80E-04
Q9GZX9	Twisted gas	TWSG1	6.7	25.017	3.1677	23	1	1	0.427	2.16E-03
Q969F9	Hermansky-	HPS3	8.9	113.73	5.8075	6	5	5	0.409	3.92E-02
Q96HY7	Probable 2-	DHTKD1	3.6	103.08	3.7863	12	2	2	0.402	5.58E-04
Q15004	PCNA-asso	PCLAF	58.6	11.986	24.359	27	6	6	0.397	9.36E-03
P56945	Breast canc	BCAR1	19	93.371	29.115	38	8	8	0.396	2.84E-04
Q12974	Protein tyro	PTP4A2	30.5	19.127	4.79	15	4	2	0.39	1.68E-02
P98082	Disabled ho	DAB2	23.8	82.447	62.546	47	12	12	0.384	1.87E-07
P16144	Integrin bet	ITGB4	28.6	202.16	94.941	126	33	32	0.373	2.87E-06
Q8NFJ5	Retinoic aci	GPRC5A	26.1	40.251	69.967	44	6	5	0.366	9.10E-07
P57076	Cilia- and fl	CFAP298	26.6	33.224	9.5693	12	5	5	0.354	4.19E-04
O94964	Protein SO(	SOGA1	11.1	159.76	8.8302	13	10	8	0.346	1.02E-03
O14879	Interferon-i	IFIT3	17.6	55.984	11.767	18	5	5	0.345	5.55E-04
O14933	Ubiquitin/IS	UBE2L6	66	17.769	30.235	22	5	5	0.334	7.62E-04
Q69YH5	Cell divisio	CDCA2	15.7	112.68	14.773	19	8	8	0.323	2.43E-02
Q8TDB6	E3 ubiquiti	DTX3L	18	83.553	12.323	22	8	8	0.313	9.44E-03
Q99523	Sortilin OS	SORT1	5.4	92.067	6.9483	10	3	3	0.31	1.01E-02
O94955	Rho-related	RHOBTB3	20.9	69.413	38.452	18	8	8	0.303	6.24E-03
P10909	Clusterin O	CLU	15.1	52.494	7.9685	15	4	4	0.292	2.27E-05
Q8WWA1	Transmemb	TMEM40	18.5	25.495	7.798	9	3	3	0.283	9.84E-03
Q13309	S-phase kin	SKP2	20.5	47.76	7.3948	9	5	5	0.251	1.09E-02
Q9NPH2	Inositol-3-p	ISYNA1	24.2	61.067	16.708	24	8	7	0.207	2.03E-05
Q53GL7	Protein mor	PARP10	9.7	110	13.179	12	5	5	0.205	2.84E-04
Q9UKK3	Protein mor	PARP4	4.2	192.59	8.2532	8	4	4	0.198	7.86E-05
O60437	Periplakin (	PPL	11.8	204.74	31.856	31	15	15	0.178	5.95E-08
P12830	Cadherin-1	CDH1	3.6	97.455	5.736	6	2	2	0.092	5.62E-03
