# Supplementary material for: An integrated network pharmacology and proteomics approach reveals the anti-fibrotic effect of Fushen Granule on peritoneal fibrosis
Source: BMC Complement Med Ther. 2026 Mar 9;26:143. doi: 10.1186/s12906-026-05333-2 (PMC13085474; doi:10.1186/s12906-026-05333-2)
Supplement: Supplementary file 9 — Supplementary Material 9. [file 12906_2026_5333_MOESM9_ESM.pdf]

Article title: An Integrated Network Pharmacology and Proteomics Approach Reveals the Anti-fibrotic Effect of Fushen Granule on Peritoneal Fibrosis
Author names: Kang Yang, Jie Li, Lin Wang, Hangxing Yu, Xinyue Liu, Zhiqing Gao, Zheng Wang, Linqi Zhang, Hongtao Yang
Affiliation and e-mail address of the corresponding author: First Teaching Hospital of Tianjin University of Traditional Chinese Medicine, tjtcmht@126.com

386 DEPs were identified in FSG group vs. model group										
**Protein accession**	**Protein descriptio n**	**Gene name**	**Sequence coverage [%]**	**Mol. weight [kDa]**	**Score**	**MS/MS Counts**	**Peptides**	**Unique peptides**	**FSG_pro/Model_p ro Ratio**	**FSG_pro/Model_pr o P value**
Q99541	Perilipin-2 (	PLIN2	59.3	48.075	142.19	56	17	17	14.279	1.03E-02
Q8WVX9	Fatty acyl-C	FAR1	18.3	59.356	8.6918	16	6	6	10.408	5.20E-03
Q9ULX9	Transcriptic	MAFF	22	17.76	6.4083	8	2	2	5.337	3.57E-04
Q9NVN8	Guanine nu	GNL3L	8.1	65.572	4.9379	11	3	3	4.522	1.88E-02
Q9UPY5	Cystine/glut	SLC7A11	5.8	55.422	5.268	11	2	2	4.132	6.24E-04
Q92626	Peroxidasin	PXDN	12	165.27	26.575	35	10	10	4.068	7.97E-04
P24593	Insulin-like	IGFBP5	30.1	30.57	9.6908	18	5	5	3.805	1.60E-04
P17301	Integrin alp	ITGA2	18	129.29	29.001	30	12	12	3.699	1.52E-03
Q8TAA5	GrpE protei	GRPEL2	31.6	25.431	15.002	14	4	4	3.616	1.64E-05
Q5U4P2	Aspartate b	ASPHD1	25.6	41.127	17.062	31	6	6	3.257	4.65E-06
P21580	Tumor necr	TNFAIP3	13.2	89.613	10.858	17	6	6	3.193	1.76E-02
O75326	Semaphorin	SEMA7A	12.5	74.823	7.7905	10	5	5	3.073	4.36E-03
Q8TDD1	ATP-depen	DDX54	30.5	98.594	59.827	76	17	17	3.032	6.02E-05
P09601	Heme oxyg	HMOX1	46.9	32.818	22.716	43	10	9	3.02	5.42E-04
Q9UK39	Nocturnin (	NOCT	18.6	48.195	14.949	14	5	5	2.969	1.10E-02
Q96QD8	Sodium-cou	SLC38A2	12.3	56.025	56.772	23	3	3	2.852	2.35E-04
Q9H116	GDNF-indu	GZF1	6.3	80.491	4.5972	8	3	3	2.79	2.04E-02
Q9Y3A2	Probable U	UTP11	21.3	30.446	14.5	15	5	5	2.707	1.26E-03
O00622	Protein CY	CYR61	46.5	42.026	96.159	64	13	13	2.685	1.02E-06
P07305	Histone H1.	H1F0	23.7	20.863	18.218	23	5	5	2.675	1.18E-03
Q9H9Y2	Ribosome p	RPF1	9.2	40.11	3.7699	6	2	2	2.653	2.08E-02
P56182	Ribosomal	RRP1	23.4	52.839	21.692	35	8	8	2.624	3.32E-03
P56945	Breast canc	BCAR1	19	93.371	29.115	38	8	8	2.559	4.45E-04
Q13501	Sequestoso	SQSTM1	68.2	47.687	305.58	122	18	18	2.528	4.51E-07
P61587	Rho-related	RND3	37.7	27.368	21.344	34	8	7	2.489	1.97E-05
Q6DKI1	60S riboson	RPL7L1	27.8	29.669	12.048	26	6	6	2.486	6.42E-04
Q6NUM9	All-trans-re	RETSAT	13.4	66.819	15.488	34	5	5	2.466	4.51E-02
Q8IZQ5	Selenoprote	SELENOH	39.3	13.453	5.6869	17	3	3	2.453	7.61E-05

P01034	Cystatin-C (	CST3	11.6	15.799	4.1459	13	2	2	2.437	8.28E-03
P30530	Tyrosine-pr	AXL	24.8	98.336	40.967	66	15	15	2.382	5.23E-07
O60427	Acyl-CoA (	FADS1	27.7	51.964	18.461	32	8	7	2.377	1.97E-04
Q9H7B2	Ribosome p	RPF2	30.7	35.582	16.476	33	8	8	2.355	1.41E-06
O95478	Ribosome b	NSA2	20.4	30.065	5.9168	20	4	4	2.325	3.73E-05
Q9UHA3	Probable rit	RSL24D1	18.4	19.621	6.0652	9	2	2	2.304	3.50E-02
O00165	HCLS1-ass	HAX1	32.3	31.62	38.887	26	5	5	2.292	1.58E-04
Q16270	Insulin-like	IGFBP7	47.2	29.13	35.17	44	11	11	2.258	7.56E-07
Q96KM6	Zinc finger	ZNF512B	20.3	97.263	26.74	37	10	10	2.239	1.58E-03
Q9NPH2	Inositol-3-p	ISYNA1	24.2	61.067	16.708	24	8	7	2.21	2.76E-04
Q9NYT0	Pleckstrin-2	PLEK2	20.7	39.97	10.215	9	4	4	2.21	2.94E-02
O43709	Probable 18	BUD23	23.8	31.88	10.653	21	4	4	2.204	1.94E-03
Q9BRS8	La-related p	LARP6	16.1	54.736	10.941	11	4	4	2.198	4.15E-04
Q9Y3A4	Ribosomal l	RRP7A	57.9	32.334	30.274	68	12	12	2.194	4.11E-05
O75616	GTPase Era	ERAL1	7.8	48.349	16.247	10	2	2	2.168	3.26E-03
P13726	Tissue facto	F3	16.3	33.067	9.386	12	3	3	2.158	4.32E-02
P17676	CCAAT/enl	CEBPB	10.4	36.105	9.9058	15	2	2	2.144	1.00E-03
Q15050	Ribosome b	RRS1	27.1	41.193	24.22	27	8	8	2.134	7.86E-05
Q9BUB7	Transmemb	TMEM70	16.2	28.969	7.6355	10	3	3	2.133	1.78E-03
Q9NZV1	Cysteine-ric	CRIM1	20.3	113.74	35.107	69	11	11	2.129	8.36E-05
O00488	Zinc finger	ZNF593	45.5	15.199	9.7011	6	4	4	2.11	5.24E-03
Q16706	Alpha-manr	MAN2A1	15.3	131.14	35.995	54	12	12	2.098	2.42E-03
Q9NZM5	Ribosome b	NOP53	10.3	54.389	5.7147	14	3	3	2.093	3.68E-02
P04920	Anion exch	SLC4A2	8	137.01	23.043	21	5	5	2.081	2.81E-04
Q9BZE4	Nucleolar G	GTPBP4	39.9	73.964	72.544	113	21	21	2.073	1.46E-07
Q9H8H2	Probable A	DDX31	5.5	94.086	5.2179	16	3	3	2.069	2.48E-03
P42696	RNA-bindir	RBM34	20.9	48.564	21.095	28	6	6	2.069	5.96E-03
P07203	Glutathione	GPX1	76.4	22.088	33.227	49	14	14	2.048	4.33E-05
Q9NVU7	Protein SD	SDAD1	11.8	79.87	9.3959	19	5	5	2.043	8.03E-05
Q13823	Nucleolar G	GNL2	19.4	83.654	22.465	44	11	11	2.038	3.24E-03
P05121	Plasminoge	SERPINE1	43.5	45.059	39.221	41	12	12	2.03	2.67E-02
P16401	Histone H1.	HIST1H1B	28.3	22.58	83.71	56	9	7	2.024	7.65E-07
Q16394	Exostosin-1	EXT1	9.8	86.254	8.9531	16	4	4	2.018	2.61E-04
Q8N4A0	Polypeptide	GALNT4	8.1	66.665	3.958	12	3	2	2.014	8.74E-03
Q9NY93	Probable A	DDX56	34.4	61.589	30.64	89	13	13	1.994	1.29E-06

Q9NQ55	Suppressor	PPAN	19.7	53.193	13.256	28	5	5	1.983	2.35E-05
Q96P11	Probable 28	NSUN5	55.9	46.691	87.343	58	14	14	1.98	5.98E-03
Q9NR30	Nucleolar R	DDX21	50.6	87.343	191.21	210	38	36	1.976	2.45E-06
Q56VL3	OCIA doma	OCIAD2	26.6	16.953	5.8804	20	4	4	1.967	4.89E-06
Q9BVP2	Guanine nu	GNL3	29.9	61.992	48.848	48	12	12	1.967	2.04E-05
Q9BVI4	Nucleolar c	NOC4L	28.5	58.467	16.118	31	9	9	1.94	7.19E-04
Q9NWU5	39S ribosom	MRPL22	23.8	23.64	4.9451	18	4	4	1.935	6.44E-05
P08243	Asparagine	ASNS	52.4	64.369	105.29	125	22	22	1.931	2.37E-06
Q9Y6V7	Probable A	DDX49	15.7	54.226	3.732	13	5	5	1.93	5.76E-04
P53814	Smoothelin	SMTN	30.3	99.058	70.136	66	15	15	1.919	1.24E-04
P13995	Bifunctiona	MTHFD2	48.6	37.895	128.6	69	9	9	1.908	2.69E-07
P18124	60S ribosom	RPL7	45.2	29.225	87.698	81	14	14	1.906	4.99E-07
Q5T3I0	G patch dor	GPATCH4	17.7	50.381	12.313	20	5	5	1.904	3.73E-06
Q9BZX2	Uridine-cyti	UCK2	34.1	29.299	7.7651	29	5	4	1.901	1.20E-04
O15213	WD repeat-	WDR46	21	68.07	23.62	42	10	10	1.901	2.78E-03
Q5JTH9	RRP12-like	RRP12	38.5	143.7	217.12	197	36	36	1.889	1.77E-05
Q9NV31	U3 small nu	IMP3	41.8	21.85	13.725	29	5	5	1.885	4.41E-04
Q13895	Bystin OS=	BYSL	26.1	49.601	27.232	49	8	8	1.883	4.06E-05
Q9NRX1	RNA-bindir	PNO1	25.4	27.924	24.181	38	5	5	1.883	8.34E-05
Q9Y2R4	Probable A	DDX52	20.7	67.497	24.056	40	8	8	1.875	2.00E-05
Q9NZQ7	Programme	CD274	23.1	33.275	22.405	34	4	4	1.875	3.88E-05
Q8IY81	pre-rRNA 2	FTSJ3	30.9	96.557	132.53	110	15	15	1.873	4.55E-07
Q8WTT2	Nucleolar c	NOC3L	22.9	92.547	50.047	62	13	13	1.867	1.45E-04
Q96BK5	PIN2/TERF	PINX1	14.9	37.034	4.9406	8	3	3	1.867	2.57E-02
Q9BYG3	MKI67 FHA	NIFK	57	34.222	79.245	59	15	15	1.866	1.13E-06
Q15397	Pumilio hor	PUM3	40.7	73.584	103.64	87	17	16	1.861	1.71E-06
Q5U5X0	Complex III	LYRM7	40.4	11.955	2.9264	16	3	3	1.853	2.54E-03
Q9UMY1	Nucleolar p	NOL7	19.5	29.426	5.336	12	4	4	1.852	4.28E-06
Q8WVM0	Dimethylad	TFB1M	18.8	39.542	19.029	12	3	3	1.851	1.62E-03
Q9NXH8	Torsin-4A (	TOR4A	17	46.914	22.917	29	5	5	1.849	1.44E-04
Q6NSJ5	Volume-reg	LRRC8E	11.8	90.246	13.004	21	6	6	1.841	4.59E-04
Q9Y343	Sorting nex	SNX24	20.7	19.818	4.1833	10	3	3	1.841	3.03E-02
Q15262	Receptor-ty	PTPRK	12.8	162.1	24.001	29	10	10	1.834	8.04E-04
Q9NW13	RNA-bindir	RBM28	20.4	85.737	30.579	55	12	12	1.83	2.02E-04
O15446	DNA-direct	CD3EAP	49.6	54.985	120.37	70	13	13	1.829	6.23E-05

Q9BRT6	Protein LLF	LLPH	23.3	15.225	17.435	20	2	2	1.827	1.05E-04
Q8NBJ4	Golgi meml	GOLM1	19.5	45.333	12.293	26	6	6	1.825	3.54E-03
P41212	Transcriptic	ETV6	12.6	52.999	14.802	15	3	3	1.824	2.63E-02
A0A0U1RF	Protein MM	MMP24OS	74.6	7.6794	26.297	25	2	2	1.822	8.06E-05
P51946	Cyclin-H O	CCNH	13.6	37.643	3.8151	12	3	3	1.818	2.44E-02
Q9H0Z9	RNA-bindir	RBM38	15.1	25.498	6.3	16	2	2	1.816	6.24E-03
Q9BVS5	tRNA (ader	TRMT61B	10.1	52.965	5.5884	16	3	3	1.81	2.50E-02
Q8N884	Cyclic GMI	CGAS	13.2	58.814	7.0586	14	5	5	1.807	1.18E-03
Q9UET6	Putative tRI	FTSJ1	13.7	36.079	6.2922	15	3	3	1.803	3.91E-02
P31431	Syndecan-4	SDC4	19.2	21.641	5.9325	18	4	4	1.801	1.85E-02
Q96AE7	Tetratricope	TTC17	9.9	129.56	9.8271	20	7	7	1.794	2.61E-04
Q4KMQ1	Taperin OS	TPRN	6	75.555	17.793	10	2	2	1.792	1.48E-03
O76021	Ribosomal l	RSL1D1	48.6	54.972	124.52	165	22	22	1.777	3.71E-07
Q9Y3C1	Nucleolar p	NOP16	30.9	21.188	12.996	29	5	5	1.776	4.02E-05
P09038	Fibroblast g	FGF2	28.1	30.77	19.742	36	7	7	1.775	1.44E-03
P29317	Ephrin type	EPHA2	39.1	108.27	93.118	154	24	21	1.771	8.72E-09
O95298	NADH deh	NDUFC2	16	14.187	1.8645	14	2	2	1.767	1.19E-02
Q96HY7	Probable 2-	DHTKD1	3.6	103.08	3.7863	12	2	2	1.762	2.66E-02
P05067	Amyloid-be	APP	25.7	86.942	28.391	65	13	13	1.751	2.39E-04
Q96QB1	Rho GTPas	DLC1	5.7	170.59	10.802	6	5	5	1.751	3.33E-02
Q9Y221	60S ribosom	NIP7	53.9	20.462	25.547	32	6	6	1.747	2.62E-04
Q00653	Nuclear fac	NFKB2	32.9	96.748	65.158	101	18	18	1.743	1.84E-08
P46087	Probable 28	NOP2	28.9	89.301	138.43	106	20	20	1.738	7.72E-07
Q9BSC4	Nucleolar p	NOL10	17.7	80.301	16.769	37	7	7	1.738	3.68E-05
Q6UXV4	MICOS cor	APOOL	41	29.159	18.578	39	7	7	1.732	2.00E-05
O14646	Chromodon	CHD1	6.9	196.69	6.9601	19	7	5	1.73	6.76E-03
Q96ME7	Zinc finger	ZNF512	26.8	64.681	28.356	36	10	10	1.727	3.18E-02
P08572	Collagen al	COL4A2	13.8	167.55	120.43	96	15	15	1.726	4.56E-06
Q92974	Rho guanin	ARHGEF2	35.9	111.54	82.462	153	25	25	1.709	2.95E-06
Q9NWT1	p21-activat	PAK1IP1	16.6	43.963	10.446	18	4	4	1.709	8.12E-05
Q9BXY0	Protein MA	MAK16	30.3	35.368	21.184	66	6	6	1.705	3.99E-04
Q9ULW3	Activator of	ABT1	32.4	31.079	5.8789	15	5	5	1.705	1.00E-02
O96005	Cleft lip and	CLPTM1	22.4	76.096	79.007	54	8	8	1.704	1.70E-05
Q9H8H0	Nucleolar p	NOL11	20.7	81.123	35.401	27	7	7	1.701	9.24E-04
Q9Y5J9	Mitochondr	TIMM8B	62.7	9.3435	13.274	36	4	4	1.699	6.35E-05

P56556	NADH deh	NDUFA6	41.4	15.136	5.1421	20	4	4	1.699	7.50E-03
Q5HYK3	2-methoxy-	COQ5	47.1	37.14	32.785	58	10	10	1.694	1.38E-03
Q9H501	ESF1 homo	ESF1	23.3	98.795	43.028	68	14	14	1.691	1.16E-04
Q9H6F5	Coiled-coil	CCDC86	32.2	40.235	35.136	31	7	7	1.691	2.66E-03
Q9NSI2	Protein FAI	FAM207A	21.3	25.456	8.9416	22	3	3	1.679	3.82E-04
Q8TDN6	Ribosome b	BRIX1	54.7	41.401	81.181	101	13	13	1.677	1.83E-06
Q96GQ7	Probable A	DDX27	28.8	89.834	39.51	71	16	16	1.672	2.05E-05
Q8IY37	Probable A	DHX37	16.5	129.54	18.071	47	12	11	1.667	1.85E-04
E9PRG8	Uncharacte	C11orf98	32.5	14.234	6.3063	16	3	3	1.665	1.45E-04
Q9Y399	28S riboson	MRPS2	12.8	33.249	4.1283	12	4	4	1.663	1.66E-05
P50914	60S riboson	RPL14	24.2	23.432	13.198	30	5	5	1.659	2.74E-06
O94804	Serine/threo	STK10	9.8	112.13	8.992	18	7	6	1.658	4.25E-02
Q14137	Ribosome b	BOP1	33.8	83.629	89.317	109	16	16	1.657	3.40E-06
Q9UNQ2	Probable di	DIMT1	41.2	35.236	28.913	67	10	10	1.657	1.89E-05
P10109	Adrenodoxi	FDX1	12.5	19.393	3.4589	16	2	2	1.657	3.48E-03
Q14690	Protein RRI	PDCD11	32.7	208.7	120.38	187	37	37	1.655	1.54E-07
Q96KC8	DnaJ homol	DNAJC1	17.3	63.882	11.76	19	6	6	1.655	1.39E-02
Q16850	Lanosterol	CYP51A1	36.6	56.805	40.053	98	13	13	1.654	2.44E-05
Q9NV06	DDB1- and	DCAF13	33	51.402	19.745	37	10	10	1.647	3.04E-04
P16402	Histone H1.	HIST1H1D	28.5	22.35	52.765	50	8	1	1.643	5.13E-07
O60906	Sphingomy	SMPD2	14.4	47.645	7.6811	16	4	4	1.643	2.11E-02
P17096	High mobili	HMGA1	41.1	11.676	33.679	27	3	3	1.641	3.61E-05
Q9Y6M5	Zinc transp	SLC30A1	14.4	55.299	16.925	24	4	4	1.638	4.27E-05
P17706	Tyrosine-pr	PTPN2	31.6	48.473	15.707	25	8	8	1.638	5.70E-03
Q9H1E5	Thioredoxi	TMX4	12	38.952	12.337	22	4	4	1.637	2.52E-03
P15822	Zinc finger	HIVEP1	1.8	296.86	7.1397	9	3	3	1.637	4.95E-02
Q8TD30	Alanine am	GPT2	35.6	57.903	77.322	69	11	11	1.631	1.56E-06
Q7KYR7	Butyrophili	BTN2A1	8.2	59.632	6.6255	13	2	2	1.631	3.42E-03
Q96PC5	Melanoma i	MIA2	8.5	159.83	19.781	44	8	8	1.63	1.26E-03
P53801	Pituitary tu	PTTG1IP	20	20.324	23.807	17	3	3	1.629	3.23E-04
Q9Y2P8	RNA 3'-terr	RCL1	31.1	40.842	15.927	32	8	8	1.626	7.40E-04
Q86SJ2	Amphoterin	AMIGO2	21.1	57.933	14.922	24	8	8	1.624	4.38E-03
Q9NRX2	39S riboson	MRPL17	30.9	20.05	18.691	29	5	5	1.618	1.82E-03
Q3ZCQ8	Mitochondr	TIMM50	32	39.646	44.335	51	8	7	1.615	3.78E-04
Q16611	Bcl-2 homo	BAK1	33.6	23.408	19.207	10	4	4	1.615	1.18E-03

Q9H3R5	Centromere	CENPH	14.2	28.481	11.214	18	2	2	1.615	1.93E-02
O75691	Small subur	UTP20	15.8	318.38	62.628	117	29	29	1.613	1.83E-05
O95361	Tripartite m	TRIM16	35.5	63.954	41.485	105	15	15	1.61	5.68E-05
P61244	Protein max	MAX	21.2	18.275	17.773	16	2	2	1.61	2.08E-03
P26572	Alpha-1,3-n	MGAT1	18.4	50.878	33.496	38	4	4	1.609	3.98E-05
P57081	tRNA (guar	WDR4	32.8	45.489	28.509	49	8	8	1.606	1.60E-05
Q86SF2	N-acetylgal	GALNT7	7.9	75.388	11.309	12	3	3	1.605	3.52E-03
Q8N9T8	Protein KRI	KRI1	25.2	82.597	54.19	67	11	11	1.604	1.51E-05
Q5C9Z4	Nucleolar M	NOM1	14.1	96.256	16.116	34	8	8	1.604	2.34E-05
Q96D70	R3H domai	R3HDM4	11.9	30.349	6.8886	11	2	2	1.604	7.28E-03
P17813	Endoglin O	ENG	20.2	70.577	35.906	42	9	9	1.602	5.56E-05
Q08380	Galectin-3-l	LGALS3BI	27.4	65.33	50.054	51	10	10	1.6	1.98E-05
O00541	Pescadillo h	PES1	30.3	68.002	37.508	68	12	12	1.6	2.38E-05
Q9H6R4	Nucleolar p	NOL6	29.4	127.59	51.256	80	19	19	1.6	2.00E-04
Q9H9Y6	DNA-direct	POLR1B	8.5	128.23	11.561	23	7	7	1.6	5.50E-03
Q9HBH1	Peptide def	PDF	24.7	27.013	10.141	17	4	4	1.596	2.66E-03
P78345	Ribonucleas	RPP38	19.1	31.834	26.95	38	5	5	1.593	3.76E-04
Q8N8R3	Mitochondr	SLC25A29	23.1	32.062	8.1246	16	4	4	1.592	2.21E-02
Q9HB09	Bcl-2-like p	BCL2L12	12.9	36.821	8.3959	20	3	3	1.592	4.02E-02
P46013	Proliferatio	MKI67	38.1	358.69	190.31	365	71	71	1.59	6.54E-07
O95602	DNA-direct	POLR1A	20.2	194.81	40	71	20	20	1.586	1.08E-03
P50416	Carnitine O	CPT1A	27.4	88.367	70.899	104	17	17	1.583	5.78E-04
Q05932	Folylpolygl	FPGS	18.2	64.608	12.416	24	6	6	1.581	5.10E-03
Q9P032	NADH deh	NDUFAF4	8	20.266	5.4354	16	1	1	1.581	7.12E-03
Q7Z7H8	39S ribosom	MRPL10	32.6	29.282	16.244	25	5	5	1.581	2.33E-02
Q14978	Nucleolar a	NOLC1	19.5	73.602	47.644	66	12	11	1.58	4.70E-03
P62424	60S ribosom	RPL7A	47.7	29.995	242.77	72	17	17	1.579	7.18E-07
Q9Y5Q0	Fatty acid d	FADS3	16.9	51.144	16.631	32	5	5	1.578	1.42E-03
Q9H3K2	Growth hor	GHITM	13	37.205	7.0907	19	4	4	1.575	8.40E-04
Q9Y385	Ubiquitin-c	UBE2J1	22.6	35.198	8.141	21	5	5	1.573	4.86E-03
Q9Y3B9	RRP15-like	RRP15	24.8	31.484	7.2457	20	6	6	1.573	1.01E-02
Q9Y5J1	U3 small nu	UTP18	32	62.003	32.517	54	11	11	1.569	1.57E-04
Q9GZR2	RNA exonu	REXO4	14.7	46.671	18.419	19	4	4	1.569	2.61E-04
Q9NRZ9	Lymphoid-s	HELLS	19.7	97.073	34.676	68	13	13	1.568	5.80E-05
Q8NEJ9	Neuroguidi	NGDN	36.5	35.894	24.481	42	9	9	1.567	3.24E-04

Q8IYS2	Uncharacter	KIAA2013	21.5	69.156	30.262	38	8	8	1.567	3.24E-03
P78316	Nucleolar p	NOP14	22.4	97.667	20.047	47	15	15	1.566	6.47E-05
Q9NW81	Distal meml	DMAC2	11.7	29.267	4.9316	18	2	2	1.565	2.00E-02
O00411	DNA-direct	POLRMT	8.5	138.62	15.736	25	6	6	1.564	3.73E-05
O75531	Barrier-to-a	BANF1	50.6	10.058	58.384	51	4	4	1.563	4.35E-06
Q9UL01	Dermatan-s	DSE	7.4	109.77	11.911	12	4	4	1.557	1.76E-03
Q9NNX1	Tuftelin OS	TUFT1	30.5	44.263	31.737	51	10	10	1.556	3.82E-04
P04114	Apolipopro	APOB	1.4	515.6	1.7919	11	5	4	1.555	3.26E-03
Q15014	Mortality fa	MORF4L2	21.5	32.307	12.485	23	5	5	1.554	8.22E-05
O43159	Ribosomal 1	RRP8	24.6	50.714	31.899	36	7	7	1.548	3.97E-04
Q9H0P0	Cytosolic 5'	NT5C3A	22.6	37.948	13.358	25	6	6	1.547	2.54E-02
Q9NY61	Protein AA'	AATF	37.3	63.132	37.444	76	14	14	1.546	3.66E-06
P19447	General trai	ERCC3	18.3	89.277	19.698	32	7	7	1.546	1.25E-02
P20337	Ras-related	RAB3B	29.7	24.758	16.717	23	4	4	1.544	1.62E-03
Q969X6	U3 small nu	UTP4	26.2	76.889	30.892	71	12	12	1.543	7.01E-04
Q9UIF9	Bromodoma	BAZ2A	6.5	211.2	14.398	28	8	8	1.542	4.66E-03
Q9BYN0	Sulfiredoxi	SRXN1	54	14.259	10.919	42	5	5	1.541	2.83E-04
Q9BUI4	DNA-direct	POLR3C	13.1	60.611	9.1823	22	4	4	1.541	5.10E-03
Q9NYJ1	Cytochrome	COA4	69	10.134	64.794	35	4	4	1.539	8.63E-07
P82663	28S ribosor	MRPS25	59	20.116	35.323	43	7	7	1.539	2.50E-03
O94851	[F-actin]-m	MICAL2	24.4	126.69	48.623	87	20	18	1.536	4.49E-06
Q14692	Ribosome b	BMS1	22.5	145.81	51.875	99	20	20	1.534	7.70E-05
Q03518	Antigen pep	TAP1	36.5	87.217	171.99	87	14	14	1.534	1.12E-02
P52566	Rho GDP-d	ARHGDIB	40.3	22.988	20.149	23	4	4	1.534	1.79E-02
Q9Y6M7	Sodium bic	SLC4A7	7.2	136.04	13.636	28	5	5	1.533	2.78E-03
Q9Y3T9	Nucleolar c	NOC2L	16.7	84.918	19.296	38	10	10	1.531	1.10E-03
Q96K49	Transmemb	TMEM87B	10.1	63.535	10.293	27	4	4	1.53	1.25E-04
Q9BQ75	Protein CM	CMSS1	22.2	31.884	6.8613	16	6	6	1.529	4.62E-02
Q9BQG0	Myb-bindin	MYBBP1A	42	148.85	290.81	225	42	42	1.525	1.19E-06
Q8TED0	U3 small nu	UTP15	42.7	58.414	38.344	82	16	16	1.524	2.82E-04
Q969S3	Zinc finger	ZNF622	27.9	54.271	43.445	38	9	9	1.523	5.02E-04
P04179	Superoxide	SOD2	64	24.75	85.116	50	9	9	1.521	6.82E-04
Q13206	Probable A'	DDX10	23.8	100.89	28.007	69	15	15	1.519	4.18E-05
Q96PU5	E3 ubiquitii	NEDD4L	24.3	111.93	54.728	59	15	13	1.518	2.50E-06
Q6PIU2	Neutral cho	NCEH1	33.8	45.807	31.815	45	10	10	1.516	6.96E-04

Q12931	Heat shock	TRAP1	36.6	80.109	150.24	155	19	19	1.514	1.77E-06
Q8TAA9	Vang-like p	VANGL1	14.9	59.974	8.5105	10	5	5	1.513	3.12E-03
Q5T440	Putative tra	IBA57	30.6	38.155	16.906	26	7	7	1.513	5.78E-03
P82933	28S riboson	MRPS9	34.3	45.834	33.063	72	10	9	1.512	1.03E-04
Q7Z2W9	39S riboson	MRPL21	34.1	22.814	23.384	34	5	5	1.51	1.95E-06
Q15599	Na(+)/H(+)	SLC9A3R2	47.2	37.413	13.349	25	10	8	1.51	1.06E-03
Q8WW59	SPRY doma	SPRYD4	37.2	23.128	14.581	22	6	6	1.51	1.01E-02
Q99848	Probable rR	EBNA1BP2	32.4	34.852	75.377	43	8	8	1.509	1.52E-03
Q96RD7	Pannexin-1	PANX1	14.1	48.05	7.9972	16	3	3	1.508	1.88E-03
O43824	Putative GT	GTPBP6	16.3	56.897	29.718	24	5	5	1.507	2.17E-02
Q5QJE6	Deoxynucle	DNTTIP2	31.9	84.468	44.353	76	14	14	1.505	3.71E-05
P06748	Nucleophos	NPM1	44.9	32.575	323.31	163	10	10	1.505	2.16E-04
A4D1E9	GTP-bindin	GTPBP10	26.6	42.932	12.901	32	7	7	1.505	2.14E-03
Q9Y314	Nitric oxide	NOSIP	47.2	33.172	43.166	56	10	10	1.504	3.61E-05
Q6DKK2	Tetratricope	TTC19	22.4	42.456	15.607	23	6	6	1.501	8.78E-04
Q9P265	Disco-intera	DIP2B	33.8	171.49	116.01	184	32	29	0.666	5.76E-05
Q04726	Transducin-	TLE3	37.6	83.416	53.991	80	17	13	0.665	1.39E-04
Q9BXW6	Oxysterol-b	OSBPL1A	5.9	108.47	5.416	18	4	4	0.665	2.07E-02
P78524	Suppressior	ST5	6	126.48	5.5441	11	4	4	0.664	2.14E-02
Q9UKE5	TRAF2 and	TNIK	9.8	154.94	16.09	27	8	6	0.663	2.26E-02
Q96IF1	LIM domai	AJUBA	51.5	56.933	61.279	78	15	15	0.662	2.56E-04
Q14689	Disco-intera	DIP2A	14.2	170.37	46.939	47	12	9	0.661	3.55E-05
O75970	Multiple PD	MPDZ	8.8	221.62	15.742	24	11	11	0.661	2.84E-04
P52630	Signal trans	STAT2	24.7	97.915	31.252	80	14	14	0.66	2.03E-04
P16949	Stathmin OS	STMN1	57.7	17.302	82.472	74	13	10	0.659	1.67E-05
Q8TDZ2	[F-actin]-mo	MICAL1	8.1	117.87	13.477	24	7	7	0.659	2.95E-04
Q9P2B2	Prostagland	PTGFRN	30	98.555	33.678	74	19	19	0.659	1.56E-03
Q8WUH6	Transmemb	TMEM263	56.9	11.748	40.722	24	4	4	0.658	3.05E-06
Q86SQ4	Adhesion G	ADGRG6	12.3	136.69	23.191	54	10	10	0.658	2.01E-04
Q14185	Dedicator o	DOCK1	13.4	215.34	24.573	57	18	18	0.656	3.51E-05
Q14642	Type I inosi	INPP5A	14.1	47.819	5.0544	11	3	3	0.655	2.90E-03
P52943	Cysteine-ric	CRIP2	59.1	22.492	305.61	48	5	5	0.654	1.72E-06
Q96A26	Protein FAM	FAM162A	27.3	17.342	15.196	22	4	4	0.654	6.34E-05
Q99538	Legumain C	LGMN	16.6	49.411	57.354	27	4	4	0.653	7.88E-07
P09104	Gamma-enc	ENO2	69.4	47.268	190.81	117	19	16	0.653	8.54E-07

Q9NRY6	Phospholipi	PLSCR3	16.9	31.648	22.885	19	3	3	0.653	1.60E-02
P08582	Melanotran:	MELTF	44.4	80.214	84.889	93	19	18	0.651	2.04E-05
O75781	Paralemmin	PALM	25.6	42.075	60.059	28	5	4	0.646	3.70E-05
Q5T0N5	Formin-binc	FNBP1L	19.2	70.065	11.948	23	7	7	0.646	7.44E-03
Q9C0B0	RING finge	UNK	11.5	88.084	35.639	28	4	4	0.645	2.58E-04
Q12929	Epidermal g	EPS8	18.1	91.88	16.312	33	9	9	0.643	4.03E-02
P62805	Histone H4	HIST1H4A	65	11.367	180.06	141	17	17	0.639	5.55E-05
O15164	Transcriptic	TRIM24	11.6	116.83	19.27	37	9	8	0.638	3.58E-04
P00558	Phosphogly	PGK1	75.1	44.614	323.31	297	30	25	0.637	5.64E-07
Q01196	Runt-relatec	RUNX1	17.9	48.736	15.501	29	5	5	0.637	1.98E-04
Q05193	Dynamin-1	DNM1	32.3	97.407	38.675	72	19	11	0.637	4.35E-04
Q9C0E8	Endoplasmi	LNPK	16.8	47.739	12.225	12	4	4	0.637	2.13E-02
Q9H334	Forkhead bo	FOXP1	13.9	75.316	8.1549	16	5	5	0.636	1.54E-02
O15460	Prolyl 4-hyc	P4HA2	48.2	60.901	323.31	173	21	21	0.633	6.27E-07
P68032	Actin, alpha	ACTC1	64.7	42.019	99.532	50	22	0	0.632	4.86E-06
Q8N8Z6	Discoidin, C	DCBLD1	13.6	77.92	11.465	18	5	5	0.631	1.04E-03
Q8IVF2	Protein AH	AHNAK2	8.4	616.62	5.9385	18	5	5	0.631	2.81E-02
Q9Y618	Nuclear rec	NCOR2	23.7	273.65	99.21	155	34	33	0.629	2.52E-07
Q3V6T2	Girdin OS=	CCDC88A	9.7	216.04	26.12	43	11	11	0.629	1.72E-03
P35611	Alpha-addu	ADD1	38.7	80.954	58.404	105	17	17	0.628	3.42E-04
P42858	Huntingtin (	HTT	6.7	347.6	17.219	31	12	12	0.628	4.98E-03
Q6ZRQ5	Protein MM	MMS22L	7.3	142.32	7.3926	25	7	7	0.628	6.12E-03
Q96CX2	BTB/POZ d	KCTD12	59.4	35.7	167.59	96	13	13	0.626	1.80E-05
Q9NSK0	Kinesin ligh	KLC4	17.8	68.639	10.045	20	8	4	0.626	3.58E-02
P13674	Prolyl 4-hyc	P4HA1	55.4	61.049	232.24	251	28	28	0.623	3.49E-07
P05106	Integrin bet	ITGB3	30.8	87.057	58.8	78	18	18	0.622	7.12E-07
Q00535	Cyclin-depe	CDK5	37	33.304	20.512	45	8	8	0.621	2.50E-06
P57078	Receptor-in	RIPK4	7.1	91.61	5.256	8	4	4	0.621	1.66E-02
Q14195	Dihydropyr	DPYSL3	55.6	61.963	107.76	138	19	17	0.62	1.86E-08
P13807	Glycogen [s	GYS1	45.7	83.785	155.22	122	23	23	0.619	2.12E-05
A6NCS6	Uncharacte	C2orf72	15.3	30.48	8.5525	10	3	3	0.619	3.41E-02
O14727	Apoptotic p	APAF1	10.9	141.84	12.624	24	9	9	0.616	3.73E-05
P98160	Basement m	HSPG2	21.8	468.83	248.49	294	52	52	0.615	3.50E-05
P23921	Ribonucleo:	RRM1	52.4	90.069	212.57	181	29	28	0.614	2.03E-06
Q9NYZ3	G2 and S pl	GTSE1	15.3	76.644	24.117	30	6	6	0.614	1.32E-03

O75110	Probable ph	ATP9A	7.4	118.58	9.6261	13	5	4	0.614	1.92E-02
O75391	Sperm-asso	SPAG7	29.1	26.034	17.387	28	5	5	0.611	1.59E-04
Q9NRG7	Epimerase f	SDR39U1	13.3	31.076	30.199	14	2	2	0.611	6.42E-04
P98082	Disabled ho	DAB2	23.8	82.447	62.546	47	12	12	0.607	1.57E-02
P08473	Neprilysin (	MME	30.1	85.513	34.965	57	15	15	0.605	7.14E-03
Q05655	Protein kina	PRKCD	19.8	77.504	30.019	45	8	8	0.604	4.84E-04
Q9BV73	Centrosome	CEP250	12.4	281.13	41.581	44	17	17	0.602	9.01E-04
P00374	Dihydrofola	DHFR	53.5	21.452	12.486	30	7	7	0.601	6.60E-03
O94855	Protein tran	SEC24D	23.7	113.01	49.262	85	16	15	0.599	8.49E-05
O14770	Homeobox	MEIS2	23.9	51.789	18.581	26	8	8	0.597	1.22E-03
Q8IWE2	Protein NO	FAM114A1	36.1	60.741	171.66	96	14	14	0.595	2.17E-05
P27540	Aryl hydroc	ARNT	16.1	86.636	15.541	31	8	8	0.594	3.42E-04
Q8WUJ3	Cell migrati	CEMIP	10.7	153	19.998	38	9	9	0.594	5.50E-03
Q8WV41	Sorting nex	SNX33	14.6	65.264	8.6879	9	4	4	0.594	3.85E-02
Q9HBM1	Kinetochore	SPC25	29	26.152	12.626	12	4	4	0.593	4.79E-02
Q5T5U3	Rho GTPas	ARHGAP2	9.9	217.46	48.568	51	12	12	0.59	6.76E-03
Q13153	Serine/threo	PAK1	19.1	60.646	16.65	18	6	3	0.589	3.92E-03
P98095	Fibulin-2 O	FBLN2	36.1	126.57	234.71	170	26	26	0.588	3.99E-06
Q8WXE0	Caskin-2 O	CASKIN2	4.4	126.78	5.3645	9	3	3	0.586	1.52E-02
Q96RG2	PAS domai	PASK	14.5	142.93	17.834	24	10	10	0.583	2.68E-03
P60002	Transcriptic	ELOF1	21.7	9.4618	4.3275	16	1	1	0.582	5.74E-03
Q9NZN4	EH domain-	EHD2	51.4	61.161	94.563	95	17	15	0.581	1.63E-05
Q9Y5V3	Melanoma-a	MAGED1	10	86.16	18.115	18	5	3	0.578	6.48E-03
Q9HBF4	Zinc finger	ZFYVE1	6.4	87.175	12.953	17	3	3	0.576	2.07E-02
P55287	Cadherin-1	CDH11	16.3	87.964	19.773	32	8	8	0.572	7.41E-04
O00469	Procollagen	PLOD2	43.4	84.685	233.89	170	26	26	0.571	1.01E-06
Q14118	Dystroglyca	DAG1	10.1	97.44	15.592	30	8	8	0.569	2.24E-05
Q16363	Laminin sul	LAMA4	3.6	202.52	7.6131	13	4	4	0.569	1.10E-03
P08069	Insulin-like	IGF1R	13.2	154.79	18.8	45	11	11	0.566	2.78E-04
Q13907	Isopentenyl	IDI1	59.5	26.319	10.89	35	8	8	0.564	3.84E-04
Q15118	[Pyruvate d	PDK1	26.6	49.244	7.2587	18	8	7	0.561	8.35E-04
Q8N3D4	EH domain-	EHBP1L1	7.3	161.85	5.627	12	5	4	0.561	3.95E-02
P54652	Heat shock-	HSPA2	57.9	70.02	209	138	29	18	0.56	1.12E-06
P05997	Collagen al	COL5A2	16.3	144.91	183.12	84	14	14	0.559	5.49E-07
P50151	Guanine nu	GNG10	52.9	7.2053	38.425	14	3	3	0.559	1.29E-02

Q9BRT3	Migration a	MIEN1	49.6	12.403	7.0721	12	3	3	0.557	1.83E-02
Q9UHP3	Ubiquitin ca	USP25	5.5	122.22	7.568	13	4	4	0.556	7.18E-04
Q96RT7	Gamma-tub	TUBGCP6	2.9	200.5	3.1966	10	3	3	0.555	2.87E-02
P18433	Receptor-ty	PTPRA	22.7	90.718	21.436	38	12	11	0.552	1.04E-04
P49716	CCAAT/enl	CEBPD	15.6	28.467	8.0462	9	2	2	0.552	7.62E-04
O00468	Agrin OS=I	AGRN	24.7	217.32	162.87	174	30	30	0.542	1.18E-06
Q765P7	Protein MT	MTSS2	16.6	79.928	12.277	27	6	6	0.537	4.34E-03
Q16790	Carbonic ar	CA9	32.9	49.697	12.169	38	9	8	0.534	1.42E-04
P84243	Histone H3.	H3F3A	60.3	15.328	230.14	57	10	1	0.527	8.36E-04
Q68CZ2	Tensin-3 OS	TNS3	52.9	155.26	210.51	251	42	41	0.525	1.74E-06
Q9UEY8	Gamma-add	ADD3	28.8	79.154	48.536	65	13	13	0.519	1.26E-02
P11166	Solute carri	SLC2A1	14	54.083	99.629	58	8	8	0.515	2.29E-06
Q15800	Methylstero	MSMO1	14.3	35.215	10.241	9	3	3	0.513	2.44E-03
Q9Y2J4	Angiomotin	AMOTL2	18.9	85.763	25.559	42	10	10	0.495	3.84E-04
P98172	Ephrin-B1 (	EFNB1	18.5	38.006	13.403	11	3	3	0.495	9.88E-03
P13987	CD59 glyco	CD59	18.8	14.177	13.887	18	3	3	0.49	4.92E-03
Q8IW35	Centrosoma	CEP97	22.9	96.98	25.547	32	10	10	0.485	3.90E-03
Q9Y4C1	Lysine-spec	KDM3A	9	147.34	14.889	16	7	6	0.482	9.44E-04
P09972	Fructose-bis	ALDOC	70.1	39.455	278.16	128	18	14	0.47	9.18E-07
O14531	Dihydropyr	DPYSL4	32.2	61.877	26.84	16	8	8	0.462	3.58E-03
Q92597	Protein ND	NDRG1	54.3	42.835	122.38	97	13	13	0.458	4.44E-05
P51911	Calponin-1	CNN1	55.2	33.17	125.81	70	11	10	0.453	5.37E-07
Q9BV57	1,2-dihydro	ADI1	46.4	21.498	11.028	30	6	6	0.447	1.08E-03
P41221	Protein Wnt	WNT5A	19.5	42.339	23.662	33	6	5	0.447	3.22E-03
Q9Y625	Glypican-6	GPC6	24.3	62.735	35.058	66	8	8	0.444	1.80E-06
Q15398	Disks large-	DLGAP5	19.4	95.114	25.099	31	9	9	0.442	6.63E-04
Q96D15	Reticulocall	RCN3	18	37.493	11.131	14	3	3	0.423	8.98E-03
Q9ULI3	Protein HEG	HEG1	10.9	147.46	52.849	58	10	10	0.417	7.79E-08
P35568	Insulin rece	IRS1	15.1	131.59	40.674	36	11	10	0.416	2.82E-04
Q99715	Collagen alp	COL12A1	21.7	333.14	112.34	147	43	43	0.407	6.11E-07
P35080	Profilin-2 C	PFN2	29.3	15.046	10.241	16	3	3	0.406	3.99E-02
Q16537	Serine/threo	PPP2R5E	14.8	54.699	6.2092	16	5	4	0.404	3.89E-02
Q86X02	Cerebellar c	CDR2L	9.9	53.01	4.6372	7	4	4	0.382	3.93E-02
Q07954	Prolow-den	LRP1	17.9	504.6	143.67	195	53	50	0.367	5.36E-04
Q712K3	Ubiquitin-co	UBE2R2	14.7	27.166	3.2752	17	3	3	0.34	6.32E-03

Q14249	Endonuclea	ENDOG	23.2	32.62	7.7109	20	5	5	0.33	1.28E-03
P0CG12	Chromoson	CHTF8	7.1	51.391	8.6471	14	2	2	0.326	4.24E-04
Q8N726	Tumor supp	CDKN2A	44.7	13.903	49.557	32	5	5	0.322	2.32E-03
P78504	Protein jagg	JAG1	30	133.8	143.42	76	22	20	0.304	3.82E-03
P20908	Collagen al	COL5A1	12.7	183.56	162.99	69	14	14	0.288	5.08E-08
P11169	Solute carri	SLC2A3	11.9	53.924	18.124	22	5	5	0.227	7.94E-03
Q8NEY1	Neuron nav	NAV1	10.1	202.47	26.482	26	10	10	0.224	1.96E-06
Q53EL6	Programme	PDCD4	32.2	51.735	23.02	48	10	10	0.089	1.63E-05
