# Supplementary material for: An integrated network pharmacology and proteomics approach reveals the anti-fibrotic effect of Fushen Granule on peritoneal fibrosis
Source: BMC Complement Med Ther. 2026 Mar 9;26:143. doi: 10.1186/s12906-026-05333-2 (PMC13085474; doi:10.1186/s12906-026-05333-2)

Supplementary Material

Figure 4B
The groups from left to right are: control, model, FSG.

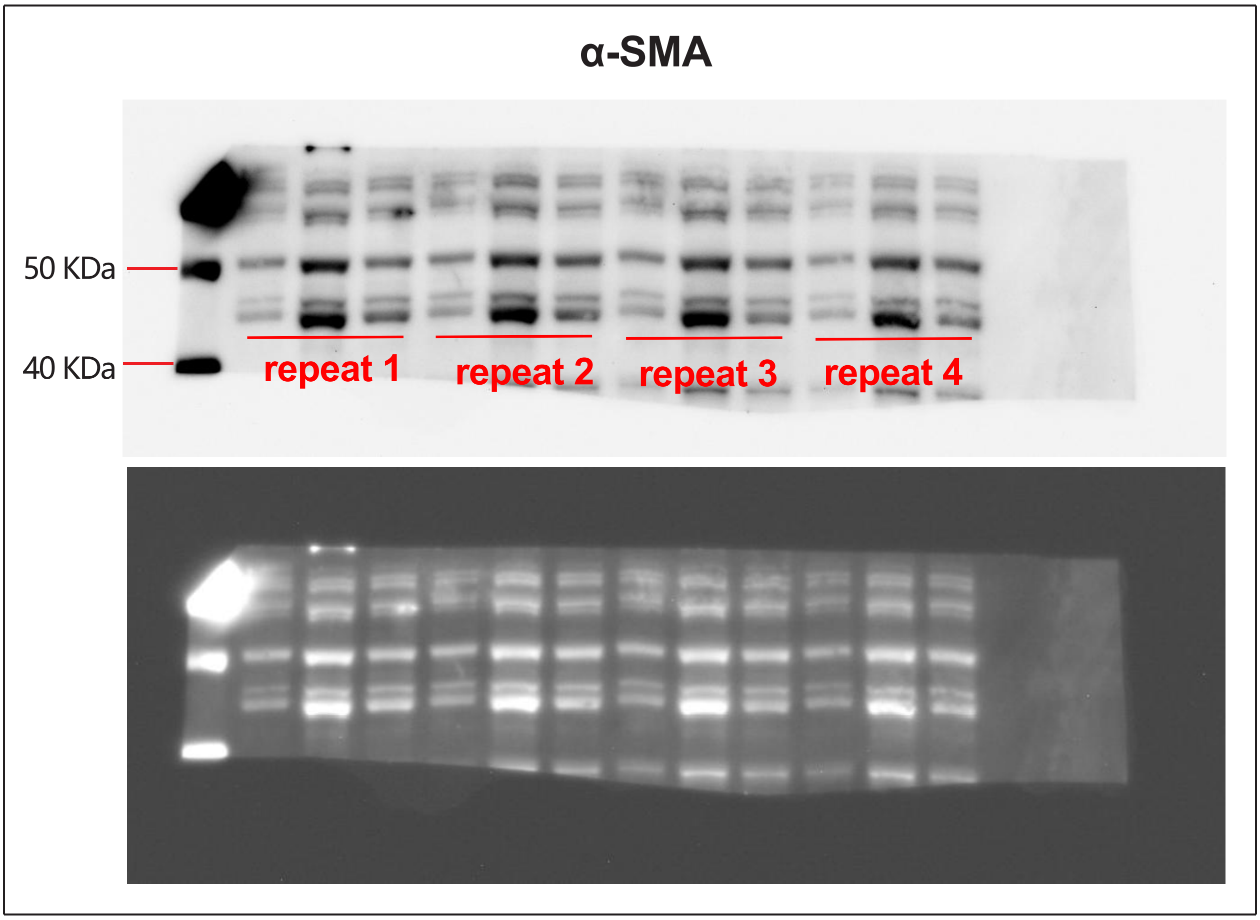

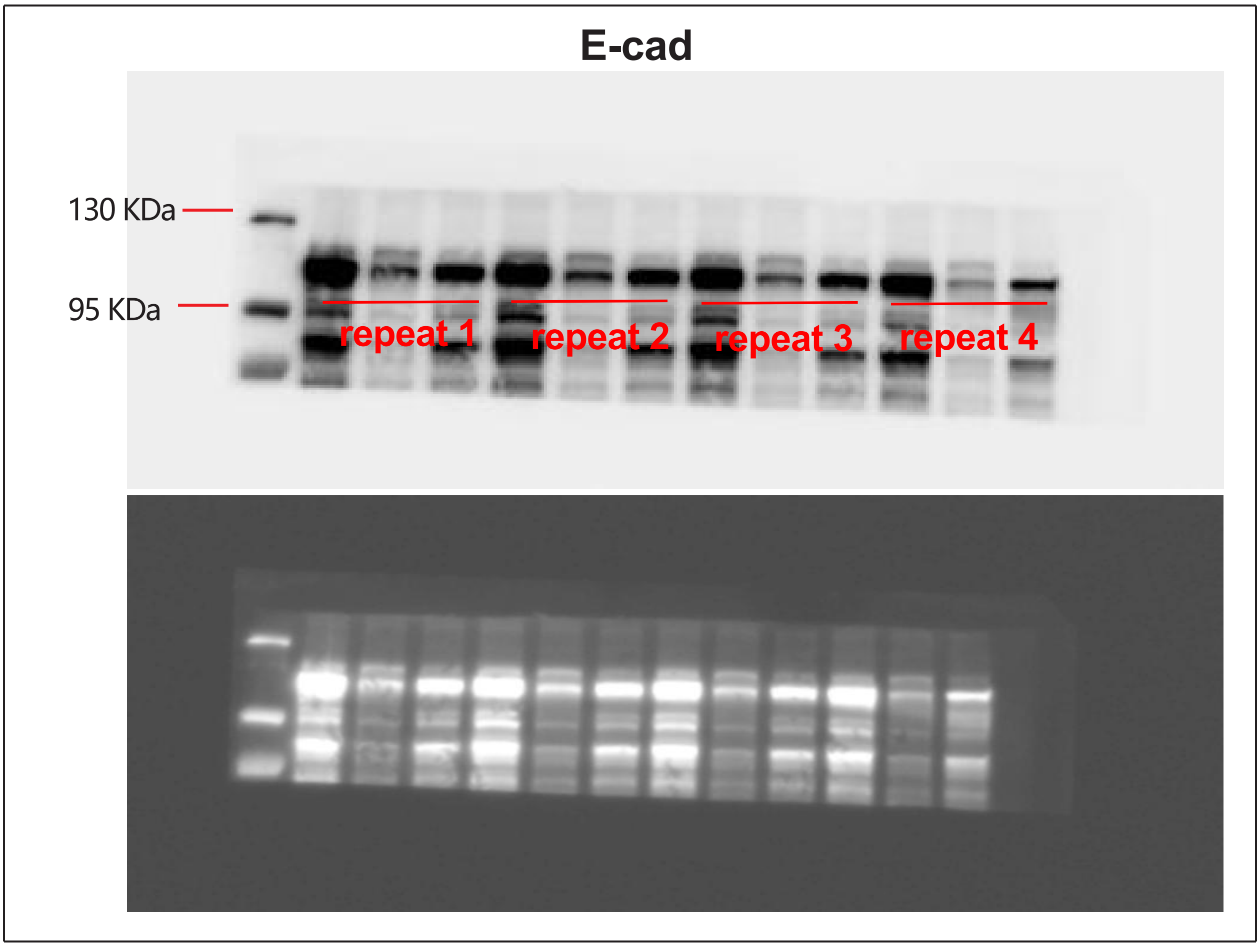

Supplementary Material

Figure 4B

The groups from left to right are: control, model, FSG.

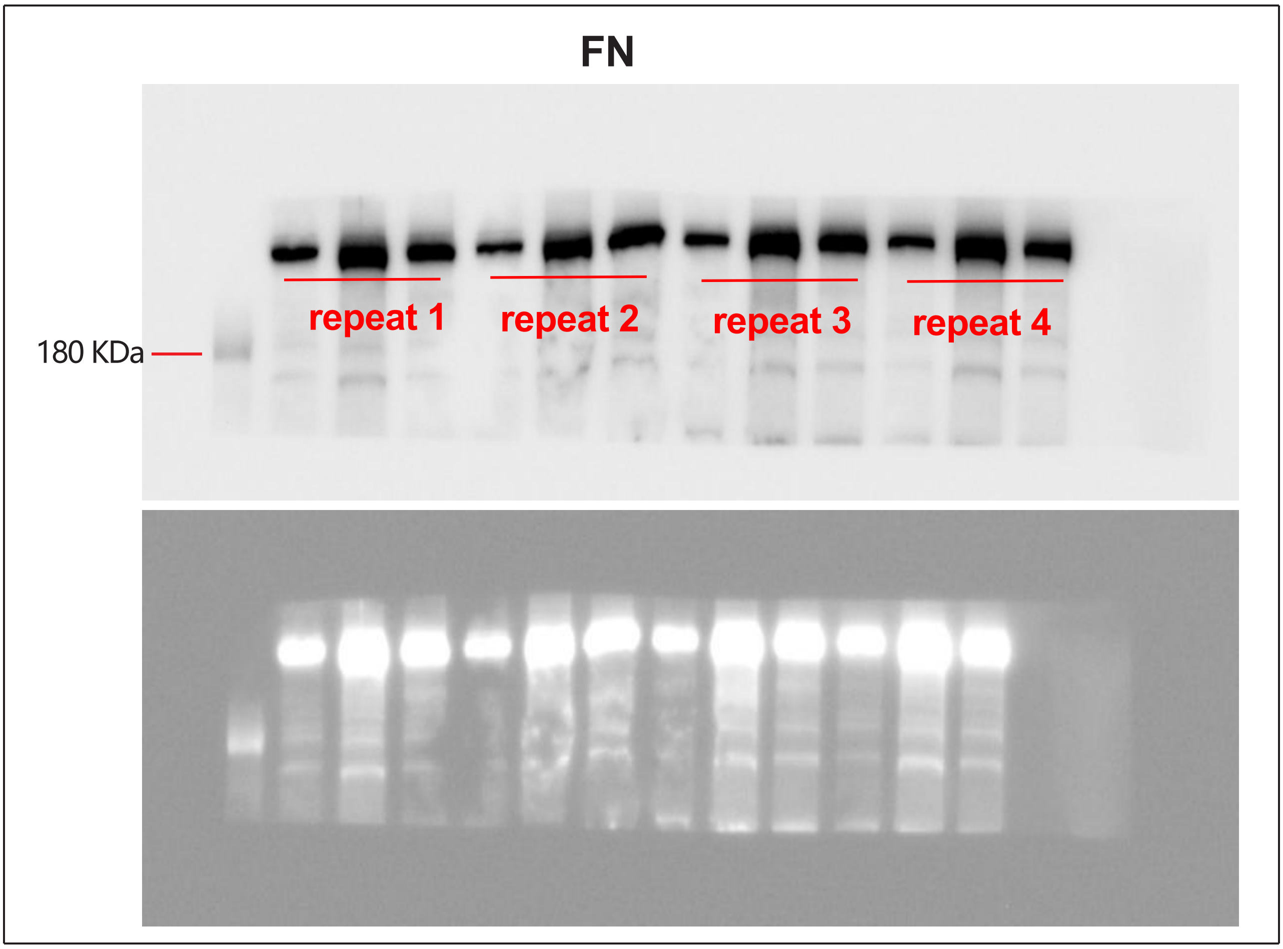

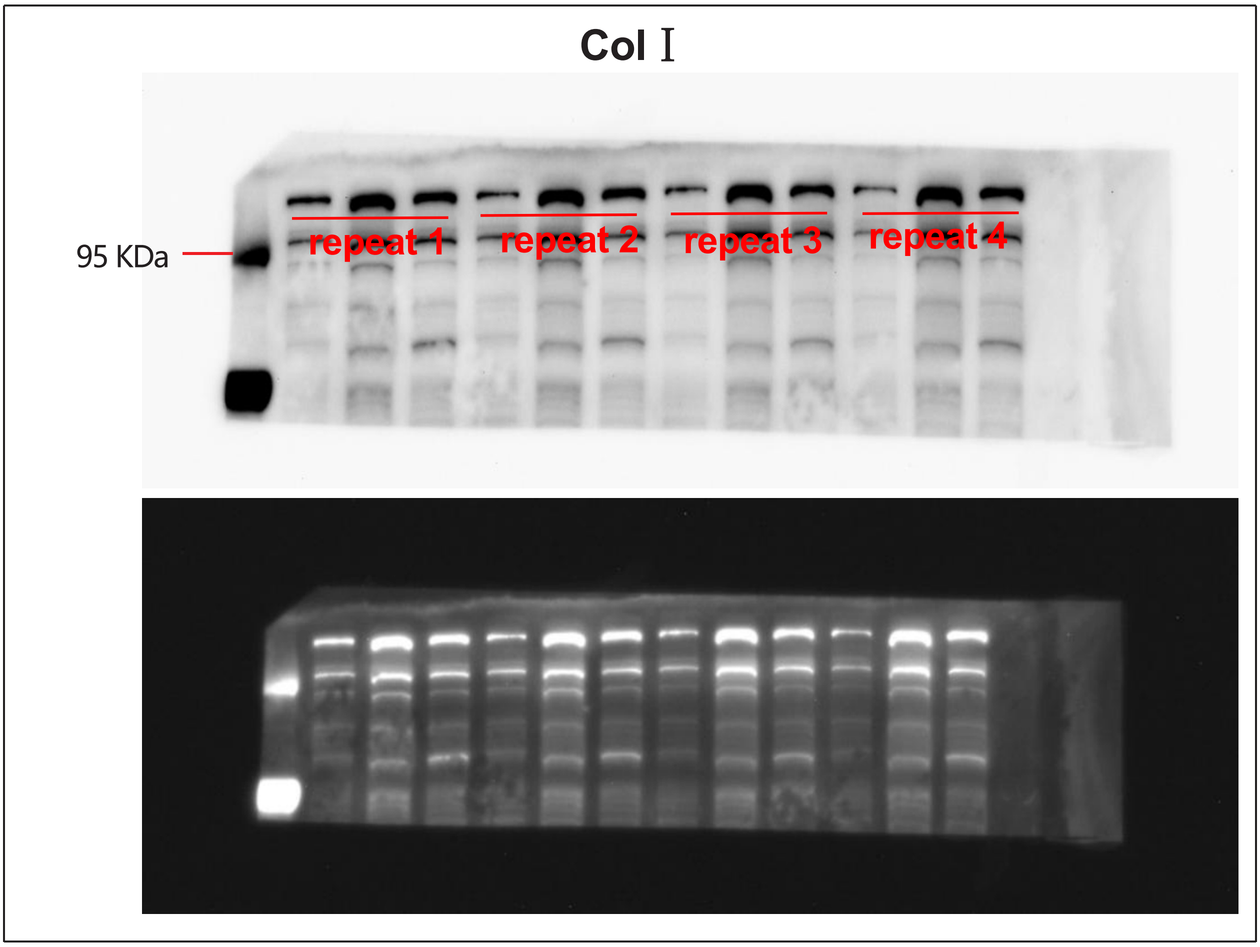

Supplementary Material

Figure 4B
The groups from left to right are: control, model, FSG.

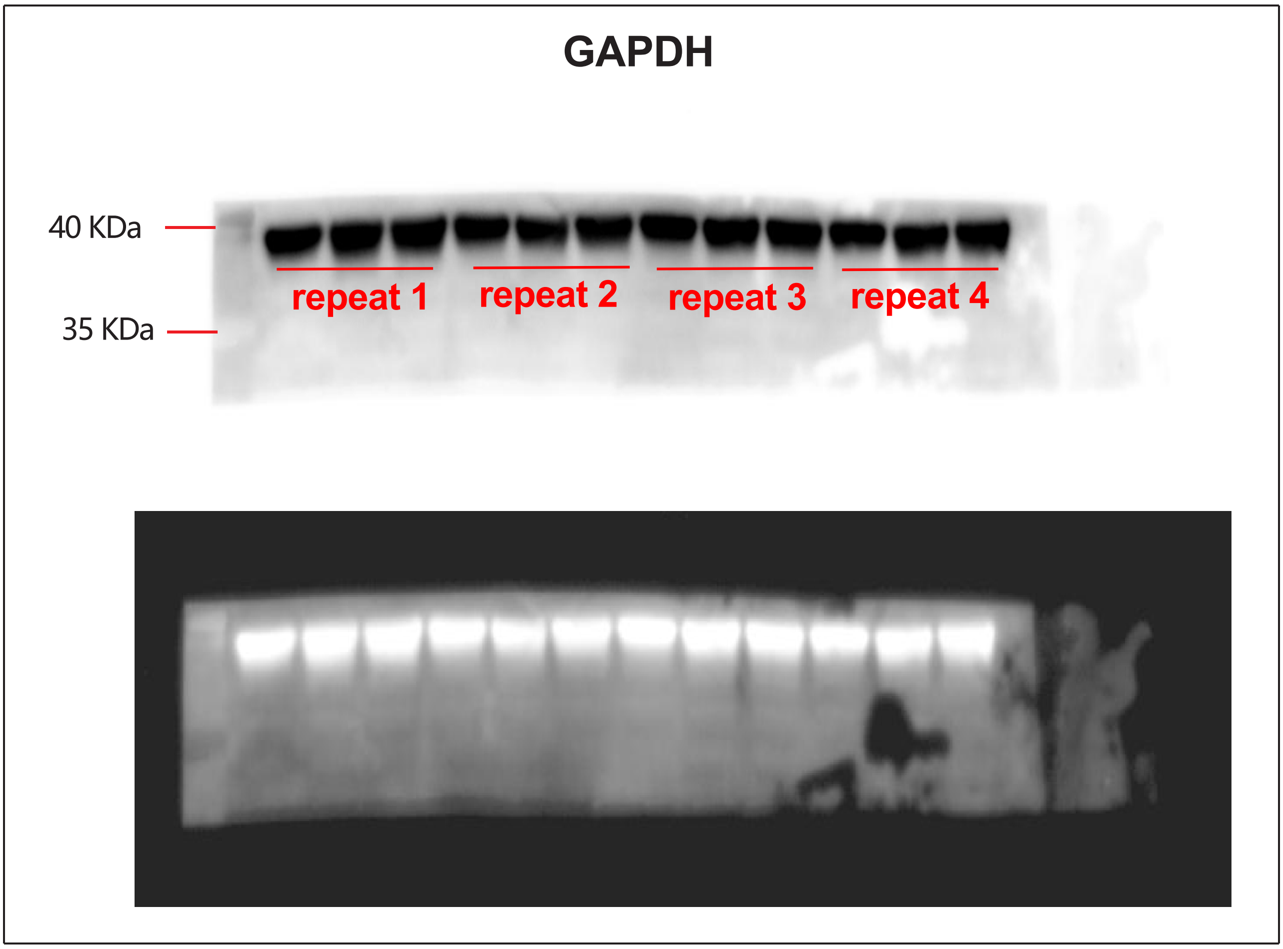

Supplementary Material

Figure 6A
The groups from left to right are: control, model, FSG.

p-mTOR

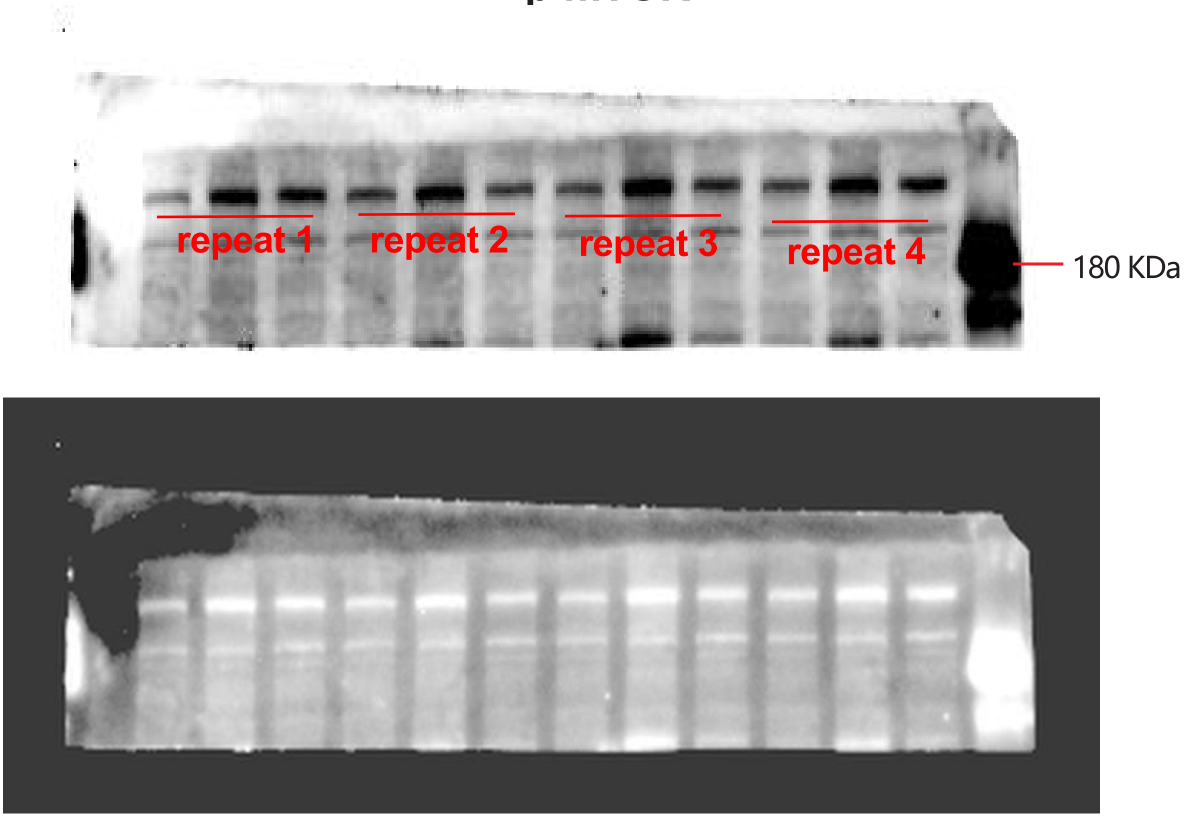

mTOR

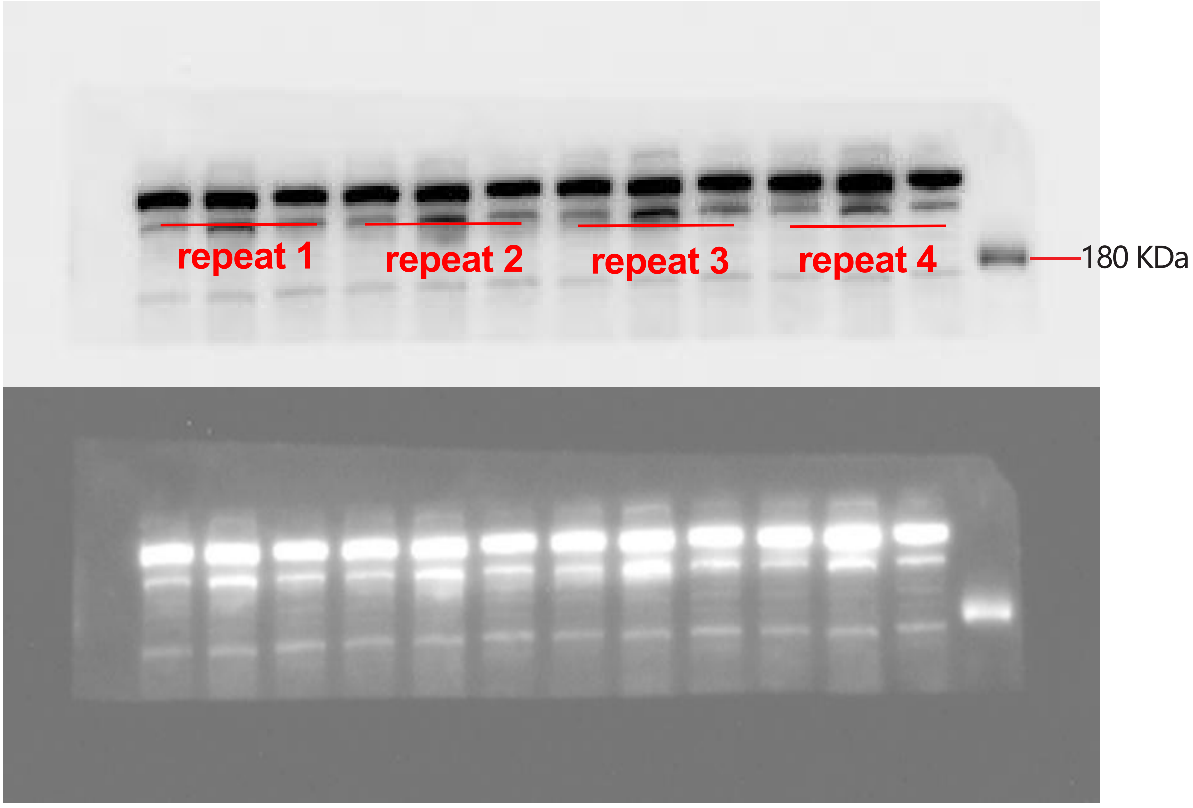

Supplementary Material

Figure 6A
The groups from left to right are: control, model, FSG.

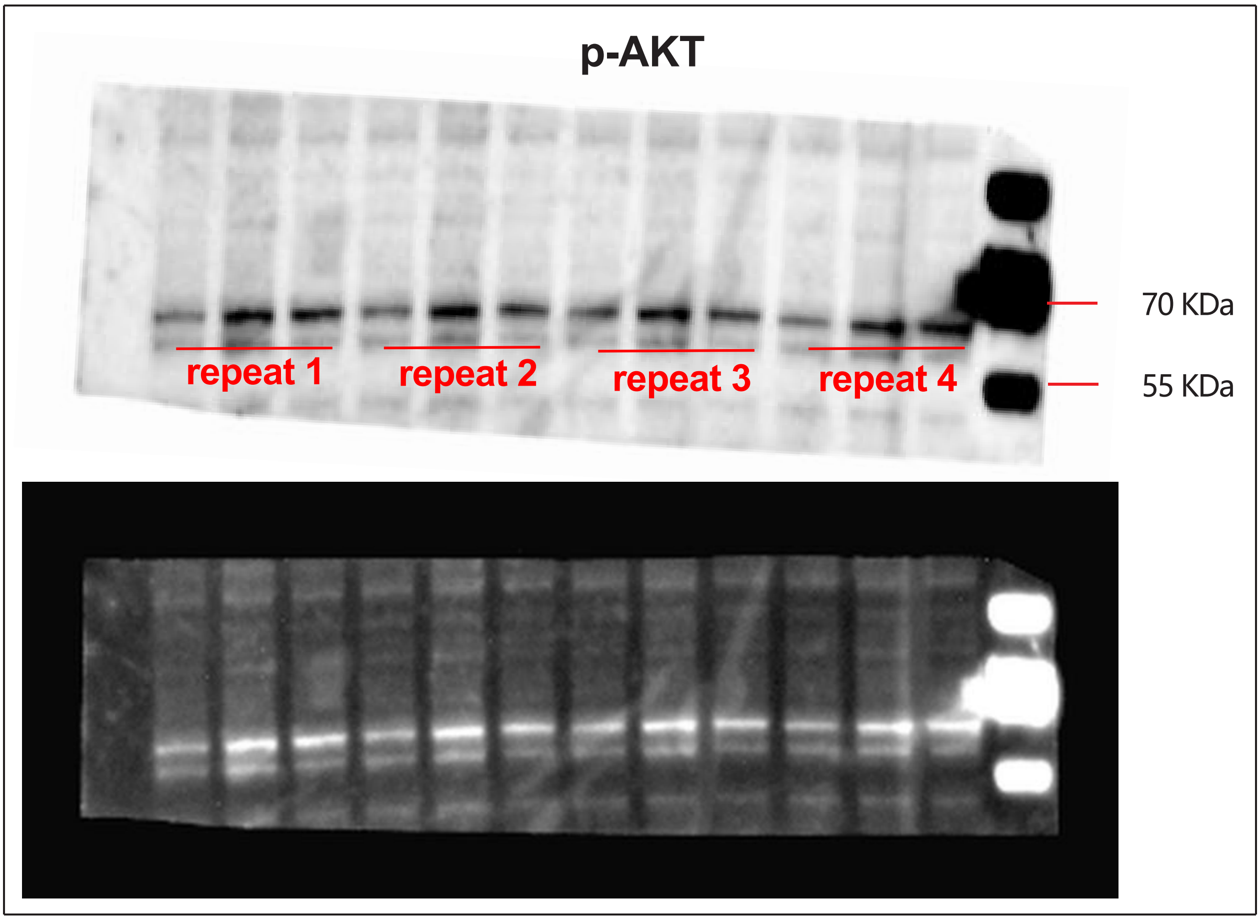

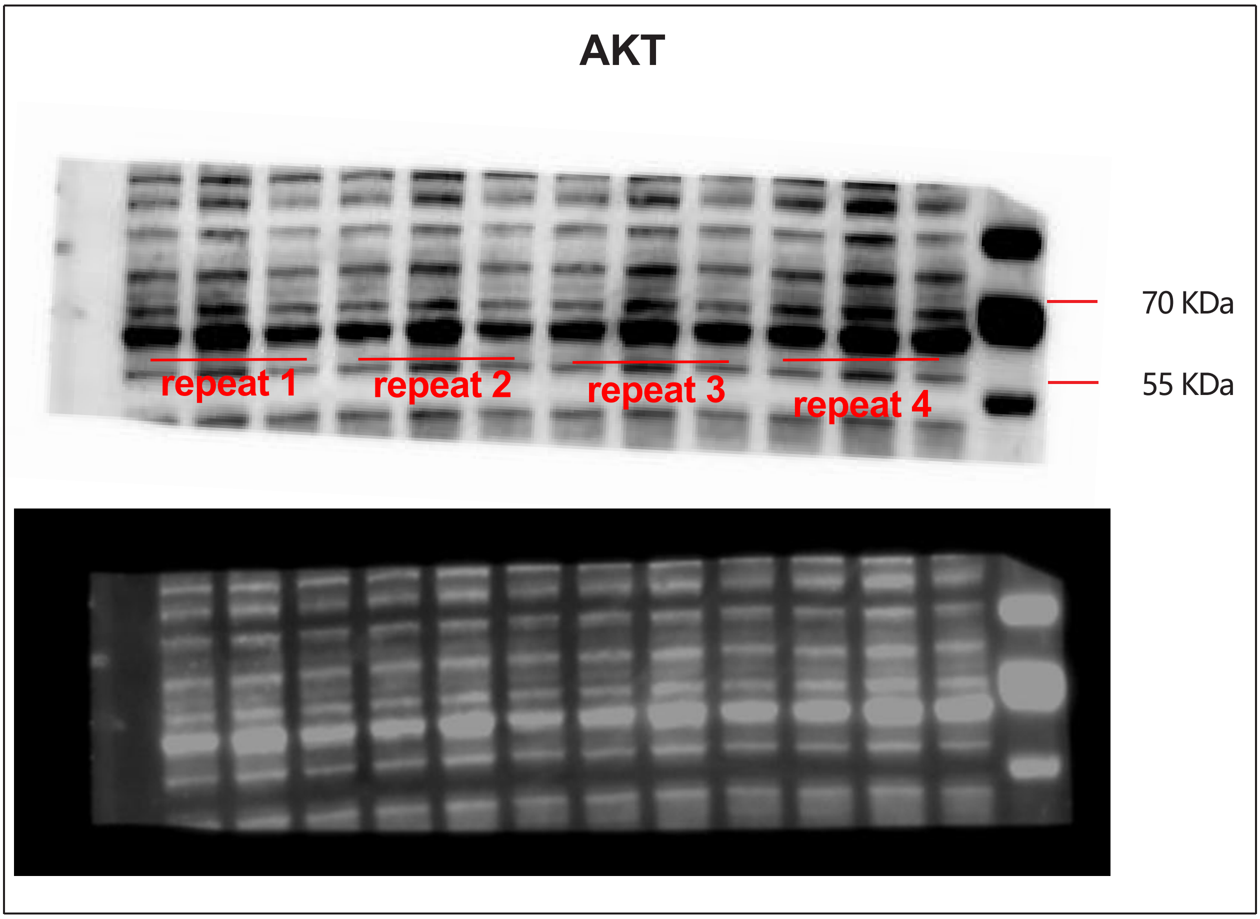

Supplementary Material

Figure 6A
The groups from left to right are: control, model, FSG.

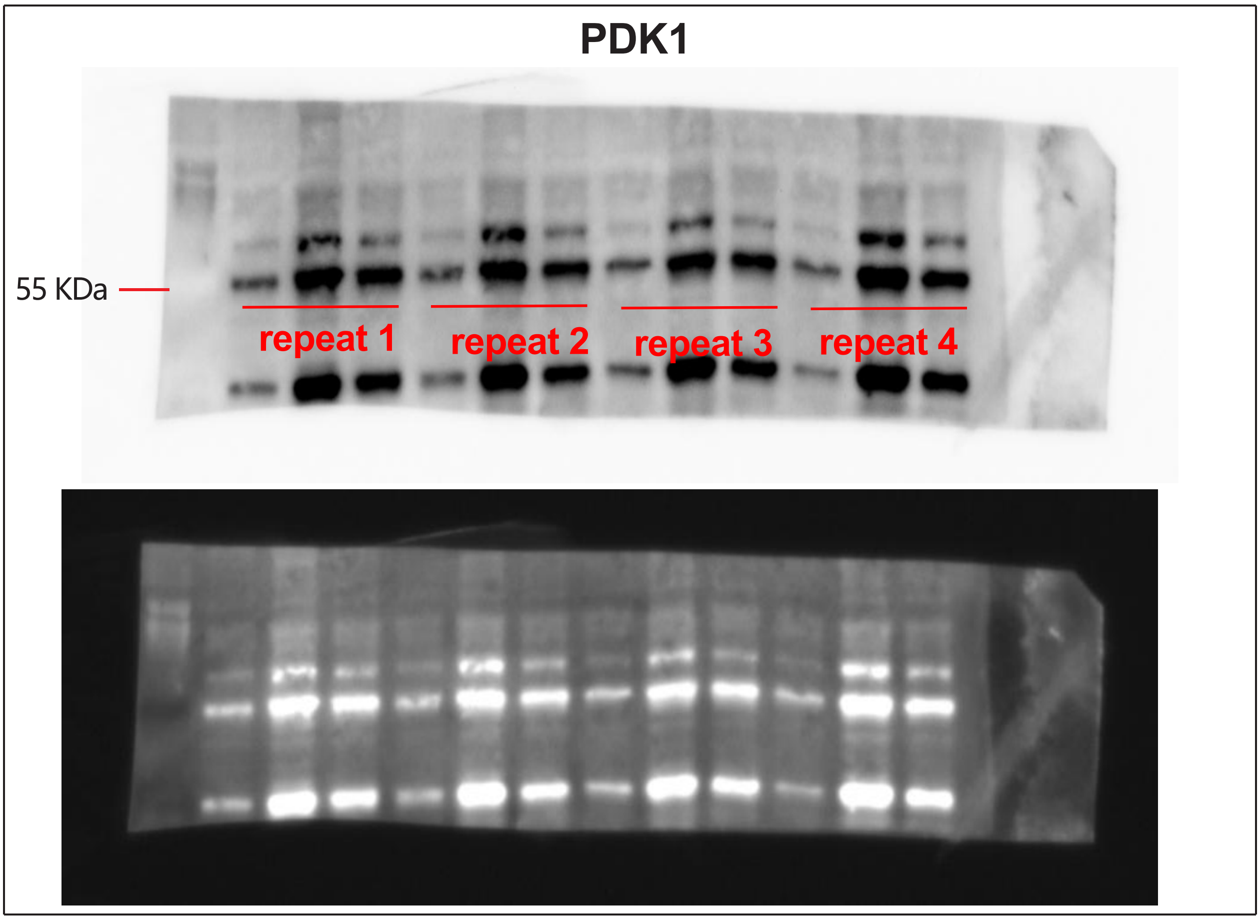

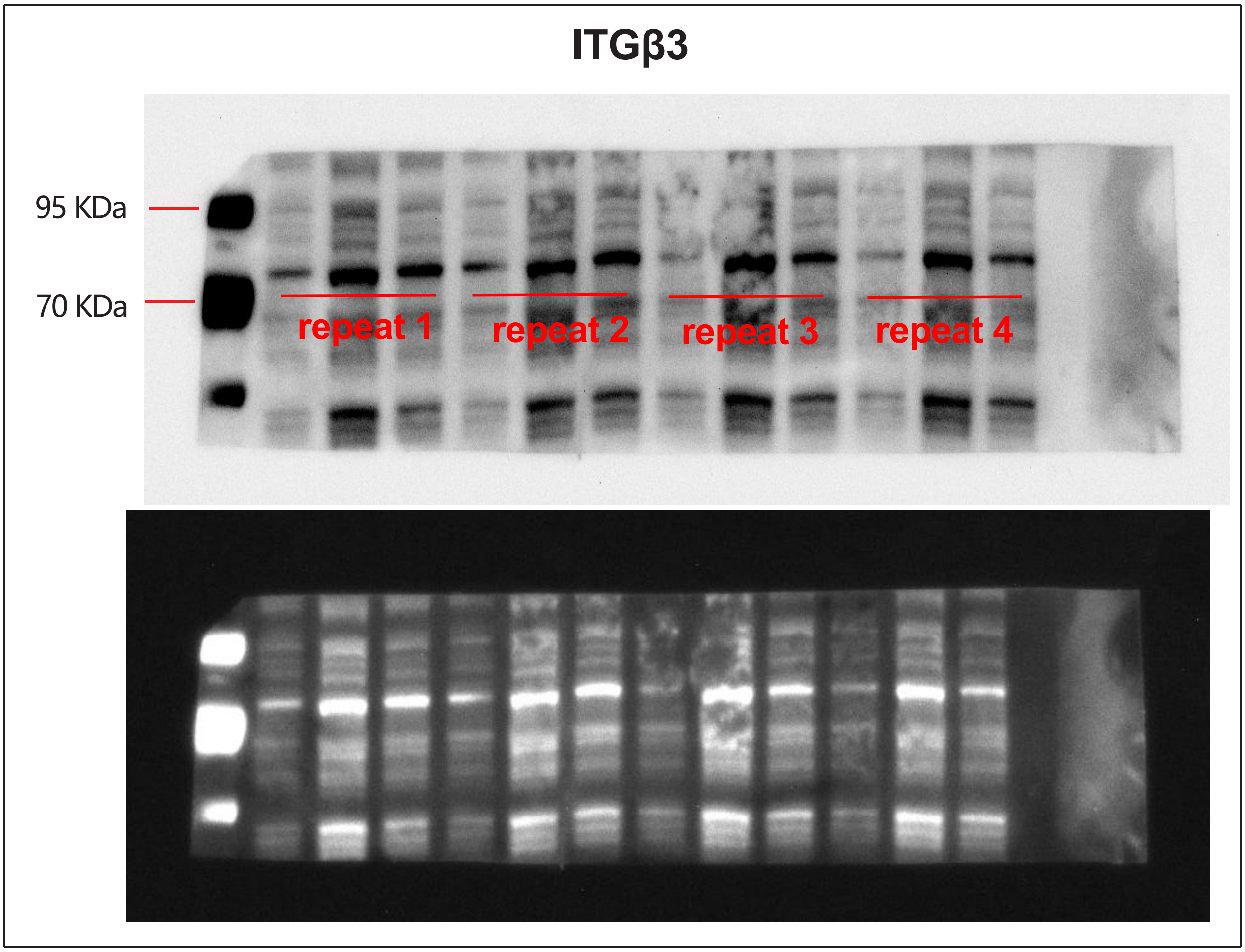

Supplementary Material

Figure 6A

The groups from left to right are: control, model, FSG.

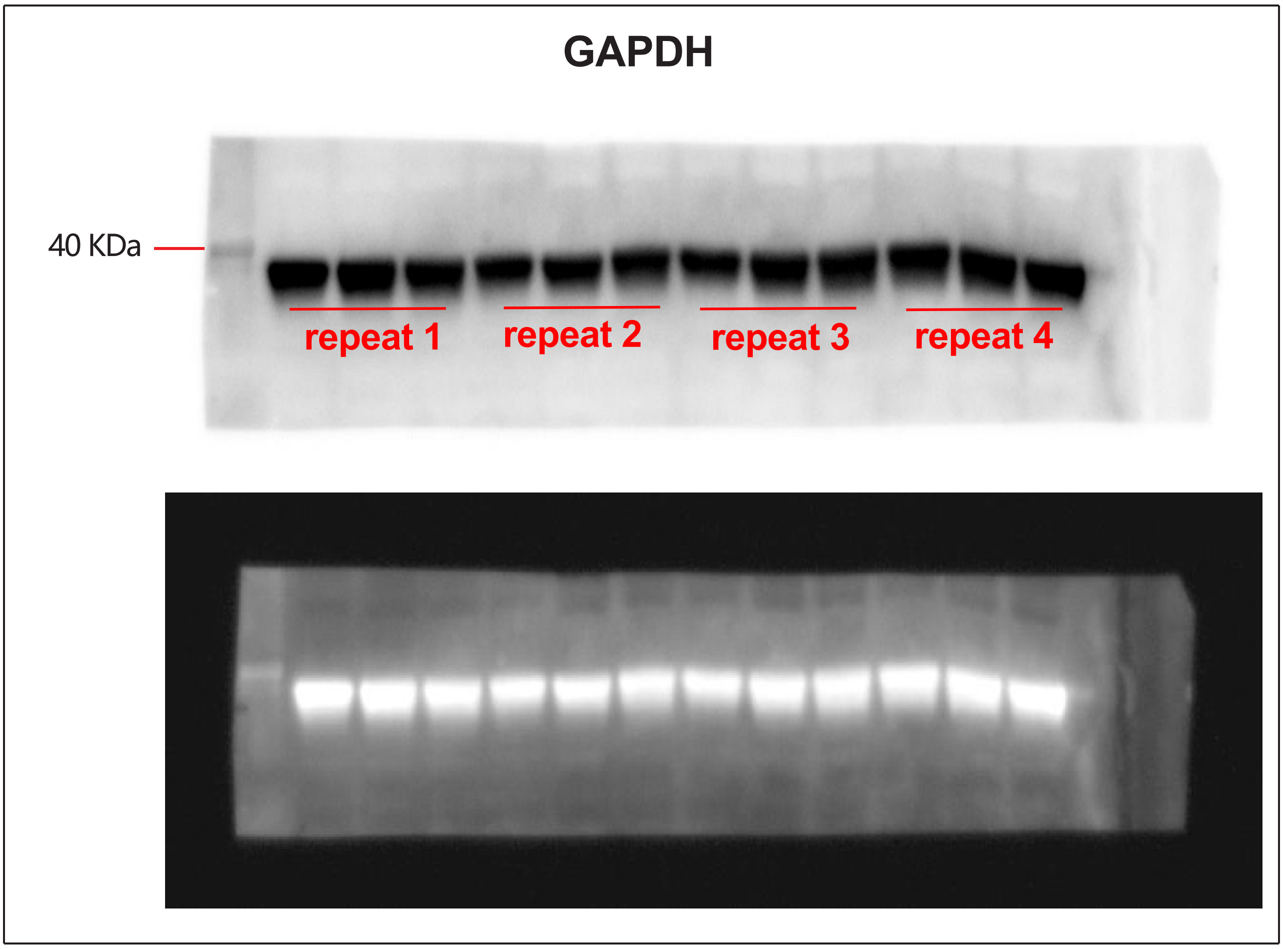

Supplement: Supplementary file 10 — Supplementary Material 10. [file 12906_2026_5333_MOESM10_ESM.pdf]
